# Multi-dimensional leaf phenotypes reflect root system genotype in grafted grapevine over the growing season

**DOI:** 10.1093/gigascience/giab087

**Published:** 2021-12-29

**Authors:** Zachary N Harris, Mani Awale, Niyati Bhakta, Daniel H Chitwood, Anne Fennell, Emma Frawley, Laura L Klein, Laszlo G Kovacs, Misha Kwasniewski, Jason P Londo, Qin Ma, Zoë Migicovsky, Joel F Swift, Allison J Miller

**Affiliations:** Department of Biology, Saint Louis University, 3507 Laclede Avenue, St. Louis, MO 63103-2010, USA; Donald Danforth Plant Science Center, 975 N. Warson Road, St. Louis, MO 63132-2918, USA; Division of Plant Sciences, University of Missouri, 135 Eckles Hall, Columbia, MO 65211, USA; Department of Biology, Saint Louis University, 3507 Laclede Avenue, St. Louis, MO 63103-2010, USA; Donald Danforth Plant Science Center, 975 N. Warson Road, St. Louis, MO 63132-2918, USA; Department of Horticulture, Michigan State University, East Lansing, MI 48824, USA; Department of Computational Mathematics, Science and Engineering, Michigan State University, East Lansing, MI 48824, USA; Department of Agronomy, Horticulture & Plant Science, South Dakota State University, Brookings, SD 57006, USA; Department of Biology, Saint Louis University, 3507 Laclede Avenue, St. Louis, MO 63103-2010, USA; Donald Danforth Plant Science Center, 975 N. Warson Road, St. Louis, MO 63132-2918, USA; Department of Biology, Saint Louis University, 3507 Laclede Avenue, St. Louis, MO 63103-2010, USA; Donald Danforth Plant Science Center, 975 N. Warson Road, St. Louis, MO 63132-2918, USA; Department of Biology, Missouri State University, 901 S. National Avenue, Springfield, MO 65897, USA; Division of Plant Sciences, University of Missouri, 135 Eckles Hall, Columbia, MO 65211, USA; Grape Genetics Research Unit, United States Department of Agriculture - Agricultural Research Service, Geneva, NY, 14456, USA; Department of Biomedical Informatics, The Ohio State University, 1585 Neil Ave, Columbus, OH 43210, USA; Department of Plant, Food, and Environmental Sciences, Faculty of Agriculture, Dalhousie University, Truro, NS B2N 5E3, Canada; Department of Biology, Saint Louis University, 3507 Laclede Avenue, St. Louis, MO 63103-2010, USA; Donald Danforth Plant Science Center, 975 N. Warson Road, St. Louis, MO 63132-2918, USA; Department of Biology, Saint Louis University, 3507 Laclede Avenue, St. Louis, MO 63103-2010, USA; Donald Danforth Plant Science Center, 975 N. Warson Road, St. Louis, MO 63132-2918, USA

## Abstract

**Background:**

Modern biological approaches generate volumes of multi-dimensional data, offering unprecedented opportunities to address biological questions previously beyond reach owing to small or subtle effects. A fundamental question in plant biology is the extent to which below-ground activity in the root system influences above-ground phenotypes expressed in the shoot system. Grafting, an ancient horticultural practice that fuses the root system of one individual (the rootstock) with the shoot system of a second, genetically distinct individual (the scion), is a powerful experimental system to understand below-ground effects on above-ground phenotypes. Previous studies on grafted grapevines have detected rootstock influence on scion phenotypes including physiology and berry chemistry. However, the extent of the rootstock's influence on leaves, the photosynthetic engines of the vine, and how those effects change over the course of a growing season, are still largely unknown.

**Results:**

Here, we investigate associations between rootstock genotype and shoot system phenotypes using 5 multi-dimensional leaf phenotyping modalities measured in a common grafted scion: ionomics, metabolomics, transcriptomics, morphometrics, and physiology. Rootstock influence is ubiquitous but subtle across modalities, with the strongest signature of rootstock observed in the leaf ionome. Moreover, we find that the extent of rootstock influence on scion phenotypes and patterns of phenomic covariation are highly dynamic across the season.

**Conclusions:**

These findings substantially expand previously identified patterns to demonstrate that rootstock influence on scion phenotypes is complex and dynamic and underscore that broad understanding necessitates volumes of multi-dimensional data previously unmet.

## Background

High-throughput data acquisition has afforded unprecedented capacity to quantify and understand plant form and function. Recent advances in imaging and computation have expanded our ability to measure plant traits or phenotypes [1,2] and to extend those comprehensive measurements into latent space phenotypes [3]. Now broadly known as phenomics, this burgeoning field is characterized as the acquisition and analysis of high-dimensional phenotypic data at different hierarchical levels [4,5], often with an eye toward multiscale data integration. A holistic and hierarchical approach to plant phenotypic variation affords unique insights into plant evolution and how plants change over development and in response to environmental cues and horticultural manipulation.

A fundamental question in plant biology is how root systems influence phenomic variation in above-ground shoot systems including leaves, flowers, and fruits. Grafting, a common horticultural manipulation that joins the shoot system of one individual (the scion) with the root system of another individual (the rootstock), is commonly used in crop species to confer favorable phenotypes to commercial scions [6], including enhanced disease resistance [7,8], fruit quality, plant form [9], response to water stress [10], and growth on particular soils [11,12]. Because grafting often uses clonally propagated materials, it is possible to manipulate and replicate different combinations of root systems and shoot systems, offering a valuable experimental system in which root system effects on shoot system phenotypes can be evaluated.

The European grapevine (*Vitis vinifera*) is among the most economically important grafted crops in the world. Grapevines are cultivated primarily for fruits used to make wine and juice, as well as for table grape and raisin production. Grafting in grapevines became widespread in the mid-1800s following the accidental introduction of the root-feeding aphid phylloxera from its native North America into Europe, where it began attacking the roots of European grapevines [13]. Because European grapevines often do not survive phylloxera infestation, in regions where phylloxera has been introduced most grapevine cultivation consists of European grapevines grafted to rootstocks derived from phylloxera-resistant North American *Vitis* species including *Vitis berlandieri, Vitis riparia*, and *Vitis rupestris*, and their hybrid derivatives. In addition to grapevines, >70 major perennial crops are grafted including many fruit trees and vines [9]. Grafting decouples the breeding of shoot systems and root systems, with selection in plants targeted for use as scions focusing primarily on fruit phenotypes, and selection in plants targeted for use as rootstocks focused on below-ground biotic and abiotic stress resistance, as well as their effects on shoot system phenotypes.

The effects of grafting in grapevine show a remarkable breadth of scion response patterns. For example, a study of *V. vinifera* cv. “Cabernet Sauvignon” grafted to different rootstocks identified transcriptome reprogramming in the scion of grafted plants; this seemed to be a general effect of grafting to a rootstock and was not rootstock specific [14]. In contrast, other studies have found signatures of rootstock genotype in the transcriptome in early berry development, although this distinction was lost in later development [15,16], but see [17]. Comprehensive phenomic analyses, including those that link transcriptome data with other high-throughput phenotyping assays, offer an opportunity to expand understanding of rootstock effects on grapevine shoots. In one study, leaves of the *V. vinifera* cultivar “Gaglioppo” showed variation in stilbene and abscisic acid concentrations owing to rootstock genotype, as well as differences in transcriptional profiles [18]. Likewise, gene expression, ion concentrations, and leaf shape in the cultivar “Chambourcin” varied in response to rootstock genotype [19]. Collectively, these studies suggest that the effects of grafting are diverse and may vary over the course of vine development. However, to date few studies have surveyed multiple high-dimensional scion phenotypes to understand rootstock influence on shoot system phenotypes over the course of the growing season or the extent to which grafting effects on the scion covary with one another.

Leaves are the photosynthetic engine of the organism and a primary site for perception and response to environmental change. Grapevine leaves have been used for centuries as markers of species and cultivar delimitation, developmental variation, disease presence, and nutrient deficiency [20,21]. More recently, analysis of grapevine leaf morphology has identified the genetic architecture of leaf shapes [22], developmental patterns across the season [23], and signatures of evolution in the grapevine genus [24]. Grapevine leaves respond to stress through gas and water exchange with the atmosphere [25,26] and have been shown to differentially partition the ionome depending on their position on the shoot [19] and their rootstock genotype [19,27,28]. The volume of work on grapevine leaves provides a foundation for the analysis of phenomic variation in a vineyard over a season in response to grafting.

In this study, we investigate effects of grafting on high-dimensional leaf phenotypes of the hybrid cultivar “Chambourcin” over the course of the growing season. We quantify leaf elemental (ion) concentrations, metabolite abundance, gene expression, shape, and vine physiology in a replicated rootstock trial where the hybrid grapevine cultivar Chambourcin is growing ungrafted and grafted to 3 different rootstocks. The 4 root-shoot combinations (Chambourcin ungrafted, Chambourcin grafted to 3 different rootstocks) are replicated 72 times in a randomized block experimental design with an irrigation treatment (Supplementary Fig. S1). Phenotypic data, data that describe variation for a particular trait within a particular modality, were collected either on the full 288-vine set (ion concentrations, leaf shape) or on a subset of 72 vines (the 72-vine set; metabolite abundance, gene expression, vine physiology). Using data collected at 3 time points that span the growing season (anthesis, veraison, and harvest), we show that all phenotyping modalities (ionomic, metabolomic, transcriptomic, morphometric, and physiology phenotypes) reflect subtle but ubiquitous responses to grafting and rootstock genotype. Rootstock effects on shoot system phenotypes were often dynamic across the season, suggesting that accounting for seasonal variation could enhance our understanding of grafting effects in viticulture.

## Data Description

### Leaf ionomics

The ionome describes the elemental composition of a tissue at a particular time point [29]. Three leaves per vine were collected from the 288-vine set at 3 seasonal time points: anthesis (roughly mid-May), veraison (roughly late July), and harvest (roughly mid-September). Leaves were sampled from a single shoot and included the youngest fully opened leaf at the shoot tip, the approximate middle leaf, and the oldest leaf at the shoot base. Teams were deployed in the vineyard so that multiple vineyard rows were being sampled concurrently. As such, “block” represented unmeasured spatial variation but did not strictly correlate with time of sampling owing to the nature of sampling (see Methods). Whole leaves were placed in zip-lock bags in the field and stored in a cooler on ice packs, scanned for leaf shape analysis in the laboratory (see Leaf Shape), and then dried in coin envelopes at 50°C for 1–3 days for elemental analysis. Between 20 and 100 mg of leaf tissue was acid digested and 20 ions were quantified using inductively coupled plasma mass spectrometry (ICP-MS) following standard protocol of the Donald Danforth Plant Science Center (DDPSC) Ionomics Pipeline [30,31]. Ion quantifications were corrected for internal standard concentrations, instrument drift, and by initial sample mass. The output of the Pipeline contained estimated concentrations of each of the following 20 elements: aluminum, arsenic, boron, calcium, cadmium, cobalt, copper, iron, potassium, magnesium, manganese, molybdenum, sodium, nickel, phosphorus, rubidium, sulfur, selenium, strontium, and zinc. For each ion concentration, we computed *z*-score distributions and used those values as the basis for linear models. Following convention, non-standardized values were used for machine learning analysis.

### Leaf metabolomics

The metabolome comprises small mostly organic molecules present in a tissue and represents a catalogue of the products of metabolic processes [32,33]. Metabolomic analysis was completed at veraison (the onset of fruit ripening) and immediately prior to harvest for the 72-vine set. For each vine, 3 mature leaves were sampled from the middle of a single shoot and immediately flash frozen in liquid nitrogen in the field to capture the metabolic state of the leaves when attached to the vine. Leaves were sampled by a single team near midday in row and block order, ensuring that “block” captured both unmeasured spatial variation and temporal variation over the sampling window (see Methods). Frozen leaves were transported to the University of Missouri Enology Lab on dry ice and stored at −80°C. Following the protocol of [34], whole leaves were manually ground in liquid nitrogen with a mortar and pestle, 0.5 g of powder was weighed into a centrifuge tube, and 1.5 mL of 1:1 methanol:acetonitrile was added. Samples were vortexed to suspend leaf particles and sonicated for 20 minutes in an ice bath. After extraction, samples were centrifuged for 10 minutes at 3,000*g*and filtered with a 0.22 PTFE syringe filter into a 1.5-mL sample vial before injecting into a Waters XEVOTM QToF LCMS system (Waters Corporation, Milford, MA, USA). Chromatographic separation was achieved using a Waters Acquity TM Ultra Performance LC H-Class system (Waters Corporation, Milford, MA, USA) equipped with Waters Acquity BEH C18 column (2.1 × 150 mm and 1.7 μm particle size) and a diode array detector. Samples were injected in random order across the sampling periods. The injection volume was set at 2.5 μL and the flow rate was set at 0.4 mL/min. The mobile phase consisted of 0.1% formic acid in water (solvent A) and 0.1% formic acid and 5% water in acetaldehyde (solvent B) and the gradient was as follows: 100% A for 0.5 min; 0.5–18 min increased to 99% B; 18–19 min held at 99% B; mobile phase was re-equilibrated for 2 min between runs. Diode array was monitored at 225–500 nm. Mass spectrometry was performed on a XevoTM QTof (Waters Corporation, Milford, MA, USA). The electrospray ionization (ESI) was operated in both positive and negative ionization modes in separate runs. The scan range was set as *m*/*z* 50–1,500 with 0.2 sec accumulation time. MS settings were as follows: capillary voltage was 2.5 kV; cone voltage ramped from 20 to 40 V; collision energy was set to 6 V; detector voltage was set to 1950 V; desolvation gas was set to 1000 L/hour; cone gas was set to 50 L/hour; source temperature was 120°C and desolvation temperature was set at 550°C.

LC-MS instrument files were converted to .cdf format and uploaded to XCMS online [35] for chromatogram normalization and feature detection via “single job” parameters. The 661 identified metabolomic features were used as the basis of a principal component (PC) analysis. The top 20 PCs were treated as distinct phenotypes to model according to the experimental design. In PCs that varied significantly by rootstock, features that loaded >1.96 standard deviations (SD) above or below the mean were fit independently with the same model design.

### Leaf gene expression

The youngest fully opened leaves on 2 shoots were collected from each plant of the 72-vine set (see Study Design). The 2 leaves, which were distinct from leaves used for ionomics, leaf shape, metabolomics, and physiology data collection, were pooled for RNA sequencing. Leaves were sampled by a single team near midday between 10:00 AM and 2:00 PM in row order, ensuring that “block” and “row” accounted for unmeasured spatial variation and temporal variation over the sampling window (see Methods). Samples were sequenced using 3′-RNAseq, a method ideal for organisms with reasonably characterized reference genomes [36]. Total RNA was extracted from plant tissues using the Sigma Spectrum Plant Total RNA kit with modification of the addition of 2% PVP40 to the extraction buffer to decrease phenolic inhibitors. All RNA extractions were checked for quality control using a Nanodrop. Sequencing was conducted using the Illumina NextSeq500 platform, which returned single-end 86-bp reads. To accommodate the large number of samples in this study, we opted to obtain fewer reads per sample, which might have limited our ability to detect differential expression in genes with low expression levels. The first 12 nucleotides from each read were trimmed to remove low-quality sequences using Trimmomatic (options: HEADCROP:12 [37]). Low-quality trimmed reads were additionally identified on the basis of overrepresentation of *k*-mers and removed using BBduk (April 2019 release) [38]. Trimmed and quality-controlled reads were mapped to the 12Xv2 reference *V. vinifera* genome [39,40] using STAR (v2.7.2b) [41] with default alignment parameters. RNAseq read alignments were quantified using HTSeq-count (v0.11.2) [42] and a modified version of the VCost.v3 reference *V. vinifera* genome annotation [40]. To capture misannotated gene body boundaries in the genome, all gene boundaries in the annotation were extended 500 bp.

Variation in gene expression was assessed using 2 methodologies. First, we identified individual genes that responded to specific factors in the experimental design using DESeq2 (v1.24.0) [43]. Each gene was fit with the model “∼ Block + Irrigation + Phenology_Rootstock,” where the “Phenology_Rootstock” model term was used to understand the potential interaction of phenology and rootstock. Genes were filtered to a gene set that included only genes with a normalized count ≥2 in ≥5 samples. To check the validity of our expression results, we assayed 2 classes of housekeeping genes (Ubiquitin-domain and actin-family) and 8 previously annotated circadian genes [44] (Supplementary Fig. S2). Differentially expressed genes were identified for each pairwise contrast in the model. Second, we used principal component analysis (PCA) to collapse variation in co-expressed genes into fewer dimensions. Normalized count-filtered genes from DESeq2 were transformed using the variance stabilizing transformation (VST [45]) and input into a PCA. We then analyzed the top 100 PCs in the context of the broader experimental design. We previously showed that the transcriptome varied by the time of collection and was potentially interacting with the rootstock effect [19]. Moreover, the other modalities in this study point to weak if any effects from the irrigation treatment (see Supplementary Note S1). Owing to the nature of the vineyard design, we could not identify both irrigation and time effects (marked by row) in a single model (irrigation and row are collinear; see Study Design). To approximate the impact from time of collection (row) in the vineyard on gene expression, linear models were first fit to remove variation imparted by irrigation from each of the top 100 PCs. The residuals were then used as the basis for linear models and machine learning analysis.

### Leaf shape

All leaves from a single shoot directly emerging from a trained cordon were collected from each vine in the 288-vine set at anthesis and veraison. At harvest, we collected only the oldest (first emerging leaf), middle (estimated from the middle of a whole shoot), and youngest (smallest fully emerged leaf at the shoot tip, >1 cm). Leaves were collected approximately in row order (from south to north) and stored in a cooler. Each leaf was imaged using an Epson DS-50,000 scanner in color against a white background at 1,200 DPI and written as JPEG formatted images. Following scanning of leaves for leaf shape analysis, the oldest, middle, and youngest leaves were dried and used to estimate leaf elemental composition (see Ionomics). Because the leaf shape samples and ionomics samples were identical, “block” represented unmeasured spatial variation but did not strictly correlate with time of sampling (see Methods). While all leaves were collected from a single shoot, only the oldest, middle, and youngest leaves were used in this analysis.

We assessed leaf shape using generalized Procrustes analysis (GPA) of landmarks. For the 3 leaves per vine used in leaf shape analysis, 17 homologous landmark features were identified [22]. The GPA-rotated coordinate space was used for all subsequent statistical analysis including PCA in order to summarize variation in leaf shape [46]. From the PCA, we extracted the top 20 PCs and fit linear models and machine learning models to describe variation.

### Vine physiology

Intracellular CO_2_ concentration, stomatal conductance, and leaf transpiration rate were measured at midday (each measured simultaneously between 10:00 AM and 1:00 PM) on one fully expanded sun-exposed leaf for each of the vines in the 72-vine set. Physiology measurements were taken in row order, ensuring that “block” correlated with temporal variation over the sampling window. Measurements were taken using an LI-6400XT Portable Photosynthesis system coupled with a pulse amplitude–modulated (PAM) leaf chamber fluorometer (Li-Cor, Inc., Lincoln, NE, USA) with the following parameters: incident photosynthetic photo flux density level of 1,000 µmol/m^2^/s generated by a red LED array and 10% blue light to maximize stomatal opening, CO_2_ mixer of 400 µmol/s, fixed flow of 300 µmol/s, and ambient leaf and block temperature. Soil moisture was measured for each plant in the 72-vine set using a fieldScout TDR 300 Moisture meter equipped with 20-cm rods (Spectrum Technologies, Inc., Aurora, IL, USA). Midday stem water potential was measured using a pressure bomb/chamber (PMS Instrument Co., Albany, OR, USA) after enclosing the leaves in an aluminum foil bag for ≥15 minutes to equilibrate the water potential of the xylem in the stem to that attached leaf (for a discussion on equilibration time, see [47, 48]).

## Analyses

### Leaf ionome

**Figure 1:**
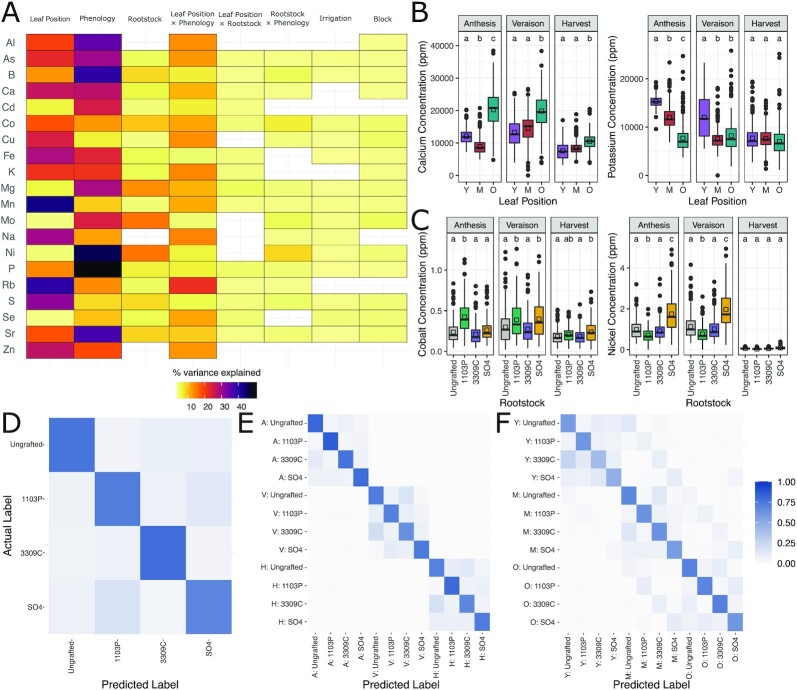
The ionome shows strong signal from rootstock genotype, leaf position, and phenological stage. **(A)** Percent variation captured in linear models fit to each of 20 ions measured in the ionomics pipeline. Presence of a cell indicates the model term (top) was significant (FDR; padj < 0.05) for that ion (left). **(B)** Example ions shown to vary significantly by the interaction of leaf position (Y = Youngest, M = Middle, O = Oldest) and phenological stage in parts per million. Boxes are bound by 25th and 75th percentile with whiskers extending 1.5 IQR from the box. Dots indicate outliers. Significant changes are indicated by letters above boxes and are only meant for comparison within each phenological stage. Group means are displayed with black squares. (**C**) Example ions shown to vary significantly by the interaction of rootstock genotype and phenological stage in parts per million. Significant changes are indicated by letters above boxes and are only meant for comparison within each phenological stage. Boxes are bound by 25th and 75th percentile with whiskers extending 1.5 IQR from the box. Group means are displayed with black squares. Dots indicate outliers. (**D**) Standardized heat map for out-of-bag (OOB) predictions by a random forest trained to predict rootstock genotype, **(E)** the interaction between rootstock genotype by phenology, and **(F)** the interaction between rootstock genotype and leaf position.

To characterize the leaf ionome over the growing season, we sampled the youngest, middle, and oldest leaf from a single shoot from each of the vines within the 288-vine set at 3 phenological stages and measured the concentrations of 20 ions in each leaf individually. Bivariate correlations showed that ion concentrations are not independent of each other but that the strength and direction of relationships between ions vary with respect to phenological stage and leaf position (Supplementary Fig. S3). As such, we fit independent linear models to each ion. Leaf position, phenological stage, or the interaction of phenological stage and leaf position explained the highest amount of variation for most ions (Fig. 1A and B). Many ions significant for the interaction showed a clear signal of leaf position at anthesis and veraison, and either no explainable variation or muted variation at harvest. For example, calcium (Fig. 1B) varied with leaf position (22.7% variation explained; *P* < 1e−05), phenology (24.0%; *P* < 1e−05), and their interaction (7.4%, *P* < 1e−05). All possible pairwise combinations of leaf position were significantly different at anthesis, and both the youngest and middle leaves were different from the oldest leaves at veraison and harvest. In the case of potassium (Fig. 1B), significant variation was explained by leaf position (16.1%; *P* < 1e−05), phenology (19.6%; *P* < 1e−05), and their interaction (10.6%; *P* < 1e−05). However, post hoc comparisons of phenology-wise mean calcium concentrations showed that differences were present only at anthesis and veraison.

Rootstock genotype showed remarkable influence on the composition of the leaf ionome. All ions except aluminum, sodium, and zinc were significant for rootstock as a single fixed effect (Fig. 1A). Rootstock explained between 0.4% (rubidium; *P* = 3.2e−05) and 14.3% (nickel; *P* < 1e−05) of variation in ion concentrations (Fig. 1A). For some ion concentrations (such as cobalt and nickel), significant variation was explained by the interaction of rootstock and phenology; this pattern was observed mostly in ions that responded weakly to the interaction of leaf position and phenology. These ions showed similar patterns to the leaf position by phenology interaction where a clear signal was exhibited at anthesis and veraison then was either absent or muted at harvest. For example, cobalt was most abundant in 1103P-grafted vines at anthesis (Fig. 1C). At veraison, both 1103P-grafted and SO4-grafted had elevated concentrations compared to ungrafted and 3309-grafted vines. However, by harvest, cobalt concentration variation was muted and only SO4-grafted vines showed evidence of elevated concentration. Similarly, nickel showed significant variation partitioned into the rootstock by the phenology effect (Fig. 1C). Both anthesis and veraison show reduced nickel concentration in 1103P-grafted vines and elevated concentrations in SO4-grafted vines. However, at harvest, no comparisons are significant.

Machine learning on ion concentrations confirms that the leaf ionome contains a signature from the rootstock genotype and the interactions of rootstock genotype with phenology and leaf position. A random forest model trained to predict rootstock showed an overall accuracy of 75.2% (Fig. 1D). Ions important for this classification were nickel (mean decrease in accuracy [MDA] = 0.089), molybdenum (MDA = 0.058), and magnesium (MDA = 0.054), corroborating the rootstock term's significance in the linear models. Notably, when we trained a model to simultaneously predict rootstock and phenological stage, rootstock prediction accuracy increased appreciably (Fig. 1E). For example, the ability of the model to detect ungrafted vines (the balanced accuracy of ungrafted predictions) improved from 81.7% accuracy overall to 91.1% accuracy at anthesis and 85.9% at harvest. Generally, performance at veraison matched the rootstock-only model performance. The ions most important for this joint (rootstock/phenological stage) prediction were nickel (MDA = 0.167), phosphorus (MDA = 0.110), and strontium (MDA = 0.065). The rootstock by phenology model term was significant in the linear models for these ions but was not a largest descriptor of variation. The joint prediction of rootstock and leaf position performed substantially better than chance (*P* < 1e−05), but accounting for leaf position did not improve rootstock prediction as was the case in the joint prediction of rootstock and phenology (Fig. 1F). Ions important for this classification were sulfur (MDA = 0.051), rubidium (MDA = 0.051), and nickel (MDA = 0.049).

### Leaf metabolomics

**Figure 2:**
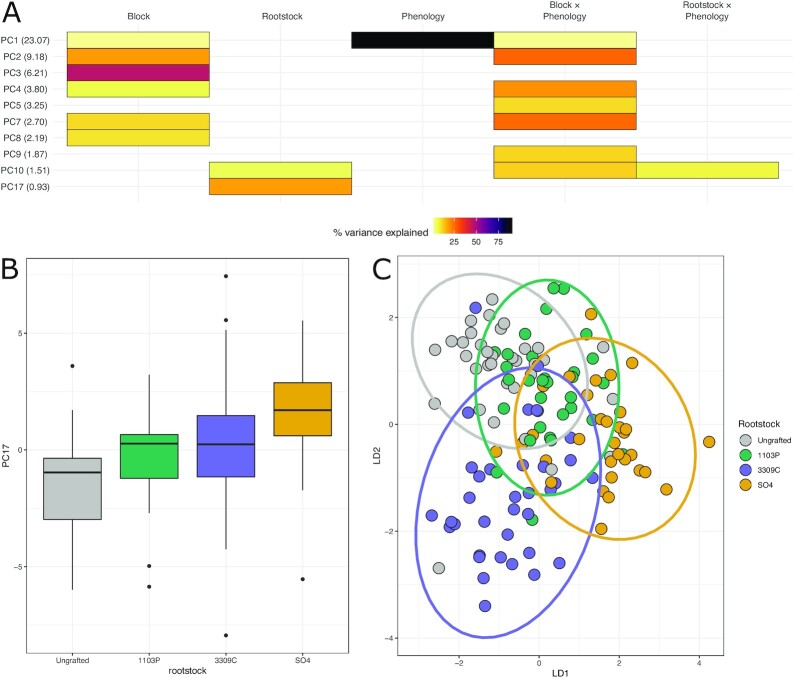
The metabolome is influenced by rootstock genotype, phenological stage, and time of sampling. **(A)** Percent variation captured in linear models fit to each of the top 20 principal components (PCs) of the metabolome (661 measured metabolites). Presence of a cell indicates the model term (top) was significant for that PC (left, percent variation explained by the PC in parentheses). **(B)** The distribution of projections onto PC17, the strongest captured rootstock effect in the metabolome. Boxes are bound by the 25th and 75th percentiles with whiskers extending 1.5 IQR from the box. Dots indicate outliers. **(C)** Projections of all samples into the first 2 dimensions of a linear discriminant space trained to maximize variation between rootstock genotypes.

We performed untargeted metabolomics on leaves from the 72-vine set at veraison and harvest, quantifying the concentrations of 661 metabolites (Fig. 2). The top 20 PCs accounted for a total of 67.3% of the total metabolomic variation, with the top 3 capturing 23.1%, 9.2%, and 6.2%, respectively. Individual PCs after the top 20 explained <0.82% of the metabolome. Linear models for each of the top 20 PCs found that the strongest drivers of variation in leaf metabolomics were phenology and temporal blocking factor. For example, 90.6% of variation on PC1 was due to phenology (*P* < 1e−05; Fig. 2A). PC2 primarily reflected the interaction of phenology and temporal block (26.4%, *P* < 1e−05) and temporal block as a main effect (18.9%, *P* < 1e−05). The patterns of variation attributable to PC2 were similar in PCs 3–10 (Fig. 2A).

PC17 was controlled by rootstock as a main effect (18.5%, *P* < 1e−03; Fig. 2B). On PC17, ungrafted vines were significantly different from vines grafted to 3309C (*P* = 0.02) and SO4 (*P* < 1e−05). Vines grafted to 1103P were also significantly different from vines grafted to SO4 (*P* = 0.009). Metabolites that loaded >1.96 SD from the mean loading on PC17 were extracted and independently fit to additional linear models. We identified 4 metabolite features (M374T1 [rt = 1.33, *m*/*z* = 374.1146], M117T1 [rt = 0.61, *m*/*z* = 117.0583], M175T1_1 [rt = 0.87, *m*/*z* = 175.1269], and M333T1_3 [rt = 0.71; *m/z* = 333.1582]) that were influenced by rootstock as a main effect and the metabolite (M112T1 [rt = 1.48, *m*/*z* = 112.0061]), which was influenced by the interaction of rootstock genotype and phenological stage. At this time, the identification of these features remains unknown.

Linear discriminant analysis confirmed that many experimental factors likely influence the metabolome. For example, when trained to maximize variation between classes of rootstocks, the model identified a space that weakly separates 1103P-grafted and SO4-grafted vines from ungrafted and 3309C-grafted vines (LD1) and separates 3309C-grafted vines from other classes (on LD2) (Fig. 2C). Despite this, machine learning showed minimal predictability for any class other than phenology, which was predictable with an accuracy of 100% for withheld samples. Rootstock genotype based on the metabolome was not predictable, with accuracy only marginally better than chance (34.6%).

### Gene expression

**Figure 3:**
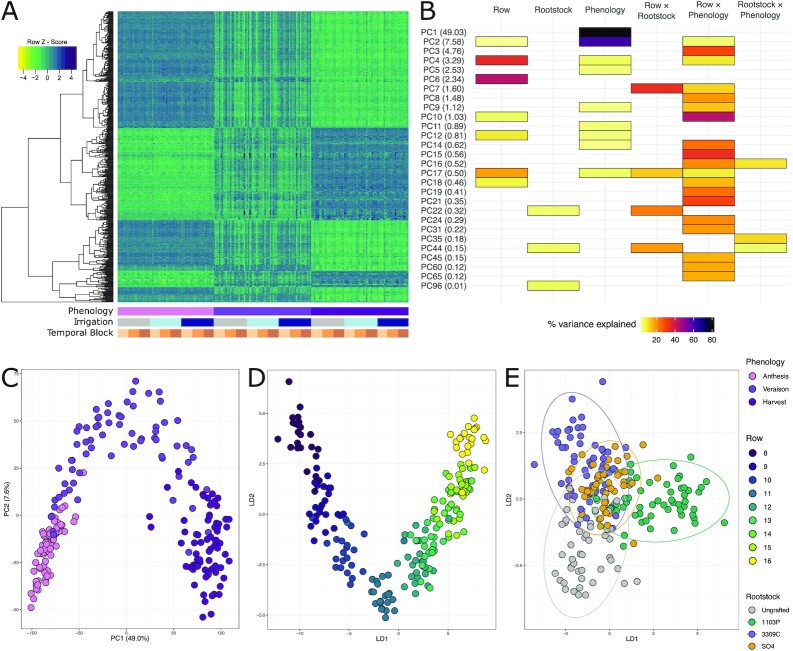
Gene expression primarily responds to time of season and circadian correlates. **(A)** Heat map showing 500 genes with the highest variance following the filtering of genes with low expression levels and gene-by-gene variance stabilizing transformations (VST) ordered by example model factors (below). **(B)** Percent variation captured in linear models fit to the top 100 principal components (PCs) of the VST-transformed gene-expression space. Presence of a cell indicates that the model term (top) was significant for that PC (left, percent variation explained by the PC in parentheses). **(C)** Projections of all samples into the first 2 PC dimensions to show that the largest descriptors of variation are due to phenology. **(D)** Projections of all samples into the first 2 dimensions of the linear discriminant space trained to maximize variation between the rows of the vineyard, and **(E)** rootstock genotype.

We performed 3′-RNAseq on the youngest fully opened leaves of the 72-vine set at 3 time points (Fig. 3). On average, each sample contained 4.1 million 3′-reads and measured the expression of 17,852 genes. Overall, we identified variation in 23,460 genes that had a DESeq2-normalized count ≥2 in ≥5 samples. We computed the expression of 2 classes of housekeeping genes and showed that they are generally stable across samples over phenological time (Supplementary Fig. S2). We noted that some variation is expected for housekeeping genes (see, e.g., [49]). Moreover, we showed that patterns of previously annotated circadian genes conform to expected results over the sampling window. For example, predicted orthologs of *LHY* and *RVE1* are correlated and decreasing over our sampling window, and a predicted *TOC1* ortholog is invariant. The results of these analyses provide general confidence in the gene expression data presented here.

Using a traditional differential expression analysis framework based on established DGE software (Deseq2), all genes returned as significantly differentially expressed by rootstock seemed to be false-positive results, evidenced by a single extreme outlier altering group means. Hierarchical clustering of the 500 most variable genes after variance stabilizing transformation (VST) showed strong latent structure in the transcriptome and that most variation in the transcriptome was explained by the phenological stage (Fig. 3A). The top 100 PCs on the VST-transformed gene counts accounted for 92.3% of variation in the transcriptome. Linear models on each of the top 100 PCs indicated that 82.4% and 61.4% of the variation on PC1 and PC2, respectively, were attributable to the phenological stage (Fig. 3B and C). Row was also a significant descriptor of variation as a single, fixed effect and in interactions with rootstock and phenological stage. For example, row accounted for 36.0% and 43.3% of the variation on PC4 and PC6, respectively. Interacting with the phenological stage, row accounted for >10% of variation on 17 additional PCs.

Patterns of gene expression identified through linear discriminant analysis (LDA) corresponded to phenological stage, vine row, and rootstock. LDA separated phenological stages into 3 distinct, non-overlapping groups in the space spanning LD1 and LD2 (Supplementary Fig. S4). When trying to separate rows into distinct classes, the model converged on a “horseshoe” shape in the LD1-LD2 space (Fig. 3D), suggesting either a circadian topology to the transcriptome or continuous spatial variation over the vineyard [50]. LD1 maximized the variation between row 8 (sampled early in the day) and row 16 (sampled a few hours later). LD2 maximized the separation of both rows 8 and 16 with row 12 (the row sampled in the middle of the sampling window). A model trained to separate rootstock classes (Fig. 3E) showed that LD1 separated the rootstock 1103P from other rootstock genotypes, and LD2 primarily separated the rootstock 3309C from ungrafted vines (Supplementary Fig. S4).

Formal machine learning on gene expression PCs largely supported the linear models. A random forest trained to predict phenological stage classified testing samples with 92.9% accuracy. Anthesis was the most predictable class, with a balanced accuracy of 100%; veraison and harvest displayed balanced accuracies of 92.7% and 92.4%, respectively. The PCs most important in phenology prediction were PC1 (MDA = 0.16) and PC2 (MDA = 0.12). Gene expression PCs were unable to predict rootstock, with a total prediction accuracy of 23.4%. While no features were especially important in the prediction processes, PC44 showed the largest mean decrease in Gini impurity, corroborating its signal in the linear models.

### Leaf shape

**Figure 4:**
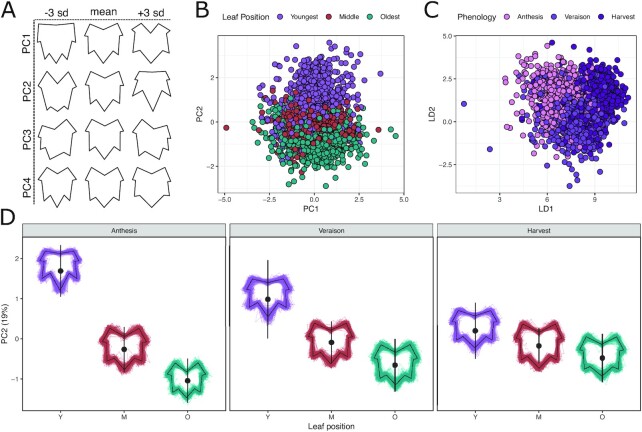
Leaf shape variation is primarily determined by shoot position but changes over the season. **(A)** Representative shapes showing leaf variation (−3 SD, mean, +3 SD) captured in each of the top 4 principal components of the generalized Procrustes analysis–rotated leaf shapes. **(B)** Projections of all leaves into the first 2 dimensions of principal component space colored by the strongest determinant of variation in the top 2 PCs. **(C)** Projections of all leaves into the first 2 dimensions of a linear discriminant space trained to maximize variation between phenological stages. **(D)** Variation in leaf shape captured on PC2 shown by leaf position and phenological stage. Large points (and error bars) represent the mean (and SD) of the group when projected onto PC2.Variation in each group is shown as a composite leaf trace scaled to a standard size and centered over the mean.

We collected leaves from the 288-vine set at 3 time points and landmarked a total of 2,422 leaves (Fig. 4). Homologous leaf landmarks were used for GPA. PCA on the GPA-rotated coordinates revealed that ∼97.2% of the total shape variation was captured by the top 20 PCs, with PC1, PC2, and PC3 explaining 24.1%, 19.0%, and 13.3% of the variation, respectively. Lower values on PC1 primarily capture leaves with shallow petiolar sinuses and short midvein distance from the depth of the superior sinus to the top of the midvein, whereas higher values on PC1 capture the opposite (Fig. 4A). Similarly, lower values on PC2 capture deep petiolar sinuses combined with very shallow superior sinuses, and vice versa for higher values. PC3 primarily captures asymmetry (Fig. 4A).

In total, 5.76% of variation on PC1 was explained by the experimental design. Of this, variation in leaf shape was explained by phenology (2.63%; padj < 1e−05), then rootstock (0.95%; padj < 0.001), leaf position (2.61%; padj = 0.03), and the interaction of phenology and leaf position (0.62%; padj = 0.009) (Supplementary Fig. S5A). Post hoc mean comparisons on PC1 showed that shapes of leaves from ungrafted vines were significantly different from leaves of vines grafted to 1103P (*P* < 0.001), 3309C (*P* < 0.001), and SO4 (*P* < 0.001) (Supplementary Fig. S5B). Moreover, PC1 captured subtle variation in the leaf position by phenological stage interaction, where middle leaves showed significant differences between anthesis and veraison (*P* < 1e−03), and the oldest leaves showed significant differences when comparing anthesis to veraison (*P* < 1e−05) and anthesis to harvest (*P* < 1e−03).

For PC2, 61.4% of variation could be assigned to an experimental factor. This included significant variation from leaf position (46.9%, padj < 1e−05), phenology (1.4%; padj < 1e−05), and the interaction of leaf position and phenology (12.05%; padj < 1e−05; Fig. 4D). Specifically, younger leaves tended to have shallower sinuses and exaggerated superior sinus depths (higher values on PC2), whereas older leaves tended to develop deeper petiolar sinuses and more shallow superior sinuses (lower values on PC2). The degree of this separation decreased across the season, and the shapes converged on the mean leaf shape on PC2, consistent with the middle leaf at all 3 phenological stages. PC2 additionally reflected the interaction of leaf position and rootstock (0.22%; *P* = 0.04; Supplementary Fig. S5B), but post hoc comparisons did not find any significant pairwise comparisons.

Machine learning on the GPA-rotated coordinate space identified moderate division of developmental and phenological classes. Random forest models could predict the leaf position with 73.1% accuracy, with the most important feature being the y-component of the leaf apex (MDA = 0.051). A model trained to predict phenology performed at 64.3%, with the most important features being the x-components of the points corresponding to superior sinus depth (left sinus MDA = 0.030, right sinus MDA = 0.019). A model trained to predict rootstock performed only marginally better than chance, at 28.1% accuracy.

### Vine physiology

**Figure 5:**
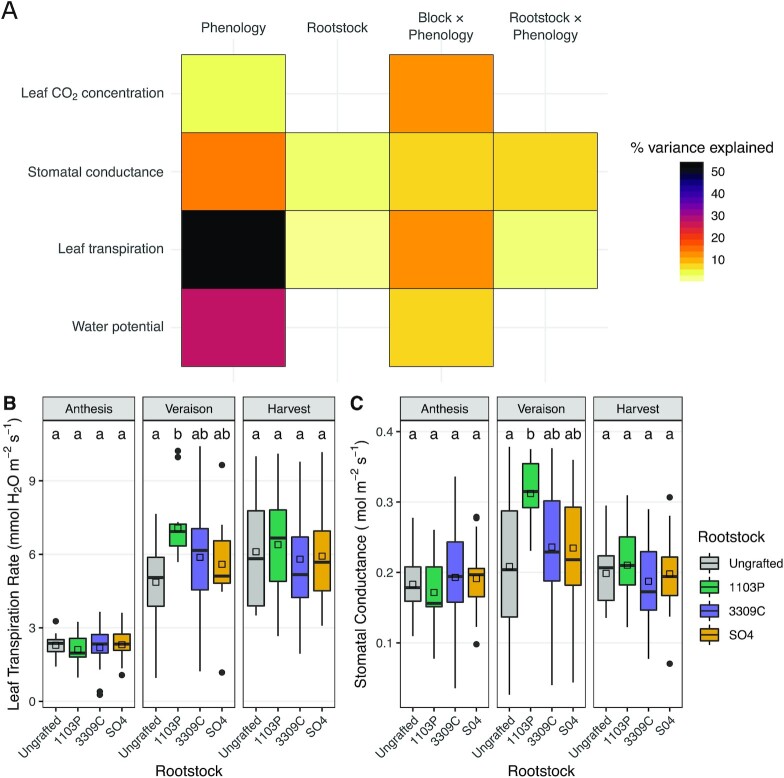
Vine physiology varies with rootstock and the rootstock by phenology interaction. **(A)** Percent variation explained by model terms (top) from linear models fit to each of 4 physiology traits (left). **(B)** Variation in leaf transpiration rate for each rootstock genotype over the course of the season. Boxes are bound by the 25th and 75th percentiles with whiskers extending 1.5 IQR from the box. Significant changes are indicated by letters above boxes and are only meant for comparison within each phenological stage. Group means are displayed with black squares. **(C)** Variation in stomatal conductance for each rootstock genotype over the course of the season. Boxes are bound by the 25th and 75th percentiles with whiskers extending 1.5 IQR from the box. Group means are displayed with black squares. Significant changes are indicated by letters above boxes and are only meant for comparison within each phenological stage. Dots indicate outliers.

We measured intracellular CO_2_ concentration (C_i_), stomatal conductance (g_s_), leaf transpiration, water potential ($\psi $), and soil moisture for the 72-vine set (Fig. 5). Each physiological phenotype varied significantly across phenology and the block by phenology interaction (Fig. 5A). For example, at harvest, we observed specific differences in leaf CO_2_ concentration (A vs C: *P* = 0.003; B vs C: *P* = 0.002) and leaf transpiration (A vs B: *P* < 1e−03; A vs C: *P* < 1e−05; B vs C: *P* < 1e−05). Leaf transpiration and stomatal conductance varied significantly with the interaction of rootstock and phenology. A post hoc comparison of means showed that leaf transpiration and stomatal conductances were elevated in Chambourcin vines grafted to 1103P at veraison as compared to leaves of ungrafted vines (leaf transpiration: *P* = 0.001; stomatal conductance: *P* = 0.002; Fig. 5B and C).

### Phenomic covariation

**Figure 6:**
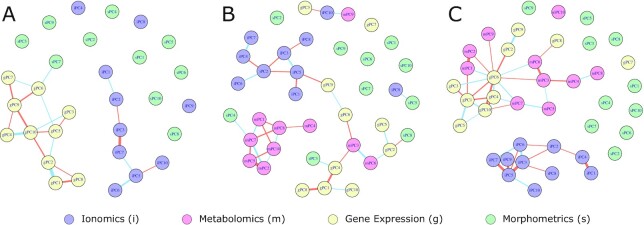
Phenomic covariation varies over the course of the season. Correlation networks showing patterns of covariation within and between phenotyping modalities. Nodes of the network are connected if they are significantly correlated (Pearson, FDR; padj < 0.05). Edge thickness is proportional to the strength of correlation (multiplied by 16 for visibility). Edge color reflects the direction of the correlation, where blue edges indicate positive correlations and orange edges indicate negative correlations. Modalities are indicated by a leading character and node color: ionomics (iPCs; purple), metabolomics (mPCs; pink), gene expression (gPCs; yellow), leaf shape (sPCs; green). Network topologies are shown for **(A)** anthesis, **(B)** veraison, and **(C)** harvest.

Four leaf phenotyping modalities consisted of ≥10 measured phenotypes and were measured for all plants in the 72-vine set (leaf ionome, leaf metabolomics, gene expression, leaf shape). Using these data, we explored the extent to which different phenotypes (within and between modalities) covaried over phenology and rootstock genotype (Fig. 6; Supplementary Figs S6 and S7). Within each phenotyping modality, we summarized the primary dimensions of phenotypic variation using PCA (see Methods) so as to not weigh any modality too heavily. From each PCA, we extracted the top 10 PCs, which explained a total of 88.9% of variation in the ionomics PCA (iPCA), 55.9% of the variation for the metabolomics PCA (mPCA), 74.8% of the variation in the gene expression PCA (gPCA), and 87.9% of the variation in the leaf shape PCA (sPCA).

Pairwise correlations of each PC within each phenological stage showed diverse correlation magnitudes and directions both within a phenotyping modality and between phenotyping modalities (Fig. 6; Supplementary Fig. S6). Generally, the strongest relationships were between PCs within phenotyping modalities. For example, the strongest correlations identified were between gene expression PCs gPC1 and gPC2 at anthesis (*r*= 0.85, CI = [0.81, 0.87]; Supplementary Fig. S6A) and metabolomics PCs mPC1 and mPC2 at harvest (*r* = −0.78, CI = [−0.82, −0.76]). Correlations between modalities represented a diversity of responses across phenological stages. For example, the correlation between gene expression gPC4 and shape sPC3 was similar across the phenological stages, but only the correlation at veraison was significant (*r* = 0.41, CI = [0.34, 0.47]; Supplementary Fig. S6B). Correlations such as between metabolomics mPC3 and gene expression gPC6 were similar and significant at both veraison (*r* = −0.44, CI = [−0.50, −0.37]; Supplementary Fig. S6C) and harvest (*r* = −0.37, CI = [−0.45, −0.28]; Supplementary Fig. S5C). While many correlations varied over the course of the season, some relationships entirely shifted in direction. For example, the correlation between metabolomics mPC3 and mPC6 shifted from a positive significant relationship (*r* = 0.58, CI = [0.52, 0.63]) at veraison to a negative significant relationship at veraison (*r* = −0.66, CI = [−0.73, −0.59]) (Supplementary Fig. S6D).

Pairwise comparisons of PCs within each rootstock genotype show a suite of latent phenotypes with significant presence/absence variation in significant correlations. Where each phenological stage showed modularity by phenotyping modality, variation over rootstock genotype shows a strong ionomics module with latent combination of other modalities interspersed (Supplementary Fig. S7). For example, in ungrafted vines, metabolomics mPC1 was correlated with 4 PCs from the ionome (Supplementary Fig. S7A). Each of the other rootstock genotypes had dramatically different topologies, with the ionome tending to be more connected within the ionome and connected to other modalities only on the periphery (Supplementary Fig. S7B–D). Examples of presence/absence variation were shown in small modules of 2 latent phenotypes that were present in only 1 rootstock genotype. For example, in the ungrafted vines, the correlation between gene expression gPC4 and metabolomics mPC3 was significant (*r* = −0.58, CI = [−0.65, −0.51]), and, in 1103P-grafted vines, the correlation between metabolomics mPC3 and shape sPC6 (*r* = 0.59, CI = [0.53, 0.70]) was significant.

## Discussion

In this study, we used grafted grapevines as an experimental system for characterizing root system effects on multi-dimensional leaf phenotypes over the course of a growing season. We detected ubiquitous but subtle effects of the root system on all assayed modalities and demonstrated that rootstock influences on leaf phenotypes can be specific to the vine's developmental stage. The strongest signals of rootstock influences on leaves were observed in the ionomics dataset, phenotypes for which the root system has a noted and well-understood role.

### Phenology explains significant variation in all leaf phenotypes

The timing of sampling or phenological stage of the vines (anthesis, veraison, harvest) was the strongest driver of phenomic variation for most leaf phenotypes. For example, all 20 ions varied with phenology and most ions showed that phenology, or the interaction of phenology with leaf developmental position, was the strongest source of variation (Fig. 1). Nearly one-third of all measured transcripts responded to seasonal variation, and the strongest effects on the transcriptome were phenology and row, a correlate for the time within a 3-hour sampling window. The only phenotype for which phenology was not the most explanatory factor is leaf shape. Consistent with previous studies [23], we confirm that most of the leaf shape variation reflects development along a single shoot, but much of this variation is explained via interaction with phenology. These data highlight the dynamic nature of biological processes taking place within grapevines over the course of a season.

The seasonal component to grapevine phenomic variation is a subject of much research, especially in the berry. In studies designed to quantify molecular underpinnings of terroir, seasonal variation was identified as the strongest signal in the metabolome [51–54]. Several studies have characterized transcriptomic variation over the course of the season. For example, in conjunction with metabolomics, seasonal variation of berry development was used to identify transcriptomic and metabolomic developmental markers in “Corvina” [55]. Follow-up analysis showed that 18% of transcripts varied seasonally [56]. Grapevine leaf shape also varies tremendously over the growing season [23] and is stable over multiple growing seasons; interestingly, grapevine leaves are patterned in the previous year, and the climate of the season in which the leaves were patterned influences aspects of leaf shape [57,58].

### Grafting and rootstock genotype exhibit a complex and subtle signal on leaf phenotypes

Consistent with previous studies, we confirm that grafting, as well as rootstock genotype, has a complex effect on phenomic variation in the scion (the grafted shoot system). Most notably, we show that the rootstock to which a scion is grafted influences ion concentrations in leaves. Rootstock genotype is predictable from ion concentrations in the leaves, and this signal is strengthened when phenological stage is included in the model. For example, we previously showed that nickel concentration was elevated in vines grafted to the rootstock SO4 [19]. At a similar point in the season, we observe the same pattern, but by harvest, nickel was almost entirely excluded from the leaf. This suggests that the biological implications of this differential uptake could be missed if not surveyed across the season. We also confirm that rootstock genotype influences the metabolome of grafted grapevine, in some cases in a season-specific manner. In the transcriptome, PCA was able to identify dimensions of variation that were significantly described by rootstock and the interaction of rootstock and time of day, confirming prior observations [19]. Patterns of gene expression were associated with rootstock in some analyses; e.g., supervised methodologies identified linear discriminants in the PC space that separated gene expression patterns of some rootstock genotypes. However, gene-by-gene analysis found no genes modulated by rootstock genotype, or even just from the act of grafting, that were not driven entirely by a single outlier. We suspect that these results are due, at least in part, to the strength of the phenology effect overpowering more subtle variation imparted by rootstock genotype. Finally, of the physiology phenotypes that we measured, leaf transpiration and stomatal conductance were higher in vines grafted to 1103P in the middle of the season. Through these analyses, we have identified subtle but ubiquitous effects of rootstock genotype on shoot system phenotype across modalities and have shown that the effect of grafting on leaf phenomic variation varies from one phenotype to the next.

Understanding the rootstock genotype influence on shoot system phenotypes is a growing area of research, especially in grapevine. For example, in Cabernet Sauvignon, grafting increased ion uptake globally and some rootstock genotypes provide a clear signal in the scion [28]. The wild *Vitis* species from which the rootstocks were derived (*V. berlandieri, V. riparia*, and *V. rupestris*) differ in root architecture, preferred soil substrate, and genetic background; however, the specific aspects of their biology that contribute to differences in ion uptake are not known [27]. To our knowledge, there is not yet a strong causal link between the micronutrient component of the ionome and factors of vine growth or development that might influence traits such as wine quality. However, it is noted that macronutrient deficiencies can have negative effects on such traits [59,60] and can be mediated by rootstock [61]. This suggests that a strong understanding of the rootstock influence on the vine's ionome is warranted, and more work needs to be done to establish these relationships. Similarly, the metabolome is a key driver of the formation of the graft junction and some key metabolites could be responsible for graft incompatibility [62]. Building on this work, targeted metabolomics showed that 2 classes of metabolites, flavanols and stilbenes, were differentially abundant at graft junctions and in the rootstocks of Cabernet Sauvignon vines 1 month after grafting [63]. However, flavanols were not differentially abundant in the scion, but scion stilbene concentrations were apparently controlled by rootstock genotype. The effect of rootstock genotype on the scion transcriptome is perhaps the most varied. For example, Cabernet Sauvignon shoot apical meristems show no effects by rootstock genotype [14], but berries of the same cultivar do, although the effect is tempered by seasonal variation [15]. Variation in Chambourcin leaf shape was also driven by rootstock genotype, especially in conjunction with differences in irrigation [19]. Collectively, these studies all suggest that rootstock genotype influences scion phenotypes, but those effects will vary by phenotype, scion genotype, and perhaps other experimental conditions.

Data presented here confirm and expand upon previous observations of rootstock effects on scion phenotypes. Notably, this study was carried out using a robust experimental design (288-vine set and 72-vine set comprising replicates of 3 rootstocks grafted with a common scion and an ungrafted control) in a vineyard that had been in the ground for 8 years at the time of sampling. Our coordinated collection of 5 multi-dimensional leaf phenotypes and inclusion of 3 sampling points spanning the growing season allowed us to investigate the comprehensive nature of rootstock influences on the scion. Furthermore, this thorough analysis demonstrates that rootstock effects on scion phenotypes shift in magnitude over the course of the season, indicating that aspects of time are tremendously influential to the observed results regardless of phenotype.

While the results of previous studies on grafted grapevine are worthy of comparison, the work presented here has a few limitations that render comparisons with other studies challenging for a variety of reasons. One novelty in our study is the exploration of a hybrid grapevine system, Chambourcin. Chambourcin has a complex pedigree, including contributions from *V. riparia* and *V. rupestris*, species that are each parent to 2 of the rootstocks used in this study [64]. Many of the significant effects that we observed in this study were subtle, which could reflect the genomic similarity between shoot and root systems. It might be expected that rootstocks derived from *V. riparia, V. rupestris*, and other North American species might prompt more pronounced responses in European scions that lack North American *Vitis* in their pedigrees. Moreover, our results were derived from data collected in a single year at a single location. The phenotypes that we measured are known to be heavily influenced by the environment, and we expect some inter-annual variation in rootstock influences on shoot system phenotypes. This study focused on a single scion, and as a result we are unable to explore how rootstock effects on shoot system phenotypes vary across scions. To our knowledge, this is among the largest populations to have been surveyed for such phenotypes in a near-decade-old established vineyard. While many studies have been conducted in greenhouses or recently planted vineyards, the juxtaposition of our results and those previously established serves as a powerful foundation for the generation of hypotheses for future studies.

### Phenomic covariation warrants work toward latent phenotypes

In the present study, we assess the extent of covariation among leaf phenotypes. For the primary dimensions of variation in each modality, within-modality correlations were strongest when accounting for phenological timing. Correlations also existed between modalities, suggesting room for the analysis of latent phenomic structure or targeted integrative analyses for experimental questions. For example, aspects of the metabolome were frequently correlated with the transcriptome and leaf shape when accounting for both phenological stage and rootstock genotype. Interestingly, correlations within and between modalities were highly dynamic over a growing season and across rootstock genotype. For example, several correlations with leaf shape were present at veraison but were not detected at anthesis and harvest. Moreover, the topology of connections in the ionomic network was variable over the rootstock genotype (Supplementary Fig. S6). This variation in topology confirms that root system genotype has a strong influence on shoot system elemental composition and suggests that root system genotype can alter correlative patterns in the ionome. We believe that phenomic covariation warrants further investigation, specifically, by further including additional phenotypes such as long non-coding RNA expression [65,66], epigenetics [67], and microbiomes [68,69], which could yield more mechanistic understandings of the influence of root systems on shoot systems and how plants interact with their environments through their root systems. These mechanistic understandings could be used to further understand and optimize consumer-facing traits such as fruit quality and yield. To date, much of the work constituting phenomics in grapevine has addressed how berries develop over the growing season, how cultivars differ from one another, and how the concept of terroir influences wine [51,52,55,70–72]. Despite data integration techniques becoming more popular, there are still many open questions as to what analytical methods are most appropriate and how to most effectively utilize them (reviewed for grapevine in [73,74]; reviewed broadly in [75,76]). Ongoing work attempts to integrate high-dimensional phenomic datasets generated within a single organ system (e.g., leaves); and future studies will expand this to explore phenomic covariation in and among organs, over time, and across space.

## Potential Implications

Our work on the influence of root system genotype on shoot system phenotype has broad implications for a holistic understanding of how plants detect and respond to changing environmental conditions and how this response is coordinated among different organ systems. Data presented here demonstrate that root systems that are genetically distinct from the scion exert influence on the scion, leading to statistically significant differences in scion phenotypes based on the identity of their root systems. This observation suggests that the above-ground phenotype results, at least in part, from below-ground activity of the root system. Further, these data highlight the value of coordinated collection of different multi-dimensional phenotypes for comparative studies, and for describing whole-plant phenotypic shifts over seasons and in response to horticultural manipulations.

Beyond its use as an experimental model that is ideal for studying root/shoot interaction, grafting is an important horticultural technique that is used in >70 major crops. In grapevines, grafting was developed primarily to combat the below-ground pest phylloxera, and grapevine rootstocks were selected initially on the basis of their resistance to this pest. Results presented here indicate that beyond phylloxera resistance, grafting to genetically distinct rootstocks is a potential source of variation for the scion. Ongoing work explores how root system effects on shoot system phenotypes vary across scion genotypes and how the rootstock × scion interaction changes over space. The long-term implications of this study are the potential honing of viticulture for future climates including the optimization of rootstock-scion combinations based in part on an understanding of how rootstock effects on scion phenotypes change over the course of the season. This work is relevant for breeding efforts and may play a role in the optimization of quantitative phenotypes such as vigor, fruit quality, and yield that may be enhanced by, constrained by, or partially predicted from phenotypic variation elsewhere in the plant.

## Methods

### Study design

Data were collected in 2017 from a split-plot experimental rootstock trial established in 2009 at the University of Missouri's Southwest Research Center near Mount Vernon, MO (37 4.26 N; 93 52.44 W; Supplementary Fig. S1). The rootstock trial includes the interspecific hybrid cultivar “Chambourcin” growing ungrafted (own-rooted) and grafted to 3 rootstocks: 1103P, 3309C, and SO4 (Supplementary Fig. S1D). Clonal replicates of each of the 4 rootstock-scion combinations were planted 72 times for a total of 288 vines planted in 9 rows. Each row was treated with 1 of 3 irrigation treatments: full evapotranspiration replacement, partial (50%) evapotranspiration replacement (reduced deficit irrigation), or no evapotranspiration replacement (Supplementary Fig. S1A). However, rainfall in 2017 likely mitigated the applied irrigation treatment (see Supplementary Note S1). Vine position in the vineyard corresponded to time of sampling for some phenotypes (metabolomics, gene expression, and physiology), as samples were taken from one end of the vineyard to the other over the course of 2–3 hours. Because vineyard microclimates and sampling time may be associated with phenomic variation, we defined “block” as a factor that captures this spatial and temporal variation inherent in sampling for those phenotypes. In the other phenotypes (ionomics and leaf shape), neither row nor block correlated with time, so block was simply a spatial covariate. Unique rootstock-scion combinations were planted in cells of 4 adjacent replicated vines (Supplementary Fig. S1A and B), with rows consisting of 8 cells (32 vines/row). To our knowledge, a field-planted rootstock experimental vineyard of this size and age is rare. For some phenotypes (ionomics and leaf shape), it was possible to collect samples from all vines in the experimental vineyard (the 288-vine set; Supplementary Fig. S1A and B). For other phenotypes (metabolomics, gene expression, and physiology), time and/or expense associated with the phenotyping process required that we reduce sampling to a nested set of 72 vines representing the middle 2 vines in each 4-vine cell in the front half of the vineyard (the 72-vine set; Supplementary Fig. S1B and C). All phenotypes were assayed at 3 phenological stages: anthesis (∼80% of open flowers; 22 May 2017), veraison (∼50% of berries had transitioned from green to red; 30 July 2017), and immediately prior to harvest (25 September 2017). At each phenological stage, effort was made to sample on days with full to partial sun and minimal precipitation.

This design was used to assess the following questions: (i) What is the influence of root system genotype on shoot system phenotype? (ii) How do systems of plant phenotypes vary over the growing season, and does rootstock genotype influence this variation? (iii) How do phenotypes covary within and between phenotyping modalities?

### Linear models

Linear models were fit to the 20 measured ion concentrations, the top 20 PCs of the leaf metabolome, the top 100 PCs of the leaf transcriptome, the top 20 PCs of leaf morphospace, and each measured physiological trait. Outliers were detected using the R function “anomalize” (options: alpha = 0.03, max_anoms = 0.1). Each model was fit with fixed-effect factors representing phenological stage (anthesis, veraison, or harvest), rootstock (ungrafted, 1103P, 3309C, or SO4), leaf position (youngest, middle, or oldest; only used in leaf morphology and leaf ion concentration models), and all pairwise interactions of those terms. Both irrigation and block were included as fixed, non-interacting effects with the exceptions of physiology and metabolomics, for which we allowed the interaction of block as it correlates with the time of sampling, potentially capturing temporal variation. Row, an additional correlate for time and spatial variation, was included in place of a temporal block for the gene expression models after removal of the variation attributable to irrigation, a factor collinear with row. All linear models were interpreted using a Type 3 sum-of-squares computation using the R package “car” [77]. Estimated *P*-values for each term in the models were corrected for multiple tests (within phenotype) using false discovery rate (FDR) correction as implemented by the R package “stats” [78]. Results from the models are reported as the variation explained by a particular term in the model and the estimated *P*-value. When appropriate, post hoc mean comparisons were computed using the package “emmeans” [79]. Where multiple linear models were being simultaneously interpreted, we applied a Bonferonni correction to reduce the number of false-positive results.

### Machine learning to identify rootstock effects

For visualization of between-class variation, we fit LDA models to each modality (ionomics, metabolomics, gene expression, and leaf morphology) using the “lda” function of the R package “MASS” [80]. Projections of all samples into the LD space were plotted using ggplot2 [81]. In addition, we used machine learning to capture subtle experimental effects. We partitioned data from each modality into 80% training and 20% testing samples. Models were fit to predict the phenological stage from which a sample was taken, the rootstock to which the scion was grafted, and the joint prediction of phenology and rootstock. We also tested the predictability of leaf position for ionomics and leaf shape, and the interaction of rootstock and leaf position for ionomics. We used the “randomForest” [82] implementation of the random forest algorithm. Models were fit and tuned using the R package “caret” [83]. Each performance was assessed using accuracy, with performance on each class being assessed using the balanced accuracy, the midpoint of class-wise sensitivity and specificity. Where appropriate, models were compared to “chance,” or the occurrence frequency of each class. Confusion matrices were visualized from the out-of-bag predictions using ggplot2. Important features were identified from the randomForest object on the basis of a phenotype-specific MDA.

### Phenomic trait covariation

We extracted ionomics, metabolomics, gene expression, and leaf shape data for the youngest available leaf from the 72-vine set. Each data modality was summarized along the primary dimensions of variation using PCA. For each class, we extracted the top 10 PCs and fit Pearson correlations across all pairs of PCs at each phenological stage. *P*-values from computed correlations were corrected using the FDR method from the package “stats” [78]. Correlations and their strengths were visualized using the R package “igraph” [84]. Example correlations were reported after running 10,000 bootstrapped subsamples of 90% of data for paired phenotypes. From the distribution of estimated correlation coefficients, confidence intervals were computed from the 0.025 and 0.975 quantiles. A subset of example correlations were plotted using the R package ggplot2.

## Availability of Source Code and Requirements

All code to replicate the findings of this article including shell scripts for RNAseq analysis and Jupyter Notebooks for data analysis in R can be found on the Vitis Underground GitHub:

Project name: mt_vernon_2017_leaf

Project home page: https://github.com/PGRP1546869/mt_vernon_2017_leaf

Operating system: Platform independent

Programming language: R and Shell

Other requirements: R requirements are listed in the Jupyter Notebooks. Shell requirements: trimmomatic v0.36, bbmap (11 February 2019), STAR v2.7.1a, htseq-count v0.11.2

License: GNU GPL 3.0

Any restrictions to use by non-academics: None

## Data Availability

Raw metabolomics data are available at MetaboLights, accession MTBLS2831. Gene expression data are available in the Sequence Read Archive under BioProject PRJNA674915. All other data supporting this article including ionomics, partially processed metabolomics, leaf scans, leaf landmarks, physiology, and weather data are available from figshare [85–89]. Other data further supporting this work are openly available in the *GigaScience* repository, GigaDB [90].

## Additional Files

**Supplementary Figure S1**. Experimental design. **(A)** Vineyard map. The vineyard features a randomized block design where “Chambourcin” is grown ungrafted and grafted to 3 rootstock genotypes: 1103P, 3309C, and SO4. Each row is treated with 1 of 3 irrigation treatments: full replacement of evapotranspiration, reduced-deficit, or no replacement of evapotranspiration. Each cell of the vineyard contains 4 replicate grafts. **(B)** Phenotype sampling scheme across the 4 replicates in a cell. For example, the top panel (purple) shows all 4 vines in the first cell of Row 8 in Block D. From each vine in that cell, ionomics and leaf shape were sampled. In contrast, the bottom panel shows the first cell in Row 8 in Block A. Here, the first and fourth replicates were sampled for ionomics and leaf shape while the second and third replicates were sampled for all phenotypes. All vines (288) were sampled for ionomics and leaf shape. The middle 2 vines in the front half of the vineyard (72) were additionally sampled for metabolomics, gene expression, and physiology. **(C)** Phenotype sample scheme within a vine (along a shoot). For each plant, young leaves were sampled for ionomics, leaf shape, and gene expression. Middle leaves were sampled for ionomics, leaf shape, metabolomics, and physiology. Older leaves were sampled for ionomics and leaf shape. Samples for ionomics and leaf shape were taken from the same shoot. All other phenotypes were sampled from independent shoots. **(D)** Rootstock relatedness. Each of the rootstocks in this trial shares a parent species with a different rootstock. 1103P is a cross between *V. rupestris* and *V. berlandieri*. 3309C is a cross between *V. rupestris* and *V. riparia*. SO4 is a cross between *V. riparia* and *V. berlandieri*. The parent that is shared between each pair of rootstocks is highlighted. This figure is partially reproduced from [19] available under a Creative Commons license (CC BY 4.0).

**Supplementary Figure S2**. Quality and validity assessment of 3′ RNAseq data. (A) A survey of recently annotated circadian clock orthologs from the grapevine genome annotation [44]. Orthologs surveyed included the morning-phased RVE1 and LHY, evening-phased LUX and ELF4, and the night-phased TOC1. (B) A survey of genes with housekeeping domains related to IPR000626 (ubiquitin) and IPR004000 (actin).

**Supplementary Figure S3**. Patterns of ion covariation change over experimental treatments. Correlation networks showing patterns of ion covariation across phenological stages and shoot position. Nodes of the network are connected if they are significantly correlated (Pearson, FDR; padj < 0.05). Edge thickness is proportional to the strength of correlation (multiplied by 16 for visibility). Edge color reflects the direction of the correlation, where blue edges indicate positive correlations and orange edges indicate negative correlations.

**Supplementary Figure S4**. Patterns of variation contributing to gene expression linear discriminants (LDs). (A) Projections of leaf gene expression samples into the first 2 dimensions of an LD space trained to maximize variation between phenological stages, rows in the vineyard, and rootstock genotype. For each LD, the PCs that loaded significantly (>1.96 SD from the mean loading) are listed in order of loading magnitude. (B) Distribution of the top loading PCs onto LD1 and LD2 for each of the trained models.

**Supplementary Figure S5**. Patterns of variation in leaf shape are subtle. (A) Percent variation captured in linear models fit to each of the top 20 principal components (PCs) of leaf morphology. Presence of a cell indicates that the model term (top) was significant for that PC (left, percent variation explained by the PC in parentheses). (B) Composite leaf traces for the main rootstock genotype effect identified on PC1.

**Supplementary Figure S6**. Example correlations within and between phenotyping modalities over the course of the season. (A) Example correlation showing a strong within-modality correlation between the ionomics gPC1 and gPC2 at anthesis. Pearson correlations by phenological stage and CIs derived from 10,000 random 90% draws are shown for each panel. Generally speaking, CIs overlapping with 0 were not accepted as significant. (B) Example correlation showing one of the stronger between-modality correlations between the gene expression gPC4 and morphology (shape) sPC3 at veraison. (C) Example correlation of a relationship that is present multiple times over the course of the season between metabolomics mPC3 and gene expression gPC6 at both veraison and harvest. (**D**) Example correlation that is dynamic over the course of the growing season between the ionomics mPC3 and mPC6.

**Supplementary Figure S7**. Phenomic covariation varies over rootstock genotype. Correlation networks showing patterns of covariation within and between phenotyping modalities. Nodes of the network are connected if they are significantly correlated (Pearson, FDR; padj < 0.05). Edge thickness is proportional to the strength of correlation (multiplied by 16 for visibility). Edge color reflects the direction of the correlation, where blue edges indicate positive correlations and orange edges indicate negative correlations. Modalities are indicated by a leading character and node color: ionomics (iPCs; purple), metabolomics (mPCs; pink), gene expression (gPCs; yellow), leaf shape (sPCs; green). Network topologies are shown for (A) ungrafted, (B) 1103P-grafted vines, (C) 3309C-grafted vines, and (D) SO4-grafted vines.

Supplementary Note 1. Supplemental analaysis of the irrigation treatement. This file contains the results of supplemental analysis of the irrigation treatment as it related to seasonal rainfall in the vineyard. In the Note, we show that rainfall more than made up the difference of the reduced-deficit and droughted irrigation treatments leading to no measureable effect from irrigation.

**Supplementary Note S1**.

## Abbreviations

bp: base pairs; CI: confidence interval; DGE: differential gene expression; DPI: dots per inch; FDR: false discovery rate; GPA: generalized Procrustes analysis; gPCA: gene expression PCA; LC-MS: liquid chromatography–mass spectroscopy; MS: mass spectroscopy; iPCA: ionomics PCA; IQR: interquartile range; LDA: linear discriminant analysis; LD: linear discriminant; MDA: mean decrease in accuracy; mPCA: metabolomics PCA; *m*/*z*: mass to charge ratio; pajd: adjusted *P*-value; PAM: pulse amplitude modulated; PCA: principal component analysis; PC: principal component; rt: retention time; sPCA: shape (morphology) PCA.

## Competing Interests

The authors declare that they have no competing interests.

## Funding

This work was funded by the National Science Foundation Plant Genome Research Project 1546869, PI: A.J.M.

## Authors' Contributions

A.J.M., D.H.C., A.F., L.G.K., M.K., J.P.L., and Q.M. designed the experiment. Z.N.H., L.L.K., M.A., J.F.S., Z.M., N.B., E.F., and J.P.L. contributed to sample collection and sample processing. Z.N.H., L.L.K., J.F.S., and M.A. contributed to data analysis. Z.N.H. and A.J.M. contributed to the writing of the manuscript. All authors contributed to manuscript editing.

## Supplementary Material

giab087_GIGA-D-21-00137_Original_Submission

giab087_GIGA-D-21-00137_Revision_1

giab087_GIGA-D-21-00137_Revision_2

giab087_GIGA-D-21-00137_Revision_3

giab087_Response_to_Reviewer_Comments_Original_Submission

giab087_Response_to_Reviewer_Comments_Revision_1

giab087_Response_to_Reviewer_Comments_Revision_2

giab087_Reviewer_1_Report_Original_Submission Pablo Carbonell-Bejerano -- 5/30/2021 Reviewed

giab087_Reviewer_1_Report_Revision_1 Pablo Carbonell-Bejerano -- 10/17/2021 Reviewed

giab087_Reviewer_2_Report_Original_Submission Sam Henderson -- 6/10/2021 Reviewed

giab087_Reviewer_2_Report_Revision_1 Sam Henderson -- 10/4/2021 Reviewed

giab087_Reviewer_3_Report_Original_Submission Giuseppe Montanaro -- 6/11/2021 Reviewed

giab087_Reviewer_3_Report_Revision_1 Giuseppe Montanaro -- 9/26/2021 Reviewed

giab087_Reviewer_4_Report_Original_Submission Larry York -- 6/22/2021 Reviewed

giab087_Reviewer_4_Report_Revision_1 Larry York -- 10/21/2021 Reviewed

giab087_Supplemental_Files
