# Supplementary material for: Multi-dimensional leaf phenotypes reflect root system genotype in grafted grapevine over the growing season
Source: Gigascience. 2021 Dec 29;10(12):giab087. doi: 10.1093/gigascience/giab087 (PMC8716362; doi:10.1093/gigascience/giab087)
Supplement: giab087_GIGA-D-21-00137_Revision_1 [file giab087_giga-d-21-00137_revision_1.pdf]

GigaScience

# Multi-dimensional leaf phenotypes reflect root system genotype in grafted grapevine over the growing season

--Manuscript Draft--

| | |
|---|---|
| Manuscript Number: | GIGA-D-21-00137R1 |
| Full Title: | Multi-dimensional leaf phenotypes reflect root system genotype in grafted grapevine over the growing season |
| Article Type: | Research |
| Funding Information: | National Science Foundation (1546869) — Dr Allison J. Miller |
| Abstract: | Background: Modern biological approaches generate volumes of multi-dimensional data, offering unprecedented opportunities to address biological questions previously beyond reach due to small or subtle effects. A fundamental question in plant biology is the extent to which below-ground activity in the root system influences above-ground phenotypes expressed in the shoot system. Grafting, an ancient horticultural practice that fuses the root system of one individual (the rootstock) with the shoot system of a second, genetically distinct individual (the scion), is a powerful experimental system to understand below-ground effects on above-ground phenotypes. Previous studies on grafted grapevines have detected rootstock influence on scion phenotypes including physiology and berry chemistry. However, the extent of the rootstock's influence on leaves, the photosynthetic engines of the vine, and how those effects change over the course of a growing season, are still largely unknown.<br>Results: Here, we investigate associations between rootstock genotype and shoot system phenotypes using five multi-dimensional leaf phenotyping modalities measured in a common grafted scion: ionomics, metabolomics, transcriptomics, morphometrics, and physiology. Rootstock influence is ubiquitous but subtle across modalities with the strongest signature of rootstock observed in the leaf ionome. Moreover, we find that the extent of rootstock influence on scion phenotypes and patterns of phenomic covariation are highly dynamic across the season.<br>Conclusions: These findings substantially expand previously identified patterns to demonstrate that rootstock influence on scion phenotypes is complex and dynamic and underscore that broad understanding necessitates volumes of multi-dimensional data previously unmet. |
| Corresponding Author: | Zachary N Harris<br>Saint Louis University<br>SAINT LOUIS, MO UNITED STATES |
| Corresponding Author Secondary Information: | |
| Corresponding Author's Institution: | Saint Louis University |
| Corresponding Author's Secondary Institution: | |
| First Author: | Zachary N Harris |
| First Author Secondary Information: | |
| Order of Authors: | Zachary N Harris |
| | Mani Awale |
| | Niyati Bhakta |
| | Daniel H. Chitwood |
| | Anne Fennell |
| | Emma Frawley |
| | Laura L. Klein |
| | |

Powered by Editorial Manager® and ProduXion Manager® from Aries Systems Corporation

| | |
|---|---|
| | Laszlo G. Kovacs |
| | Misha Kwasniewski |
| | Jason P. Londo |
| | Qin Ma |
| | Zoë Migicovsky |
| | Joel F. Swift |
| | Allison J. Miller |
| **Order of Authors Secondary Information:** | |
| **Response to Reviewers:** | Editor Comments:<br>Overall, the reviewers have highlighted that in its current form, the manuscript requires more biological validation and more detailed methods to ensure reproducibility of the work presented. Limitations on the study should be discussed and how they may impact the results; and more data should be provided to understand the reliability of the RNA-seq experiment.  Furthermore, RNA-seq methods are incomplete - we strongly encourage all authors to add their detailed methods to protocols.io (if not already open in protocols.io and cite the protocol DOI in the paper.<br>-Response: Thank you for this. We have added all details that have been requested on L191-195. In addition, we added a supplemental figure (Supplemental Figure 2) showcasing our analysis for gene expression validity. A summary of this analysis is on L207-210.<br><br>We also see reviewer #4 suggests to use Figshare - but this is not an appropriate database to share large-scale data, such as this work presents. Github is still the most appropriate place to share scripts and associated documentation, and our open repository, GigaDB can host the other metadata not already open in other community approved repositories; we will also host snapshots of your scripts in GitHub.<br>-Response: In addressing Reviewer #4's specific recommendation, we have moved the document in question from GitHub to a Supplementary Note in the manuscript. In line with previous communications, we are also in the curation process of metabolomics data uploaded to Metabolights. We are happy to jointly upload other data sets to preferred repositories and databases, we just need guidance on the preferred locations for those data. As of today, we are unaware of standard databases for ionomics and leaf shape data. Scripts used for analysis are still available on GitHub, but can be hosted elsewhere if this is of concern.<br><br>Reviewer #1<br><br>The manuscript by Harris and co-workers presents a characterization of rootstock genotype effects on multilevel leaf phenotypes of one grafted grapevine scion cultivar. Three rootstock genotypes along with the ungrafted cultivar were compared for ionomics, transcriptomics, metabolomics, leaf morphology and physiology in three phenological stages. Analytical and statistical analyses applied were generally sound. While authors identify larger effects in most cases for stage and vineyard position/sampling time, ionic composition was the phenotype most significantly affected by rootstock genotype. Co-variance among multilevel phenotypes is also presented.<br><br>Major issues<br>1.      The study comprises a vast dataset, with a total of 288 plants independently analyzed for two phenotypes (morphometry and ionomics) and 72 plants were used for the rest of phenotypes. Nevertheless, the experiment is limited in terms of genotypes tested and reproducibility. Only one year of study and under the specific soil and climate conditions of a single field plot. Moreover, the effects were only tested on a single scion genotype, a bred interspecific hybrid including Vitis riparia and V. rupestris in its pedigree. At least one of these species is also in the pedigree of the three rootstocks tested, which might involve lower diversity than in common interactions between rootstocks and V. vinifera cultivars. These limitations should at least be considered when discussing the results.<br>-Response: The comments provided by the reviewer are all excellent observations that were clearly missing from the discussion of our work. We have added a paragraph to |

Powered by Editorial Manager® and ProduXion Manager® from Aries Systems Corporation

| | |
|---|---|
| | the Discussion (L544 - L559) to better couch how our work should be compared to other studies and the considerations that may account for those differences.<br><br>2. The RNA-seq assay did not identify differentially expressed genes (DEGs) in response to rootstock genotype, which disagrees with previous reports. While the origin of the lack of effects here is unclear, further data should be provided to understand the reliability of the RNA-seq experiment:<br>-Response: We thank the reviewer for this comment. We were similarly interested to see that our results differ from previously published analyses in similar systems. We added comments to the discussion to clarify why we think these differences might be present. To ensure such reliability, we confirmed in our data that previously established patterns of house-keeping and circadian phased genes were behaving as expected. The following has been added to the manuscript:<br>-Added to data description L208-210, “To check the validity of our expression results, we assayed two classes of housekeeping gene (Ubiquitin-domain and actin-family) and eight previously annotated circadian genes (Carbonell-Bejerano et al. 2014)”<br>-Added to Analyses, L339 - 345, “We computed the expression of two classes of housekeeping genes, and showed that they are generally stable across samples over phenological time (Supplemental Figure 2). We noted that some variation is expected for housekeeping genes; see, for example, [49]. Moreover, we showed that patterns of previously annotated circadian genes conform to expected results over the sampling window. For example, predicted orthologs of LHY and RVE1 are correlated and decreasing over our sampling window, and a predicted TOC1 ortholog is invariant. The results of these analyses provide general confidence in the gene expression data presented here.“<br>-Created and added Supplemental Figure (now supplemental Figure 2) showing these patterns.<br><br>2.1. Which was the timing of sample collection for RNA-seq samples? Was the same sampling order followed for the three phenological stages? Which were the weather conditions on each of the three sampling dates? It is relevant to describe that information since environmental and circadian changes between and within days can alter gene expression.<br>-Response: We added the following to the section describing the gene expression data set (L187-190): “Leaves were sampled by a single team near midday between 10AM and 2PM in row order ensuring that ‘block’ and ‘row’ accounted for unmeasured environmental variation and temporal variation over the sampling window.” A statement was added on L637-638 that “At each phenological stage, effort was made to sample on days with full to partial sun and minimal precipitation.“<br><br>2.2. Data on RNA sequencing depth should be provided to understand the resolution of the transcriptomics experiment. For instance, how many bases/reads per sample were produced? How many genes per sample were called as expressed (DESeq2-norm counts >2 according to authors own threshold)?<br>-Response: Information on sequencing depth and genes per sample were added to the Analyses section. Specifically, we added the following to L337-338: “On average, each sample contained 4.1 million 3’-reads and showed the expression of 17,852 genes.”<br><br>3. The interpretation of the origin of the results is generally shallow and several questions or limitations are overlooked. For instance:<br>3.1. It is described that physiological parameters were measured from 10 am to 1 pm, a wide interval with expected changes in environmental conditions affecting these measurements. To understand for possible covariances, it should be indicated if these measurements were carried out simultaneously and following the same order than that of leaf sample collection for the other phenotyping.<br>-Response: We agree that we missed crucial details about the timing of this sampling. To fix this, we included the clarifications that (on L241) all physiology measurements were being taken simultaneously by different groups moving though the vineyard and (on L243-245) the measurements were all taken in row order ensuring that the vineyard blocking factor captured temporal variation. SImilar notes were added for the other phenotypes to better explain sampling. As was noted in the next reviewer comment, block is missing from Figure 5 which means it was not significant as a main effect. |

Powered by Editorial Manager® and ProduXion Manager® from Aries Systems Corporation

3.2. Related to the previous, why block effect alone was not considered for physiological measurements in Figure 5?
-Response: Thank you for this comment. Block (or row for gene expression) was included in the models as a main effect for every modality (see analysis section). Non-significant factors were dropped from figures summarizing linear models; because the block main effect was not significant, it was not included in Figure 5. .

3.3. Did the horseshoe shape for row effect on the transcriptome correlate with oscillation of environmental/circadian clock conditions during the sampling interval or with vineyard heterogeneity? Functional analysis of the genes contributing to row effect could be informative on the origin of these effects that might have hindered the identification of rootstock effect on the transcriptome.
-Response: This is a really interesting comment. We agree with the reviewer that the horseshoe shape in LDA space is either a function of circadian conditions or spatial heterogeneity. We have added to the Data Description section a comment on assaying genes with known circadian topology (L207-210) and show in Supplemental Figure 2 that those genes are variable over our sampling window. In addition, we commented on this outcome in the Analyses section on L360-362. We show that the impact of vineyard position/spatial variation is weak in other measured phenotypes (captured by the 'block' model term; see, for example, Figure 1A and Supplemental Figure 5A). Future studies should assess potential intra-vineyard variation either through blocking (as we did here) or explicit measurement (for example, soil composition) and control for that variation.

3.4. Is there a rootstock effect on vigor, biomass, fruit fertility and production that could explain or condition the effects in leaf phenotypes that were measured? Were these factors normalized in any way, either by agronomic practices or statistical treatment?
-Response: This is an excellent question that is perhaps beyond the scope of this comprehensive analysis of leaf phenotypes, but one that is certainly an important next step in our research trajectory. Conditioning or normalizing on aspects of vigor or yield or looking for correlates of those traits in early season leaf phenotypes would be immensely valuable to viticulture and a general understanding of grapevine biology. To explore this idea, we have amended the language of the Potential Implications section on L588-608. In addition, some of these data were collected and are being prepared for papers focused toward berry phenotypes. In the meantime, we point to (https://doi.org/10.1002/pld3.324) to show that this is absolutely a valid direction of inquiry for future work and data integration efforts.

4. This study comprises similar experiments to these already published by the same group in the same set of plants (Migicovsky et al., Hort Res 2019), although extended to include metabolomics and physiology data and two additional phenologcal stages. While the effect of phenology is clearly presented here, the addition of the metabolite data is undermined. What are the metabolites determining rootstock effect in Figure 2C? What about metabolites determining a rootstock effect depending on phenology that could be inferred from PC10?
-Response: We thank the reviewer for this comment and appreciate the careful consideration of this manuscript in the context of the Migicovsky et al, 2019 manuscript. We completely agree that the value of the metabolomics data is undermined in the manuscript. This is primarily the result of current challenges in mapping peaks from LC-MS onto named metabolites. The current state of untargeted metabolomics from LC-MS would require significant chemical laboratory work to narrow down the space of potential metabolites. While we believe this work should absolutely be done, our goal with this study was not necessarily to identify specific metabolites but to determine if the metabolome was a potential avenue through which the rootstock is influencing scion phenotypes. To address this, we used only a portion of the runs available to show there is a signal. Future work will focus on merging the various additional LC-MS runs (not presented here) and chemical experimentation to uncover the full scope of this effect. We note that we are uploaded raw data to Metabolights, QC/filtered data to FigShare, and reported the retention times and m/z ratios for the compounds of putative interest in the manuscript. We hope that these data may be useful in future analyses of grapevine metabolites, either by our group or others.

Powered by Editorial Manager® and ProduXion Manager® from Aries Systems Corporation

Minor revisions
1. "Ubiquitous" effects of rootstock genotype are described along the MS. However, since only one location was analyzed (leaves), would "pleotropic" be more appropriate to define the different phenotypes affected by rootstock-scion genotype interaction in this study?
-Response: This is a great point. We clarified our usage of the word “ubiquitous” to ensure its intended meaning (across modalities within leaves, not as an inherent feature of grapevine) was clear (for example, on L514) throughout the manuscript. Supporting the reviewer’s observation that this study only included one environment, we are hesitant to use the word “pleiotropy”, though we are interested in the implication that the different rootstock/scion pairs create different local environments and will consider this idea in future works.

2. Methods on RNA-seq procedures are incomplete. Which sequencing technology was used? Which type and length of reads? Etc.
-Response: Good catch. We added the following to the L194-195: “Sequencing was conducted using the Illumina NextSeq500 platform which returned single-end 86 bp reads.”

3. Inter-annual comparison for anthesis ionomics, transcriptomics and morphology between this study and their previous publication (Migicovsky et al., Hort Res 2019) could enable a broader interpretation of rootstock effects, overcoming the reproducibility limitation of considering only a single season here.
-Response: We absolutely agree that interannual analyses are required for a detailed understanding of the root system influence on shoot phenotypes and these analyses are underway. Our goal with this manuscript was to carefully quantify different phenotyping modalities and to understand how they relate to one another. The results from this study have helped us consider what is worth more detailed investigations, and analyses that address longer (multi-year) studies for those phenotypes are currently in the works. Given the magnitude of the data presented here and the extent of analyses conducted, we struggled to fit this detailed work in a single manuscript that also covered inter-annual variation as well as additional phenotypes (berry chemistry, etc.). As a result of work presented here, we are currently exploring tradeoffs between deep analyses of individual phenotypes and shallower analyses of more modalities over longer time periods, additional scions, and multiple sites. In the meantime, wherever possible we note some comparisons to the Migicovsky 2019 study where appropriate. The Migicovsky 2019 pilot study used considerably different methods for many phenotypes, which preclude direct comparisons.

4. L426. The sentence might not be completely fair as no DEG was identified for rootstock effect (transcriptome phenotype would therefore be mostly unaffected) and developmental stage-specific could be more adequate than season-specific.
-Response: Thank you for this. We agree with the suggested change in language for the effect of phenology and changed “season specific” to “specific to the vine’s developmental stage” on L468. On gene expression, while no DEGs were identified, we were able to identify latent combinations of genes that were responsive to rootstock treatment. While this effect is subtle, it was nonetheless detectable.

5. Any biological interpretation of the specific metabolites, genes, iones, shapes determining the resulting PC covariation networks? While it can be interesting to add to covariation networks additional levels of phenomics as authors propose (lcRNA, micorobiome, epigenetics), it would also be informative to exploit the interpretation of the dataset that they have already produced.
-Response: Excellent suggestion. Unfortunately, after much consideration, at this point we do not feel comfortable with detailed biological interpretations based on specific metabolites or genes that underlie PC covariation networks shown here. Some of the limitations of our dataset, and why we are unable to make these mechanistic connections with data presented here, are detailed in the discussion. We note that the ionome offers a very rich source of data ripe for deep analysis, and that an additional manuscript describing a deep dive into multi-year, multi-time point ionomic dataset is in preparation now. We agree that future work should be targeted toward biological understanding of these relationships. On suggesting inclusion of other phenotypes, this comment reflects our enthusiasm for other existing approaches and exciting areas of

Powered by Editorial Manager® and ProduXion Manager® from Aries Systems Corporation

research that might further uncover mechanistic understanding of the effects we are seeing from grafting and over time. The analysis presented in the paper, unfortunately, does little to advance us toward the goal of mechanistic understanding, but it does help us see where future studies could be targeted. To this end, we added language to clarify this point on L573-578.

6. L470, If the lack of rootstock effect on the transcriptome was due to the phenology effect, specific analysis at each phenology stage would identify rootstock genotype factor significant DEGs. Is it the case? Would there be any rootstock effect detected on transcriptome if the analysis was restricted to single blocks at specific phenological stages?
-Response: We thank the reviewer for this comment. This is certainly something we are trying to wade through as our results suggest that the rootstock influence on our vines is incredibly complex and works through interaction with other factors of the experimental design. Ongoing work is focused on identifying these complex effects, in a statistically robust way. We are also currently working with collaborators to identify genes and gene regions worth further exploration. Moreover, we are seeking to use results from other phenotypes to focus on genes in a more 'hypothesis-driven' approach that can further the 'discovery-driven' results observed here.

7. Apart from the seasonal effect, the "Potential implications" presented are not directly inferred from the Results obtained here but from the potential of the approach used. Any other potential implication of the specific results?
-Response: This is a very helpful suggestion. In response to this and comments from other reviewers we have re-worked the potential implications section. Other reviewers called for an enhanced focus on yield/viticultural implications, while others have asked us to minimize such speculation. Consequently, we have attempted to carefully place this work in the context of both basic plant biology and viticulture. If the current revision does not meet the expectations of the reviewer(s) or editors we would be happy to revise further.

8. Is there any data available for the distribution of soil properties across the experimental plot that could be considered to discuss the origin of block effects? Could the human factor during that extensive sampling be another variable accounting for block effect?
-Response: Thank you for this excellent observation. For the data presented here, we do not have paired soil samples. We anticipate some heterogeneity in soil properties across the experimental plot; however it is unclear how strongly this would correspond directly to block effect. Regarding the human factor, we have added a sentence into each data modality clarifying what variation is captured by the blocking factor. See each addition below:
-L136-138 added, "Teams were deployed in the vineyard so that multiple vineyard rows were being sampled concurrently. As such, 'block' represented unmeasured spatial variation, but did not strictly correlate with time of sampling due to the nature of sampling (see Methods)."
-L155 - 157 added, "ensuring that 'block' captured both unmeasured environmental variation and temporal variation over the sampling window".
-L187-190 added, "Leaves were sampled by a single team near midday between 10AM and 2PM in row order ensuring that 'block' and 'row' accounted for unmeasured spatial variation and temporal variation over the sampling window (see Methods)"
-Overall, block is not a large descriptor of variation in our study except for the phenotypes for which block is collinear with time of day. In these phenotypes (the metabolome and the transcriptome) there is a noted circadian topology. The other phenotypes (ionomics, leaf shape, and physiology) see little effect from block suggesting there is little spatial variation (or at least that the spatial variation is unimportant for those phenotypes).

9. Because half of 3309C reps would have been collected before any ungrafted rep was taken, could the LD2 effect in discriminating 3309C and ungrafted from RNA-seq data be related with sampling times? What are the genes involved in this effect?
-Response: We thank the reviewer for this comment. While it is always possible that results correlate with unmeasured confounders, rootstock genotype was not

Powered by Editorial Manager® and ProduXion Manager® from Aries Systems Corporation

confounded with any of the terms in our model (including time of sampling, which was a correlate for row in our study; L653-655). Each rootstock was present in each row of the vineyard in cells of four replicated vines (See supplemental Figure 1A). For the transcriptome sample, we sampled leaves from the middle two vines in each cell. While it certainly takes time to sample in a vineyard, each rootstock in each row would have been sampled within minutes of one another. Due to this, the grouping we report in LD space is not confounded with time. The current results suggest that this effect is driven by complex combinations of genes (from the PCA results) and not any particular genes being strongly affected (from the traditional DGE results). We are currently working on exploring these subtle effects in more statistically robust ways in a multi-year study.

10. Any discussion on the origin of leaf position effects in specific ions?
-Response: Thank you for this comment, and this is something we think about often. Leaf position was added to this study on the ionome because it is known that leaves vary in their elemental composition over development. The major question here was whether or not rootstock would influence the elemental composition of those leaves in such a way that the known patterns might be interrupted. While we observed significant variation in ion concentrations as a function of leaf position, it did not strongly interact with rootstock genotype. In other words, the rootstock effect was present in all leaves, not just leaves of a particular age. As such, we struggled to fit a detailed description of the effect of leaf position in the current paper. However, we have made all data from this analysis publicly available if there exists specific interest for the leaf position. Ongoing work focused explicitly on the leaf ionome will provide a deep dive on how ion concentrations vary by rootstock, over development, across seasons, and across multiple years.

11. L556. Indicating in there that "only the middle two vines of the four cells in the front half of the vineyard were included in the 72-vine set" would be handy to understand the distribution of this set.
-Response: Thank you for this comment. We have clarified the description of the experimental vine throughout the manuscript. The experimental design of the vineyard included groups of four identical vines (e.g., Chambourcin grafted to 3309C) that are distributed in a randomized block experimental design throughout the vineyard. For some data modalities we were unable to process samples from all four vines per cell. In these circumstances, we opted to collect samples from the middle two vines of the four vine set. We collected from the middle two vines from a total of 36 cells for the 72 vine set. To improve the understanding of this section, we have amended the text to point to more appropriate sections of Supplemental Figure 1. In addition, we have improved the Figure Legend for this figure so it is more clear what each panel is showing with explicit descriptions for Supplemental Figure 1B, which should improve the clarity of this section. Finally, we clarified that this description only applied to the front half of the vineyard which was missing from the previous draft. We would be happy to make additional edits to the text if this description does not provide sufficient clarification.

Reviewer #2
The manuscript by Harris et al investigates the effect of grafting on a number of physiological and molecular phenotypes within grapevine (Vitis spp.) scions. The hybrid Vitis cultivar Chambourcin was compared when grown on its own-roots, or when grafted to three different commercial hybrid rootstocks: 1103P, 3309C, and SO4. The vines were grown in the field, irrigated with different volumes of water, and sampled over a single growing season. Large data sets have been generated for leaf metabolites, solutes (ions), transcripts, shape, and physiology (stomatal conductance, transpiration). As such, the manuscript fits the scope of Gigascience well. The manuscript is well written, however I found it was very statistical and would benefit from additional biological analyses to confirm and validate the findings. The methods section is lacking some details that would enable reproducibility. Some of the figures could be improved for readability. My comments and suggestions are detailed below:

Major comments
1. There is no information on the age of the vines at the time of the experiments.

Powered by Editorial Manager® and ProduXion Manager® from Aries Systems Corporation

-Response: This is a great observation of information we overlooked. The vineyard had been in the ground for eight years at the time of sampling. The age of the vineyard has been added to L538 and commented on in the discussion.

2. A quantitative analysis of the elemental content of the irrigation water by ICP-MS would be beneficial. In this study, it is unknown whether the irrigation treatments contained varying levels of the elements that were measured in leaves. To this end, it is perhaps not surprising that rootstocks had minimal effect on, for example, the Na+ content of grafted scions. However, it has been demonstrated previously that own-rooted vines cannot efficiently exclude Na+ compared to grafted vines when irrigated with 100 mM NaCl (see Fisarakis el al (2001) Agricultural Water Management 51 13-27).
-Response: This is a really intriguing suggestion, and one we wish we would have thought of in 2017. Regrettably, it was not something that was considered for this study at the time. However, we can be confident that irrigation was not significantly altering the findings of our study due to the weak nature of the irrigation effect (See Supplemental Note 1). Had there been variation in the ionome of the irrigation water, we would have expected to see a stronger irrigation or irrigation by phenology effect, neither of which were strongly observed in this study. We thank the reviewer for pointing us to this helpful article.

3. The manuscript would be more useful to the plant science community if a subset of the actual metabolites and genes identified within the principle components were named and confirmed using a second method. It would then be possible to discuss which physiological, metabolic, and molecular processes within Vitis scions are impacted by rootstock selection.
-Response: We absolutely agree gene-level and metabolite-level understanding of the root system influence on shoot system phenotypes is the direction this work needs to head. This is perhaps one of the biggest limitations of large-scale analyses of multi-dimensional phenotypes: it is sometimes hard to narrow in on individual phenotypes for some systems. We acknowledge that there is a trade-off between large-scale analyses like the one presented here and identification of actual metabolites/genes and their functional role in the vine. We see these as very complimentary approaches that illuminate different aspects of vine biology; however, we were unable to do both in this study. Ongoing work is attempting to, in a statistically robust way, uncover those subtle effects from even deeper sampling of the transcriptome. The metabolome as described using the untargeted approach here is a whole different monster. We were able to show that some metabolites are responding to the rootstock treatment and even to the rootstock by season interaction. The current nature of LC-MS and untargeted metabolomics in Vitis generally make it incredibly non-trivial to map these metabolites. Here we sought to catalog the basic responses of multiple phenotypes to help guide more targeted analyses and guide us toward studies that could produce mechanistic understanding.

4. Similar to my comment above, some of the data could be integrated. For example, transpiration was increased for scions grafted to 1103P (Fig 5B). Were genes or metabolites involved in the regulation of stomatal aperture differentially abundant when grafted to 1103P?
-Response: This kind of data integration is an excellent suggestion. While such analyses would require work beyond the scope of this paper, we think that this comment is exactly in line with how we should be guiding future work. We proposed in the previous comment that this work was meant as a foundation on which we establish the basic responses of many complex phenotypes over the growing season with respect to the rootstock genotype. The PCA-based integration was to help us narrow down which types of data modalities warrant future integrative work. As we move toward identifying and annotating individual genes and metabolites, these suggestions will certainly help in that future planning.

5. The ionomics data in Fig 1B and C would be easier to interpret if presented as a percentage - for example, % DW, % FW, or mM of tissue water. Currently, there are no units on the Y-axis.
-We agree that z-scores were not the ideal choice for this figure. To address this, we remade this portion of this figure to show the elements as concentrations in parts per million of acid-digested dried leaves. We have updated the Figure Legend L706 and

L710 to reflect this change.

6. There is no mention of how the RNA was extracted from plant tissues. Further, a quality control would normally be performed, e.g. by measuring the 260/280 ratios at the very least. Was any quality control performed on these RNA samples? How do we know the samples were pure and not degraded?
-Response: Excellent point that was also noted by Reviewer 1. The information requested has been added to the Data Description section, L191 - 193: "Total RNA was extracted from plant tissues using the Sigma Spectrum Plant Total RNA kit with modification of the addition of 2% PVP40 to the extraction buffer to decrease phenolic inhibitors. All RNA extractions were checked for quality control using a Nanodrop. Sequencing was conducted using the Illumina NextSeq500 platform which returned single-end 86 bp reads."

7. It is unclear how many biological replicates were used for the RNAseq experiments.
-Response: Good catch- thank you for this. Language changes were made throughout the manuscript (in conjunction with other review comments in the section "Study Design" and within the Data Description for each modality to improve clarity. In short, each modality was sampled from either a 72-vine set (metabolomics, gene expression, physiology) or a 288-vine set (ionomics, leaf shape). In the 72-vine set, we only sampled the middle two vines from each four-vine cell in the vineyard (shown in Supplemental Figure 1B-C). At the highest order interaction for gene expression (rootstock:row:phenology), the number of biological replicates would be two. Since this is clearly underpowered, we put little effort into estimating or interpreting those effects. However, lower order interactions (like rootstock:row or rootstock:phenology) are averaged over the remaining samples. For example, the rootstock:row effect is estimated from 6 samples (averaged over phenology). Similarly, main effects are averaged over all other terms, so the rootstock effect would be estimated from 18 samples.

8. Usually, for genome-wide transcriptional studies, the expression patterns of a subset of genes are confirmed using another method (e.g. quantitative real-time PCR). This has not been performed in this manuscript. Authors need to confirm the validity of the RNA seq dataset.
- Response: Thank you for this comment. We agree that for studies focused on identifying genes associated with specific phenotypes, that the gold standard for genome-wide transcriptional studies is cross-validation using qPCR. The goal of this study is to understand the influence of root system genotypes on shoot system phenotypes, and here we are treating gene expression in the leaves as a shoot system phenotype. Because we are not testing explicit hypotheses about any particular gene or pathway, we determined that PCR-based confirmation would not add value. This decision was not made lightly, and was done following consideration of other recent work that applied various RNAseq platforms to address structurally similar questions, including:
1) Griffith M, Griffith OL, Mwenifumbo J, Goya R, Morrissy a S, et al. (2010) Alternative expression analysis by RNA sequencing. Nat Methods 7: 843–847. Doi:10.1038/nmeth.1503.
2) Asmann YW, Klee EW, Thompson EA, Perez E a, Middha S, et al. (2009) 3' tag digital gene expression profiling of human brain and universal reference RNA using Illumina Genome Analyzer. BMC Genomics 10: 531. Doi:10.1186/1471-2164-10-531.
3) Wu AR, Neff NF, Kalisky T, Dalerba P, Treutlein B, et al. (2014) Quantitative assessment of single-cell RNA-sequencing methods. Nat Methods 11: 41–46. Doi:10.1038/nmeth.2694.
4) Shi Y, He M (2014) Differential gene expression identified by RNA-Seq and qPCR in two sizes of pearl oyster (Pinctada fucata). Gene 538: 313–322. Doi:10.1016/j.gene.2014.01.031.
-Moreover, we point to an excellent blogpost on the same validation-discussion that goes further in to this debate:
http://dave-bridges.blogspot.no/2014/11/validation-of-rnaseq-experiments-by-qpcr.html?m=1
-We agree that it adds value to ensure that our data show previously established patterns of house-keeping and circadian-phased genes relevant to our sampling

paradigm. As such, the following have been added to the manuscript:
-Added to data description L207 - 210, “To check the validity of our expression results, we assayed two classes of housekeeping gene (Ubiquitin-domain and actin-family) and eight previously annotated circadian genes (Carbonell-Bejerano et al. 2014)”
-Added to Analyses, L339 - 341, “We computed the expression of two classes of housekeeping genes, and show that they are generally stable (Supplemental Figure 2). We note that some variation is expected for housekeeping genes; see, for example, (Liang et al. 2018). Moreover, we show that patterns of previously annotated circadian genes show expected results over the sampling window. For example, predicted orthologs of LHY and RVE1 are correlated and decreasing over our sampling window, and a predicted TOC1 ortholog is invariant. That our samples showed such patterns suggested the gene expression data presented here were valid.”
-Created and added Supplemental Figure (now supplemental Figure 2) showing these patterns.

9. The effect of the different irrigation regimes is not adequately discussed in this manuscript.
-Response: Thank you for this note - good catch. Effects of different irrigation regimes were originally presented in a Supplemental Note hosted on GitHub. To increase accessibility, we moved Supplemental Note 1 from GitHub so that it is now a Supplemental Note to the manuscript.
-Some additional notes: our study site is located in southwestern Missouri where it can be quite rainy. In 2017, the year in which samples were collected for this study, it rained a lot, essentially rendering the vines all properly irrigated despite the amount of irrigation applied as part of the study. Not surprisingly, our physiological metrics showed little/no evidence of stress. As such, we opted to include irrigation as a term in the model that is there and could impart variation into some phenotypes, but those effects are very small.

10. The Abstract must be structured into three separate sections: Background; Results; Conclusions.
-Response: The abstract has been restructured to meet the suggested format. We thank the reviewer for catching this oversight.

Minor comments
1. A lot of the information under "data description" should be moved to the methods section. For me, the data description should provide more of a background and rationale of the work, while the methods should provide the actual steps that were taken.
-Response: Thank you for this note. We struggled to balance the need to provide enough information for readers to understand the work up front, while saving the majority of methodological details for the methods section. In its current version, we hope that we have provided the appropriate information in the approximately preferred locations according to the journal’s instructions. If there are persistent issues with information placement in the current version of the manuscript, we would be happy to address those in whatever way the editor/reviewers request.

2. Although it is alluded to in the introduction and data description, the tissue type that was harvested and used for the RNAseq experiments is not mentioned in the methods or analyses sections.
-Response: Good catch, and we regret this omission. The tissue type used for RNAseq experiments were young, fully opened leaves. We added information about the tissue type in the analysis section, and assured it was explicitly mentioned in the data description.
-L185 starts “The youngest fully-opened leaves”
-L336 added “youngest fully-opened leaves”

3. The Figure 1B legend should denote what Y, M, and O mean. I realise that it is young, middle and old, but the legend should stand alone.
-Response: Another good catch. We added a short key to L705 - 706 indicating Y (young), M (middle), and O (old).

4. Significant differences in Fig 1B and Fig 5 B&C could be annotated within the

Figure, for example with an asterisk.
-Response: Excellent suggestion. Significant comparisons have been labelled with letters in the identified figures, and the figure legends have been edited to explain them.

5. Please be careful to use the past tense consistently, for example P16, L403 "correlation between gPC4 and pPC3 is similar" should be 403 "correlation between gPC4 and pPC3 was similar".
-Response: Thank you for this important catch. Tense was changed in the cited examples and edited throughout the manuscript.

6. P19, L472 "stomatal conductance were higher vines" should be "stomatal conductance were higher in vines".
-Response: Added 'in' to L512

7. P19, L475 "Understanding of rootstock genotype influence shoot system phenotypes" should be "Understanding of how rootstock genotype influence shoot system phenotypes".
-Response: Edited L516 to read, "Understanding rootstock genotype influence on shoot system phenotypes"

8. Perhaps consider re-writing the title to the Fig 5 legend. "Vine physiology measurements show signal from most experimental manipulation" does not make sense to me.
-Response: Edited title on L746 to "Vine physiology varies with rootstock and the rootstock by phenology interaction"

Nice work.
Thank you for this. We appreciate the detailed review.

Reviewer #3
This study investigate associations between rootstock genotype and shoot system phenotypes using five multi-dimensional approaches contributing to elucidate how root systems influence vine phenotype.

the influence of rootstock on the traits analyzed are roughly well documented in literature and authors are aware about this since they very often commented that results are consistent with previous study. Hence the reader might question about the limited new information provided. I would recommend the authors at the "potential implications" paragraph to avoid speculation on "yield" and to emphasis the novelty of engaging a simultaneously analysis as they did in order to speed up comparative studies.
-Response: This is a very helpful suggestion. In response to this and comments from other reviewers we have re-worked the potential implications section. Other reviewers called for an enhanced focus on yield/viticultural implications; however, we agree with this reviewer's request to minimize speculation. Consequently, we have attempted to carefully place this work in the context of both basic plant biology and viticulture. If the current revision does not meet the expectations of the reviewer(s) or editors we would be happy to revise further.

Minor comments
1.At line 226-227, check "umol/s" replace with [?]mol s-1 ?
-Response: Unfortunately the symbol the reviewer suggested did not render in the communications (we can't see it). We have replaced the umol with the more commonly accepted µmol where appropriate. If another symbol is preferred or a different symbol was meant, please let us know and we would be happy to make the requested change.

2.At line 231, is 15 min interval time enough to equilibrate? Considering that usually 30 or 60 min are required (e.g., J.Int.Sci.VigneVin, 2012, 46, n°3, 207-219, See https://urldefense.com/v3/__https://doi.org/10.20870/IVES-TR.2020.3620__;!!K543PA!bnhJBaYGb-6O8nkV-F90YaIlxoa2UGVyHkLiToTGXSjDbduO2MrZFPISJayIAoKRcnAWzw$See ISBN 978-90-481-9282-3 at pag 89), please justify your 15 min interval.

Powered by Editorial Manager® and ProduXion Manager® from Aries Systems Corporation

| | |
|---|---|
| | -Response: We thank the reviewer for this thoughtful comment. We acknowledge that this is a topic of much debate. A 15 minute equilibration has been used in the past to measure midday stem water potential for tree species, and explicit testing showed that there was little difference between a 10-15 equilibration and a >1hr equilibration in oak trees (https://www.fs.fed.us/psw/publications/documents/psw_gtr184/psw_gtr184_035_ShackelGross.pdf). A more recent study (published after our work was completed) suggests that there is a small effect from different equilibration times in grapevine, but that effect is smaller than the effect from the person operating the pressure chamber (https://doi.org/10.1016/j.agwat.2019.03.026). We have amended the section of the manuscript to include these references for future readers (L254-255).<br><br>3.Please note that "old" and "young" communicate leaf age rather than leaf position, what's about top, middle, bottom?<br>-Response: This has been a topic of much debate on our team and we really appreciate this comment. The designation of “old”, “middle” and “young” stem essentially reflect terminology used by our team since the inception of the project. We totally agree that these terms reflect leaf age rather than position. However, in grapevines these are equivalent because the oldest leaf along a vine is at the bottom of the shoot and the youngest leaves are at the top of the shoot. All things being equal we would readily make this change; however, the current terminology is used in this paper and in many other completed or ongoing manuscripts being carried out by members of our team. If it is amenable to the editor and the reviewer, we would prefer to retain the “old” “middle” and “young” designation. However, if this is unworkable we will make changes to the language.<br><br>4.It is not clear why 1103 P had a very little variability of gs at anthesis compared to other rootstocks, for these plant water status seems to range from well irrigated to deep stressed vines while 1103P vines seem to be all roughly well irrigated.<br>-Response: We appreciate this observation. It is not immediately clear why vines grafted to 1103P showed such little variation in stomatal conductance at anthesis. Unfortunately we don’t think we can test this with the current study. To investigate this and related questions we completed a greenhouse study with 1103P and other rootstocks grafted with a common scion with an irrigation treatment. This work is in preparation now.<br><br>5.Providing VPD data might help to explain why transpiration is low at anthesis (approx. 2.5 mmol m-2 s-1) while gs at anthesis is comparable to that of other sampling time.<br>-Response: Thank you for this interesting point. We agree that features of the environment (like VPD) will partially explain the differences we see across the time point in this and future studies. Ongoing work is attempting to identify features of the environments that correlate and can explain some of the variation we see in these traits. This is partially undermined by natural season changes, so these relationships are hard to untangle and require a substantial amount of data, much beyond the three time points presented here. However, we appreciate this comment and hope to address this in future works.<br><br>6. "leaf position" should also be discussed against "leaf angle" (e.g., https://urldefense.com/v3/__https://doi.org/10.3389/fpls.2020.00595__;!!K543PA!bnhJBaYGb-6O8nkV-F90YalIxoa2UGVyHkLiToTGXSjDbduO2MrZFPISJayIAoLo-b4lwA$ ) which likely change across the season due to change of soil water availability. was leaf angle accounted for image analysis? Considering that soil moisture reasonably differed at the three stages considered (Fig. 5).<br>-Response: Thank you for this insightful comment. We agree that leaf angle is important for physiology, and would likely correlate with some of the traits we measured. Unfortunately, leaf angle was not quantified at the time of collection in the field. Leaves were simply chosen from vines that emerged directly from the cordon and had intact young, middle, and old leaves. Leaf scans were completed in the lab after leaves had been removed from the vine, and it was not possible to quantify leaf angle at this time. Having said that, this is an important consideration for future studies and we very much appreciate this observation. |

Powered by Editorial Manager® and ProduXion Manager® from Aries Systems Corporation

7.Please add the mean leaf water potential and soil moisture values directly in the Fig. 5 panels to help the readers.
-Response: We thank the reviewer for this suggestion on improving our figures. We have added the mean value for reach rootstock/phenology combination in Figure 5 and, for consistency, to Figure 1. Figurel legends have been edited to address these changes on L712 and L751.

Reviewer #4
This 'big data' manuscripts offers a comprehensive snapshot of the grape phenome as influenced by several factors, including ionomics, leaf morphology, physiological data, metabolomics and transcriptomics. The overall scope is ambitious and a step forward for the phenomics community. Overall the paper was well-written and the design and analysis are sound, though I had a few questions below.
- Response: Thank you for these very kind and encouraging words.

I had a few suggestions.
1.Phenomic and phenotypic are used interchangeably, and I would ask they be clearly defined - should they really mean the same thing? What's the difference between a phenome and a phenotype?
-Draft response: Thank you for this important observation. We define "phenomics" to be a "field characterized as the acquisition and analysis of high-dimensional phenotypic data at hierarchical levels, often with an eye toward multiscale data integration" in the introduction. We define a phenotype as a single particular trait (e.g., calcium concentration). As such, we have amended usages of these words to comport with this definition: phenomic (and phenomics) now refer to the joint analysis of multiple data modalites, each of which contain several phenotypes (or a single multi-dimensional phenotype). In addition, we recognize that we were being imprecise with language here, so we have fixed this and other terms used interchangeably (trait/phenotype, phenotypic/phenomic, and data type/modality) throughout the manuscript.

2.Is the paper considering transcriptomics as phenomics? I know it's a debated issue really, but would be good to state so and why.
-Draft response: Thanks for this comment, like many groups we have spent a lot of time thinking about the question of whether or not the transcriptome is a phenomic modality. In the introduction of this manuscript, we loosely acknowledge phenomics as the field of study concerned with high-throughput data acquisition through multiple simultaneous trait measurements, often requiring advanced computation to analyze and integrate L62-63. Following this definition, we treat the transcriptome as a multi-dimensional phenotype (or that the extent to which a particular gene is expressed at a particular time in a particular place is a measurable trait/phenotype). In the analysis and interpretation of the data in this manuscript, we treat the transcriptome like the other data modalities presented here.

3.Related, phenotype and trait are inconsistently used as detailed below. I recommend to define them and use consistently. This is a huge problem for phenomics and I think prevents clear discussion of the topic.
-Response: We thank the reviewer for this comment on clarity. Throughout the manuscript we have edited the language we used to describe phenotypes to be consistent. In particular, we have edited each usage of 'trait' to 'phenotype. As above, we recognize that we were being imprecise with language, so we have fixed this and other terms used interchangeably (trait/phenotype, phenotypic/phenomic, and data type/modality) throughout the manuscript.
-

4.I had some questions about the experimental design and randomization, detailed in line comments. I'm not sure about the claim of 72 replicates. Maybe it's a question of what should be considered an experimental unit.
-Response: Other reviewers also noted lack of clarity with respect to experimental design, and we appreciate this observationt. A full response to this concern can be found in our response to your comment on L561 (below); which is partially copied here: I think some additional confusion may stem from us using "replicate" as a vague stand-in for both clonal replicates and statistical replicates. To address this, we have

Powered by Editorial Manager® and ProduXion Manager® from Aries Systems Corporation

| | |
|---|---|
| | amended the language about the four rootstock scion combinations as follows on L617: “Clonal replicates of each of the four rootstock-scion combinations were planted 72 times for a total of 288 vines planted in nine rows”. In addition, we included the specific type of design (split-plot) to this section. Finally, we addressed the number of true replicates in a comment by reviewer 2 concerning RNAseq. The same logic can be used to derive the total number of biological replicates for leaf shape and ionomics at the highest order interactions (4) and for all other phenotypes (2). In the case where the number of biological replicates is two, the estimation and interpretation of effects is minimized due to lack of power.<br><br>5.The analysis of individual datasets (or modalities, good word) seems good, and I think the approach to combine into a larger set using the PCA is pretty clever. I still wondered how 'fused' the data really is but can't really think of a better way other than combining all the raw data except then the number of genes and metabolites would just swamp the analysis I guess. Perhaps the authors could articulate why this is a good fusion approach they've used, and perhaps what could be done in the future.<br>-Response: Thank you for this kind observation and really insightful comment. We considered a larger integrative framework that would include all phenotypes measured in the study. However, as the reviewer identified, this would include a heavy bias toward gene expression (expression data for 24,000+ transcripts) and metabolomics (600+ different features measured) which would likely overpower leaf shape (17 x,y coordinates) and ionomics (20 ions). We felt that the PCA approach allowed us to weigh each modality more evenly in order to see if further integrative efforts were warranted. Based on these high-level results, it looks like integration among modalities is a warranted effort, especially if we could collect more targeted data that could expand mechanistic understanding of observed patterns. However, the scope of these integrative techniques is broad and several papers could likely be written just exploring differences in integration techniques with just a single phenotype, for example, gene expression. We have edited the sentence on L430-431 to reflect this logic: “Within each phenotyping modality, we summarized the primary dimensions of phenotypic variation using PCA (see Methods), so as to not weigh any modality too heavily.”<br><br>6.I Biologically, I'd like to see more insights to why these traits matter. How could understanding that these traits change help production? I think some arm waving is warranted. Especially, how is understanding the correlation among modalities important? One idea is to identify trade-offs and synergisms?<br>-Response: We thank the reviewer for this suggestion. We modified the language of the potential implications to suggest some ways in which this kind of work could balloon into other phenotypes (not measured for this study) that are more useful to breeding through synergistic relationships (enhancement), trade-offs (constraint), or just simply predictability. Moreover, we maintain that the broadest implication is the notion that there is a strong temporal component to phenotypic expression in long-lived perennial plants and that grafting and rootstock genotype add another dimension to it.<br><br>7.Last, I'm happy to see how much data is shared. However, GitHub is not appropriate for sharing data, which should all be on a public repository, including the analysis scripts. I think FigShare has been used for other permanent data, so I recommend to share the scripts there.<br>-Response: We thank the reviewer for this insight. The note on irrigation, which was initially uploaded to GitHub, has been added as a Supplemental Note to this manuscript. This note will additionally stay on github for easy access. All phenotypic data from the ionome, metabolome, leaf shape, and physiology are on Figshare and the gene expression are on the SRA. In addition, we are in the process of submitting raw metabolomics data to the Metabolights database, as requested by GigaScience.<br>Line comments and other details follow:<br><br>39: In my opinion, the 'hyphens' are not needed in belowground and aboveground.<br>-Response: From what I can gather, above-ground and aboveground are considered to have the same meaning. We leave this stylistic choice up to the editor.<br><br>45: "change"<br>-Response: L46: changes -> change |

Powered by Editorial Manager® and ProduXion Manager® from Aries Systems Corporation

46: long sentence with semicolon, consider making that a period, but the use of many interjections make it a little hard to parse still
-Response: Good catch. This sentence has been split into two (now L44).

99: are phenotypes and traits taken to be completely synonymous in this paper? Given that many definitions are used of each, it would be helpful to define. For example, both can be used to describe the 'general' properties like 'eye color' or the specific like 'blue eyes.' Phenotype, in addition, is sometimes used to describe the totality of all trait values in an organism. More careful and exact usage would benefit the paper. For example, trait value can also describe the specific like 'blue eyes' while trait the general 'eye color.' The title of your paper suggests that you additionally consider the phenotype as all traits (or trait values?). Leaf shape is referred to as a phenotype at line 439, so consistent with the 'general trait' definition.
-Response: We agree this was a persistent problem in the initial version of the manuscript. As above, we recognize that we were being imprecise with language, so we have fixed this and other terms used interchangeably (trait/phenotype, phenotypic/phenomic, and data type/modality) throughout the manuscript.

111: what would the difference bet tween phenomic and phenotypic variation? what is the definition of the phenome? phenotypic variation is also used at line 434
-Response: Another good catch. We have edited the language throughout the text so that it is now consistent. We did not actually mean to distinguish between these two things in the highlighted example. As above, we recognize that we were being imprecise with language, so we have fixed this and other terms used interchangeably (trait/phenotype, phenotypic/phenomic, and data type/modality) throughout the manuscript.

115: Were there any hypotheses? Is the intent to be descriptive?
-Response: The goals of this study were to address three questions: 1) what is the influence of root system genotype on shoot system phenotype? 2) How do systems of plant phenotypes vary over the growing season and does rootstock genotype influence this variation? And 3) how do phenotypes covary within and between phenotyping modalities? For clarity, we have enumerated these questions in the Study Design section. L639 - 642.

118: Are the details of the experimental design needed here because of the wonky format of a GigaScience paper with methods at the end? Not your fault, but I find these formats so confusing and redundant since authors try to move methods into other sections to make up for it.
-Response: We appreciate this comment. This was certainly a design choice by us so that the paper could be understood linearly.

139: if this pipeline is capitalized and sort of 'official' - is there a citation or access to details of it?
-Response: This is a standardized pipeline at the Donald Danforth Plant Science Center. The sentences surrounding this line (now L140-146) have been restructured to make this more clear: “Between 20 and 100 mg of leaf tissue was acid digested and 20 ions were quantified using inductively coupled plasma mass spectrometry (ICP-MS) following standard protocol of the Donald Danforth Plant Science Center (DDPSC) Ionomics Pipeline [30,31]. Ion quantifications were corrected for internal standard concentrations, instrument drift and by initial sample mass. The output of the Pipeline contained measures for each of the following 20 elements: Al, As, B, Ca, Cd, Co, Cu, Fe, K, Mg, Mn, Mo, Na, Ni, P, Rb, S, Se, Sr, and Zn.”

140: Why the difference for ML?
-Response: Z-scores were used in the linear models for ion concentrations so that models could be compared. However, the random forest is a single model that needs no adjustment on the input space. We included a small comment that non-standardized input is the convention for random forests (however many ML models do need to be standardized to equally weight each feature).

141: This Leaf Ionomics section, to me, describes the method to sample and measure, but fails to describe the final output? How many ions? which?  I don't fully understand

Powered by Editorial Manager® and ProduXion Manager® from Aries Systems Corporation

| | |
|---|---|
| | why GigaScience requests this format, but it does mention the background should be given. SO, I think you should say why the ionome is important, and the same for other trait conglomerates mentioned in the paper.<br>-Response: This is helpful - thank you. We have added the following:<br>- to L145-146 to explain the ionomics data set, “The output of the Pipeline contained measures for each of the following 20 elements: Al, As, B, Ca, Cd, Co, Cu, Fe, K, Mg, Mn, Mo, Na, Ni, P, Rb, S, Se, Sr, and Zn.”<br>-To L179 to explain the metabolomics data set, “The 661 identified metabolomic features...”<br><br>143: carbon-based molecules? For example, not nutrient ions?<br>-Response: Added “mostly organic” to enhance description of the metabolome<br><br>144: I had to look up 'veraison' - could you put 'ripening' in parentheses if that captures that idea?<br>-Response: Clarified as the “onset of fruit ripening” on L153<br><br>210: scanning details? background, color, DPI, image format?<br>-Response: L228 - 229: added “in color against a white background at 1200 DPI and written as JPEG formatted images”.<br><br>236: recommend to again announce the number of ions analyzed<br>-Response: Added “and measured the concentrations of 20 ions” to L262<br><br>244: It's not clear what the percentage refers to, I imagine percentage of total variation accounted for by that factor, ie the effect size. recommend to include 'effect size'<br>-Response: Added variation explained to first usage<br><br>249: Giving the effect sizes is a reasonable summary given your multiple factors, however I think giving some indication of absolutely changes is also relevant? Like, what type of percent changes were observed across all the samples in absolute terms, or give the min and max for some ions? Obviously you can't be exhaustive, but this would put the effect size in some type of context of biological influence, like rootstock explaining 10% of variation in a 1% change in Ca vs a 100% change in in Ca. Hope that's understandable. Perhaps these absolute changes would be most relevant where you highlight the influence of rootstocks?<br>-Response: Thanks for this thoughtful comment. The value of effect sizes like percent variation explained are that they can be directly compared if the models are parameterized in an identical manner. However, I think this confusion could be clarified by projecting samples back into a real concentration space which has now been done for the figure.<br><br>267: Could MDA be spelled out on first mention?<br>-Response: L293 now includes Mean Decrease in Accuracy. It is also defined in the methods.<br><br>350: personal placeholder to check discussion for how so much variation isn't accounted for - seems surprising!<br>-Response: We agree that the lack of variation explained in the models for leaf shape is quite interesting. Future work will certainly explore factors such as variation imparted from individual vine and environmental variation to attempt to explain this.<br><br>400: I'm confused that the PCs should correlate from the same modality, something which I thought didn't usually happen?<br>-Response: There is statistical literature on this topic. In short, principal components are orthogonal, however orthogonal does not always mean uncorrelated. See Rodgers, Nicewander, and Toothaker, 1984.<br><br>462: Good to bring up the biological implications - what are they? Are these changes relevant for growth, taste, etc?<br>-Response: We agree that the earlier version of this manuscript was missing key information about why the ionome is important. Unfortunately, there is not a lot of work tying together the elements of the ionome that we identified as responsive to rootstock genotype. Traits of biological interest, features that are known to be influenced by ion |

Powered by Editorial Manager® and ProduXion Manager® from Aries Systems Corporation

uptake by the root system (rootstock) are now mentioned in the manuscript, including vine growth and fruit/wine quality. We have added a comment on this in the discussion to address that there is a known connection between macronutrients and these traits, but more work is needed is to uncover these connections with micronutrients in grapevine. To this end, we added the following to L518 - 520: “To our knowledge, there is not yet a strong causal link between the micronutrient component of the ionome and factors of vine growth or development that might influence traits like wine quality. However, it is noted that macronutrient deficiencies can have negative effects on such traits (Bravdo 2000; Brunetto et al. 2015) and can be mediated by rootstock (Gautier et al. 2018). This suggests a strong understanding of the rootstock influence on the vine’s ionome is warranted, and more work needs to be done to establish these relationships”

474: Can stomatal conductance be limited by flow in the roots? Do you think it's more likely such an indirect effect, or a direct effect such as signals from the rootstock actually change the rates by stomatal closure, etc?
-Response: This is a really good question that is particularly challenging to fully address. We would wager that root architecture is a key driver of physiological variation, and we tested this in a greenhouse study the results of which are in preparation now. However, it is worth noting signals could be passed from the rootstock, but the space of signal passing through graft junctions is complex and this work was not designed to address anything to that end.

501: I think here, rather than 'phenotype' as 'traits' you meant to say something about the 'data types,' which you referred to as modalities before and would be appropriate to use here. 'traits of different modalities'. I would suggest this instead of 'data types' perhaps for consistency. Definitely it would be a benefit to the field to have ways to describe these 'groups' of traits from the same instrument, my lab also runs into this with fused, multi-dimensional data.
-Response: Great observation, and we agree. We have fixed this instance of improper usage and we have clarified the language in the discussion (and the rest of the manuscript) to be more consistent. Specific to this comment, ‘data type’ has been replaced with modality/ies throughout the piece. As above, we recognize that we were being imprecise with language, so we have fixed this and other terms used interchangeably (trait/phenotype, phenotypic/phenomic, and data type/modality) throughout the manuscript.

502: latent phenotypes were mentioned in the definition of phenomics (where I only see them as one possibility, not a defining feature). Some definition would be useful.
-Response: This is a good catch. We did not mean to imply latent phenotypes were the only possible outcome of this work. This sentence was expanded a bit to include that idea that latent structure is one possibility, but using this to target integrative analysis is also a strong possibility. (L565)

510: back to phenomic correlation - what's the difference with phenotypic correlation?
-Response: Good catch of this persistent issue in the earlier version of the manuscript. We did not mean to imply there was a difference, or to make any kind of statement on this distinction. As above, we recognize that we were being imprecise with language, so we have fixed this and other terms used interchangeably (trait/phenotype, phenotypic/phenomic, and data type/modality) throughout the manuscript.

533: Very little information was provided about how the changes measured here in traits would affect yield or other consumer-facing traits. Not only that, but why is the multi-dimensionality important? Does it reveal trade-offs in traits, for example? I'm trying to help you improve the biological impact component. Some arm waving may be warranted.
-Response: We very much appreciate the direction the reviewer is going here, and we have attempted to address this in the potential implications section of the manuscript and elsewhere. Multi-dimensional data are data that consist of many different observations (for example, the ionome which includes measurements of 20 different ions). Multi-dimensional data offer more robust, approaching comprehensive observations of plant phenotypes. They offer a rich source of information that can be used to more comprehensively understand the basic biology of the organism - for example, how root systems influence features of shoot systems in grafted plants. This is described in, for example, L94-100 of the introduction. The influence of the

Powered by Editorial Manager® and ProduXion Manager® from Aries Systems Corporation

| | |
|---|---|
| | phenotypes we measured on yield or other consumer facing traits are under active investigation. For example, ongoing work by others members of our project team describes berry chemistry and wine volatiles for the experimental vineyard described here. The volume of data was so large; this manuscript represents the first step in processing and interpreting multiple multi-dimensional phenotypes and trying to understand what approaches can be used to understand how they relate to one another. The next steps will be to connect these data with observations that might be more directly relevant to viticulturists. Our hope is that this manuscript will provide the foundation for those analyses that integrate multi-dimensional data from different organ systems, such as leaves and berries.<br><br>457: This note is very thorough and appreciated, however a github link is not permanent and therefore I suggest to include as supplemental to this paper or else place on a 'permanent' public repository such data dryad, Zenodo, etc. If the irrigation factor was ignored, you should say so.<br>-Response: Good point. The note on irrigation has been added as a Supplemental Note to this manuscript. Irrigation was treated as an additional blocking factor in the analyses done here. While we will keep the other data available on Figshare, we are exploring other homes for the data that are in line with GigaScience's preferences.<br><br>561: After reading this section, I wasn't sure about the experimental design, especially what type of randomization was used. I would guess that an appropriate design here would have been split plot block design taking into account irrigation (which I guess you are saying you ignored in the end). Were genotype randomized? the groups of 4 are mentioned, should that be taken as the experimental unit? I'm not super picky about stats, but some might say there are flaws here, and perhaps the 72 should be divided by 4 as as far as complete replicates? In Supp Fig 1 in the map, I see up to Block F - so should it be 6 true replicates? In cases likes this, I usually think of the additional plants as subreplicates. Your design seems basically just like a annual crop field trial with small plots with multiple plants. We usually measure a trait on those subreps then average it to the plot level for further analysis. In that case, the subreplication isn't used in stats directly, but does allow a better approximation of the value for each plot and decrease overall 'random' or 'environmental' error.<br>-Response: This is a great catch by the reviewer. We regret that the earlier version of this manuscript did not fully explain the experimental design of the research vineyard used in this study. These details have been filled in in section Study Design of the manuscript. Further, I think some additional confusion may stem from us using "replicate" as a vague stand-in for both clonal replicates and statistical replicates. To address this, we have amended the language about the four rootstock scion combinations as follows on L617: "Clonal replicates of each of the four rootstock-scion combinations were planted 72 times for a total of 288 vines planted in nine rows". In addition, we included the specific type of design (split-plot) to this section. Finally, we addressed the number of true replicates in a comment by reviewer 2 concerning RNAseq. The same logic can be used to derive the total number of biological replicates for leaf shape and ionomics at the highest order interactions (4) and for all other phenotypes (2). In the case where the number of biological replicates is two, the estimation and interpretation of effects is minimized due to lack of power. |
| **Additional Information:** | |
| **Question** | **Response** |
| Are you submitting this manuscript to a special series or article collection? | No |
| **Experimental design and statistics**<br><br>Full details of the experimental design and statistical methods used should be given in the Methods section, as detailed in our Minimum Standards Reporting Checklist. Information essential to interpreting the | Yes |

| | |
|---|---|
| data presented should be made available in the figure legends.<br><br>Have you included all the information requested in your manuscript? | |
| **Resources**<br><br>A description of all resources used, including antibodies, cell lines, animals and software tools, with enough information to allow them to be uniquely identified, should be included in the Methods section. Authors are strongly encouraged to cite Research Resource Identifiers (RRIDs) for antibodies, model organisms and tools, where possible.<br><br>Have you included the information requested as detailed in our Minimum Standards Reporting Checklist? | Yes |
| **Availability of data and materials**<br><br>All datasets and code on which the conclusions of the paper rely must be either included in your submission or deposited in publicly available repositories (where available and ethically appropriate), referencing such data using a unique identifier in the references and in the “Availability of Data and Materials” section of your manuscript.<br><br>Have you have met the above requirement as detailed in our Minimum Standards Reporting Checklist? | Yes |

Manuscript

Click here to access/download;Manuscript;MV2017Leaves_GigaScience_rev1

Formatted: Header

# Multi-dimensional leaf phenotypes reflect root system genotype in grafted grapevine over the growing season

Zachary N. Harris[1,2*] (zachary.n.harris@slu.edu), Mani Awale[3] (maybd@mail.missouri.edu), Niyati Bhakta[1,2] (niyatisbhakta@gmail.com), Daniel H. Chitwood[4,5] (chitwoo9@msu.edu), Anne Fennell[6] (anne.fennell@sdstate.edu), Emma Frawley[1,2] (emma.frawley@wustl.edu), Laura L. Klein[1,2] (laura@leafworks.com), Laszlo G. Kovacs[7] (laszlokovacs@missouristate.edu), Misha Kwasniewski[3] (mtk5407@psu.edu), Jason P. Londo[8] (jason.londo@usda.gov), Qin Ma[9] (qin.ma@osumc.edu), Zoë Migicovsky[10] (zoe.migicovsky@dal.ca), Joel F. Swift[1,2] (joel.swift@slu.edu), and Allison J. Miller[1,2*] (allison.j.miller@slu.edu)

[1]Department of Biology, Saint Louis University, 3507 Laclede Avenue, St. Louis, MO, 63103-2010, USA
[2]Donald Danforth Plant Science Center, 975 North Warson Road, St. Louis, MO, 63132-2918, USA
[3]Division of Plant Sciences, University of Missouri, 135 Eckles Hall, Columbia, MO, 65211, USA
[4]Department of Horticulture, Michigan State University, East Lansing, MI, 48824, USA
[5]Department of Computational Mathematics, Science and Engineering, Michigan State University, East Lansing, MI, 48824, USA
[6]Department of Agronomy, Horticulture & Plant Science, South Dakota State University, Brookings, SD, 57006, USA
[7]Department of Biology, Missouri State University, 901S. National Avenue, Springfield, MO, 65897, USA
[8]United States Department of Agriculture, Agricultural Research Service: Grape Genetics Research Unit, 630 West North Street, Geneva, NY, 14456-1371, USA
[9]Department of Biomedical Informatics, The Ohio State University, 1585 Neil Ave, Columbus, OH, 43210
[10]Department of Plant, Food, and Environmental Sciences, Faculty of Agriculture, Dalhousie University, Truro, NS, B2N 5E3, Canada
* To whom correspondence should be addressed

**Formatted:** Header


**Abstract**

Background: Modern biological approaches generate volumes of multi-dimensional data, offering unprecedented opportunities to address ~~fundamental~~ biological questions previously beyond reach due to small or subtle effects. A fundamental question in plant biology is the extent to which below-ground activity in the root system influences above-ground ~~traits (~~phenotypes~~)~~ expressed in the shoot system. Grafting, an ancient ~~agricultural~~horticultural practice that fuses the root system of one individual (the rootstock) with the shoot system of a second, genetically distinct individual (the scion), is a powerful experimental system to understand below-ground effects on above-ground phenotypes. Previous studies on grafted grapevines have detected rootstock influence on scion phenotypes including physiology and berry chemistry~~; however~~. However, the extent of the rootstock's influence on leaves, the photosynthetic engines of the vine, and how those effects ~~changes~~change over the course of a growing season, are still largely unknown.

Results: Here, we investigate associations between rootstock genotype and shoot system phenotypes using five multi-dimensional leaf phenotyping modalities measured in a common grafted scion: ionomics, metabolomics, transcriptomics, morphometrics, and physiology. Rootstock influence is ubiquitous but subtle across modalities with the strongest signature of rootstock observed in the leaf ionome. Moreover, we find that the extent of rootstock influence on scion phenotypes and patterns of ~~phenotypic~~phenomic covariation are highly dynamic across the season.

Conclusions: These findings substantially expand previously identified patterns to ~~suggest~~demonstrate that rootstock influence on scion phenotypes is complex and dynamic and underscore that broad understanding necessitates volumes of multi-dimensional data previously unmet.


**Background**

High-throughput data acquisition has afforded unprecedented capacity to quantify and understand plant form and function. Recent advances in imaging and computation have expanded our ability to

**Formatted:** Header

measure plant ~~structures [1,2]~~traits or phenotypes [1,2], and to extend those comprehensive measurements into latent space phenotypes ~~[3].~~[3]. Now broadly known as phenomics, this burgeoning field is characterized as the acquisition and analysis of high-dimensional phenotypic data at different hierarchical levels ~~[4,5]~~[4,5], often with an eye toward multiscale data integration. A holistic and hierarchical approach to plant phenotypic variation affords unique insights into plant evolution~~,~~ and how plants change over development and in response to environmental cues and horticultural manipulation.

A fundamental question in plant biology is how root systems influence ~~phenotypic~~phenomic variation in above-ground shoot systems including leaves, flowers, and fruits. Grafting, a common horticultural manipulation that joins the shoot system of one individual (the scion) with the root system of another individual (the rootstock), is commonly used in crop species to confer favorable phenotypes to commercial scions ~~[6]~~[6], including enhanced disease resistance ~~[7,8]~~[7,8], fruit quality, plant form ~~[9]~~[9], response to water stress ~~[10]~~[10], and growth on particular soils ~~[11,12]~~[11,12]. Because grafting often uses clonally propagated materials, it is possible to manipulate and replicate different combinations of root systems and shoot systems, offering a valuable experimental system in which root system impacts on shoot system phenotypes can be evaluated.

The European grapevine (*Vitis vinifera*) is among the most economically important grafted crops in the world. Grapevines are cultivated primarily for fruits used to make wine and juice, as well as for table grape and raisin production. Grafting in grapevines became widespread in the mid-1800's following the accidental introduction of the root-feeding aphid phylloxera from its native North America into Europe, where it began attacking the roots of European grapevines ~~[13].~~[13]. Because European grapevines often ~~did~~do not survive phylloxera infestation, in regions where phylloxera has been introduced most grapevine cultivation~~ now~~ consists of European grapevines grafted to rootstocks derived from phylloxera-resistant North American *Vitis* species including *V. berlandieri, V. riparia,* and *V, rupestris*, and their hybrid derivatives. In addition to grapevines, more than 70 major perennial crops are grafted including many fruit trees and vines ~~[9]. In these crops, grafting~~[9]. Grafting decouples the breeding of shoot systems and root systems, with selection in plants targeted for use as scions focusing

Formatted: Header

primarily on fruit ~~traits~~phenotypes, and selection in plants targeted for use as rootstocks focused on below-ground biotic and abiotic stress resistance, as well as their impacts on shoot system phenotypes.

The effects of grafting in grapevine show a remarkable breadth of scion response patterns. For example, a study of *Vitis vinifera* cv. 'Cabernet Sauvignon' grafted to different rootstocks identified transcriptome reprogramming in the scion of grafted plants; this appeared to be a general effect of grafting to a rootstock and was not rootstock-specific ~~[14].~~[14]. In contrast, other studies have found signatures of rootstock genotype in the transcriptome in early berry development, although this distinction was lost in later development ~~[15,16], but see [17].~~[15,16], but see [17]. Comprehensive phenomic analyses, including those that link transcriptome data with other high-throughput ~~phenotypic~~phenotyping assays, offer an opportunity to expand understanding of rootstock effects on grapevine shoots. In one study, leaves of the *V. vinifera* cultivar 'Gaglioppo' showed variation in stilbene and abscisic acid concentrations due to rootstock genotype, as well as differences in transcriptional profiles ~~[18].~~[18]. Likewise, gene expression, ion concentrations, and leaf shape in the cultivar 'Chambourcin' varied in response to rootstock genotype ~~[18,19].~~[18,19]. Collectively, these studies suggest the impacts of grafting are diverse and may vary over the course of vine development. However, to date few studies have surveyed multiple high-dimensional scion phenotypes to understand ~~the~~ rootstock influence on shoot system ~~traits~~phenotypes over the course of the growing season or the extent to which grafting effects on the scion covary with one another.

~~Grapevine leaves~~ Leaves are the photosynthetic engine of the organism and a primary site for perception and response to environmental change. ~~Leaves present a wide variety of highly variable and readily assayable phenotypes, providing an important opportunity for phenomic assessment.~~ Grapevine leaves have been used for centuries as markers of species and cultivar delimitation, developmental variation, disease presence, and nutrient deficiency ~~[20,21].~~[20,21]. More recently, analysis of grapevine leaf morphology has identified genetic architecture of leaf shapes ~~[22]~~[22], developmental patterns across the season ~~[23]~~[23], and signatures of evolution in the grapevine genus ~~[24].~~[24]. Grapevine leaves respond to stress through gas and water exchange with the atmosphere

Formatted: Header

[25,26][25,26] and have been shown to differentially partition the ionome depending on their position on the shoot [19][19] and their rootstock genotype [19,27,28][19,27,28]. The volume of work on grapevine leaves provides a foundation for the analysis of phenomic variation in a vineyard over a season in response to grafting.

In this study, we investigate effects of grafting on high dimensional leaf phenotypes of the hybrid cultivar ‘Chambourcin’ over the course of the growing season. We quantify leaf elemental (ion) concentrations, metabolite abundance, gene expression, shape, and vine physiology in a replicated rootstock trial where the hybrid grapevine cultivar ‘Chambourcin’ is growing ungrafted and grafted to three different rootstocks. The four root-shoot combinations (‘Chambourcin’ ungrafted, ‘Chambourcin’ grafted to three different rootstocks) are replicated 72 times in a randomized block experimental design with an irrigation treatment (Supplemental Figure 1). DataPhenotypic data, data that describe variation for a particular trait within a particular modality, were collected either on the full 288-vine set (ion concentrations, leaf shape) or on a subset of 72 vines (the 72-vine set; metabolite abundance, gene expression, vine physiology). Using data collected at three time points that span the growing season (anthesis, veraison, and harvest), we show that all phenotyping modalities (ionomic, metabolomic, transcriptomic, morphometric, and physiology phenotypes) reflect subtle but ubiquitous responses to grafting and rootstock genotype. Rootstock effects on shoot system phenotypes were often dynamic across the season, suggesting that accounting for seasonal variation could alterenhance our understanding of grafting effects in viticulture.

**Data Description**

*Leaf Ionomics*

The ionome describes elemental composition of a tissue at a particular time point [29].[29]. Three leaves per vine were collected from the 288-vine set. at three seasonal time points: anthesis (~mid May), veraison (~late July), and harvest ~mid September). Leaves were sampled from a single shoot and

**Formatted:** Header

included the youngest fully opened leaf at the shoot tip, the approximate middle leaf, and the oldest leaf at the shoot base. Teams were deployed in the vineyard so that multiple vineyard rows were being sampled concurrently. As such, ‘block’ represented unmeasured spatial variation, but did not strictly correlate with time of sampling due to the nature of sampling (see Methods). Whole leaves were placed in zip-lock bags in the field and stored in a cooler on ice packs, scanned for leaf shape analysis in the lab (see Leaf Shape) and then dried in coin envelopes at 50°C for one to three days for elemental analysis. Between 20 and 100 mg of leaf tissue was acid digested and 20 ions were quantified using inductively coupled plasma mass spectrometry (ICP-MS) following standard protocol ~~[30,31] at~~of the Donald Danforth Plant Science Center (DDPSC~~).~~) Ionomics Pipeline [30,31]. Ion quantifications were corrected for internal standard concentrations, instrument drift and by initial sample mass~~ as part of the DDPSC Ionomics Pipeline.~~. The output of the Pipeline contained estimated concentrations of each of the following 20 elements: Al, As, B, Ca, Cd, Co, Cu, Fe, K, Mg, Mn, Mo, Na, Ni, P, Rb, S, Se, Sr, and Zn. For each ion concentration, we computed z-score distributions and used those values as the basis for linear models. ~~Non~~Following convention, non-standardized values were used for machine learning analysis.

*Leaf Metabolomics*

The metabolome comprises small mostly organic molecules present in a tissue~~, representing~~ and represents a catalogue of the products of metabolic processes ~~[32,33].~~[32,33]. Metabolomic analysis was completed at veraison ~~and~~(the onset of fruit ripening) and immediately prior to harvest for the 72-vine set. For each vine, three mature leaves were sampled from the middle of a single shoot and immediately flash frozen in liquid nitrogen in the field to capture the metabolic state of the leaves when attached to the vine. Leaves were sampled by a single team near midday in row and block order~~.~~, ensuring that ‘block’ captured both unmeasured spatial variation and temporal variation over the sampling window (see Methods). Frozen leaves were transported to the University of Missouri Enology ~~lab~~Lab on dry ice and stored at -80˚C. Following the protocol of ~~[34]~~[34], whole leaves were manually ground in liquid nitrogen

with a mortar and pestle, 0.5g of powder was weighed into a centrifuge tube, 1.5ml of 1:1 MeOH: ACN was added. Samples were vortexed to suspend leaf particles and sonicated for 20 minutes in an ice bath. ~~Following~~After extraction, samples were centrifuged for 10 minutes at 3,000 g and filtered with a 0.22 PTFE syringe filter into a 1.5ml sample vial before injecting into a Waters XEVOTM QToF LCMS system (Waters Corporation, Milford, MA, USA). Chromatographic separation was achieved using a Waters Acquity TM Ultra Performance LC H-Class system (Waters Corporation, Milford, MA, USA) equipped with Waters Acquity BEH C18 column (2.1mmx150mm and 1.7um particle size) and a diode array detector. Samples were injected in random order across the sampling periods. The injection volume was set at 2.5ul and the flow rate was set at 0.4 ml/min. The mobile phase consisted of 0.1% formic acid in water (solvent A) and 0.1% formic acid and 5% water in acetaldehyde (solvent B) and the gradient was as follows: 100% A for 0.5 min; 0.5-18min increased to 99% B; 18-19 min. held at 99% B; mobile phase was re-equilibrated for 2 min between runs. Diode array was monitored at 225-500nm. Mass spectrometry was performed on a XevoTM QTof (Waters Corporation, Milford, MA, USA). The electrospray ionization (ESI) was operated in both positive or negative ionization modes in separate runs. The scan range was set as m/z 50-1500 with 0.2 sec accumulation time. MS settings were as follows: capillary voltage was 2.5kV; cone voltage ramped from 20-40V; collision energy was set to 6V; detector voltage was set to 1950V; desolvation gas was set to 1000 L/hour; cone gas was set to 50 L/hr; source temperature was 120℃ and desolvation temperature was set at 550℃.

LC-MS instrument files were converted to .cdf format and uploaded to XCMS online ~~[35]~~[35] for chromatogram normalization and feature detection via "single job" parameters. ~~Identified~~The 661 identified metabolomic features were used as the basis of a principal components (PC) analysis. The top 20 PCs were treated as distinct phenotypes to model according to the experimental design. In PCs that varied significantly by rootstock, features that loaded more than 1.96 standard deviations above or below the mean were fit independently with the same model design.

**Formatted:** Header

Formatted: Header

*Leaf Gene Expression*

The youngest fully-opened leaves on two shoots were collected from each plant of the 72-vine set (see Study Design). The two leaves, which were distinct from leaves used for ionomics, leaf shape, metabolomics and physiology data collection, were pooled for RNA sequencing. Leaves were sampled by a single team near midday between 10AM and 2PM in row order ensuring that 'block' and 'row' accounted for unmeasured spatial variation and temporal variation over the sampling window (see Methods). Samples were sequenced using 3'-RNAseq, a method ideal for organisms with reasonably characterized reference genomes ~~[36].~~[36]. Total RNA was extracted from plant tissues using the Sigma Spectrum Plant Total RNA kit with modification of the addition of 2% PVP40 to the extraction buffer to decrease phenolic inhibitors. All RNA extractions were checked for quality control using a Nanodrop. Sequencing was conducted using the Illumina NextSeq500 platform which returned single-end 86 bp reads. The first 12 nucleotides from each read were trimmed to remove low-quality sequences using Trimmomatic (options: HEADCROP:12; ~~[37]).~~[37]). Low quality trimmed reads were additionally identified based on overrepresentation of kmers and removed using BBduk (April 2019 release) ~~[38].~~[38]. Trimmed and QC-controlled reads were mapped to the 12Xv2 reference *Vitis vinifera* genome ~~[39,40]~~[39,40] using STAR (v2.7.2b) ~~[41]~~[41] with default alignment parameters. RNAseq read alignments were quantified using HTSeq-count (v0.11.2) ~~[42]~~[42] and a modified version of the VCost.v3 reference *V. vinifera* genome annotation ~~[40].~~[40]. To capture mis-annotated gene body boundaries in the genome, all gene boundaries in the annotation were extended 500 bp.

Variation in gene expression was assessed using two methodologies. First, we identified individual genes which responded to specific factors in the experimental design using DESeq2 (v1.24.0) ~~[43].~~[43]. Each gene was fit with the model "~ Block + Irrigation + Phenology_Rootstock" where the 'Phenology_Rootstock' model term was used to understand the potential interaction of phenology and rootstock. ~~Differentially expressed genes were identified for each pairwise contrast in the model.~~ Genes were filtered to a gene set that included only genes with a normalized count greater than or equal to two in at least five samples. To check the validity of our expression results, we assayed two classes of

Formatted: Header

housekeeping gene (Ubiquitin-domain and actin-family) and eight previously annotated circadian genes [44] (Supplemental Figure 2). Differentially expressed genes were identified for each pairwise contrast in the model. Second, we used principal component analysis (PCA) to collapse variation in co-expressed genes into fewer dimensions. Normalized count-filtered genes from DESeq2 were transformed using the variance stabilizing transformation (VST; [44][45]) and input into a PCA. We then analyzed the top 100 PCs in the context of the broader experimental design. We previously showed that the transcriptome varied by the time of collection and was potentially interacting with the rootstock effect [19].[19]. Moreover, the other modalities in this study point to weak if any effects from the irrigation treatment. (see Supplemental Note 1). Due to the nature of the vineyard design, we could not identify both irrigation and time effects (marked by row) in a single model (irrigation and row are collinear; see Study Design). To approximate the impact from time of collection (row) in the vineyard on gene expression, linear models were first fit to remove variation imparted by irrigation from each of the top 100 PCs. The residuals were then used as the basis for linear models and machine learning analysis.

*Leaf Shape*

All leaves from a single shoot directly emerging from a trained cordon were collected from each vine in the 288 vine set at 80% anthesis and veraison. At harvest, we collected only the oldest (first emerging leaf), middle (estimated from the middle of a whole shoot), and youngest (smallest fully emerged leaf at the shoot tip, >1cm). Leaves were collected approximately in row order (from south to north) and stored in a cooler. Each leaf was imaged using an Epson DS-50000 scanner. in color against a white background at 1200 DPI and written as JPEG formatted images. Following scanning of leaves for leaf shape analysis, the oldest, middle, and youngest leaves were dried and used to estimate leaf elemental composition (see Ionomics). As the leaf shape samples and ionomics samples were identical, ‘block’ represented unmeasured spatial variation, but did not strictly correlate with time of sampling (see Methods). While all leaves were collected from a single shoot, only the oldest, middle, and youngest leaves were used in this analysis.

We assessed leaf shape using ~~generalized procrustes analysis~~Generalized Procrustes Analysis (GPA) of landmarks. For the three leaves per vine used in leaf shape analysis, 17 homologous landmark features were identified ~~[22].~~[22]. The GPA-rotated coordinate space was used for all subsequent statistical analysis including PCA in order to summarize variation in leaf shape ~~[45].~~[46]. From the PCA, we extracted the top 20 PCs and fit linear models and machine learning models to describe variation.

*Vine physiology*

Intracellular $CO_2$ concentration, stomatal conductance and leaf transpiration rate were measured at midday (each measured simultaneously between 10am to 1pm) on one fully expanded sun-exposed leaf for each of the vines in the 72-vine set. Physiology measurements were taken in row order ensuring that 'block' correlated with temporal variation over the sampling window. Measurements were taken using an LI-6400XT Portable Photosynthesis system coupled with a pulse amplitude-modulated (PAM) leaf chamber fluorometer (Li-Cor, Inc., Lincoln, NE, USA) with the following parameters: incident photosynthetic photo flux density level of 1000 μmol m−2 s−1 generated by a red LED array and 10% blue light to maximize stomatal opening, $CO_2$ mixer of 400 ~~umol~~μmol/s, fixed flow of 300 ~~umol~~μmol/s, and ambient leaf and block temperature. Soil moisture was measured for each plant in the 72-vine set using a fieldScout TDR 300 Moisture meter equipped with 20 cm rods (Spectrum Technologies, Inc. Aurora, IL, USA). Midday stem water potential was measured using a pressure bomb/chamber (PMS Instrument Co., Albany, OR, USA) after enclosing the leaves in an aluminum foil bag for at least 15 minutes to equilibrate the water potential of the xylem in the stem to that attached leaf~~.~~ (for a discussion on equilibration time, see [47,48]).

**Analyses**

**Formatted:** Header

**Formatted:** Font: Times New Roman

*Leaf ionome*

To characterize the leaf ionome over the growing season, we sampled the youngest, middle, and oldest leaf from a single shoot from each of the vines within the 288-vine set at three phenological stages ~~(Figure 1).~~and measured the concentrations of 20 ions in each leaf individually. Bivariate correlations showed that ion concentrations are not independent of each other, but that the strength and direction of relationships between ions vary with respect to phenological stage and leaf position (Supplemental Figure ~~2~~3). As such, we fit independent linear models to each ion. Leaf position, phenological stage, or the interaction of phenological stage and leaf position explained the highest amount of variation for most ions (Figure 1A-B). Many ions significant for the interaction showed a clear signal of leaf position at anthesis and veraison, and either no explainable variation or muted variation at harvest. For example, calcium (Figure 1B) varied with leaf position (22.7~~%;~~% variation explained; p < 1e-05), phenology (24.0%; p < 1e-05), and their interaction (7.4%, p < 1e-05). All possible pairwise combinations of leaf position were significantly different at anthesis, and both the youngest and middle leaves were different from the oldest leaves at veraison and harvest. In the case of potassium (Figure 1B), significant variation was explained by leaf position (16.1%; p < 1e-05), phenology (19.6%; p < 1e-05), and their interaction (10.6%; p < 1e-05). However, post-hoc comparisons of phenology-wise mean calcium concentrations showed that differences were present only at anthesis and veraison.

~~The rootstock~~Rootstock genotype showed remarkable influence on the composition of the leaf ionome. All ions except aluminum, sodium, and zinc were significant for rootstock as a single fixed effect (Figure 1A). Rootstock explained between 0.4% (rubidium; p = 3.2e-05) and 14.3% (nickel; p < 1e-05) of variation ~~in each~~ ion concentrations (Figure 1A). ~~Ions that responded weakly to the interaction of leaf position and phenology tended to show~~For some ion concentrations (such as cobalt and nickel), significant variation was explained by the interaction of rootstock and phenology; this pattern was observed mostly in ions that responded weakly to the interaction of leaf position and phenology. These ions showed similar patterns to the leaf position by phenology interaction where clear signal was exhibited at anthesis and veraison then is either absent or muted at harvest. For example, cobalt was most

**Formatted:** Header

Formatted: Header

abundant in ‘1103P’-grafted vines at anthesis (Figure 1C). At veraison, both ‘1103P’-grafted and ‘SO4’-grafted had elevated concentrations compared to Ungrafted and ‘3309’-grafted vines. However, by harvest, cobalt concentration variation was muted and only ‘SO4’-grafted vines showed evidence of elevated concentration. Similarly, nickel showed significant variation partitioned into the rootstock by phenology effect (Figure 1C). Both anthesis and veraison show reduced nickel concentration in ‘1103P’-grafted vines and elevated concentrations in ‘SO4’-grafted vines. However, at harvest, no comparisons are significant.

Machine learning on ion concentrations confirms that the leaf ionome contains a signature from the rootstock genotype and the interactions of rootstock genotype with phenology and leaf position. A random forest model trained to predict rootstock showed an overall accuracy of 75.2% (Figure 1D). Ions important for this classification were nickel (~~MDA=~~Mean Decrease in Accuracy (MDA)=0.089), molybdenum (MDA=0.058), and magnesium (MDA=0.054), corroborating the rootstock term’s significance in the linear models. Notably, when we trained a model to simultaneously predict rootstock and phenological stage, rootstock prediction accuracy increased appreciably (Figure 1E). For example, the ability of the model to detect ungrafted vines (the balanced accuracy of ungrafted predictions) improved from 81.7% accuracy overall to 91.1% accuracy at anthesis and 85.9% at harvest. Generally, performance at veraison matched the rootstock-only model performance. The ions most important for this joint (rootstock/phenological stage) prediction were nickel (MDA=0.167), phosphorus (MDA=0.110), and strontium (MDA=0.065). The rootstock by phenology model term was significant in the linear models for these ions, but was not a largest descriptor of variation. The joint prediction of rootstock and leaf position performed substantially better than chance ($p < 1e\text{-}05$), but accounting for leaf position did not improve rootstock prediction as was the case in the joint prediction of rootstock and phenology (Figure 1F). Ions important for this classification were sulfur (MDA = 0.051), rubidium (MDA = 0.051), and nickel (MDA = 0.049).

Formatted: Header

*Leaf metabolomics*

We performed untargeted metabolomics on leaves from the 72-vine set at veraison and harvest, quantifying the concentrations of 661 metabolites (Figure 2). The top 20 PCs accounted for a total of 67.3% of the total metabolomic variation, with the top three capturing 23.1%, 9.2%, and 6.2%, respectively. Individual PCs after the top 20 explained less than 0.82% of the metabolome. Linear models for each of the top 20 PCs found that the strongest drivers of variation in leaf metabolomics were phenology and temporal blocking factor. For example, 90.6% of variation on PC1 was due to phenology ($p < 1e-05$; Figure 2A). PC2 primarily reflected the interaction of phenology and temporal block (26.4%, $p < 1e-05$) and temporal block as a main effect (18.9%, $p < 1e-05$). The patterns of variation attributable to PC2 were similar in PCs 3-10 (Figure 2A).

PC17 was controlled by rootstock as a main effect (18.5%, $p < 1e-03$; Figure 2B). On PC17, ungrafted vines were significantly different from vines grafted to ‘3309C’ ($p = 0.02$) and ‘SO4’ ($p < 1e-05$). Vines grafted to ‘1103P’ were also significantly different from vines grafted to ‘SO4’ ($p = 0.009$). Metabolites that loaded more than 1.96 sd from the mean loading on PC17 were extracted and independently fit to additional linear models. We identified four metabolite features (M374T1 [rt = 1.33, m/z = 374.1146], M117T1 [rt = 0.61, m/z = 117.0583], M175T1_1 [rt = 0.87, m/z = 175.1269], and M333T1_3 [rt = 0.71; m/z = 333.1582]) which were influenced by rootstock as a main effect and the metabolite (M112T1 [rt = 1.48, m/z = 112.0061]) which was influenced by the interaction of rootstock genotype and phenological stage. At this time, the identification of these features remains unknown.

Linear discriminant analysis confirmed that many experimental factors likely influence the metabolome. For example, when trained to maximize variation between classes of rootstocks, the model identified a space that weakly separates ‘1103P’-grafted and ‘SO4’-grafted vines from ~~Ungrafted~~ungrafted and ‘3309C’-grafted vines (LD1) and separates ‘3309C’-grafted vines from other classes (on LD2) (Figure 2C). Despite this, machine learning showed minimal predictability for any class other than phenology, which was predictable with an accuracy of 100% for withheld samples. Rootstock

Formatted: Header

genotype based on the metabolome was not predictable with accuracy only marginally better than chance (34.6%).

*Gene Expression*

We performed 3’-RNAseq on the youngest fully-opened leaves of the 72~~-~~-vine set at three time points (Figure 3). ~~We~~On average, each sample contained 4.1 million 3’-reads and measured the expression of 17,852 genes. Overall, we identified variation in 23,460 genes that had a DESeq2-normalized count greater than two in at least five samples. We computed the expression of two classes of housekeeping genes, and showed that they are generally stable across samples over phenological time (Supplemental Figure 2). We noted that some variation is expected for housekeeping genes; see, for example, [49]. Moreover, we showed that patterns of previously annotated circadian genes conform to expected results over the sampling window. For example, predicted orthologs of *LHY* and *RVE1* are correlated and decreasing over our sampling window, and a predicted *TOC1* ortholog is invariant. The results of these analyses provide general confidence in the gene expression data presented here.

Using a traditional differential expression analysis framework~~,~~ based on established DGE software (Deseq2), all genes returned as significantly differentially expressed by rootstock appeared to be false positives, evidenced by a single extreme outlier altering group means. Hierarchical clustering of the 500 most variable genes after variance stabilizing transformation (VST) showed strong latent structure in the transcriptome and that most variation in the transcriptome was explained by phenological stage (Figure 3A). The top 100 PCs on the VST-transformed gene counts accounted for nearly 92.3% of variation in the transcriptome. Linear models on each of the top 100 PCs indicated that 82.4% and 61.4% of the variation on PC1 and PC2 respectively were attributable to the phenological stage (Figure 3B-C). Row was also a significant descriptor of variation as a single, fixed effect and in interactions with rootstock and phenological stage. For example, row accounted for 36.0% and 43.3% of the variation on PC4 and PC6, respectively. Interacting with phenological stage, row accounted for >10% of variation on 17 additional PCs.

Formatted: Header

Patterns of gene expression identified through LDA corresponded to phenological stage, vine row, and rootstock. LDA separated phenological stages into three distinct, non-overlapping groups in the space spanning LD1 and LD2 (Supplemental Figure 34). When trying to separate rows into distinct classes, the model converged on a 'horseshoe' shape in the LD1- LD2 space (Figure 3D).), suggesting either a circadian topology to the transcriptome or continuous spatial variation over the vineyard [50]. LD1 maximized the variation between row 8 (sampled early in the day) and row 16 (sampled a few hours later). LD2 maximized the separation of both rows 8 and 16 with row 12 (the row sampled in the middle of the sampling window). A model trained to separate rootstock classes (Figure 3E) showed that LD1 separated the rootstock 1103P from other rootstock genotypes, and LD2 primarily separated the rootstock '3309C' from ungrafted vines (Supplemental Figure 34).

Formal machine learning on gene expression PCs largely supported the linear models. A random forest trained to predict phenological stage classified testing samples with 92.9% accuracy. Anthesis was the most predictable class with a balanced accuracy of 100%; veraison and harvest displayed balanced accuracies of 92.7% and 92.4%, respectively. The PCs most important in phenology prediction were PC1 (MDA = 0.16) and PC2 (MDA = 0.12). Gene expression PCs were unable to predict rootstock, with a total prediction accuracy of 23.4%. While no features were especially important in the prediction processes, PC44 showed the largest mean decrease in Gini impurity corroborating its signal in the linear models.

*Leaf shape*

We collected leaves from the 288-vine set at three time points and landmarked a total of 2,422 leaves (Figure 4). Homologous leaf landmarks were used for generalized procrustes analysisGeneralized Procrustes Analysis (GPA). PCA on the GPA-rotated coordinates revealed ~97.2% of the total shape variation was captured by the top 20 principal components with PC1, PC2, and PC3 explaining 24.1%, 19.0%, and 13.3% of the variation respectively. Lower values on PC1 primarily capture leaves with shallow petiolar sinuses and short midvein distance from the depth of the superior sinus to the top of the

**Formatted:** Header

midvein, whereas higher values on PC1 capture the opposite (Figure 4A). Similarly, lower values on PC2 capture deep petiolar sinuses combined with very shallow superior sinuses, and vice versa for higher values. PC3 primarily captures asymmetry (Figure 4A).

In total, ~~only~~ 5.76% of variation on PC1 was explained by the experimental design~~, with most~~. Of this, variation in leaf shape was explained by phenology (2.63%; padj < 1e-05), then rootstock (0.95%; padj < 0.001), leaf position (2.61%; padj = 0.03), and the interaction of phenology and leaf position (0.62%; padj = 0.009) (Supplemental Figure ~~4A~~5A). Post-hoc mean comparisons on PC1 showed that shapes of leaves from ungrafted vines were significantly different from leaves of vines grafted to 1103P (p < 0.001), 3309C (p < 0.001) and SO4 (p < 0.001) (Supplemental Figure ~~4B~~5B). Moreover, PC1 captured subtle variation in the leaf position by phenological stage interaction where middle leaves showed significant differences between anthesis and veraison (p < 1e-03), and the oldest leaves showed significant differences when comparing anthesis to veraison (p < 1e-05) and anthesis to harvest (p < 1e-03).

For PC2, 61.4% of variation could be assigned to an experimental factor. This included significant variation from leaf position (46.9%, padj < 1e-05), phenology (1.4%; padj < 1e-05), and the interaction of leaf position and phenology (12.05%; padj < 1e-05; Figure 4D). Specifically, younger leaves tended to have shallower sinuses and exaggerated superior sinus depths (higher values on PC2), whereas older leaves tended to develop deeper petiolar sinuses and more shallow superior sinuses (lower values on PC2). The degree of this separation decreased across the season, and the shapes converged on the mean leaf shape on PC2, consistent with the middle leaf at all three phenological stages. PC2 additionally reflected the interaction of leaf position and rootstock (0.22%; p = 0.04; Supplemental Figure ~~4B~~5B), but post-hoc comparisons did not find any significant pairwise comparisons.

Machine learning on the GPA-rotated coordinate space identified moderate division of developmental and phenological classes. Random forest models could predict the leaf position with 73.1% accuracy, with the most important feature being the y-component of the leaf apex (MDA = 0.051). A model trained to predict phenology performed at 64.3% with the most important features being the x-

Formatted: Header

components of the points corresponding to superior sinus depth (left sinus MDA = 0.030, right sinus MDA = 0.019). A model trained to predict rootstock performed only marginally better than chance at 28.1% accuracy.

*Vine physiology*

~~For the 72-vine set, we~~We measured intracellular $CO_2$ concentration ($C_i$), stomatal conductance ($g_s$), leaf transpiration, water potential ($\psi$), and soil moisture for the 72-vine set (Figure 5). Each physiological ~~trait~~phenotype varied significantly across phenology and the block by phenology interaction (Figure 5A). For example, at harvest, we observed specific differences in leaf $CO_2$ concentration (A vs C: p=0.003; B vs C: p=0.002) and leaf transpiration (A vs B: $p < 1e-03$; A vs C: $p < 1e-05$; B vs C: $p < 1e-05$). Leaf transpiration and stomatal conductance varied significantly with the interaction of rootstock and phenology. A post-hoc comparison of means showed that leaf transpiration and stomatal conductances were elevated in 'Chambourcin' vines grafted to '1103P' at veraison as compared to leaves of ungrafted vines (leaf transpiration: p = 0.001; stomatal conductance: p = 0.002 Figure 5B-C).

*Phenomic ~~trait~~ covariation*

Four leaf ~~data~~phenotyping modalities consisted of ~~at least~~ 10 ~~traits~~or more measured phenotypes and were measured for all plants in the 72-vine set (leaf ionome, leaf metabolomics, gene expression, leaf shape). Using these data, we explored the extent to which different phenotypes (within and between modalities) covaried over phenology and rootstock genotype (Figure 6; Supplemental Figure ~~5~~6; Supplemental Figure ~~6~~7). Within each phenotyping modality, we summarized the primary dimensions of phenotypic variation using PCA (see Methods~~).~~), so as to not weigh any modality too heavily. From each PCA, we extracted the top 10 PCs, which explained a total of 88.9% of variation in the ionomics PCA (iPCA), 55.9% of the variation for the metabolomics PCA (mPCA), 74.8% of the variation in the gene expression PCA (gPCA) and 87.9% of the variation in the leaf shape PCA (sPCA).

Formatted: Header

Pairwise correlations of each PC within each phenological stage showed diverse correlation magnitudes and directions both within a phenotyping modality and between phenotyping modalities (Figure 6A-C; Supplemental Figure ~~5~~6). Generally, the strongest relationships were between PCs within ~~phenotypic~~phenotyping modalities. For example, the strongest correlations identified were between gene expression PCs gPC1 and gPC2 at anthesis (r = 0.85, CI = [0.81, 0.87]; Supplemental Figure ~~5A~~6A, and metabolomics PCs mPC1 and mPC2 at harvest (r = -0.78, CI = [-0.82. -0.76]). Correlations between modalities represented a diversity of responses across phenological stages. For example, the correlation between gene expression gPC4 and shape sPC3 ~~is~~was similar across the phenological stages, but only the correlation at veraison ~~is~~was significant (r = 0.41, CI = [0.34, 0.47]; Supplemental Figure ~~5B~~6B). Correlations such as between metabolomics mPC3 and gene expression gPC6 were similar and significant at both veraison (r = -0.44, CI = [-0.50, -0.37]; Supplemental Figure ~~5C~~6C) and harvest (r = -0.37, CI = [-0.45, -0.28]; Supplemental Figure 5C). While many correlations varied over the course of the season, some relationships entirely shifted in direction. For example, the correlation between metabolomics mPC3 and mPC6 shifted from a positive significant relationship (r = 0.58, CI = [0.52, 0.63]) at veraison to a negative significant relationship at veraison (r = -0.66, CI = [-0.73, -0.59]) (Supplemental Figure ~~5D~~6D).

Pairwise comparisons of PCs within each rootstock genotype show a suite of ~~traits~~latent phenotypes with significant presence/absence variation in significant correlations. Where each phenological stage showed modularity by phenotyping modality, variation over rootstock genotype shows a strong ionomics module with latent combination of other modalities interspersed (Supplemental Figure ~~6~~7). For example, in ungrafted vines, metabolomics mPC1 was correlated with four PCs from the ionome (Supplemental Figure ~~6A~~7A). Each of the other rootstock genotypes ~~have~~had dramatically different topologies with the ionome tending to be more connected within the ionome and connected to other modalities only on the periphery (Supplemental Figure ~~6B~~7B-D). Examples of presence/absence variation ~~are~~were shown in small modules of two latent phenotypes that ~~are~~were present in only one rootstock genotype. For example, in the ungrafted vines, the correlation between gene expression gPC4 and

Formatted: Header

metabolomics mPC3 was significant (r = -0.58, CI = [-0.65, -0.51]) and, in ‘1103P’-grafted vines, the correlation between metabolomics mPC3 and shape sPC6 (r = 0.59, CI = [0.53, 0.70]) was significant.

**Discussion**

In this study, we used grafted grapevines as an experimental system for characterizing root system impacts on ~~high~~ multi-dimensional leaf phenotypes over the course of a growing season. We detected ubiquitous but subtle effects of the root system on all assayed ~~phenotypes~~modalities, and demonstrated that ~~rootock~~rootstock influences on leaf phenotypes can be ~~season~~ specific to the vine’s developmental stage. The strongest signals of rootstock influences on leaves were observed in the ionomics dataset, phenotypes for which the root ~~systems have~~system has a noted and well-understood role.

*Phenology explains significant variation in all leaf phenotypes*

The timing of sampling or phenological stage of the vines (anthesis, veraison, harvest) was the strongest driver of ~~phenotypic~~phenomic variation for most leaf phenotypes. For example, all 20 ions varied with phenology and most ions showed that phenology, or the interaction of phenology with leaf developmental position, was the strongest source of variation (Figure 1). Nearly one third of all measured transcripts responded to seasonal variation, and the strongest effects on the transcriptome were phenology and row, a correlate for the time within a three-hour sampling window. The only phenotype for which phenology was not the most explanatory factor is leaf shape. Consistent with previous studies ~~[23]~~[23], we confirm that most of the leaf shape variation ~~measured~~ reflects development along a single shoot, but much of this variation is explained via interaction with phenology. These data highlight the dynamic nature of biological processes taking place within grapevines over the course of a season.

The seasonal component to grapevine ~~phenotypic~~phenomic variation is a subject of much research, especially in the berry. In studies designed to quantify molecular underpinnings of terroir, seasonal variation was identified as the strongest signal in the metabolome ~~[46–49].~~[51–54]. Several

Formatted: Header

studies have characterized transcriptomic variation over the course of the season. For example, in conjunction with metabolomics, seasonal variation of berry development was used to identify transcriptomic and metabolomic developmental markers in 'Corvina' [50].[55]. Follow-up analysis showed that nearly 18% of transcripts varied seasonally [51].[56]. Grapevine leavesleaf shape also varyvaries tremendously in shape over the growing season [23][23] and areis stable over multiple growing seasons; interestingly, grapevine leaves are patterned in the previous year, and the climate of the season in which the leaves were patterned influence aspects of leaf shape [52,53].[57,58].

*Grafting and rootstock genotype exhibit a complex and subtle signal on leaf phenotypes*

Consistent with previous studies, we confirm that grafting, as well as rootstock genotype, has a complex effect on phenotypicphenomic variation in the scion (the grafted shoot system). Most notably, we show that the rootstock to which a scion is grafted influences ion concentrations in leaves. Rootstock genotype is predictable from ion concentrations in the leaves; further,, and this signal is strengthened when phenological stage is included in the model. For example, we previously showed that nickel concentration was elevated in vines grafted to the rootstock 'SO4' [19].[19]. At a similar point in the season, we observe the same pattern, but by harvest, nickel was almost entirely excluded from the leaf. This suggests that the biological implications of this differential uptake could be missed if not surveyed across the season. We also confirm that rootstock genotype influences the metabolome of grafted grapevine, in some cases in a season-specific manner. In the transcriptome, PCA was able to identify dimensions of variation that were significantly described by rootstock and the interaction of rootstock and time of day, confirming prior observations [19].[19]. Patterns of gene expression were associated with rootstock in some analyses; for example, supervised methodologies identified linear discriminants in the PC space that separated gene expression patters of some rootstock genotypes. However, gene-by-gene analysis found no genes modulated by rootstock genotype, or even just from the act of grafting that were not driven entirely by a single outlier. We suspect these results are due, at least in part, to the strength of the phenology effect overpowering more subtle variation imparted by rootstock genotype. Finally, of the

Formatted: Header

physiology ~~traits~~phenotypes we measured, leaf transpiration and stomatal conductance were higher in vines grafted to '1103P' in the middle of the season. ~~Thus, the impact of grafting on leaf phenotypic variation varies by phenotype. Regardless,~~Through these analyses we ~~identify~~have identified subtle but ubiquitous effects ~~from~~of rootstock genotype on shoot system phenotype across modalities, and have shown that the impact of grafting on leaf phenomic variation varies from one phenotype to the next.

Understanding ~~of~~ rootstock genotype influence on shoot system phenotypes is a growing area of research, especially in grapevine. For example, in 'Cabernet Sauvignon', grafting increased ion uptake globally and some rootstock genotypes provide a clear signal in the scion ~~[28,54]. Also~~[28,59]. To our knowledge, there is not yet a strong causal link between the micronutrient component of the ionome and factors of vine growth or development that might influence traits like wine quality. However, it is noted that macronutrient deficiencies can have negative effects on such traits [60,61] and can be mediated by rootstock [62]. This suggests a strong understanding of the rootstock influence on the vine's ionome is warranted, and more work needs to be done to establish these relationships. Similarly, the metabolome is a key driver of the formation of the graft junction and some key metabolites could be responsible for graft incompatibility ~~[55].~~[63]. Building on this work, targeted metabolomics showed two classes of metabolites, flavanols and stilbenes, were differentially abundant at graft junctions and in the rootstocks of 'Cabernet Sauvignon' vines one month after grafting ~~[56].~~[64]. However, flavanols were not differentially abundant in the scion, but scion stilbene concentrations were apparently controlled by rootstock genotype. The effect of rootstock genotype on the scion transcriptome is perhaps the most varied. For example, 'Cabernet Sauvignon' shoot apical meristems show no effects by rootstock genotype ~~[14]~~[14], but berries of the same cultivar do, although the effect is tempered by seasonal variation ~~[15].~~[15]. Variation in 'Chambourcin' leaf shape ~~is~~was also driven by rootstock genotype, especially in conjunction with differences in irrigation ~~[19].~~[19]. Collectively, these studies all suggest that rootstock genotype influences scion phenotypes, but those effects will vary by phenotype, scion genotype, and perhaps other experimental conditions.

**Formatted:** Header

Data presented here confirm and expand upon previous observations of rootstock effects on scion phenotypes. Notably, ~~the~~this study was carried out using a robust experimental design (288 vine set and 72 vine set comprising replicates of three rootstocks grafted with a common scion and an ungrafted control), a vineyard that had been in the ground for eight years at the time of sampling. Our coordinated collection of five multi-dimensional leaf phenotypes, and inclusion of three sampling points spanning the growing season ~~allow~~allowed us to ~~hone~~investigate in ~~on~~ the comprehensive nature of rootstock influences on the scion. Further, this thorough analysis demonstrates that rootstock effects on scion phenotypes shift in magnitude over the course of the season, indicating that aspects of time are tremendously influential to the observed results regardless of phenotype.

While the results of previous studies on grafted grapevine are worthy of comparison, the work presented here has a few limitations that render comparisons with other studies challenging for a variety of reasons. One novelty in our study is the exploration of a hybrid grapevine system, 'Chambourcin'. 'Chambourcin' has a complex pedigree, including contributions from *Vitis riparia* and *V. rupestris*, species which are each parent to two of the rootstocks used in this study [65]. Many of the significant effects we observed in this study were subtle, which could reflect the genomic similarity between shoot and root systems. It might be expected that rootstocks derived from *V. riparia*,*V. rupestris* and other North American species might prompt more pronounced responses in European scions that lack North American *Vitis* in their pedigrees . Moreover, our results were derived from data collected in a single year at a single location. The phenotypes we measured are known to be heavily influenced by the environment, and we expect some inter-annual variation in rootstock influences on shoot system phenotypes. This study focused on a single scion, and as a result we are unable to explore how rootstock effects on shoot system phenotypes vary across scions. To our knowledge, this is among the largest populations to have been surveyed for such phenotypes in a decade old established vineyard. While many studies have been conducted in green houses or recently planted vineyards, the juxtaposition of our results and those previously established serve as a powerful foundation for the generation of hypotheses for future studies.

Formatted: Header

*Phenomic covariation warrants work toward latent phenotypes*

In the present study, we assess the extent of covariation among leaf phenotypes. For the primary dimensions of variation in each data typemodality, within-data typemodality correlations are strongest when accounting for phenological timing. Correlations also exist between phenotypesmodalities, suggesting room for the analysis of latent phenotypicphenomic structure or targeted integrative analyses for experimental questions. For example, aspects of the metabolome were frequently correlated with the transcriptome and leaf shape when accounting for both phenological stage and rootstock genotype. Interestingly, correlations within and between data types aremodalities were highly dynamic over a growing season and across rootstock genotype. For example, several correlations with leaf shape were present at veraison, but were not detected at anthesis and harvest. Moreover, the topology of connections in the ionomic network was variable over the rootstock genotype (Supplemental Figure 6). This variation in topology confirms that root system genotype has a strong influence on shoot system elemental composition, and suggests that root system genotype can alter correlative patterns in the ionome. We believe the work of understanding phenomic covariation warrants further investigation, specifically, by further including additional phenotypes such as lncRNA expression [57,58], epigenetics [59], and microbiomes [60,61]. Much[66,67], epigenetics [68], and microbiomes [69,70] which could yield more mechanistic understandings of the influence of root systems on shoot systems and how plants interact with their environments through their root systems. These mechanistic understandings could be used to further understand and optimize consumer-facing traits such as fruit quality and yield. To date, much of the work constituting phenomics in grapevine has addressed how berries develop over the growing season, how cultivars differ from one another, and how the concept of terroir influences wine [46,47,50,62–64].[51,52,55,71–73]. Despite data integration techniques becoming more popular, there are still many open questions as to what analytical methods are most appropriate and how to most effectively utilize them (reviewed for grapevine in [65,66][74,75]; reviewed broadly in [67,68]).[76,77]). Ongoing work attempts to integrate high-dimensional phenotypicphenomic datasets generated within a single

Formatted: Header

organ system (e.g., leaves); and future studies will expand this to explore phenomic covariation in and among organs, over time, and across space.

**Potential Implications**

Our work on the influence of root system genotype on shoot system phenotype has broad implications for a holistic understanding of how plants detect and respond to changing environmental conditions~~. In particular, this study highlights the influence of root system genotype and its interaction with phenology on shoot system phenotype: there is a seasonal component to the extent to which rootstock shapes phenotypic variation in the scion. Expanding this multi-dimensional understanding of phenotypic variation over time to include different tissues (e.g., root architecture, floral and fruit development), and different spatial scales (replicated root-shoot combinations located in geographically distinct vineyards) presents a challenging but exciting next frontier. Of particular note, patterns of phenomic covariation derived from complex datasets have implications for understanding how individuals perceive and respond to their environments, and how that response is coordinated throughout the plant body. This work is relevant for breeding efforts aimed at optimizing yield and other desired traits that can be optimized, or constrained by, phenotypic variation elsewhere in the plant~~<u>, and how this response is coordinated among different organ systems. Data presented here demonstrate that root systems that are genetically distinct from the scion exert influence on the scion, leading to statistically significant differences in scion phenotypes based on the identity of their root systems. This observation suggests that the above-ground phenotype of plants results, at least in part, from below-ground activity of the root system. Further, these data highlight the value of coordinated collection of different multi-dimensional phenotypes for comparative studies, and for describing whole-plant phenotypic shifts over seasons and in response to horticultural manipulations</u>.

<u>Beyond its use as an experimental model that is ideal for studying root/shoot interaction, grafting is an important horticultural technique that is used in over 70 major crops. In grapevines, grafting was developed primarily to combat the below-ground pest phylloxera, and grapevine rootstocks were selected</u>

Formatted: Header

initially based on their resistance to this pest. Results presented here indicate that beyond phylloxera resistance, grafting to genetically distinct rootstocks is a potential source of variation for the scion. Ongoing work explores how root system impacts on shoot system phenotypes vary across scion genotypes, and how the rootstock × scion interaction changes over space. The long-term implications of this study are the potential honing of viticulture for future climates including the optimization of rootstock-scion combinations based in part on an understanding of how rootstock effects on scion phenotypes change over the course of the season. This work is relevant for breeding efforts, and may play a role in the optimization of quantitative phenotypes such as vigor, fruit quality, and yield that may be enhanced by, constrained by, or partially predicted from phenotypic variation elsewhere in the plant.

Formatted: Normal, Indent: First line: 0.5"

## Methods

*Study Design*

Data were collected in 2017 in anfrom a split-plot experimental rootstock trial established in 2009 at the University of Missouri's Southwest Research Center near Mount Vernon, MO (37.074167 N; 93.879167 W; Supplemental Figure 1). The rootstock trial includes the interspecific hybrid cultivar 'Chambourcin' growing ungrafted (own-rooted) and grafted to three rootstocks: '1103P', '3309C', and 'SO4' (Supplemental Figure 1D). EachClonal replicates of each of the four rootstock-scion combinations was replicatedwere planted 72 times for a total of 288 vines planted in nine rows. Each row was treated with one of three irrigation treatments: full evapotranspiration replacement, partial (50%) evapotranspiration replacement (reduced deficit irrigation; RDI), or no evapotranspiration replacement (Supplemental Figure 1A). However, rainfall in 2017 likely mitigated the applied irrigation treatment (see Supplemental Note at: https://github.com/PGRP1546869/mt_vernon_2017_leaf/blob/main/On_the_irrigation_treatment.pdf).1). Vine position in the vineyard corresponded to time of sampling for some phenotypes, (metabolomics, gene expression, and physiology), as samples were taken from one end of the vineyard to the other over

**Formatted:** Header

the course of two to three hours. Because vineyard microclimates and sampling time may be associated with ~~phenotypic~~phenomic variation, we defined '~~temporal~~ block' as a factor that captures this spatial and temporal variation inherent in sampling for those phenotypes. In the other phenotypes (ionomics and leaf shape), neither row nor block correlated with time, so 'block' was simply a spatial covariate. Unique rootstock-scion combinations were planted in cells of four adjacent replicated vines (Supplemental Figure ~~1B~~1A-B), with rows consisting of eight cells (32 vines/row). To our knowledge, a field-planted rootstock experimental vineyard of this size and age is rare. For some phenotypes (~~leaves for ionome~~ionomics and leaf shape~~ analysis~~), it was possible to collect samples from all vines in the experimental vineyard (the 288-vine set; Supplemental Figure ~~1C~~1A-B). For other phenotypes (~~physiology,~~ metabolomics, ~~and~~ gene expression, and physiology), time and/or expense associated with the phenotyping process required that we reduce sampling to a nested set of 72 vines representing the middle two vines in each four-vine cell in the front half of the vineyard (the 72-vine set; Supplemental Figure ~~1C~~1B-C). All phenotypes were assayed ~~\~~at three phenological stages: anthesis (~80% of open flowers; 22 May 2017); veraison (~50% of berries had transitioned from green to red; 30 July 2017); and immediately prior to harvest (25 September 2017). At each phenological stage, effort was made to sample on days with full to partial sun and minimal precipitation.

This design was used to assess the following questions: 1) What is the influence of root system genotype on shoot system phenotype? 2) How do systems of plant phenotypes vary over the growing season and does rootstock genotype influence this variation? And 3) how do phenotypes covary within and between phenotyping modalities?

*Linear Models*

Linear models were fit to the 20 measured ion concentrations, the top 20 PCs of the leaf metabolome, the top 100 PCs of the leaf transcriptome, the top 20 PCs of leaf morphospace, and each measured physiological trait. Outliers were detected using the R function 'anomalize' (options: alpha=0.03, max_anoms=0.1). Each model was fit with fixed effect factors representing phenological

Formatted: Header

stage (anthesis, veraison, or harvest), rootstock (Ungrafted, ‘1103P’, ‘3309C’, or ‘SO4’), leaf position (youngest, middle, or oldest; only used in leaf morphology and leaf ion concentration models), and all pairwise interactions of those terms. Both irrigation and block were included as fixed, non-interacting effects with the exceptions of physiology and metabolomics, for which we allowed the interaction of ‘Block’ as it correlates with the time of sampling, potentially capturing temporal variation. Row, an additional correlate for time and spatial variation, was included in place of a temporal block for the gene expression models after removal of the variation attributable to irrigation, a factor collinear with row. All linear models were interpreted using a type-3 sum of squares computation using the R package ‘car’ ~~[69].~~[78]. Estimated p-values for each term in the models were corrected for multiple tests (within phenotype) using FDR correction as implemented by the R package ‘stats’ ~~[70].~~[79]. Results from the models are reported as the variation explained by a particular term in the model and the estimated p-value. When appropriate, post-hoc mean comparisons were computed using the package ‘emmeans’ ~~[71].~~[80]. Where multiple linear models were being simultaneously interpreted, we applied a Bonferonni correction to reduce the number of false positives.

*Machine Learning to Identify Rootstock Effects*

For visualization of between-class variation, we fit linear discriminant analysis models (LDA) to ~~the full phenotypic data sets of~~ each modality (ionomics, metabolomics, gene expression, and leaf morphology) using the ‘lda’ function of the R package ‘MASS’ ~~[72]~~[81]. Projections of all samples into the LD space were plotted using ggplot2 ~~[73].~~[82]. In addition, we employed machine learning to capture subtle experimental effects. We partitioned ~~phenotypic~~ data ~~sets~~from each modality into 80% training partitions and 20% testing partitions. Models were fit to predict the phenological stage from which a sample was taken, the rootstock to which the scion was grafted, and the joint prediction of phenology and rootstock. We also tested the predictability of leaf position for ionomics and leaf shape, and the interaction of rootstock and leaf position for ionomics. We used the ‘randomForest’ ~~[74]~~[83] implementation of the random forest algorithm. Models were fit and tuned using the R package ‘caret’

[75].[84]. Each performance was assessed using accuracy, with performance on each class being assessed using the balanced accuracy, the midpoint of class-wise sensitivity and specificity. Where appropriate, models were compared to ‘chance’, or the occurrence frequency of each class. Confusion matrices were visualized from the out-of-bag predictions using ggplot2. Important features were identified from the randomForest object based on a phenotype-specific mean decrease in model accuracy (MDA).

*Phenomic trait covariation*

We extracted ionomics, metabolomics, gene expression, and leaf shape data for the youngest available leaf from the 72 vine-set. Each class of phenotypic data modality was summarized along the primary dimensions of variation using PCA. For each class, we extracted the top 10 PCs and fit Pearson’s correlations across all pairs of PCs at each phenological stage. P-values from computed correlations were corrected using the FDR method from the package ‘stats’ [76].[85]. Correlations and their strengths were visualized using the R package ‘igraph’ [77].[86]. Example correlations were reported after running 10,000 bootstrapped subsamples of 90% of data for paired traitsphenotypes. From the distribution of estimated correlation coefficients, confidence intervals were computed from the 0.025 and 0.975 quantiles. A subset of example correlations were plotted using the R package ‘ggplot2’ [7382].

**Acknowledgments:**

This work was funded by the National Science Foundation Plant Genome Research Project 1546869. We thank members of the Miller Lab at Saint Louis University and the Donald Danforth Plant Science Center, members of the Kovacs Lab at Missouri State University, members of the Kwasniewski Lab at the University of Missouri, and members Londo Lab at the USDA-ARS Grape Research Unit for vineyard sampling and sample processing. We express special thanks to Matthew Rubin and Elizabeth Kellogg at the Donald Danforth Plant Science Center for valuable comments on the manuscript. Finally, we thank the reviewers of this manuscript whose comments have led to clearer and more complete manuscript.



**Formatted:** Header

**Field Code Changed**

Formatted: Header

**Figure Legends:**

Figure 1: The ionome shows strong signal from rootstock genotype, leaf position, and phenological stage **(A)** Percent variation captured in linear models fit to each of 20 ions measured in the ionomics pipeline. Presence of a cell indicates the model term (top) was significant (FDR; p.adj < 0.05) for that ion (left). **(B)** Example ions shown to vary significantly by the interaction of leaf position (Y=Youngest, M=Middle, O=Oldest) and phenological stage~~.~~ in parts per million. Boxes are bound by 25th and 75th percentile with whiskers extending 1.5 IQR from the box. Significant changes are indicated by letters above boxes, and are only meant for comparison within each phenological stage. Group means are displayed with black squares. (**C**) Example ions shown to vary significantly by the interaction of rootstock genotype and phenological ~~state.~~stage in parts per million. Significant changes are indicated by letters above boxes, and are only meant for comparison within each phenological stage. Boxes are bound by 25th and 75th percentile with whiskers extending 1.5 IQR from the box. Group means are displayed with black squares. (**D**) Standardized heatmap for out-of-bag (OOB) predictions by a random forest trained to predict rootstock genotype, **(E)** the interaction between rootstock genotype by phenology, and **(F)** the interaction between rootstock genotype and leaf position.

Formatted: Font color: Black

Figure 2: The metabolome is influenced by rootstock genotype, phenological stage, and time of sampling. **(A)** Percent variation captured in linear models fit to each of the top 20 principal components of the metabolome (661 measured metabolites). Presence of a cell indicates the model term (top) was significant for that PC (left, percent variation explained by the PC in parentheses). **(B)** The distribution of projections onto PC17, the strongest captured rootstock effect in the metabolome. Boxes are bound by the 25th and 75th percentiles with whiskers extending 1.5 IQR from the box. **(C)** Projections of all samples into the first two dimensions of a linear discriminant space trained to maximize variation between rootstock genotypes.

Formatted: Font color: Black

Figure 3: Gene expression primarily responds to time of season and circadian correlates

Formatted: Font color: Black

**(A)** Heatmap showing 500 genes with the highest variance following the filtering of lowly expressed genes and gene-by-gene variance stabilizing transformations (VST) ordered by example model factors (below). **(B)** Percent variation captured in linear models fit to the top 100 Principal Components of the VST-transformed gene-expression space. Presence of a cell indicates the model term (top) was significant for that PC (left, percent variation explained by the PC in parentheses). **(C)** Projections of all samples into the first two principal component dimensions to show that the largest descriptors of variation are due to phenology. **(D)** Projections of all samples into the first two dimensions of the linear discriminant space trained to maximize variation between the rows of the vineyard, and **(E)** rootstock genotype.

Figure 4: Leaf shape variation is primarily determined by shoot position but changes over the season

**(A)** Representative shapes showing leaf variation (-3 sd, mean, +3 sd) captured in each of the top 4 principal components of the Generalized Procrustes Analysis-rotated leaf shapes. **(B)** Projections of all leaves into the first two dimensions of principal component space colored by the strongest determinant of variation in the top two PCs. **(C)** Projections of all leaves into the first two dimensions of a linear discriminant space trained to maximize variation between phenological stages. **(D)** Variation in leaf shape captured on PC2 shown by leaf position and phenological stage. Large points represent the mean of the group when projected onto PC2. Bars surrounding the mean show one standard deviation. Variation in each group is shown as a composite leaf trace scaled to a standard size and centered over the mean.

Figure 5: Vine physiology ~~measurements show signal from most experimental manipulation~~varies with rootstock and the rootstock by phenology interaction

**(A)** Percent variation explained by model terms (top) from linear models fit to each of four physiology traits (left). **(B)** Variation in leaf transpiration rate for each rootstock genotype over the course of the season. Boxes are bound by the 25th and 75th percentiles with whiskers extending 1.5 IQR from the box. Significant changes are indicated by letters above boxes, and are only meant for comparison within each phenological stage. Group means are displayed with black squares. **(C)** Variation in stomatal conductance

**Formatted:** Header

**Formatted:** Font color: Black

**Formatted:** Font color: Black

for each rootstock genotype over the course of the season. Boxes are bound by the 25th and 75th percentiles with whiskers extending 1.5 IQR from the box. Group means are displayed with black squares. Significant changes are indicated by letters above boxes, and are only meant for comparison within each phenological stage.

Figure 6: ~~Trait~~Phenomic covariation varies over the course of the season

Correlation networks showing patterns of covariation within and between phenotyping modalities. Nodes of the network are connected if they are significantly correlated (Pearson, FDR; p.adj < 0.05). Edge thickness is proportional to the strength of correlation (multiplied by 16 for visibility). Edge color reflects the direction of the correlation where blue edges indicate positive correlations and orange edges indicate negative correlations. Modalities are indicated by a leading character and node color: ionomics (iPCs; purple), metabolomics (mPCs; pink), gene expression (gPCs; yellow), leaf shape (sPCs; green). Network topologies are shown for **(A)** anthesis, **(B)** veraison, and **(C)** harvest.

**Figure Supplement Legends:**

Supplemental Figure 1: Experimental Design

**(A)** Vineyard Map. The vineyard features a randomized block design where ‘Chambourcin’ is grown ungrafted and grafted to three rootstock genotypes: ‘1103P’, ‘3309C’, and ‘SO4’. Each row is treated with one of three irrigation treatments: full replacement of ET, reduced-deficit, no replacement of ET. Each cell of the vineyard contains four replicate grafts. **(B)** Phenotype sampling scheme across the four replicates in a cell. For example, the top panel (purple) shows all four vines in the first cell of Row 8 in Block D. From each vine in that cell, ionomics and leaf shape were sampled. In contrast, the lower panel shows the first cell in Row 8 in Block A. Here, the first and fourth replicates were sampled for ionomics and leaf shape while the second and third replicates were sampled for all phenotypes. All vines (288) were sampled for ionomics and leaf shape. The middle two vines in the front half of the vineyard (72) were additionally sampled for metabolomics, gene expression, and physiology. **(C)** Phenotype sample

**Formatted:** Header

**Formatted:** Font color: Black

**Formatted:** Font color: Black

scheme within a vine (along a shoot). For each plant, young leaves were sampled for ionomics, leaf shape, and gene expression. Middle leaves were sampled for ionomics, leaf shape, metabolomics, and physiology. Older leaves were sampled for ionomics and leaf shape. Samples for ionomics and leaf shape were taken from the same shoot. All other phenotypes were sampled from independent shoots. **(D)** Rootstock relatedness. Each of the rootstocks in this trial shares a parent species with a different rootstock. '1103P' is a cross between *Vitis rupestris* and *V. berlandieri*. '3309C' is a cross between *V. rupestris* and *V. riparia*. 'SO4' is a cross between *V. riparia* and *V. berlandieri*. The parent that is shared between each pair of rootstocks is highlighted. This figure is partially reproduced from [19] available under a Creative Common license (CC BY 4.0).

Supplemental Figure 2Supplemental Figure 2: Quality and validity assessment of 3' RNAseq data. **(A)** A survey of recently annotated circadian clock orthologs from the grapevine genome annotation [44]. Orthologs surveyed included the morning-phased RVE1 and LHY, evening-phased LUX and ELF4, and the nigh-phased TOC1 **(B)** A survey of genes with housekeeping domains related to IPR000626 (ubiquitin) and IPR004000 (actin).

Supplemental Figure 3: Patterns of ion covariation change over experimental treatments
Correlation networks showing patterns of ion covariation across phenological stages and shoot position. Nodes of the network are connected if they are significantly correlated (Pearson, FDR; p.adj < 0.05). Edge thickness is proportional to the strength of correlation (multiplied by 16 for visibility). Edge color reflects the direction of the correlation where blue edges indicate positive correlations and orange edges indicate negative correlations.

Supplemental Figure 34: Patterns of variation contributing to gene expression linear discriminants
(**A**) Projections of leaf gene expression samples into the first two dimensions of a linear discriminant space trained to maximize variation between phenological stages, rows in the vineyard, and rootstock

**Formatted:** Header
**Formatted:** Font color: Black
**Field Code Changed**
**Formatted:** Font color: Black
**Formatted:** Font color: Black

Formatted: Header

genotype. For each LD, the PCs that loaded significantly (>1.96 sd from the mean loading) are listed in order of loading magnitude. (**B**) Distribution of the top loading PCs onto LD1 and LD2 for each of the trained models.

Supplemental Figure ~~4~~5: Patterns of variation in leaf shape are subtle

Formatted: Font color: Black

Formatted: Font color: Black

Formatted: Font color: Black

(**A**) Percent variation captured in linear models fit to each of the top 20 principal components of leaf morphology. Presence of a cell indicates the model term (top) was significant for that PC (left, percent variation explained by the PC in parentheses). (**B**) Composite leaf traces for the main rootstock genotype effect identified on PC1.

Supplemental Figure ~~5~~6: Example correlations within and between ~~data~~phenotyping modalities over the course of the season

Formatted: Font color: Black

Formatted: Font color: Black

(**A**) Example correlation showing a strong within-modality correlation between the ionomics gPC1 and gPC2 at anthesis. Pearson correlations by phenological stage and CIs derived from 10000 random 90% draws are shown for each panel. Generally speaking, CIs overlapping with 0 were not accepted as significant. (**B**) Example correlation showing one of the stronger between-modality correlations between the gene expression gPC4 and morphology (shape) sPC3 at veraison. (**C**) Example correlation of a relationship that is present multiple times over the course of the season between metabolomics mPC3 and gene expression gPC6 at both veraison and harvest. (**D**) Example correlation that is dynamic over the course of the growing season between the ionomics mPC3 and mPC6.

Supplemental Figure ~~6: Trait~~7: Phenomic covariation varies over rootstock genotype

Formatted: Font color: Black

Correlation networks showing patterns of covariation within and between phenotyping modalities. Nodes of the network are connected if they are significantly correlated (Pearson, FDR; p.adj < 0.05). Edge thickness is proportional to the strength of correlation (multiplied by 16 for visibility). Edge color reflects

Formatted: Header

the direction of the correlation where blue edges indicate positive correlations and orange edges indicate negative correlations. Modalities are indicated by a leading character and node color: ionomics (iPCs; purple), metabolomics (mPCs; pink), gene expression (gPCs; yellow), leaf shape (sPCs; green). Network topologies are shown for **(A)** Ungrafted, **(B)** ‘1103P’-grafted vines, **(C)** ‘3309C’-grafted vines, and **(D)** ‘SO4’-grafted vines.

**Availability of Data:**

Ionomics data are available at https://dx.doi.org/10.6084/m9.figshare.13200980 . Metabolomics data are available at https://dx.doi.org/10.6084/m9.figshare.13201043. Gene expression data are available in the Sequence Read Archive under BioProject PRJNA674915. Leaf scans and leaf landmarks are available at https://dx.doi.org/10.6084/m9.figshare.13200953. Weather and physiology data are available at https://dx.doi.org/10.6084/m9.figshare.13198682 and https://dx.doi.org/10.6084/m9.figshare.13201016, respectively.

**Availability of Code:**

All code for this paper including shell scripts for RNAseq analysis and Jupyter Notebooks for data analysis in R can be found on the Vitis Underground GitHub (https://github.com/PGRP1546869/mt_vernon_2017_leaf).

**Author Contributions:**

AJM, DHC, AF, LGK, MK, JPL, and QM designed the experiment. ZNH, LLK, MA, JFS, ZM, NB, EF, and JPL contributed to sample collection and sample processing. ZNH, LLK, JFS, and MA contributed to data analysis. ZNH and AJM contributed to the writing of the manuscript. All authors contributed to manuscript editing.

Formatted: Header

**References:**


~~1. Gehan MA, Fahlgren N, Abbasi A, Berry JC, Callen ST, Chavez L, et al.. PlantCV v2: Image analysis software for high-throughput plant phenotyping. *PeerJ*. 2017; doi: 10.7717/peerj.4088.~~

~~2. Ubbens JR, Stavness I. Deep Plant Phenomics: A Deep Learning Platform for Complex Plant Phenotyping Tasks. *Front Plant Sci*. 2017; doi: 10.3389/fpls.2017.01190.~~

~~3. Ubbens J, Cieslak M, Prusinkiewicz P, Stavness I. Latent Space Phenotyping: Automatic Image-Based Phenotyping for Treatment Studies.~~

~~4. Soulé M. PHENETICS OF NATURAL POPULATIONS I. PHENETIC RELATIONSHIPS OF INSULAR POPULATIONS OF THE SIDE-BLOTCHED LIZARD. *Evolution*. 1967; doi: 10.1111/j.1558-5646.1967.tb03413.x.~~

~~5. Houle D, Govindaraju DR, Omholt S. Phenomics: the next challenge. *Nat Rev Genet*. 2010; doi: 10.1038/nrg2897.~~

~~6. Mudge K, Janick J, Scofield S, Goldschmidt EE. A History of Grafting. In: Janick J, editor. *Horticultural Reviews*. Hoboken, NJ, USA: John Wiley & Sons, Inc.;~~

~~7. Pouget R. Histoire de la lutte contre le phylloxéra de la vigne en France: 1868-1895. *Hist Sci Med*. INRA; 1990;~~

~~8. Walker MA, Lund K, Agüero C, Riaz S, Fort K, Heinitz C, et al.. BREEDING GRAPE ROOTSTOCKS FOR RESISTANCE TO PHYLLOXERA AND NEMATODES – IT'S NOT ALWAYS EASY. Acta Horticulturae.~~

~~9. Warschefsky EJ, Klein LL, Frank MH, Chitwood DH, Londo JP, von Wettberg EJB, et al.. Rootstocks: Diversity, Domestication, and Impacts on Shoot Phenotypes. *Trends Plant Sci*. Elsevier Current Trends; 2016; doi: 10.1016/j.tplants.2015.11.008.~~

Formatted: Header


~~10. Tramontini S, Vitali M, Centioni L, Schubert A, Lovisolo C. Rootstock control of scion response to water stress in grapevine. Environmental and Experimental Botany.~~

~~11. Bavaresco L, Lovisolo C. Effect of grafting on grapevine chlorosis and hydraulic conductivity. *VITIS Journal of Grapevine Research*. Citeseer; 2015;~~

~~12. Ferlito F, Distefano G, Gentile A, Allegra M, Lakso AN, Nicolosi E. Scion–rootstock interactions influence the growth and behaviour of the grapevine root system in a heavy clay soil. Australian Journal of Grape and Wine Research.~~

~~13. Ordish G. The great wine blight. J.M. Dent & Sons;~~

~~14. Cookson SJ, Ollat N. Grafting with rootstocks induces extensive transcriptional re-programming in the shoot apical meristem of grapevine. *BMC Plant Biol*. BioMed Central; 2013; doi: 10.1186/1471-2229-13-147.~~

~~15. Corso M, Vannozzi A, Ziliotto F, Zouine M, Maza E, Nicolato T, et al.. Grapevine Rootstocks Differentially Affect the Rate of Ripening and Modulate Auxin-Related Genes in Cabernet Sauvignon Berries. *Front Plant Sci*. Frontiers; 2016; doi:~~ 1. Gehan MA, Fahlgren N, Abbasi A, Berry JC, Callen ST, Chavez L, et al.. PlantCV v2: Image analysis software for high-throughput plant phenotyping. *PeerJ*. 5:e40882017;

2. Ubbens JR, Stavness I. Deep Plant Phenomics: A Deep Learning Platform for Complex Plant Phenotyping Tasks. *Front Plant Sci*. 8:11902017;

3. Ubbens J, Cieslak M, Prusinkiewicz P, Stavness I. Latent Space Phenotyping: Automatic Image-Based Phenotyping for Treatment Studies.

4. Soulé M. PHENETICS OF NATURAL POPULATIONS I. PHENETIC RELATIONSHIPS OF INSULAR POPULATIONS OF THE SIDE-BLOTCHED LIZARD. *Evolution*. 21:584–911967;

5. Houle D, Govindaraju DR, Omholt S. Phenomics: the next challenge. *Nat Rev Genet*. 11:855–662010;

6. Mudge K, Janick J, Scofield S, Goldschmidt EE. A History of Grafting. In: Janick J, editor. *Horticultural Reviews*. Hoboken, NJ, USA: John Wiley & Sons, Inc.; p. 437–93.

7. Pouget R. Histoire de la lutte contre le phylloxéra de la vigne en France: 1868-1895. *Hist Sci Med*. INRA; 1990;

8. Walker MA, Lund K, Agüero C, Riaz S, Fort K, Heinitz C, et al.. BREEDING GRAPE ROOTSTOCKS FOR RESISTANCE TO PHYLLOXERA AND NEMATODES - IT'S NOT ALWAYS

EASY. Acta Horticulturae.

9. Warschefsky EJ, Klein LL, Frank MH, Chitwood DH, Londo JP, von Wettberg EJB, et al.. Rootstocks: Diversity, Domestication, and Impacts on Shoot Phenotypes. *Trends Plant Sci*. Elsevier Current Trends; 21:418–372016;

10. Tramontini S, Vitali M, Centioni L, Schubert A, Lovisolo C. Rootstock control of scion response to water stress in grapevine. Environmental and Experimental Botany.

11. Bavaresco L, Lovisolo C. Effect of grafting on grapevine chlorosis and hydraulic conductivity. *VITIS-Journal of Grapevine Research*. Citeseer; 2015;

12. Ferlito F, Distefano G, Gentile A, Allegra M, Lakso AN, Nicolosi E. Scion–rootstock interactions influence the growth and behaviour of the grapevine root system in a heavy clay soil. Australian Journal of Grape and Wine Research.

13. Ordish G. The great wine blight. J.M. Dent & Sons;

14. Cookson SJ, Ollat N. Grafting with rootstocks induces extensive transcriptional re-programming in the shoot apical meristem of grapevine. *BMC Plant Biol*. BioMed Central; 13:1–142013;

15. Corso M, Vannozzi A, Ziliotto F, Zouine M, Maza E, Nicolato T, et al.. Grapevine Rootstocks Differentially Affect the Rate of Ripening and Modulate Auxin-Related Genes in Cabernet Sauvignon Berries. *Front Plant Sci*. Frontiers; 2016; doi: 10.3389/fpls.2016.00069.

~~16. Berdeja M, Nicolas P, Kappel C, Dai ZW, Hilbert G, Peccoux A, et al.. Water limitation and rootstock genotype interact to alter grape berry metabolism through transcriptome reprogramming. *Hortic Res*. 2015; doi: 10.1038/hortres.2015.12.~~

~~17. Zombardo A, Crosatti C, Bagnaresi P, Bassolino L, Reshef N, Puccioni S, et al.. Transcriptomic and biochemical investigations support the role of rootstock-scion interaction in grapevine berry quality. *BMC Genomics*. 2020; doi: 10.1186/s12864-020-06795-5.~~

~~18. Chitarra W, Perrone I, Avanzato CG, Minio A, Boccacci P, Santini D, et al.. Grapevine Grafting: Scion Transcript Profiling and Defense-Related Metabolites Induced by Rootstocks. *Front Plant Sci*. Frontiers; 2017; doi:~~ 16. Berdeja M, Nicolas P, Kappel C, Dai ZW, Hilbert G, Peccoux A, et al.. Water limitation and rootstock genotype interact to alter grape berry metabolism through transcriptome reprogramming. *Hortic Res*. 2:150122015;

17. Zombardo A, Crosatti C, Bagnaresi P, Bassolino L, Reshef N, Puccioni S, et al.. Transcriptomic and biochemical investigations support the role of rootstock-scion interaction in grapevine berry quality. *BMC Genomics*. 21:4682020;

18. Chitarra W, Perrone I, Avanzato CG, Minio A, Boccacci P, Santini D, et al.. Grapevine Grafting: Scion Transcript Profiling and Defense-Related Metabolites Induced by Rootstocks. *Front Plant Sci*. Frontiers; 2017; doi: 10.3389/fpls.2017.00654.

**Formatted:** Header

**Formatted:** Line spacing: single

**Formatted:** Line spacing: single

**Formatted:** Header


19. Migicovsky Z, Harris ZN, Klein LL, Li M, McDermaid A, Chitwood DH, et al.. Rootstock effects on scion phenotypes in a “Chambourcin” experimental vineyard. *Horticulture Research*. Nature Publishing Group; 2019; doi: 10.1038/s41438-019-0146-2.

20. Galet P. A Practical Ampelography: Grapevine Identification. Comstock Pub. Associates;

21. Mullins MG, Bouquet A, Williams LE. Biology of the Grapevine. Cambridge University Press;

22. Chitwood DH, Ranjan A, Martinez CC, Headland LR, Thiem T, Kumar R, et al.. A modern ampelography: a genetic basis for leaf shape and venation patterning in grape. *Plant Physiol*. 2014; doi: 10.1104/pp.113.229708.

23. Chitwood DH, Klein LL, O’Hanlon R, Chacko S, Greg M, Kitchen C, et al.. Latent developmental and evolutionary shapes embedded within the grapevine leaf. New Phytologist.

24. Klein LL, Caito M, Chapnick C, Kitchen C, O’Hanlon R, Chitwood DH, et al.. Digital Morphometrics of Two North American Grapevines (Vitis: Vitaceae) Quantifies Leaf Variation between Species, within Species, and among Individuals. *Front Plant Sci*. 2017; doi: 10.3389/fpls.2017.00373.

25. Grimes DW, Williams LE. Irrigation Effects on Plant Water Relations and Productivity of Thompson Seedless Grapevines. *Crop Sci*. 1990; doi: 10.2135/cropsci1990.0011183X003000020003x.

26. Williams LE, Grimes DW. Modelling vine growth-development of a data set for a water balance subroutine. *Proceedings of the Sixth Australian Wine Industry Technical Conference*. p. 169–74.

27. Gautier A, Cookson SJ, Lagalle L, Ollat N, Marguerit E. Influence of the three main genetic backgrounds of grapevine rootstocks on petiolar nutrient concentrations of the scion, with a focus on phosphorus. *OENO One*. 2020; doi: 10.20870/oeno-one.2020.54.1.2458.

28. Lecourt J, Lauvergeat V, Ollat N, Vivin P, Cookson SJ. Shoot and root ionome responses to nitrate supply in grafted grapevines are rootstock genotype dependent: Rootstock and nitrogen supply affect

Formatted: Header

grapevine ionome. *Aust J Grape Wine Res*. 2015; doi: 10.1111/ajgw.12136.

29. Salt DE, Baxter I, Lahner B. Ionomics and the study of the plant ionome. *Annu Rev Plant Biol*. 2008; doi: 10.1146/annurev.arplant.59.032607.092942.

30. Baxter I. Ionomics: The functional genomics of elements. *Brief Funct Genomics*. 2010; doi: 10.1093/bfgp/elp055.

31. Ziegler G, Terauchi A, Becker A, Armstrong P, Hudson K, Baxter I. Ionomic Screening of Field-Grown Soybean Identifies Mutants with Altered Seed Elemental Composition. The Plant Genome.

32. Oliver SG, Winson MK, Kell DB, Baganz F. Systematic functional analysis of the yeast genome. *Trends Biotechnol*. 1998; doi: 10.1016/s0167-7799(98)01214-1.

33. Tweeddale H, Notley-McRobb L, Ferenci T. Effect of slow growth on metabolism of Escherichia coli, as revealed by global metabolite pool (“metabolome”) analysis. *J Bacteriol*. 1998; doi: 10.1128/JB.180.19.5109-5116.1998.

34. Islam MN, Downey F, Ng CKY. Comparative analysis of bioactive phytochemicals from Scutellaria baicalensis, Scutellaria lateriflora, Scutellaria racemosa, Scutellaria tomentosa and Scutellaria wrightii by LC-DAD-MS. *Metabolomics*. Springer; 7:446–532011;

35. Tautenhahn R, Patti GJ, Rinehart D, Siuzdak G. XCMS Online: a web-based platform to process untargeted metabolomic data. *Anal Chem*. 2012; doi: 10.1021/ac300698c.

36. Tandonnet S, Torres TT. Traditional versus 3’ RNA-seq in a non-model species. *Genom Data*. 2017; doi: 10.1016/j.gdata.2016.11.002.

37. Bolger AM, Lohse M, Usadel B. Trimmomatic: a flexible trimmer for Illumina sequence data. *Bioinformatics*. 2014; doi: 10.1093/bioinformatics/btu170.

Formatted: Header


38. Bushnell B. BBTools software package. *URL http://sourceforge net/projects/bbmap*. 2017;

39. Jaillon O, Aury J-M, Noel B, Policriti A, Clepet C, Casagrande A, et al.. The grapevine genome sequence suggests ancestral hexaploidization in major angiosperm phyla. *Nature*. 2007; doi: 10.1038/nature06148.

40. Canaguier A, Grimplet J, Di Gaspero G, Scalabrin S, Duchêne E, Choisne N, et al.. A new version of the grapevine reference genome assembly (12X.v2) and of its annotation (VCost.v3). *Genom Data*. 2017; doi: 10.1016/j.gdata.2017.09.002.

41. Dobin A, Davis CA, Schlesinger F, Drenkow J, Zaleski C, Jha S, et al.. STAR: ultrafast universal RNA-seq aligner. *Bioinformatics*. 2013; doi: 10.1093/bioinformatics/bts635.

42. Anders S, Pyl PT, Huber W. HTSeq: Analysing high-throughput sequencing data with Python.

43. Love MI, Huber W, Anders S. Moderated estimation of fold change and dispersion for RNA-seq data with DESeq2. *Genome Biol*. 2014; doi: 10.1186/s13059-014-0550-8.

44. Anders S, Huber W. Differential expression analysis for sequence count data. *Genome Biol*. 2010; doi: 10.1186/gb-2010-11-10-r106.

45. Dryden IL, Mardia KV. Statistical Shape Analysis: With Applications in R. John Wiley & Sons;

46. Degu A, Hochberg U, Sikron N, Venturini L, Buson G, Ghan R, et al.. Metabolite and transcript profiling of berry skin during fruit development elucidates differential regulation between Cabernet Sauvignon and Shiraz cultivars at branching points in the polyphenol pathway. *BMC Plant Biol*. 2014; doi: 10.1186/s12870-014-0188-4.

47. Anesi A, Stocchero M, Dal Santo S, Commisso M, Zenoni S, Ceoldo S, et al.. Towards a scientific interpretation of the terroir concept: plasticity of the grape berry metabolome. *BMC Plant Biol*. 2015; doi: 10.1186/s12870-015-0584-4.

Formatted: Header


~~48. Cuadros-Inostroza A, Ruíz-Lara S, González E, Eckardt A, Willmitzer L, Peña-Cortés H. GC-MS metabolic profiling of Cabernet Sauvignon and Merlot cultivars during grapevine berry development and network analysis reveals a stage- and cultivar-dependent connectivity of primary metabolites. *Metabolomics*. 2016; doi: 10.1007/s11306-015-0927-z.~~

~~49. Dal Santo S, Fasoli M, Negri S, D'Incà E, Vicenzi N, Guzzo F, et al.. Plasticity of the Berry Ripening Program in a White Grape Variety. *Front Plant Sci*. 2016; doi: 10.3389/fpls.2016.00970.~~

~~50. Zamboni A, Di Carli M, Guzzo F, Stocchero M, Zenoni S, Ferrarini A, et al.. Identification of putative stage-specific grapevine berry biomarkers and omics data integration into networks. *Plant Physiol*. 2010; doi: 10.1104/pp.110.160275.~~

~~51. Dal Santo S, Tornielli GB, Zenoni S, Fasoli M, Farina L, Anesi A, et al.. The plasticity of the grapevine berry transcriptome. *Genome Biol*. 2013; doi: 10.1186/gb-2013-14-6-r54.~~

~~52. Chitwood DH, Rundell SM, Li DY, Woodford QL, Yu TT, Lopez JR, et al.. Climate and Developmental Plasticity: Interannual Variability in Grapevine Leaf Morphology. *Plant Physiol*. 2016; doi: 10.1104/pp.15.01825.~~

~~53. Chitwood DH, Mullins J, Migicovsky Z, Frank M, VanBuren R, Londo JP. Vein-to-blade ratio is an allometric indicator of climate-induced changes in grapevine leaf size and shape. bioRxiv.~~

~~54. Gautier A, Cookson SJ, Lagalle L, Ollat N, Marguerit E. Influence of the three main genetic backgrounds of grapevine rootstocks on petiolar nutrient concentrations of the scion, with a focus on phosphorus. *OENO One*. 2020; doi: 10.20870/oeno-one.2020.54.1.2458.~~

~~55. Canas S, Assunção M, Brazão J, Zanol G, Eiras-Dias JE. Phenolic compounds involved in grafting incompatibility of Vitis spp: development and validation of an analytical method for their quantification. *Phytochem Anal*. 2015; doi: 10.1002/pca.2526.~~

Formatted: Header


56. Prodhomme D, Valls Fonayet J, Hévin C, Franc C, Hilbert G, de Revel G, et al.. Metabolite profiling during graft union formation reveals the reprogramming of primary metabolism and the induction of stilbene synthesis at the graft interface in grapevine. *BMC Plant Biol*. 2019; doi: 10.1186/s12870-019-2055-9.

57. Vitulo N, Forcato C, Carpinelli EC, Telatin A, Campagna D, D'Angelo M, et al.. A deep survey of alternative splicing in grape reveals changes in the splicing machinery related to tissue, stress condition and genotype. *BMC Plant Biol*. 2014; doi: 10.1186/1471-2229-14-99.

58. Harris ZN, Kovacs LG, Londo JP. RNA-seq-based genome annotation and identification of long-noncoding RNAs in the grapevine cultivar "Riesling." *BMC Genomics*. BioMed Central; 18:9372017;

59. Williams BR, Edwards CE, Kwasniewski MT, Miller AJ. Epigenomic patterns reflect irrigation and grafting in the grapevine clone'Chambourcin'. *bioRxiv*. Cold Spring Harbor Laboratory; 2020;

60. Marasco R, Rolli E, Fusi M, Michoud G, Daffonchio D. Grapevine rootstocks shape underground bacterial microbiome and networking but not potential functionality. *Microbiome*. 2018; doi: 10.1186/s40168-017-0391-2.

61. Swift JF, Hall ME, Harris ZN, Kwasniewski MT, Miller AJ. Grapevine microbiota reflect diversity among compartments and complex interactions within and among root and shoot systems. bioRxiv.

62. Palumbo MC, Zenoni S, Fasoli M, Massonnet M, Farina L, Castiglione F, et al.. Integrated network analysis identifies fight-club nodes as a class of hubs encompassing key putative switch genes that induce major transcriptome reprogramming during grapevine development. *Plant Cell*. Am Soc Plant Biol; 26:4617–352014;

63. Savoi S, Wong DCJ, Arapitsas P, Miculan M, Bucchetti B, Peterlunger E, et al.. Transcriptome and metabolite profiling reveals that prolonged drought modulates the phenylpropanoid and terpenoid pathway in white grapes (Vitis vinifera L.). *BMC Plant Biol*. 2016; doi: 10.1186/s12870-016-0760-1.

Formatted: Header


64. Savoi S, Wong DCJ, Degu A, Herrera JC, Bucchetti B, Peterlunger E, et al.. Multi-Omics and Integrated Network Analyses Reveal New Insights into the Systems Relationships between Metabolites, Structural Genes, and Transcriptional Regulators in Developing Grape Berries (Vitis vinifera L.) Exposed to Water Deficit. *Front Plant Sci*. 2017; doi: 10.3389/fpls.2017.01124.

65. Wong DCJ, Matus JT. Constructing Integrated Networks for Identifying New Secondary Metabolic Pathway Regulators in Grapevine: Recent Applications and Future Opportunities. *Front Plant Sci*. 2017; doi: 10.3389/fpls.2017.00505.

66. Fabres PJ, Collins C, Cavagnaro TR, Rodríguez López CM. A Concise Review on Multi-Omics Data Integration for Terroir Analysis in Vitis vinifera. *Front Plant Sci*. 2017; doi: 10.3389/fpls.2017.01065.

67. Huang S, Chaudhary K, Garmire LX. More Is Better: Recent Progress in Multi-Omics Data Integration Methods. *Front Genet*. 2017; doi: 10.3389/fgene.2017.00084.

68. Stein-O'Brien GL, Arora R, Culhane AC, Favorov AV, Garmire LX, Greene CS, et al.. Enter the Matrix: Factorization Uncovers Knowledge from Omics. *Trends Genet*. 2018; doi: 10.1016/j.tig.2018.07.003.

69. Fox J, Friendly M, Weisberg S. Hypothesis tests for multivariate linear models using the car package. *R J*. Citeseer; 5:39–522013;

70. R Core Team. R: A language and environment for statistical computing. Vienna, Austria;

71. Lenth R, Singmann H, Love J, Others. Emmeans: Estimated marginal means, aka least-squares means. *R package version*. 12018;

72. Ripley BD. Modern applied statistics with S. Springer;

73. Wickham H. ggplot2: Elegant Graphics for Data Analysis. Springer;

Formatted: Header


74. Liaw A, Wiener M, Others. Classification and regression by randomForest. *R news*. 2:18–222002;

75. Kuhn M. Predictive Modeling with R and the caret Package. *Google Scholar*. 2013;

76. Team RC, Others. R foundation for statistical computing. *Vienna, Austria*. 32013;

77. Csardi G, Nepusz T, Others. The igraph software package for complex network research. *InterJournal, complex systems*. 1695:1–92006;

19. Migicovsky Z, Harris ZN, Klein LL, Li M, McDermaid A, Chitwood DH, et al.. Rootstock effects on scion phenotypes in a "Chambourcin" experimental vineyard. *Horticulture Research*. Nature Publishing Group; 6:1–132019;

20. Galet P. A Practical Ampelography: Grapevine Identification. Comstock Pub. Associates;

21. Mullins MG, Bouquet A, Williams LE. Biology of the Grapevine. Cambridge University Press;

22. Chitwood DH, Ranjan A, Martinez CC, Headland LR, Thiem T, Kumar R, et al.. A modern ampelography: a genetic basis for leaf shape and venation patterning in grape. *Plant Physiol*. 164:259–722014;

23. Chitwood DH, Klein LL, O'Hanlon R, Chacko S, Greg M, Kitchen C, et al.. Latent developmental and evolutionary shapes embedded within the grapevine leaf. New Phytologist.

24. Klein LL, Caito M, Chapnick C, Kitchen C, O'Hanlon R, Chitwood DH, et al.. Digital Morphometrics of Two North American Grapevines (Vitis: Vitaceae) Quantifies Leaf Variation between Species, within Species, and among Individuals. *Front Plant Sci*. 8:3732017;

25. Grimes DW, Williams LE. Irrigation Effects on Plant Water Relations and Productivity of Thompson Seedless Grapevines. *Crop Sci*. 30:2551990;

26. Williams LE, Grimes DW. Modelling vine growth-development of a data set for a water balance subroutine. *Proceedings of the Sixth Australian Wine Industry Technical Conference*. p. 169–74.

27. Gautier A, Cookson SJ, Lagalle L, Ollat N, Marguerit E. Influence of the three main genetic backgrounds of grapevine rootstocks on petiolar nutrient concentrations of the scion, with a focus on phosphorus. *OENO One*. 54:1–132020;

28. Lecourt J, Lauvergeat V, Ollat N, Vivin P, Cookson SJ. Shoot and root ionome responses to nitrate supply in grafted grapevines are rootstock genotype dependent: Rootstock and nitrogen supply affect grapevine ionome. *Aust J Grape Wine Res*. 21:311–82015;

29. Salt DE, Baxter I, Lahner B. Ionomics and the study of the plant ionome. *Annu Rev Plant Biol*. 59:709–332008;

30. Baxter I. Ionomics: The functional genomics of elements. *Brief Funct Genomics*. 9:149–562010;

31. Ziegler G, Terauchi A, Becker A, Armstrong P, Hudson K, Baxter I. Ionomic Screening of Field-

Formatted: Header


Grown Soybean Identifies Mutants with Altered Seed Elemental Composition. The Plant Genome.

32. Oliver SG, Winson MK, Kell DB, Baganz F. Systematic functional analysis of the yeast genome. *Trends Biotechnol*. 16:373–81998;

33. Tweeddale H, Notley-McRobb L, Ferenci T. Effect of slow growth on metabolism of Escherichia coli, as revealed by global metabolite pool (“metabolome”) analysis. *J Bacteriol*. 180:5109–161998;

34. Islam MN, Downey F, Ng CKY. Comparative analysis of bioactive phytochemicals from Scutellaria baicalensis, Scutellaria lateriflora, Scutellaria racemosa, Scutellaria tomentosa and Scutellaria wrightii by LC-DAD-MS. *Metabolomics*. Springer; 7:446–532011;

35. Tautenhahn R, Patti GJ, Rinehart D, Siuzdak G. XCMS Online: a web-based platform to process untargeted metabolomic data. *Anal Chem*. 84:5035–92012;

36. Tandonnet S, Torres TT. Traditional versus 3’ RNA-seq in a non-model species. *Genom Data*. 11:9–162017;

37. Bolger AM, Lohse M, Usadel B. Trimmomatic: a flexible trimmer for Illumina sequence data. *Bioinformatics*. 30:2114–202014;

38. Bushnell B. BBTools software package. *URL http://sourceforge net/projects/bbmap*. 2017;

39. Jaillon O, Aury J-M, Noel B, Policriti A, Clepet C, Casagrande A, et al.. The grapevine genome sequence suggests ancestral hexaploidization in major angiosperm phyla. *Nature*. 449:463–72007;

40. Canaguier A, Grimplet J, Di Gaspero G, Scalabrin S, Duchêne E, Choisne N, et al.. A new version of the grapevine reference genome assembly (12X.v2) and of its annotation (VCost.v3). *Genom Data*. 14:56–622017;

41. Dobin A, Davis CA, Schlesinger F, Drenkow J, Zaleski C, Jha S, et al.. STAR: ultrafast universal RNA-seq aligner. *Bioinformatics*. 29:15–212013;

42. Anders S, Pyl PT, Huber W. HTSeq: Analysing high-throughput sequencing data with Python.

43. Love MI, Huber W, Anders S. Moderated estimation of fold change and dispersion for RNA-seq data with DESeq2. *Genome Biol*. 15:5502014;

44. Carbonell-Bejerano P, Rodríguez V, Royo C, Hernáiz S, Moro-González LC, Torres-Viñals M, et al.. Circadian oscillatory transcriptional programs in grapevine ripening fruits. *BMC Plant Biol*. 14:782014;

45. Anders S, Huber W. Differential expression analysis for sequence count data. *Genome Biol*. 11:R1062010;

46. Dryden IL, Mardia KV. Statistical Shape Analysis: With Applications in R. John Wiley & Sons;

47. Shackel K, Gross R. Using midday stem water potential to assess irrigation needs of landscape valley oaks. 2002;

48. Levin AD. Re-evaluating pressure chamber methods of water status determination in field-grown grapevine (Vitis spp.). *Agric Water Manag*. Elsevier BV; 221:422–92019;

49. Liang W, Zou X, Carballar-Lejarazú R, Wu L, Sun W, Yuan X, et al.. Selection and evaluation of

Formatted: Header

reference genes for qRT-PCR analysis in Euscaphis konishii Hayata based on transcriptome data. *Plant Methods*. Springer Science and Business Media LLC; 2018; doi: 10.1186/s13007-018-0311-x.

50. Podani J, Miklós I. Resemblance coefficients and the horseshoe effect in principal coordinates analysis. *Ecology*. Wiley; 83:3331–432002;

51. Degu A, Hochberg U, Sikron N, Venturini L, Buson G, Ghan R, et al.. Metabolite and transcript profiling of berry skin during fruit development elucidates differential regulation between Cabernet Sauvignon and Shiraz cultivars at branching points in the polyphenol pathway. *BMC Plant Biol*. 14:1882014;

52. Anesi A, Stocchero M, Dal Santo S, Commisso M, Zenoni S, Ceoldo S, et al.. Towards a scientific interpretation of the terroir concept: plasticity of the grape berry metabolome. *BMC Plant Biol*. 15:1912015;

53. Cuadros-Inostroza A, Ruíz-Lara S, González E, Eckardt A, Willmitzer L, Peña-Cortés H. GC-MS metabolic profiling of Cabernet Sauvignon and Merlot cultivars during grapevine berry development and network analysis reveals a stage- and cultivar-dependent connectivity of primary metabolites. *Metabolomics*. 12:392016;

54. Dal Santo S, Fasoli M, Negri S, D'Incà E, Vicenzi N, Guzzo F, et al.. Plasticity of the Berry Ripening Program in a White Grape Variety. *Front Plant Sci*. 7:9702016;

55. Zamboni A, Di Carli M, Guzzo F, Stocchero M, Zenoni S, Ferrarini A, et al.. Identification of putative stage-specific grapevine berry biomarkers and omics data integration into networks. *Plant Physiol*. 154:1439–592010;

56. Dal Santo S, Tornielli GB, Zenoni S, Fasoli M, Farina L, Anesi A, et al.. The plasticity of the grapevine berry transcriptome. *Genome Biol*. 14:r542013;

57. Chitwood DH, Rundell SM, Li DY, Woodford QL, Yu TT, Lopez JR, et al.. Climate and Developmental Plasticity: Interannual Variability in Grapevine Leaf Morphology. *Plant Physiol*. 170:1480–912016;

58. Chitwood DH, Mullins J, Migicovsky Z, Frank M, VanBuren R, Londo JP. Vein-to-blade ratio is an allometric indicator of climate-induced changes in grapevine leaf size and shape. bioRxiv.

59. Gautier A, Cookson SJ, Lagalle L, Ollat N, Marguerit E. Influence of the three main genetic backgrounds of grapevine rootstocks on petiolar nutrient concentrations of the scion, with a focus on phosphorus. *OENO One*. 54:1–132020;

60. Bravdo B. EFFECT OF MINERAL NUTRITION AND SALINITY ON GRAPE PRODUCTION AND WINE QUALITY. *Acta Horticulturae*. 512:23–302000;

61. Brunetto G, Melo GWBDE, Toselli M, Quartieri M, Tagliavini M. The role of mineral nutrition on yields and fruit quality in grapevine, pear and apple. *Rev Bras Frutic*. FapUNIFESP (SciELO); 37:1089–1042015;

62. Gautier A, Cookson SJ, Hevin C, Vivin P, Lauvergeat V, Mollier A. Phosphorus acquisition efficiency and phosphorus remobilization mediate genotype-specific differences in shoot phosphorus content in grapevine. *Tree Physiol*. Oxford University Press (OUP); 38:1742–512018;

63. Canas S, Assunção M, Brazão J, Zanol G, Eiras-Dias JE. Phenolic compounds involved in grafting

**Formatted:** Header


incompatibility of Vitis spp: development and validation of an analytical method for their quantification. *Phytochem Anal*. 26:1–72015;

64. Prodhomme D, Valls Fonayet J, Hévin C, Franc C, Hilbert G, de Revel G, et al.. Metabolite profiling during graft union formation reveals the reprogramming of primary metabolism and the induction of stilbene synthesis at the graft interface in grapevine. *BMC Plant Biol*. 19:5992019;

65. Migicovsky Z, Sawler J, Money D, Eibach R, Miller AJ, Luby JJ, et al.. Genomic ancestry estimation quantifies use of wild species in grape breeding. *BMC Genomics*. 17:4782016;

66. Vitulo N, Forcato C, Carpinelli EC, Telatin A, Campagna D, D'Angelo M, et al.. A deep survey of alternative splicing in grape reveals changes in the splicing machinery related to tissue, stress condition and genotype. *BMC Plant Biol*. 14:992014;

67. Harris ZN, Kovacs LG, Londo JP. RNA-seq-based genome annotation and identification of long-noncoding RNAs in the grapevine cultivar "Riesling." *BMC Genomics*. BioMed Central; 18:9372017;

68. Williams BR, Edwards CE, Kwasniewski MT, Miller AJ. Epigenomic patterns reflect irrigation and grafting in the grapevine clone'Chambourcin'. *bioRxiv*. Cold Spring Harbor Laboratory; 2020;

69. Marasco R, Rolli E, Fusi M, Michoud G, Daffonchio D. Grapevine rootstocks shape underground bacterial microbiome and networking but not potential functionality. *Microbiome*. 6:32018;

70. Swift JF, Hall ME, Harris ZN, Kwasniewski MT, Miller AJ. Grapevine microbiota reflect diversity among compartments and complex interactions within and among root and shoot systems. bioRxiv.

71. Palumbo MC, Zenoni S, Fasoli M, Massonnet M, Farina L, Castiglione F, et al.. Integrated network analysis identifies fight-club nodes as a class of hubs encompassing key putative switch genes that induce major transcriptome reprogramming during grapevine development. *Plant Cell*. Am Soc Plant Biol; 26:4617–352014;

72. Savoi S, Wong DCJ, Arapitsas P, Miculan M, Bucchetti B, Peterlunger E, et al.. Transcriptome and metabolite profiling reveals that prolonged drought modulates the phenylpropanoid and terpenoid pathway in white grapes (Vitis vinifera L.). *BMC Plant Biol*. 16:672016;

73. Savoi S, Wong DCJ, Degu A, Herrera JC, Bucchetti B, Peterlunger E, et al.. Multi-Omics and Integrated Network Analyses Reveal New Insights into the Systems Relationships between Metabolites, Structural Genes, and Transcriptional Regulators in Developing Grape Berries (Vitis vinifera L.) Exposed to Water Deficit. *Front Plant Sci*. 8:11242017;

74. Wong DCJ, Matus JT. Constructing Integrated Networks for Identifying New Secondary Metabolic Pathway Regulators in Grapevine: Recent Applications and Future Opportunities. *Front Plant Sci*. 8:5052017;

75. Fabres PJ, Collins C, Cavagnaro TR, Rodríguez López CM. A Concise Review on Multi-Omics Data Integration for Terroir Analysis in Vitis vinifera. *Front Plant Sci*. 8:10652017;

76. Huang S, Chaudhary K, Garmire LX. More Is Better: Recent Progress in Multi-Omics Data Integration Methods. *Front Genet*. 8:842017;

77. Stein-O'Brien GL, Arora R, Culhane AC, Favorov AV, Garmire LX, Greene CS, et al.. Enter the Matrix: Factorization Uncovers Knowledge from Omics. *Trends Genet*. 34:790–8052018;

78. Fox J, Friendly M, Weisberg S. Hypothesis tests for multivariate linear models using the car package. *R J*. Citeseer; 5:39–522013;

79. R Core Team. R: A language and environment for statistical computing. Vienna, Austria;

80. Lenth R, Singmann H, Love J, Others. Emmeans: Estimated marginal means, aka least-squares means. *R package version*. 12018;

81. Ripley BD. Modern applied statistics with S. Springer;

82. Wickham H. ggplot2: Elegant Graphics for Data Analysis. Springer;

83. Liaw A, Wiener M, Others. Classification and regression by randomForest. *R news*. 2:18–222002;

84. Kuhn M. Predictive Modeling with R and the caret Package. *Google Scholar*. 2013;

85. Team RC, Others. R foundation for statistical computing. *Vienna, Austria*. 32013;

86. Csardi G, Nepusz T, Others. The igraph software package for complex network research. *InterJournal, complex systems*. 1695:1–92006;

**Formatted:** Header

Figure 1

Click here to access/download;Figure;Figure 1.pdf

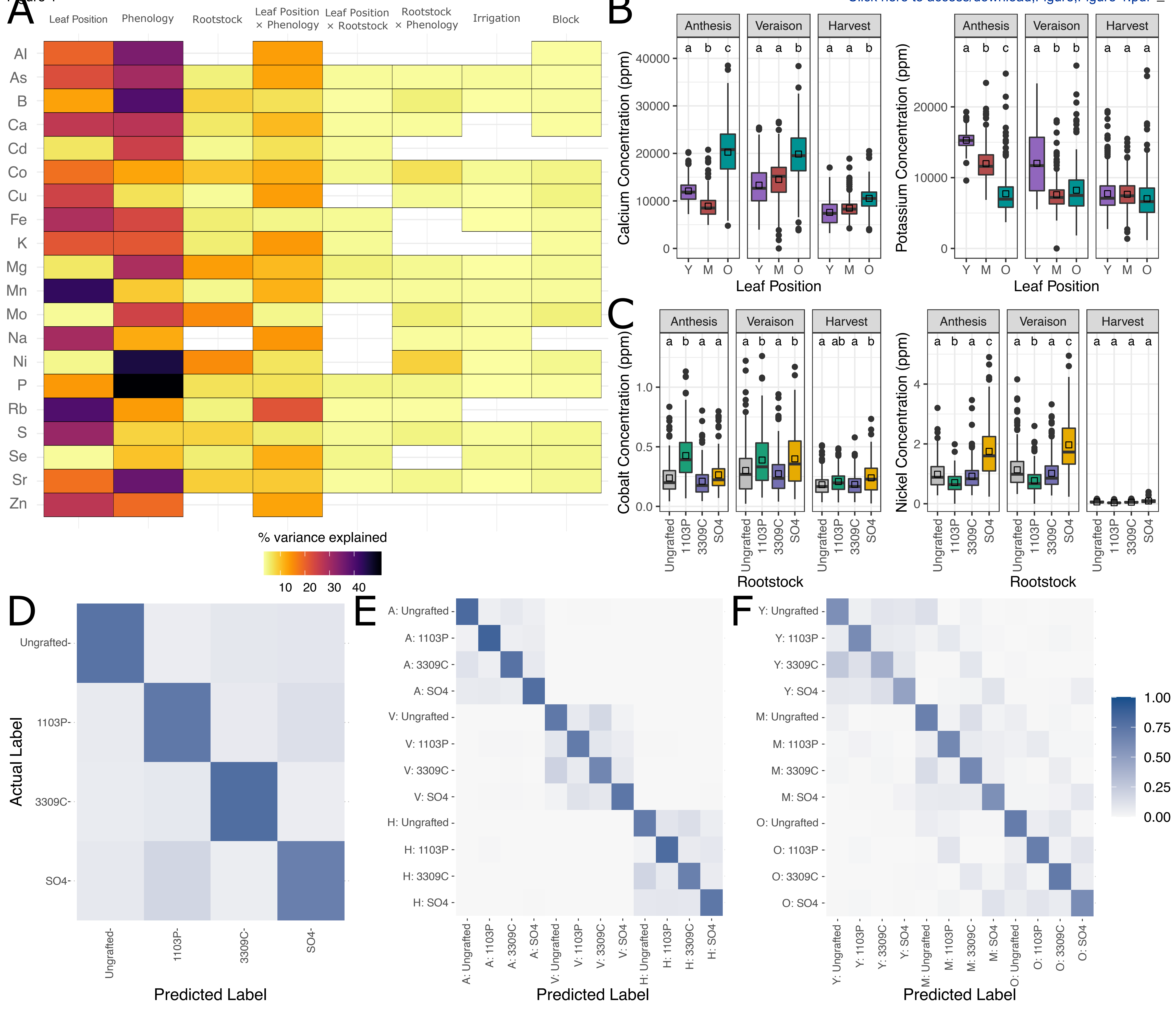

Figure 2

Click here to access/download;Figure;Figure 2.pdf

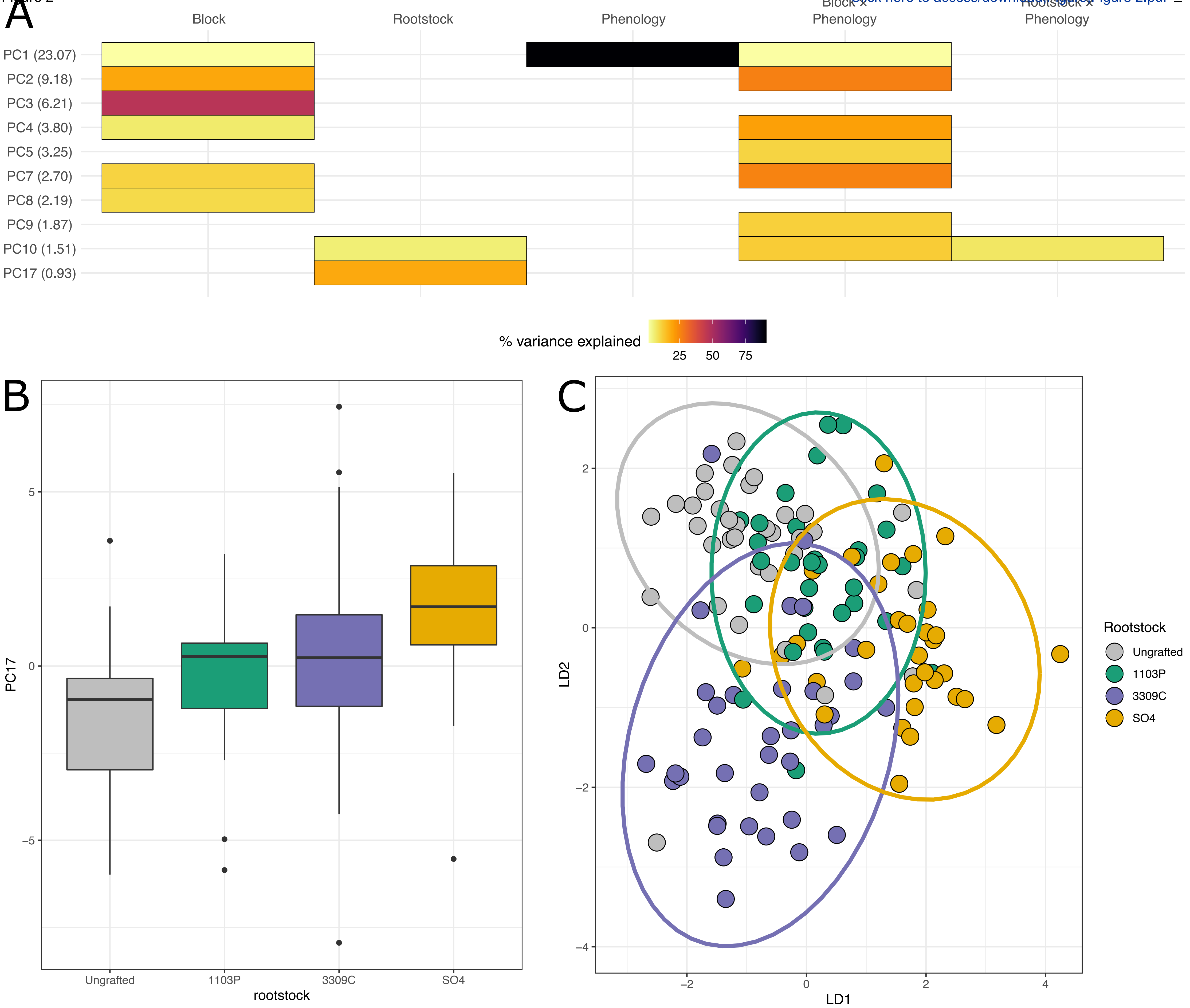

Figure 3

Click here to access/download;Figure;Figure 3.pdf

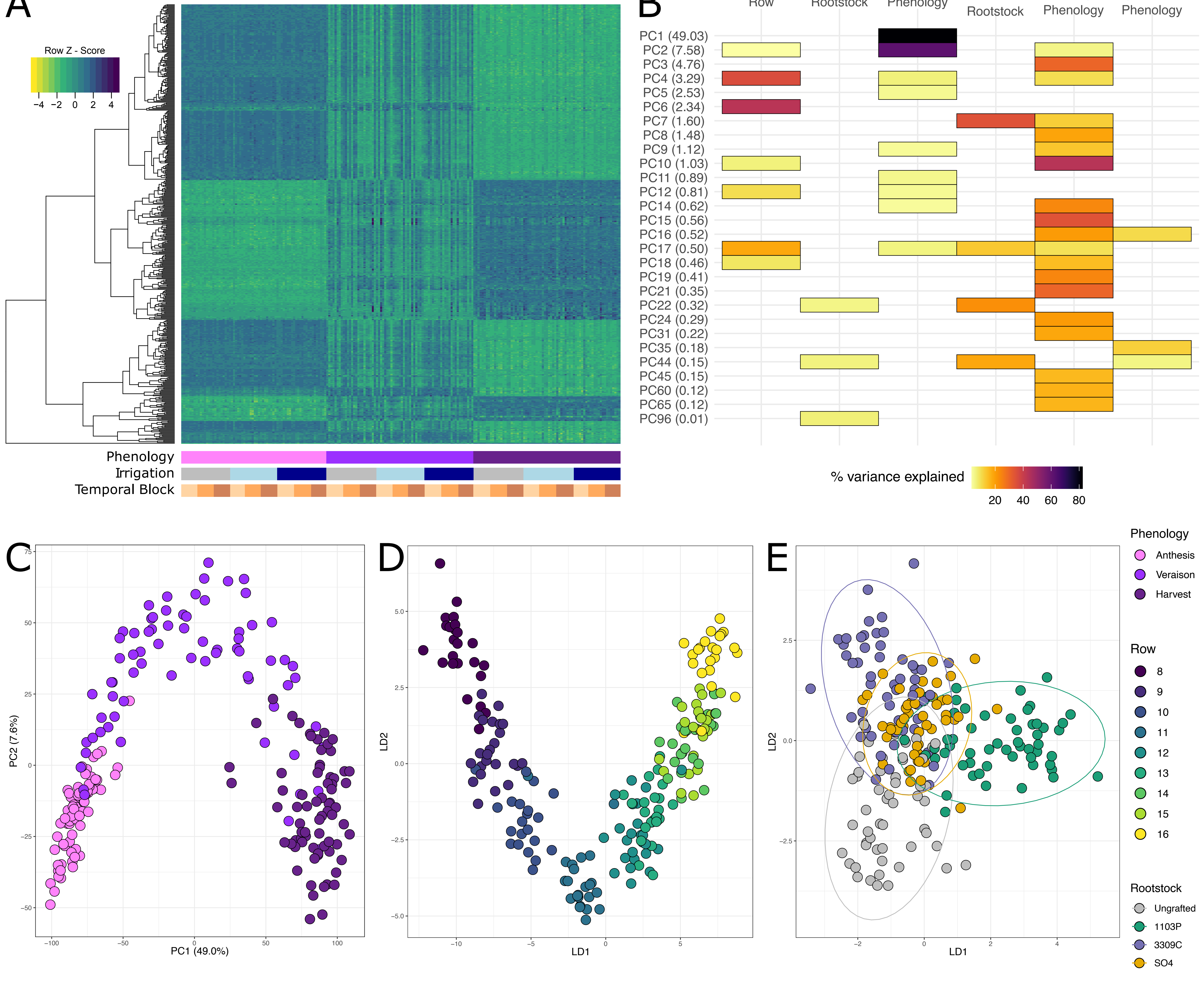

Figure 4

Click here to access/download;Figure;Figure 4.pdf

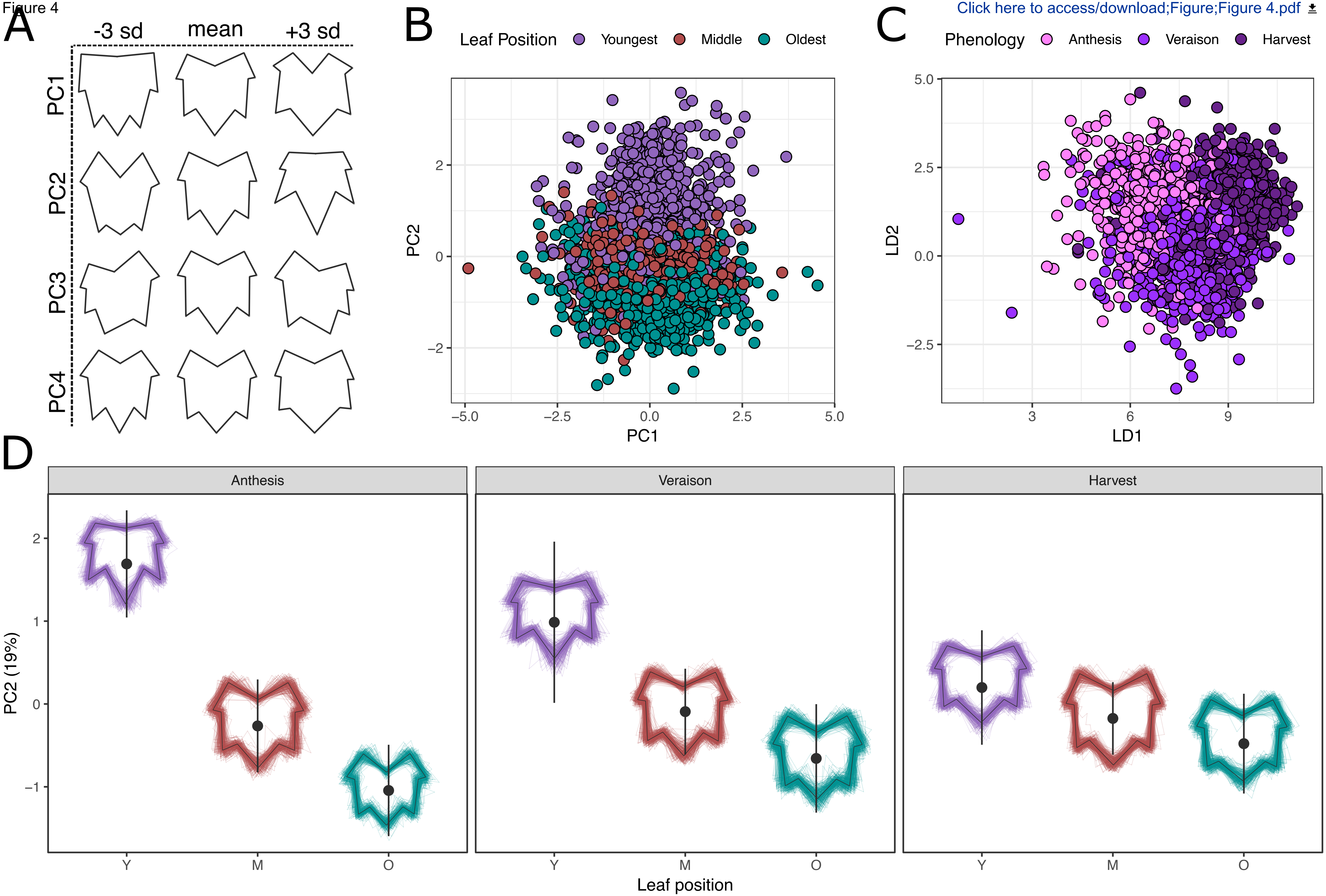

Figure 5

Click here to access/download;Figure;Figure 5.pdf

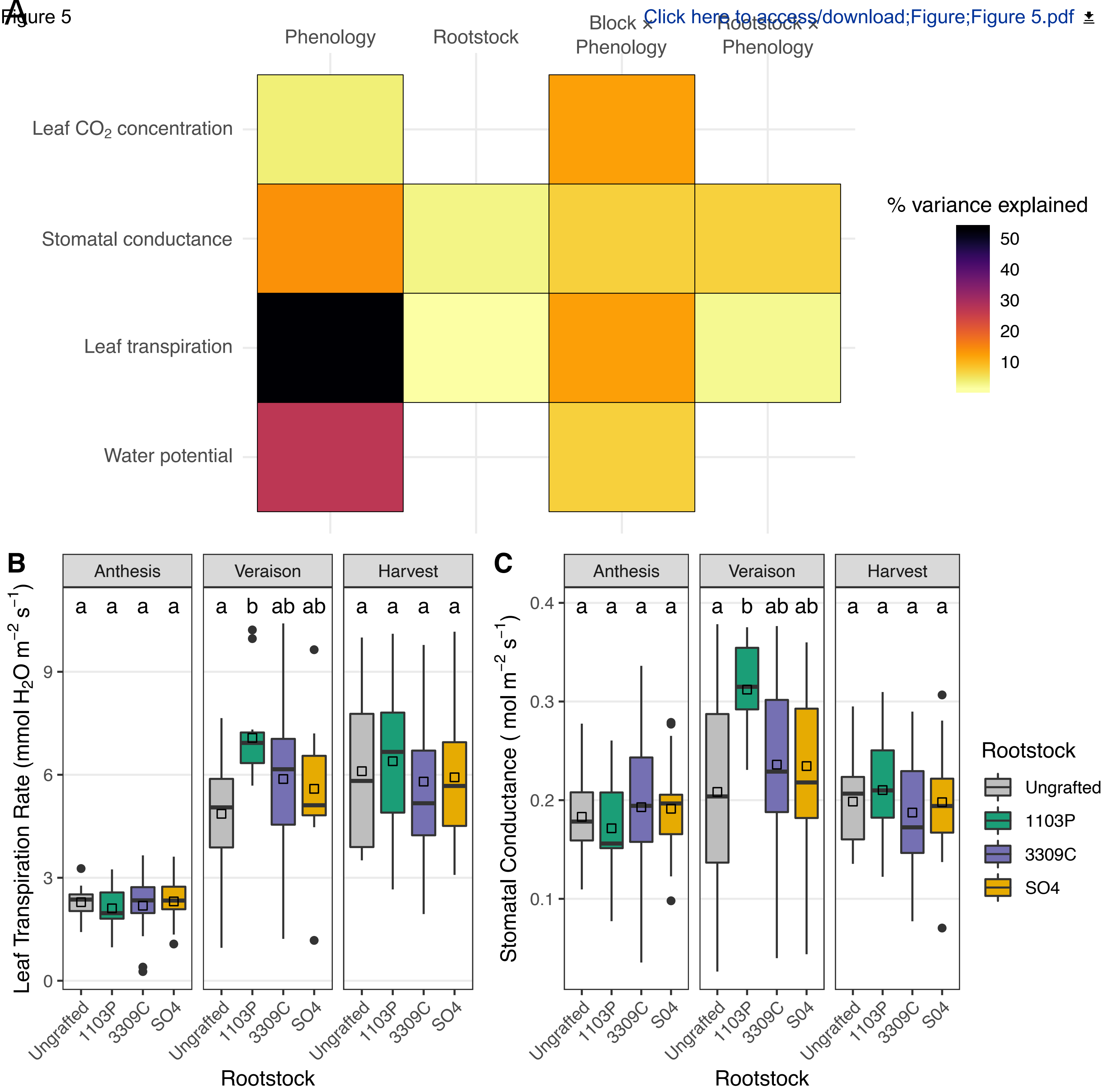

Figure 6

Click here to access/download;Figure;Figure 6.pdf

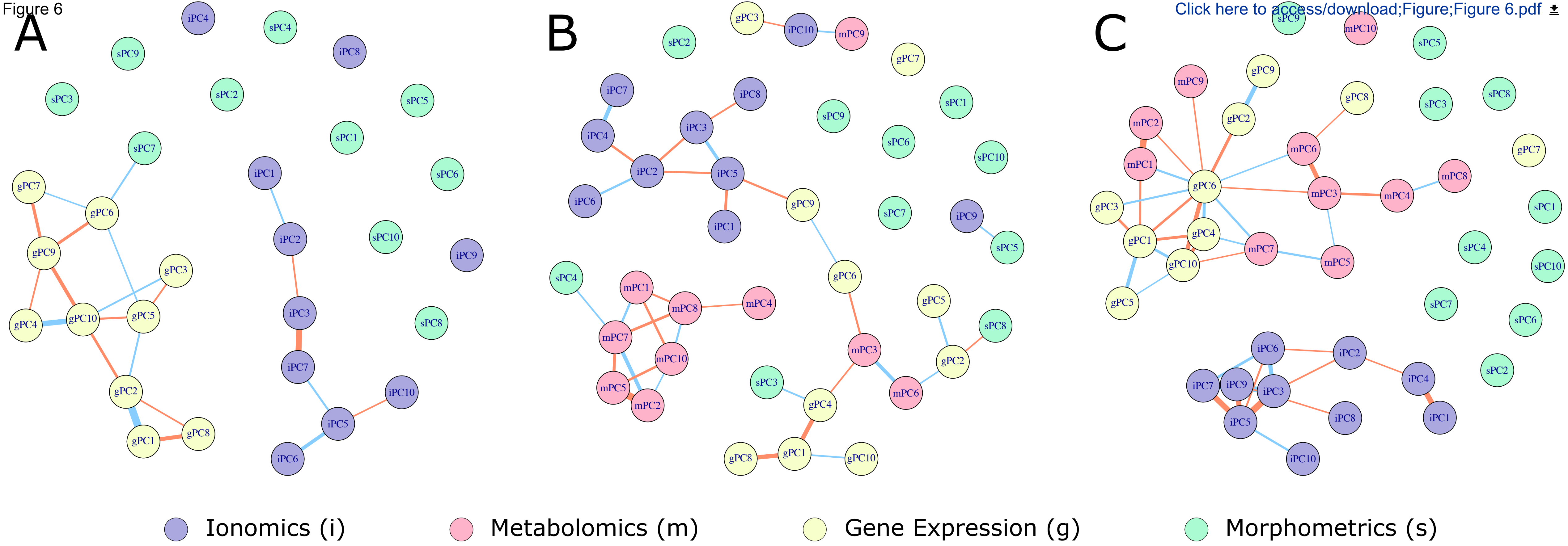

Supplemental Figure 1

Click here to access/download
**Supplementary Material**
Supplemental Figure 1.pdf

Click here to access/download
**Supplementary Material**
Supplemental Figure 2.pdf

Supplemental Figure 3

Click here to access/download
**Supplementary Material**
Supplemental Figure 3.pdf

Supplemental Figure 4

Click here to access/download
**Supplementary Material**
Supplemental Figure 4.pdf

Supplemental Figure 5

Click here to access/download
**Supplementary Material**
Supplemental Figure 5.pdf

Supplemental Figure 6

Click here to access/download
**Supplementary Material**
Supplemental Figure 6.pdf

Supplemental Figure 7

Click here to access/download
**Supplementary Material**
Supplemental Figure 7.pdf

Click here to access/download
**Supplementary Material**
Supplemental Note 1.pdf

Click here to access/download;Personal Cover;MV2017Leaves_GigaScience_revisionCoverLetter.docx

Dr. Nicole Nogoy,

We are happy to submit our revised manuscript (GIGA-D-21-00137) for review. We would like to start by thanking the reviewers for their robust, extremely careful and thorough consideration of our manuscript. The suggestions made by the reviewers were invaluable, and addressing them has led to a stronger manuscript and a more complete and reproducible analysis.

The largest changes to the manuscript address the issues of validation of RNAseq data, more detailed descriptions of RNAseq methods, changes to language for the sake of clarity, and a discussion on the limitations of our work. Every change made to the manuscript is addressed and summarized in the attached (and pasted below) point-by-point response to the reviewers.

In addition to the changes made at the request of the reviewers, we made changes to the manuscript based on editor comments. Primarily, we agree with your summary on the appropriateness of data placement. As such, we have moved a supplemental note on this manuscript from GitHub to a Supplemental Note in the manuscript. Following previous correspondence, we are in the process of uploading the metabolomics data to Metabolights. There is currently an extended waiting period for review of Metabolights submissions, but we have been given a temporary accession (MTBLS2831), and we will update you when this review is complete.

We deeply appreciate the time that everyone has spent on this manuscript, and we look forward to hearing back soon.

Best,
Zachary N Harris and Allison J Miller

Black Text  = Reviewer Comments
Grey Text = Author Response

Note to all: Microsoft Word on macOS does not allow correct continuous line numbering with “track changes” on. All referenced line numbers were identified such that they were continuous. If line numbers appear way off, try changing “All Markup” to “Simple Markup” under the Review tab to align the line numbers.

## Editor Comments

Overall, the reviewers have highlighted that in its current form, the manuscript requires more biological validation and more detailed methods to ensure reproducibility of the work presented. Limitations on the study should be discussed and how they may impact the results; and more data should be provided to understand the reliability of the RNA-seq experiment.  Furthermore, RNA-seq methods are incomplete - we strongly encourage all authors to add their detailed methods to protocols.io (if not already open in protocols.io and cite the protocol DOI in the paper.

- Response: Thank you for this. We have added all details that have been requested on L191-195. In addition, we added a supplemental figure (Supplemental Figure 2) showcasing our analysis for gene expression validity. A summary of this analysis is on L207-210.

We also see reviewer #4 suggests to use Figshare - but this is not an appropriate database to share large-scale data, such as this work presents. Github is still the most appropriate place to share scripts and associated documentation, and our open repository, GigaDB can host the other metadata not already open in other community approved repositories; we will also host snapshots of your scripts in GitHub.

- Response: In addressing Reviewer #4’s specific recommendation, we have moved the document in question from GitHub to a Supplementary Note in the manuscript. In line with previous communications, we are also in the curation process of metabolomics data uploaded to Metabolights. We are happy to jointly upload other data sets to preferred repositories and databases, we just need guidance on the preferred locations for those data. As of today, we are unaware of standard databases for ionomics and leaf shape data. Scripts used for analysis are still available on GitHub, but can be hosted elsewhere if this is of concern.

## Reviewer #1

The manuscript by Harris and co-workers presents a characterization of rootstock genotype effects on multilevel leaf phenotypes of one grafted grapevine scion cultivar. Three rootstock genotypes along with the ungrafted cultivar were compared for ionomics, transcriptomics, metabolomics, leaf morphology and physiology in three phenological stages. Analytical and statistical analyses applied were generally sound. While authors identify larger effects in most

cases for stage and vineyard position/sampling time, ionic composition was the phenotype most significantly affected by rootstock genotype. Co-variance among multilevel phenotypes is also presented.

## Major issues

1. The study comprises a vast dataset, with a total of 288 plants independently analyzed for two phenotypes (morphometry and ionomics) and 72 plants were used for the rest of phenotypes. Nevertheless, the experiment is limited in terms of genotypes tested and reproducibility. Only one year of study and under the specific soil and climate conditions of a single field plot. Moreover, the effects were only tested on a single scion genotype, a bred interspecific hybrid including Vitis riparia and V. rupestris in its pedigree. At least one of these species is also in the pedigree of the three rootstocks tested, which might involve lower diversity than in common interactions between rootstocks and V. vinifera cultivars. These limitations should at least be considered when discussing the results.

- Response: The comments provided by the reviewer are all excellent observations that were clearly missing from the discussion of our work. We have added a paragraph to the Discussion (L544 - L559) to better couch how our work should be compared to other studies and the considerations that may account for those differences.

2. The RNA-seq assay did not identify differentially expressed genes (DEGs) in response to rootstock genotype, which disagrees with previous reports. While the origin of the lack of effects here is unclear, further data should be provided to understand the reliability of the RNA-seq experiment:

- Response: We thank the reviewer for this comment. We were similarly interested to see that our results differ from previously published analyses in similar systems. We added comments to the discussion to clarify why we think these differences might be present. To ensure such reliability, we confirmed in our data that previously established patterns of house-keeping and circadian phased genes were behaving as expected. The following has been added to the manuscript:
    - Added to data description L208-210, “To check the validity of our expression results, we assayed two classes of housekeeping gene (Ubiquitin-domain and actin-family) and eight previously annotated circadian genes (Carbonell-Bejerano et al. 2014)”
    - Added to Analyses, L339 - 345, “We computed the expression of two classes of housekeeping genes, and showed that they are generally stable across samples over phenological time (Supplemental Figure 2). We noted that some variation is expected for housekeeping genes; see, for example, [49]. Moreover, we showed that patterns of previously annotated circadian genes conform to expected results over the sampling window. For example, predicted orthologs of LHY and RVE1 are correlated and decreasing over our sampling window, and a predicted TOC1 ortholog is invariant. The results of these analyses provide general confidence in the gene expression data presented here.“
    - Created and added Supplemental Figure (now supplemental Figure 2) showing these patterns.

2.1. Which was the timing of sample collection for RNA-seq samples? Was the same sampling order followed for the three phenological stages? Which were the weather conditions on each of the three sampling dates? It is relevant to describe that information since environmental and circadian changes between and within days can alter gene expression.

- Response: We added the following to the section describing the gene expression data set (L187-190): “Leaves were sampled by a single team near midday between 10AM and 2PM in row order ensuring that ‘block’ and ‘row’ accounted for unmeasured environmental variation and temporal variation over the sampling window.” A statement was added on L637-638 that “At each phenological stage, effort was made to sample on days with full to partial sun and minimal precipitation.“

2.2. Data on RNA sequencing depth should be provided to understand the resolution of the transcriptomics experiment. For instance, how many bases/reads per sample were produced? How many genes per sample were called as expressed (DESeq2-norm counts >2 according to authors own threshold)?

- Response: Information on sequencing depth and genes per sample were added to the Analyses section. Specifically, we added the following to L337-338: “On average, each sample contained 4.1 million 3’-reads and showed the expression of 17,852 genes.”

3. The interpretation of the origin of the results is generally shallow and several questions or limitations are overlooked. For instance:

3.1. It is described that physiological parameters were measured from 10 am to 1 pm, a wide interval with expected changes in environmental conditions affecting these measurements. To understand for possible covariances, it should be indicated if these measurements were carried out simultaneously and following the same order than that of leaf sample collection for the other phenotyping.

- Response: We agree that we missed crucial details about the timing of this sampling. To fix this, we included the clarifications that (on L241) all physiology measurements were being taken simultaneously by different groups moving though the vineyard and (on L243-245) the measurements were all taken in row order ensuring that the vineyard blocking factor captured temporal variation. SImilar notes were added for the other phenotypes to better explain sampling. As was noted in the next reviewer comment, block is missing from Figure 5 which means it was not significant as a main effect.

3.2. Related to the previous, why block effect alone was not considered for physiological measurements in Figure 5?

- Response: Thank you for this comment. Block (or row for gene expression) was included in the models as a main effect for every modality (see analysis section). Non-significant factors were dropped from figures summarizing linear models; because the block main effect was not significant, it was not included in Figure 5. .

3.3. Did the horseshoe shape for row effect on the transcriptome correlate with oscillation of environmental/circadian clock conditions during the sampling interval or with vineyard

heterogeneity? Functional analysis of the genes contributing to row effect could be informative on the origin of these effects that might have hindered the identification of rootstock effect on the transcriptome.

- Response: This is a really interesting comment. We agree with the reviewer that the horseshoe shape in LDA space is either a function of circadian conditions or spatial heterogeneity. We have added to the Data Description section a comment on assaying genes with known circadian topology (L207-210) and show in Supplemental Figure 2 that those genes are variable over our sampling window. In addition, we commented on this outcome in the Analyses section on L360-362. We show that the impact of vineyard position/spatial variation is weak in other measured phenotypes (captured by the 'block' model term; see, for example, Figure 1A and Supplemental Figure 5A). Future studies should assess potential intra-vineyard variation either through blocking (as we did here) or explicit measurement (for example, soil composition) and control for that variation.

3.4. Is there a rootstock effect on vigor, biomass, fruit fertility and production that could explain or condition the effects in leaf phenotypes that were measured? Were these factors normalized in any way, either by agronomic practices or statistical treatment?

- Response: This is an excellent question that is perhaps beyond the scope of this comprehensive analysis of leaf phenotypes, but one that is certainly an important next step in our research trajectory. Conditioning or normalizing on aspects of vigor or yield or looking for correlates of those traits in early season leaf phenotypes would be immensely valuable to viticulture and a general understanding of grapevine biology. To explore this idea, we have amended the language of the Potential Implications section on L588-608. In addition, some of these data were collected and are being prepared for papers focused toward berry phenotypes. In the meantime, we point to (https://doi.org/10.1002/pld3.324) to show that this is absolutely a valid direction of inquiry for future work and data integration efforts.

4. This study comprises similar experiments to these already published by the same group in the same set of plants (Migicovsky et al., Hort Res 2019), although extended to include metabolomics and physiology data and two additional phenologcal stages. While the effect of phenology is clearly presented here, the addition of the metabolite data is undermined. What are the metabolites determining rootstock effect in Figure 2C? What about metabolites determining a rootstock effect depending on phenology that could be inferred from PC10?

- Response: We thank the reviewer for this comment and appreciate the careful consideration of this manuscript in the context of the Migicovsky et al, 2019 manuscript. We completely agree that the value of the metabolomics data is undermined in the manuscript. This is primarily the result of current challenges in mapping peaks from LC-MS onto named metabolites. The current state of untargeted metabolomics from LC-MS would require significant chemical laboratory work to narrow down the space of potential metabolites. While we believe this work should absolutely be done, our goal with this study was not necessarily to identify specific metabolites but to determine if the metabolome was a potential avenue through which the rootstock is influencing scion phenotypes. To address this, we used only a portion of the runs available to show there

is a signal. Future work will focus on merging the various additional LC-MS runs (not presented here) and chemical experimentation to uncover the full scope of this effect. We note that we are uploaded raw data to Metabolights, QC/filtered data to FigShare, and reported the retention times and m/z ratios for the compounds of putative interest in the manuscript. We hope that these data may be useful in future analyses of grapevine metabolites, either by our group or others.

## Minor revisions

1. "Ubiquitous" effects of rootstock genotype are described along the MS. However, since only one location was analyzed (leaves), would "pleotropic" be more appropriate to define the different phenotypes affected by rootstock-scion genotype interaction in this study?

- Response: This is a great point. We clarified our usage of the word "ubiquitous" to ensure its intended meaning (across modalities within leaves, not as an inherent feature of grapevine) was clear (for example, on L514) throughout the manuscript. Supporting the reviewer's observation that this study only included one environment, we are hesitant to use the word "pleiotropy", though we are interested in the implication that the different rootstock/scion pairs create different local environments and will consider this idea in future works.

2. Methods on RNA-seq procedures are incomplete. Which sequencing technology was used? Which type and length of reads? Etc.

- Response: Good catch. We added the following to the L194-195: "Sequencing was conducted using the Illumina NextSeq500 platform which returned single-end 86 bp reads."

3. Inter-annual comparison for anthesis ionomics, transcriptomics and morphology between this study and their previous publication (Migicovsky et al., Hort Res 2019) could enable a broader interpretation of rootstock effects, overcoming the reproducibility limitation of considering only a single season here.

- Response: We absolutely agree that interannual analyses are required for a detailed understanding of the root system influence on shoot phenotypes and these analyses are underway. Our goal with this manuscript was to carefully quantify different phenotyping modalities and to understand how they relate to one another. The results from this study have helped us consider what is worth more detailed investigations, and analyses that address longer (multi-year) studies for those phenotypes are currently in the works. Given the magnitude of the data presented here and the extent of analyses conducted, we struggled to fit this detailed work in a single manuscript that also covered inter-annual variation as well as additional phenotypes (berry chemistry, etc.). As a result of work presented here, we are currently exploring tradeoffs between deep analyses of individual phenotypes and shallower analyses of more modalities over longer time periods, additional scions, and multiple sites. In the meantime, wherever possible we note some comparisons to the Migicovsky 2019 study where appropriate. The Migicovsky 2019 pilot

study used considerably different methods for many phenotypes, which preclude direct comparisons.

4. L426. The sentence might not be completely fair as no DEG was identified for rootstock effect (transcriptome phenotype would therefore be mostly unaffected) and developmental stage-specific could be more adequate than season-specific.

- Response: Thank you for this. We agree with the suggested change in language for the effect of phenology and changed “season specific” to “specific to the vine’s developmental stage” on L468. On gene expression, while no DEGs were identified, we were able to identify latent combinations of genes that were responsive to rootstock treatment. While this effect is subtle, it was nonetheless detectable.

5. Any biological interpretation of the specific metabolites, genes, iones, shapes determining the resulting PC covariation networks? While it can be interesting to add to covariation networks additional levels of phenomics as authors propose (lcRNA, micorobiome, epigenetics), it would also be informative to exploit the interpretation of the dataset that they have already produced.

- Response: Excellent suggestion. Unfortunately, after much consideration, at this point we do not feel comfortable with detailed biological interpretations based on specific metabolites or genes that underlie PC covariation networks shown here. Some of the limitations of our dataset, and why we are unable to make these mechanistic connections with data presented here, are detailed in the discussion. We note that the ionome offers a very rich source of data ripe for deep analysis, and that an additional manuscript describing a deep dive into multi-year, multi-time point ionomic dataset is in preparation now. We agree that future work should be targeted toward biological understanding of these relationships. On suggesting inclusion of other phenotypes, this comment reflects our enthusiasm for other existing approaches and exciting areas of research that might further uncover mechanistic understanding of the effects we are seeing from grafting and over time. The analysis presented in the paper, unfortunately, does little to advance us toward the goal of mechanistic understanding, but it does help us see where future studies could be targeted. To this end, we added language to clarify this point on L573-578.

6. L470, If the lack of rootstock effect on the transcriptome was due to the phenology effect, specific analysis at each phenology stage would identify rootstock genotype factor significant DEGs. Is it the case? Would there be any rootstock effect detected on transcriptome if the analysis was restricted to single blocks at specific phenological stages?

- Response: We thank the reviewer for this comment. This is certainly something we are trying to wade through as our results suggest that the rootstock influence on our vines is incredibly complex and works through interaction with other factors of the experimental design. Ongoing work is focused on identifying these complex effects, in a statistically robust way. We are also currently working with collaborators to identify genes and gene regions worth further exploration. Moreover, we are seeking to use results from other phenotypes to focus on genes in a more ‘hypothesis-driven’ approach that can further the ‘discovery-driven’ results observed here.

7. Apart from the seasonal effect, the "Potential implications" presented are not directly inferred from the Results obtained here but from the potential of the approach used. Any other potential implication of the specific results?

- Response: This is a very helpful suggestion. In response to this and comments from other reviewers we have re-worked the potential implications section. Other reviewers called for an enhanced focus on yield/viticultural implications, while others have asked us to minimize such speculation. Consequently, we have attempted to carefully place this work in the context of both basic plant biology and viticulture. If the current revision does not meet the expectations of the reviewer(s) or editors we would be happy to revise further.

8. Is there any data available for the distribution of soil properties across the experimental plot that could be considered to discuss the origin of block effects? Could the human factor during that extensive sampling be another variable accounting for block effect?

- Response: Thank you for this excellent observation. For the data presented here, we do not have paired soil samples. We anticipate some heterogeneity in soil properties across the experimental plot; however it is unclear how strongly this would correspond directly to block effect. Regarding the human factor, we have added a sentence into each data modality clarifying what variation is captured by the blocking factor. See each addition below:
    - L136-138 added, “Teams were deployed in the vineyard so that multiple vineyard rows were being sampled concurrently. As such, ‘block’ represented unmeasured spatial variation, but did not strictly correlate with time of sampling due to the nature of sampling (see Methods).“
    - L155 - 157 added, “ensuring that ‘block’ captured both unmeasured environmental variation and temporal variation over the sampling window”.
    - L187-190 added, “Leaves were sampled by a single team near midday between 10AM and 2PM in row order ensuring that ‘block’ and ‘row’ accounted for unmeasured spatial variation and temporal variation over the sampling window (see Methods)”
- Overall, block is not a large descriptor of variation in our study except for the phenotypes for which block is collinear with time of day. In these phenotypes (the metabolome and the transcriptome) there is a noted circadian topology. The other phenotypes (ionomics, leaf shape, and physiology) see little effect from block suggesting there is little spatial variation (or at least that the spatial variation is unimportant for those phenotypes).

9. Because half of 3309C reps would have been collected before any ungrafted rep was taken, could the LD2 effect in discriminating 3309C and ungrafted from RNA-seq data be related with sampling times? What are the genes involved in this effect?

- Response: We thank the reviewer for this comment. While it is always possible that results correlate with unmeasured confounders, rootstock genotype was not confounded

with any of the terms in our model (including time of sampling, which was a correlate for row in our study; L653-655). Each rootstock was present in each row of the vineyard in cells of four replicated vines (See supplemental Figure 1A). For the transcriptome sample, we sampled leaves from the middle two vines in each cell. While it certainly takes time to sample in a vineyard, each rootstock in each row would have been sampled within minutes of one another. Due to this, the grouping we report in LD space is not confounded with time. The current results suggest that this effect is driven by complex combinations of genes (from the PCA results) and not any particular genes being strongly affected (from the traditional DGE results). We are currently working on exploring these subtle effects in more statistically robust ways in a multi-year study.

10. Any discussion on the origin of leaf position effects in specific ions?

- Response: Thank you for this comment, and this is something we think about often. Leaf position was added to this study on the ionome because it is known that leaves vary in their elemental composition over development. The major question here was whether or not rootstock would influence the elemental composition of those leaves in such a way that the known patterns might be interrupted. While we observed significant variation in ion concentrations as a function of leaf position, it did not strongly interact with rootstock genotype. In other words, the rootstock effect was present in all leaves, not just leaves of a particular age. As such, we struggled to fit a detailed description of the effect of leaf position in the current paper. However, we have made all data from this analysis publicly available if there exists specific interest for the leaf position. Ongoing work focused explicitly on the leaf ionome will provide a deep dive on how ion concentrations vary by rootstock, over development, across seasons, and across multiple years.

11. L556. Indicating in there that "only the middle two vines of the four cells in the front half of the vineyard were included in the 72-vine set" would be handy to understand the distribution of this set.

- Response: Thank you for this comment. We have clarified the description of the experimental vine throughout the manuscript. The experimental design of the vineyard included groups of four identical vines (e.g., Chambourcin grafted to 3309C) that are distributed in a randomized block experimental design throughout the vineyard. For some data modalities we were unable to process samples from all four vines per cell. In these circumstances, we opted to collect samples from the middle two vines of the four vine set. We collected from the middle two vines from a total of 36 cells for the 72 vine set. To improve the understanding of this section, we have amended the text to point to more appropriate sections of Supplemental Figure 1. In addition, we have improved the Figure Legend for this figure so it is more clear what each panel is showing with explicit descriptions for Supplemental Figure 1B, which should improve the clarity of this section. Finally, we clarified that this description only applied to the front half of the vineyard which was missing from the previous draft. We would be happy to make additional edits to the text if this description does not provide sufficient clarification.

## Reviewer #2

The manuscript by Harris et al investigates the effect of grafting on a number of physiological and molecular phenotypes within grapevine (Vitis spp.) scions. The hybrid Vitis cultivar Chambourcin was compared when grown on its own-roots, or when grafted to three different commercial hybrid rootstocks: 1103P, 3309C, and SO4. The vines were grown in the field, irrigated with different volumes of water, and sampled over a single growing season. Large data sets have been generated for leaf metabolites, solutes (ions), transcripts, shape, and physiology (stomatal conductance, transpiration). As such, the manuscript fits the scope of Gigascience well. The manuscript is well written, however I found it was very statistical and would benefit from additional biological analyses to confirm and validate the findings. The methods section is lacking some details that would enable reproducibility. Some of the figures could be improved for readability. My comments and suggestions are detailed below:

### Major comments

1. There is no information on the age of the vines at the time of the experiments.
   - Response: This is a great observation of information we overlooked. The vineyard had been in the ground for eight years at the time of sampling. The age of the vineyard has been added to L538 and commented on in the discussion.

2. A quantitative analysis of the elemental content of the irrigation water by ICP-MS would be beneficial. In this study, it is unknown whether the irrigation treatments contained varying levels of the elements that were measured in leaves. To this end, it is perhaps not surprising that rootstocks had minimal effect on, for example, the Na+ content of grafted scions. However, it has been demonstrated previously that own-rooted vines cannot efficiently exclude Na+ compared to grafted vines when irrigated with 100 mM NaCl (see Fisarakis el al (2001) Agricultural Water Management 51 13-27).
   - Response: This is a really intriguing suggestion, and one we wish we would have thought of in 2017. Regrettably, it was not something that was considered for this study at the time. However, we can be confident that irrigation was not significantly altering the findings of our study due to the weak nature of the irrigation effect (See Supplemental Note 1). Had there been variation in the ionome of the irrigation water, we would have expected to see a stronger irrigation or irrigation by phenology effect, neither of which were strongly observed in this study. We thank the reviewer for pointing us to this helpful article.

3. The manuscript would be more useful to the plant science community if a subset of the actual metabolites and genes identified within the principle components were named and confirmed using a second method. It would then be possible to discuss which physiological, metabolic, and molecular processes within Vitis scions are impacted by rootstock selection.
   - Response:  We absolutely agree gene-level and metabolite-level understanding of the root system influence on shoot system phenotypes is the direction this work needs to head. This is perhaps one of the biggest limitations of large-scale analyses of multi-

dimensional phenotypes: it is sometimes hard to narrow in on individual phenotypes for some systems. We acknowledge that there is a trade-off between large-scale analyses like the one presented here and identification of actual metabolites/genes and their functional role in the vine. We see these as very complimentary approaches that illuminate different aspects of vine biology; however, we were unable to do both in this study. Ongoing work is attempting to, in a statistically robust way, uncover those subtle effects from even deeper sampling of the transcriptome. The metabolome as described using the untargeted approach here is a whole different monster. We were able to show that some metabolites are responding to the rootstock treatment and even to the rootstock by season interaction. The current nature of LC-MS and untargeted metabolomics in Vitis generally make it incredibly non-trivial to map these metabolites. Here we sought to catalog the basic responses of multiple phenotypes to help guide more targeted analyses and guide us toward studies that could produce mechanistic understanding.

4. Similar to my comment above, some of the data could be integrated. For example, transpiration was increased for scions grafted to 1103P (Fig 5B). Were genes or metabolites involved in the regulation of stomatal aperture differentially abundant when grafted to 1103P?

- Response: This kind of data integration is an excellent suggestion. While such analyses would require work beyond the scope of this paper, we think that this comment is exactly in line with how we should be guiding future work. We proposed in the previous comment that this work was meant as a foundation on which we establish the basic responses of many complex phenotypes over the growing season with respect to the rootstock genotype. The PCA-based integration was to help us narrow down which types of data modalities warrant future integrative work. As we move toward identifying and annotating individual genes and metabolites, these suggestions will certainly help in that future planning.

5. The ionomics data in Fig 1B and C would be easier to interpret if presented as a percentage - for example, % DW, % FW, or mM of tissue water. Currently, there are no units on the Y-axis.

- We agree that z-scores were not the ideal choice for this figure. To address this, we remade this portion of this figure to show the elements as concentrations in parts per million of acid-digested dried leaves. We have updated the Figure Legend L706 and L710 to reflect this change.

6. There is no mention of how the RNA was extracted from plant tissues. Further, a quality control would normally be performed, e.g. by measuring the 260/280 ratios at the very least. Was any quality control performed on these RNA samples? How do we know the samples were pure and not degraded?

- Response: Excellent point that was also noted by Reviewer 1. The information requested has been added to the Data Description section, L191 - 193: "Total RNA was extracted from plant tissues using the Sigma Spectrum Plant Total RNA kit with modification of the addition of 2% PVP40 to the extraction buffer to decrease phenolic

inhibitors.  All RNA extractions were checked for quality control using a Nanodrop. Sequencing was conducted using the Illumina NextSeq500 platform which returned single-end 86 bp reads."

7. It is unclear how many biological replicates were used for the RNAseq experiments.

- Response: Good catch- thank you for this. Language changes were made throughout the manuscript (in conjunction with other review comments in the section "Study Design" and within the Data Description for each modality to improve clarity. In short, each modality was sampled from either a 72-vine set (metabolomics, gene expression, physiology) or a 288-vine set (ionomics, leaf shape). In the 72-vine set, we only sampled the middle two vines from each four-vine cell in the vineyard (shown in Supplemental Figure 1B-C). At the highest order interaction for gene expression (rootstock:row:phenology), the number of biological replicates would be two. Since this is clearly underpowered, we put little effort into estimating or interpreting those effects. However, lower order interactions (like rootstock:row or rootstock:phenology) are averaged over the remaining samples. For example, the rootstock:row effect is estimated from 6 samples (averaged over phenology). Similarly, main effects are averaged over all other terms, so the rootstock effect would be estimated from 18 samples.

8. Usually, for genome-wide transcriptional studies, the expression patterns of a subset of genes are confirmed using another method (e.g. quantitative real-time PCR). This has not been performed in this manuscript. Authors need to confirm the validity of the RNA seq dataset.

- Response: Thank you for this comment. We agree that for studies focused on identifying genes associated with specific phenotypes, that the gold standard for genome-wide transcriptional studies is cross-validation using qPCR.  The goal of this study is to understand the influence of root system genotypes on shoot system phenotypes, and here we are treating gene expression in the leaves as a shoot system phenotype. Because we are not testing explicit hypotheses about any particular gene or pathway, we determined that PCR-based confirmation would not add value. This decision was not made lightly, and was done following consideration of other recent work that applied various RNAseq platforms to address structurally similar questions, including:

  1) Griffith M, Griffith OL, Mwenifumbo J, Goya R, Morrissy a S, et al. (2010) Alternative expression analysis by RNA sequencing. Nat Methods 7: 843–847. Doi:10.1038/nmeth.1503.

  2) Asmann YW, Klee EW, Thompson EA, Perez E a, Middha S, et al. (2009) 3' tag digital gene expression profiling of human brain and universal reference RNA using Illumina Genome Analyzer. BMC Genomics 10: 531. Doi:10.1186/1471-2164-10-531.

  3) Wu AR, Neff NF, Kalisky T, Dalerba P, Treutlein B, et al. (2014) Quantitative assessment of single-cell RNA-sequencing methods. Nat Methods 11: 41–46. Doi:10.1038/nmeth.2694.

  4) Shi Y, He M (2014) Differential gene expression identified by RNA-Seq and

qPCR in two sizes of pearl oyster (Pinctada fucata). Gene 538: 313–322. Doi:10.1016/j.gene.2014.01.031.

- Moreover,  we point to an excellent blogpost on the same validation-discussion that goes further in to this debate: http://dave-bridges.blogspot.no/2014/11/validation-of-rnaseq-experiments-by-qpcr.html?m=1
- We agree that it adds value to ensure that our data show previously established patterns of house-keeping and circadian-phased genes relevant to our sampling paradigm. As such, the following have been added to the manuscript:
    - Added to data description L207 - 210, “To check the validity of our expression results, we assayed two classes of housekeeping gene (Ubiquitin-domain and actin-family) and eight previously annotated circadian genes (Carbonell-Bejerano et al. 2014)”
    - Added to Analyses, L339 - 341, “We computed the expression of two classes of housekeeping genes, and show that they are generally stable (Supplemental Figure 2). We note that some variation is expected for housekeeping genes; see, for example, (Liang et al. 2018). Moreover, we show that patterns of previously annotated circadian genes show expected results over the sampling window. For example, predicted orthologs of LHY and RVE1 are correlated and decreasing over our sampling window, and a predicted TOC1 ortholog is invariant. That our samples showed such patterns suggested the gene expression data presented here were valid.”
    - Created and added Supplemental Figure (now supplemental Figure 2) showing these patterns.

9. The effect of the different irrigation regimes is not adequately discussed in this manuscript.

- Response: Thank you for this note - good catch. Effects of different irrigation regimes were originally presented in a Supplemental Note hosted on GitHub. To increase accessibility, we moved Supplemental Note 1 from GitHub so that it is now a Supplemental Note to the manuscript.
- Some additional notes: our study site is located in southwestern Missouri where it can be quite rainy. In 2017, the year in which samples were collected for this study,  it rained a lot, essentially rendering the vines all properly irrigated despite the amount of irrigation applied as part of the study. Not surprisingly, our physiological metrics showed little/no evidence of stress. As such, we opted to include irrigation as a term in the model that is there and could impart variation into some phenotypes, but those effects are very small.

10. The Abstract must be structured into three separate sections: Background; Results; Conclusions.

- Response: The abstract has been restructured to meet the suggested format. We thank the reviewer for catching this oversight.

## Minor comments

1. A lot of the information under "data description" should be moved to the methods section. For me, the data description should provide more of a background and rationale of the work, while the methods should provide the actual steps that were taken.
    - Response: Thank you for this note. We struggled to balance the need to provide enough information for readers to understand the work up front, while saving the majority of methodological details for the methods section. In its current version, we hope that we have provided the appropriate information in the approximately preferred locations according to the journal's instructions. If there are persistent issues with information placement in the current version of the manuscript, we would be happy to address those in whatever way the editor/reviewers request.

2. Although it is alluded to in the introduction and data description, the tissue type that was harvested and used for the RNAseq experiments is not mentioned in the methods or analyses sections.
    - Response: Good catch, and we regret this omission. The tissue type used for RNAseq experiments were young, fully opened leaves. We added information about the tissue type in the analysis section, and assured it was explicitly mentioned in the data description.
        - L185 starts "The youngest fully-opened leaves"
        - L336 added "youngest fully-opened leaves"

3. The Figure 1B legend should denote what Y, M, and O mean. I realise that it is young, middle and old, but the legend should stand alone.
    - Response: Another good catch. We added a short key to L705 - 706 indicating Y (young), M (middle), and O (old).

4. Significant differences in Fig 1B and Fig 5 B&C could be annotated within the Figure, for example with an asterisk.
    - Response: Excellent suggestion. Significant comparisons have been labelled with letters in the identified figures, and the figure legends have been edited to explain them.

5. Please be careful to use the past tense consistently, for example P16, L403 "correlation between gPC4 and pPC3 is similar" should be 403 "correlation between gPC4 and pPC3 was similar".
    - Response: Thank you for this important catch. Tense was changed in the cited examples and edited throughout the manuscript.

6. P19, L472 "stomatal conductance were higher vines" should be "stomatal conductance were higher in vines".
    - Response: Added 'in' to L512

7. P19, L475 "Understanding of rootstock genotype influence shoot system phenotypes" should be "Understanding of how rootstock genotype influence shoot system phenotypes".

- Response: Edited L516 to read, “Understanding rootstock genotype influence on shoot system phenotypes”

8. Perhaps consider re-writing the title to the Fig 5 legend. "Vine physiology measurements show signal from most experimental manipulation" does not make sense to me.

- Response: Edited title on L746 to “Vine physiology varies with rootstock and the rootstock by phenology interaction”

**Nice work.**

Thank you for this. We appreciate the detailed review.

## Reviewer #3

This study investigate associations between rootstock genotype and shoot system phenotypes using five multi-dimensional approaches contributing to elucidate how root systems influence vine phenotype.

the influence of rootstock on the traits analyzed are roughly well documented in literature and authors are aware about this since they very often commented that results are consistent with previous study. Hence the reader might question about the limited new information provided. I would recommend the authors at the "potential implications" paragraph to avoid speculation on "yield" and to emphasis the novelty of engaging a simultaneously analysis as they did in order to speed up comparative studies.

- Response: This is a very helpful suggestion. In response to this and comments from other reviewers we have re-worked the potential implications section. Other reviewers called for an enhanced focus on yield/viticultural implications; however, we agree with this reviewer’s request to minimize speculation. Consequently, we have attempted to carefully place this work in the context of both basic plant biology and viticulture. If the current revision does not meet the expectations of the reviewer(s) or editors we would be happy to revise further.

### Minor comments

1. At line 226-227, check "umol/s" replace with [?]mol s-1 ?
   - Response: Unfortunately the symbol the reviewer suggested did not render in the communications (we can’t see it). We have replaced the umol with the more commonly accepted µmol where appropriate. If another symbol is preferred or a different symbol was meant, please let us know and we would be happy to make the requested change.

2. At line 231, is 15 min interval time enough to equilibrate? Considering that usually 30 or 60 min are required (e.g., J.Int.Sci.VigneVin, 2012, 46, n°3, 207-219, See https://urldefense.com/v3/__https://doi.org/10.20870/IVES-TR.2020.3620__;!!K543PA!bnhJBaYGb-6O8nkV-F90YalIxoa2UGVyHkLiToTGXSjDbduO2MrZFPISJayIAoKRcnAWzw$See ISBN 978-90-481-9282-3 at pag 89), please justify your 15 min interval.

- Response: We thank the reviewer for this thoughtful comment. We acknowledge that this is a topic of much debate. A 15 minute equilibration has been used in the past to measure midday stem water potential for tree species, and explicit testing showed that there was little difference between a 10-15 equilibration and a >1hr equilibration in oak trees (https://www.fs.fed.us/psw/publications/documents/psw_gtr184/psw_gtr184_035_ShackelGross.pdf). A more recent study (published after our work was completed) suggests that there is a small effect from different equilibration times in grapevine, but that effect is smaller than the effect from the person operating the pressure chamber (https://doi.org/10.1016/j.agwat.2019.03.026). We have amended the section of the manuscript to include these references for future readers (L254-255).

3. Please note that "old" and "young" communicate leaf age rather than leaf position, what's about top, middle, bottom?

- Response: This has been a topic of much debate on our team and we really appreciate this comment. The designation of “old”, “middle” and “young” stem essentially reflect terminology used by our team since the inception of the project. We totally agree that these terms reflect leaf age rather than position. However, in grapevines these are equivalent because the oldest leaf along a vine is at the bottom of the shoot and the youngest leaves are at the top of the shoot. All things being equal we would readily make this change; however, the current terminology is used in this paper and in many other completed or ongoing manuscripts being carried out by members of our team. If it is amenable to the editor and the reviewer, we would prefer to retain the “old” “middle” and “young” designation. However, if this is unworkable we will make changes to the language.

4. It is not clear why 1103 P had a very little variability of gs at anthesis compared to other rootstocks, for these plant water status seems to range from well irrigated to deep stressed vines while 1103P vines seem to be all roughly well irrigated.

- Response: We appreciate this observation. It is not immediately clear why vines grafted to 1103P showed such little variation in stomatal conductance at anthesis. Unfortunately we don’t think we can test this with the current study. To investigate this and related questions we completed a greenhouse study with 1103P and other rootstocks grafted with a common scion with an irrigation treatment. This work is in preparation now.

5. Providing VPD data might help to explain why transpiration is low at anthesis (approx. 2.5 mmol m-2 s-1) while gs at anthesis is comparable to that of other sampling time.

- Response: Thank you for this interesting point. We agree that features of the environment (like VPD) will partially explain the differences we see across the time point in this and future studies. Ongoing work is attempting to identify features of the environments that correlate and can explain some of the variation we see in these traits. This is partially undermined by natural season changes, so these relationships are hard to untangle and require a substantial amount of data, much beyond the three time points

presented here. However, we appreciate this comment and hope to address this in future works.

6. "leaf position" should also be discussed against "leaf angle" (e.g., https://urldefense.com/v3/__https://doi.org/10.3389/fpls.2020.00595__;!!K543PA!bnhJBaYGb-6O8nkV-F90YalIxoa2UGVyHkLiToTGXSjDbduO2MrZFPISJayIAoLo-b4lwA$ ) which likely change across the season due to change of soil water availability. was leaf angle accounted for image analysis? Considering that soil moisture reasonably differed at the three stages considered (Fig. 5).

- Response: Thank you for this insightful comment. We agree that leaf angle is important for physiology, and would likely correlate with some of the traits we measured. Unfortunately, leaf angle was not quantified at the time of collection in the field. Leaves were simply chosen from vines that emerged directly from the cordon and had intact young, middle, and old leaves. Leaf scans were completed in the lab after leaves had been removed from the vine, and it was not possible to quantify leaf angle at this time. Having said that, this is an important consideration for future studies and we very much appreciate this observation.

7. Please add the mean leaf water potential and soil moisture values directly in the Fig. 5 panels to help the readers.

- Response: We thank the reviewer for this suggestion on improving our figures. We have added the mean value for reach rootstock/phenology combination in Figure 5 and, for consistency, to Figure 1. Figurel legends have been edited to address these changes on L712 and L751.

## Reviewer #4

This 'big data' manuscripts offers a comprehensive snapshot of the grape phenome as influenced by several factors, including ionomics, leaf morphology, physiological data, metabolomics and transcriptomics. The overall scope is ambitious and a step forward for the phenomics community. Overall the paper was well-written and the design and analysis are sound, though I had a few questions below.

Thank you for these very kind and encouraging words.

### I had a few suggestions.

1. Phenomic and phenotypic are used interchangeably, and I would ask they be clearly defined - should they really mean the same thing? What's the difference between a phenome and a phenotype?

- Draft response: Thank you for this important observation. We define “phenomics” to be a “field characterized as the acquisition and analysis of high-dimensional phenotypic data at hierarchical levels, often with an eye toward multiscale data integration” in the introduction. We define a phenotype as a single particular trait (e.g., calcium concentration). As such, we have amended usages of these words to comport with this

definition: phenomic (and phenomics) now refer to the joint analysis of multiple data modalites, each of which contain several phenotypes (or a single multi-dimensional phenotype). In addition, we recognize that we were being imprecise with language here, so we have fixed this and other terms used interchangeably (trait/phenotype, phenotypic/phenomic, and data type/modality) throughout the manuscript.

2. Is the paper considering transcriptomics as phenomics? I know it's a debated issue really, but would be good to state so and why.
- Draft response: Thanks for this comment, like many groups we have spent a lot of time thinking about the question of whether or not the transcriptome is a phenomic modality. In the introduction of this manuscript, we loosely acknowledge phenomics as the field of study concerned with high-throughput data acquisition through multiple simultaneous trait measurements, often requiring advanced computation to analyze and integrate L62-63. Following this definition, we treat the transcriptome as a multi-dimensional phenotype (or that the extent to which a particular gene is expressed at a particular time in a particular place is a measurable trait/phenotype). In the analysis and interpretation of the data in this manuscript, we treat the transcriptome like the other data modalities presented here.

3. Related, phenotype and trait are inconsistently used as detailed below. I recommend to define them and use consistently. This is a huge problem for phenomics and I think prevents clear discussion of the topic.
- Response: We thank the reviewer for this comment on clarity. Throughout the manuscript we have edited the language we used to describe phenotypes to be consistent. In particular, we have edited each usage of ‘trait’ to ‘phenotype. As above, we recognize that we were being imprecise with language, so we have fixed this and other terms used interchangeably (trait/phenotype, phenotypic/phenomic, and data type/modality) throughout the manuscript.
-

4. I had some questions about the experimental design and randomization, detailed in line comments. I'm not sure about the claim of 72 replicates. Maybe it's a question of what should be considered an experimental unit.
- Response: Other reviewers also noted lack of clarity with respect to experimental design, and we appreciate this observationt. A full response to this concern can be found in our response to your comment on L561 (below); which is partially copied here: I think some additional confusion may stem from us using “replicate” as a vague stand-in for both clonal replicates and statistical replicates. To address this, we have amended the language about the four rootstock scion combinations as follows on L617: “Clonal replicates of each of the four rootstock-scion combinations were planted 72 times for a total of 288 vines planted in nine rows”. In addition, we included the specific type of design (split-plot) to this section. Finally, we addressed the number of true replicates in a comment by reviewer 2 concerning RNAseq. The same logic can be used to derive the total number of biological replicates for leaf shape and ionomics at the highest order

interactions (4) and for all other phenotypes (2). In the case where the number of biological replicates is two, the estimation and interpretation of effects is minimized due to lack of power.

5. The analysis of individual datasets (or modalities, good word) seems good, and I think the approach to combine into a larger set using the PCA is pretty clever. I still wondered how 'fused' the data really is but can't really think of a better way other than combining all the raw data except then the number of genes and metabolites would just swamp the analysis I guess. Perhaps the authors could articulate why this is a good fusion approach they've used, and perhaps what could be done in the future.
- Draft response: Thank you for this kind observation and really insightful comment. We considered a larger integrative framework that would include all phenotypes measured in the study. However, as the reviewer identified, this would include a heavy bias toward gene expression (expression data for 24,000+ transcripts) and metabolomics (600+ different features measured) which would likely overpower leaf shape (17 x,y coordinates) and ionomics (20 ions). We felt that the PCA approach allowed us to weigh each modality more evenly in order to see if further integrative efforts were warranted. Based on these high-level results, it looks like integration among modalities is a warranted effort, especially if we could collect more targeted data that could expand mechanistic understanding of observed patterns. However, the scope of these integrative techniques is broad and several papers could likely be written just exploring differences in integration techniques with just a single phenotype, for example, gene expression. We have edited the sentence on L430-431 to reflect this logic: "Within each phenotyping modality, we summarized the primary dimensions of phenotypic variation using PCA (see Methods), so as to not weigh any modality too heavily."

6. I Biologically, I'd like to see more insights to why these traits matter. How could understanding that these traits change help production? I think some arm waving is warranted. Especially, how is understanding the correlation among modalities important? One idea is to identify trade-offs and synergisms?
- Response: We thank the reviewer for this suggestion. We modified the language of the potential implications to suggest some ways in which this kind of work could balloon into other phenotypes (not measured for this study) that are more useful to breeding through synergistic relationships (enhancement), trade-offs (constraint), or just simply predictability. Moreover, we maintain that the broadest implication is the notion that there is a strong temporal component to phenotypic expression in long-lived perennial plants and that grafting and rootstock genotype add another dimension to it.

7. Last, I'm happy to see how much data is shared. However, GitHub is not appropriate for sharing data, which should all be on a public repository, including the analysis scripts. I think FigShare has been used for other permanent data, so I recommend to share the scripts there.

- Response: We thank the reviewer for this insight. The note on irrigation, which was initially uploaded to GitHub, has been added as a Supplemental Note to this manuscript. This note will additionally stay on github for easy access. All phenotypic data from the ionome, metabolome, leaf shape, and physiology are on Figshare and the gene expression are on the SRA. In addition, we are in the process of submitting raw metabolomics data to the Metabolights database, as requested by GigaScience.

**Line comments and other details follow:**

39: In my opinion, the 'hyphens' are not needed in belowground and aboveground.

- Response: From what I can gather, above-ground and aboveground are considered to have the same meaning. We leave this stylistic choice up to the editor.

45: "change"

- Response: L46: changes -> change

46: long sentence with semicolon, consider making that a period, but the use of many interjections make it a little hard to parse still

- Response: Good catch. This sentence has been split into two (now L44).

99: are phenotypes and traits taken to be completely synonymous in this paper? Given that many definitions are used of each, it would be helpful to define. For example, both can be used to describe the 'general' properties like 'eye color' or the specific like 'blue eyes.' Phenotype, in addition, is sometimes used to describe the totality of all trait values in an organism. More careful and exact usage would benefit the paper. For example, trait value can also describe the specific like 'blue eyes' while trait the general 'eye color.' The title of your paper suggests that you additionally consider the phenotype as all traits (or trait values?). Leaf shape is referred to as a phenotype at line 439, so consistent with the 'general trait' definition.

- Response: We agree this was a persistent problem in the initial version of the manuscript. As above, we recognize that we were being imprecise with language, so we have fixed this and other terms used interchangeably (trait/phenotype, phenotypic/phenomic, and data type/modality) throughout the manuscript.

111: what would the difference bet tween phenomic and phenotypic variation? what is the definition of the phenome? phenotypic variation is also used at line 434

- Response: Another good catch.  We have edited the language throughout the text so that it is now consistent. We did not actually mean to distinguish between these two things in the highlighted example. As above, we recognize that we were being imprecise with language, so we have fixed this and other terms used interchangeably (trait/phenotype, phenotypic/phenomic, and data type/modality) throughout the manuscript.

115: Were there any hypotheses? Is the intent to be descriptive?

- Response: The goals of this study were to address three questions: 1) what is the influence of root system genotype on shoot system phenotype? 2) How do systems of plant phenotypes vary over the growing season and does rootstock genotype influence this variation? And 3) how do phenotypes covary within and between phenotyping modalities? For clarity, we have enumerated these questions in the Study Design section. L639 - 642.

118: Are the details of the experimental design needed here because of the wonky format of a GigaScience paper with methods at the end? Not your fault, but I find these formats so confusing and redundant since authors try to move methods into other sections to make up for it.

- Response: We appreciate this comment. This was certainly a design choice by us so that the paper could be understood linearly.

139: if this pipeline is capitalized and sort of 'official' - is there a citation or access to details of it?

- Response: This is a standardized pipeline at the Donald Danforth Plant Science Center. The sentences surrounding this line (now L140-146) have been restructured to make this more clear: “Between 20 and 100 mg of leaf tissue was acid digested and 20 ions were quantified using inductively coupled plasma mass spectrometry (ICP-MS) following standard protocol of the Donald Danforth Plant Science Center (DDPSC) Ionomics Pipeline [30,31]. Ion quantifications were corrected for internal standard concentrations, instrument drift and by initial sample mass. The output of the Pipeline contained measures for each of the following 20 elements: Al, As, B, Ca, Cd, Co, Cu, Fe, K, Mg, Mn, Mo, Na, Ni, P, Rb, S, Se, Sr, and Zn.”

140: Why the difference for ML?

- Response: Z-scores were used in the linear models for ion concentrations so that models could be compared. However, the random forest is a single model that needs no adjustment on the input space. We included a small comment that non-standardized input is the convention for random forests (however many ML models do need to be standardized to equally weight each feature).

141: This Leaf Ionomics section, to me, describes the method to sample and measure, but fails to describe the final output? How many ions? which? I don't fully understand why GigaScience requests this format, but it does mention the background should be given. SO, I think you should say why the ionome is important, and the same for other trait conglomerates mentioned in the paper.

- Response: This is helpful - thank you. We have added the following:
    - to L145-146 to explain the ionomics data set, “The output of the Pipeline contained measures for each of the following 20 elements: Al, As, B, Ca, Cd, Co, Cu, Fe, K, Mg, Mn, Mo, Na, Ni, P, Rb, S, Se, Sr, and Zn.”
    - To L179 to explain the metabolomics data set, “The 661 identified metabolomic features...”

143: carbon-based molecules? For example, not nutrient ions?

- Response: Added “mostly organic” to enhance description of the metabolome

144: I had to look up 'veraison' - could you put 'ripening' in parentheses if that captures that idea?

- Response: Clarified as the “onset of fruit ripening” on L153

210: scanning details? background, color, DPI, image format?

- Response: L228 - 229: added “in color against a white background at 1200 DPI and written as JPEG formatted images”.

236: recommend to again announce the number of ions analyzed

- Response: Added “and measured the concentrations of 20 ions” to L262

244: It's not clear what the percentage refers to, I imagine percentage of total variation accounted for by that factor, ie the effect size. recommend to include 'effect size'

- Response: Added variation explained to first usage

249: Giving the effect sizes is a reasonable summary given your multiple factors, however I think giving some indication of absolutely changes is also relevant? Like, what type of percent changes were observed across all the samples in absolute terms, or give the min and max for some ions? Obviously you can't be exhaustive, but this would put the effect size in some type of context of biological influence, like rootstock explaining 10% of variation in a 1% change in Ca vs a 100% change in in Ca. Hope that's understandable. Perhaps these absolute changes would be most relevant where you highlight the influence of rootstocks?

- Response: Thanks for this thoughtful comment. The value of effect sizes like percent variation explained are that they can be directly compared if the models are parameterized in an identical manner. However, I think this confusion could be clarified by projecting samples back into a real concentration space which has now been done for the figure.

267: Could MDA be spelled out on first mention?

- Response: L293 now includes Mean Decrease in Accuracy. It is also defined in the methods.

350: personal placeholder to check discussion for how so much variation isn't accounted for - seems surprising!

- Response: We agree that the lack of variation explained in the models for leaf shape is quite interesting. Future work will certainly explore factors such as variation imparted from individual vine and environmental variation to attempt to explain this.

400: I'm confused that the PCs should correlate from the same modality, something which I thought didn't usually happen?

- Response: There is statistical literature on this topic. In short, principal components are orthogonal, however orthogonal does not always mean uncorrelated. See Rodgers, Nicewander, and Toothaker, 1984.

462: Good to bring up the biological implications - what are they? Are these changes relevant for growth, taste, etc?

- Response: We agree that the earlier version of this manuscript was missing key information about why the ionome is important. Unfortunately, there is not a lot of work tying together the elements of the ionome that we identified as responsive to rootstock genotype. Traits of biological interest, features that are known to be influenced by ion uptake by the root system (rootstock) are now mentioned in the manuscript, including vine growth and fruit/wine quality. We have added a comment on this in the discussion to address that there is a known connection between macronutrients and these traits, but more work is needed is to uncover these connections with micronutrients in grapevine. To this end, we added the following to L518 - 520: "To our knowledge, there is not yet a strong causal link between the micronutrient component of the ionome and factors of vine growth or development that might influence traits like wine quality. However, it is noted that macronutrient deficiencies can have negative effects on such traits (Bravdo 2000; Brunetto et al. 2015) and can be mediated by rootstock (Gautier et al. 2018). This suggests a strong understanding of the rootstock influence on the vine's ionome is warranted, and more work needs to be done to establish these relationships"

474: Can stomatal conductance be limited by flow in the roots? Do you think it's more likely such an indirect effect, or a direct effect such as signals from the rootstock actually change the rates by stomatal closure, etc?

- Response: This is a really good question that is particularly challenging to fully address. We would wager that root architecture is a key driver of physiological variation, and we tested this in a greenhouse study the results of which are in preparation now. However, it is worth noting signals could be passed from the rootstock, but the space of signal passing through graft junctions is complex and this work was not designed to address anything to that end.

501: I think here, rather than 'phenotype' as 'traits' you meant to say something about the 'data types,' which you referred to as modalities before and would be appropriate to use here. 'traits of different modalities'. I would suggest this instead of 'data types' perhaps for consistency. Definitely it would be a benefit to the field to have ways to describe these 'groups' of traits from the same instrument, my lab also runs into this with fused, multi-dimensional data.

- Response: Great observation, and we agree. We have fixed this instance of improper usage and we have clarified the language in the discussion (and the rest of the manuscript) to be more consistent. Specific to this comment, 'data type' has been replaced with modality/ies throughout the piece. As above, we recognize that we were being imprecise with language, so we have fixed this and other terms used interchangeably (trait/phenotype, phenotypic/phenomic, and data type/modality) throughout the manuscript.

502: latent phenotypes were mentioned in the definition of phenomics (where I only see them as one possibility, not a defining feature). Some definition would be useful.

- Response: This is a good catch. We did not mean to imply latent phenotypes were the only possible outcome of this work. This sentence was expanded a bit to include that idea that latent structure is one possibility, but using this to target integrative analysis is also a strong possibility. (L565)

510: back to phenomic correlation - what's the difference with phenotypic correlation?

- Response: Good catch of this persistent issue in the earlier version of the manuscript. We did not mean to imply there was a difference, or to make any kind of statement on this distinction. As above, we recognize that we were being imprecise with language, so we have fixed this and other terms used interchangeably (trait/phenotype, phenotypic/phenomic, and data type/modality) throughout the manuscript.

533: Very little information was provided about how the changes measured here in traits would affect yield or other consumer-facing traits. Not only that, but why is the multi-dimensionality important? Does it reveal trade-offs in traits, for example? I'm trying to help you improve the biological impact component. Some arm waving may be warranted.

- Response: We very much appreciate the direction the reviewer is going here, and we have attempted to address this in the potential implications section of the manuscript and elsewhere. Multi-dimensional data are data that consist of many different observations (for example, the ionome which includes measurements of 20 different ions). Multi-dimensional data offer more robust, approaching comprehensive observations of plant phenotypes. They offer a rich source of information that can be used to more comprehensively understand the basic biology of the organism - for example, how root systems influence features of shoot systems in grafted plants. This is described in, for example, L94-100 of the introduction. The influence of the phenotypes we measured on yield or other consumer facing traits are under active investigation. For example, ongoing work by others members of our project team describes berry chemistry and wine volatiles for the experimental vineyard described here. The volume of data was so large; this manuscript represents the first step in processing and interpreting multiple multi-dimensional phenotypes and trying to understand what approaches can be used to understand how they relate to one another. The next steps will be to connect these data with observations that might be more directly relevant to viticulturists. Our hope is that this manuscript will provide the foundation for those analyses that integrate multi-dimensional data from different organ systems, such as leaves and berries.

457: This note is very thorough and appreciated, however a github link is not permanent and therefore I suggest to include as supplemental to this paper or else place on a 'permanent' public repository such data dryad, Zenodo, etc. If the irrigation factor was ignored, you should say so.

- Response: Good point. The note on irrigation has been added as a Supplemental Note to this manuscript. Irrigation was treated as an additional blocking factor in the analyses done here. While we will keep the other data available on Figshare, we are exploring other homes for the data that are in line with GigaScience's preferences.

561: After reading this section, I wasn't sure about the experimental design, especially what type of randomization was used. I would guess that an appropriate design here would have been split plot block design taking into account irrigation (which I guess you are saying you ignored in the end). Were genotype randomized? the groups of 4 are mentioned, should that be taken as the experimental unit? I'm not super picky about stats, but some might say there are flaws here, and perhaps the 72 should be divided by 4 as as far as complete replicates? In Supp Fig 1 in the map, I see up to Block F - so should it be 6 true replicates? In cases likes this, I usually think of the additional plants as subreplicates. Your design seems basically just like a annual crop field trial with small plots with multiple plants. We usually measure a trait on those subreps then average it to the plot level for further analysis. In that case, the subreplication isn't used in stats directly, but does allow a better approximation of the value for each plot and decrease overall 'random' or 'environmental' error.

- Response: This is a great catch by the reviewer. We regret that the earlier version of this manuscript did not fully explain the experimental design of the research vineyard used in this study. These details have been filled in in section Study Design of the manuscript. Further, I think some additional confusion may stem from us using "replicate" as a vague stand-in for both clonal replicates and statistical replicates. To address this, we have amended the language about the four rootstock scion combinations as follows on L617: "Clonal replicates of each of the four rootstock-scion combinations were planted 72 times for a total of 288 vines planted in nine rows". In addition, we included the specific type of design (split-plot) to this section. Finally, we addressed the number of true replicates in a comment by reviewer 2 concerning RNAseq. The same logic can be used to derive the total number of biological replicates for leaf shape and ionomics at the highest order interactions (4) and for all other phenotypes (2). In the case where the number of biological replicates is two, the estimation and interpretation of effects is minimized due to lack of power.
