# Supplementary material for: Multi-dimensional leaf phenotypes reflect root system genotype in grafted grapevine over the growing season
Source: Gigascience. 2021 Dec 29;10(12):giab087. doi: 10.1093/gigascience/giab087 (PMC8716362; doi:10.1093/gigascience/giab087)
Supplement: giab087_GIGA-D-21-00137_Revision_2 [file giab087_giga-d-21-00137_revision_2.pdf]

GigaScience

Multi-dimensional leaf phenotypes reflect root system genotype in grafted grapevine over the growing season

--Manuscript Draft--

Manuscript Number:	GIGA-D-21-00137R2
Full Title:	Multi-dimensional leaf phenotypes reflect root system genotype in grafted grapevine over the growing season
Article Type:	Research
Funding Information:	National Science Foundation (1546869) — Dr Allison J. Miller
Abstract:	Background: Modern biological approaches generate volumes of multi-dimensional data, offering unprecedented opportunities to address biological questions previously beyond reach due to small or subtle effects. A fundamental question in plant biology is the extent to which below-ground activity in the root system influences above-ground phenotypes expressed in the shoot system. Grafting, an ancient horticultural practice that fuses the root system of one individual (the rootstock) with the shoot system of a second, genetically distinct individual (the scion), is a powerful experimental system to understand below-ground effects on above-ground phenotypes. Previous studies on grafted grapevines have detected rootstock influence on scion phenotypes including physiology and berry chemistry. However, the extent of the rootstock's influence on leaves, the photosynthetic engines of the vine, and how those effects change over the course of a growing season, are still largely unknown.<br>Results: Here, we investigate associations between rootstock genotype and shoot system phenotypes using five multi-dimensional leaf phenotyping modalities measured in a common grafted scion: ionomics, metabolomics, transcriptomics, morphometrics, and physiology. Rootstock influence is ubiquitous but subtle across modalities with the strongest signature of rootstock observed in the leaf ionome. Moreover, we find that the extent of rootstock influence on scion phenotypes and patterns of phenomic covariation are highly dynamic across the season.<br>Conclusions: These findings substantially expand previously identified patterns to demonstrate that rootstock influence on scion phenotypes is complex and dynamic and underscore that broad understanding necessitates volumes of multi-dimensional data previously unmet.
Corresponding Author:	Zachary N Harris<br>Saint Louis University<br>SAINT LOUIS, MO UNITED STATES
Corresponding Author Secondary Information:	
Corresponding Author's Institution:	Saint Louis University
Corresponding Author's Secondary Institution:	
First Author:	Zachary N Harris
First Author Secondary Information:	
Order of Authors:	Zachary N Harris
	Mani Awale
	Niyati Bhakta
	Daniel H. Chitwood
	Anne Fennell
	Emma Frawley
	Laura L. Klein

Powered by Editorial Manager® and ProduXion Manager® from Aries Systems Corporation

	Laszlo G. Kovacs
	Misha Kwasniewski
	Jason P. Londo
	Qin Ma
	Zoë Migicovsky
	Joel F. Swift
	Allison J. Miller
**Order of Authors Secondary Information:**	
**Response to Reviewers:**	Note to all: Microsoft Word on macOS does not allow correct continuous line numbering with "track changes" on. All referenced line numbers were identified such that they were continuous. If line numbers appear way off, try changing "All Markup" to "Simple Markup" under the Review tab to align the line numbers.<br><br>All revisions from this round are labelled Revision_2.<br><br>Editor Comments:<br>With regards to Reviewer #4 comments and Github - if the manuscript is deemed acceptable for publication, GigaScience will always take snapshots and host that, along with other supporting data and metadata under a CC0 license. So despite the reviewer's' concern about GitHub not being a permanent repository, there will be copies permanent in our open repository, GigaDB.<br>Response: We thank the editor for their work on this manuscript. We are happy to have additional copies of all of our scripts and data sets hosted redundantly across multiple repositories. Our intention with GitHub was to store the analysis scripts as permanent versions of record. As we do not come from software development, we were using GitHub as a convenient home rather than as a live repository for ongoing projects. One additional note: we uploaded all metabolomics data to Metabolites, but have not received a response from that submission. We would be happy to store an additional copy on GigaDB, if appropriate.<br><br>Reviewer 1:<br>Relevant methodological information that was missing from the previous submission has been added to the revised manuscript by Harris and co-workers, which enables a more conscious interpretation of the results. Experimental limitations and external sources of variation have also been considered when discussing the results. In addition, cross-check of expected expression profiles for a selection of genes has been included as a validation of the RNA-seq experiment reliability.<br>Response: We thank the reviewer for their careful review and re-review of the manuscript. Comments made by reviewers have considerably strengthened the manuscript and we really appreciate it.<br><br>Considering all the information, despite a huge multilevel dataset was generated, its value is limited by experimental design deficiencies recognized by the authors (e.g.: only one year of study under field conditions, noise of environmental/circadian variation during extensive physiological phenotyping and RNA-seq sampling throughout relatively long periods of the day, theoretically low power of the RNA-seq experiment due to relatively low read depth and low replication in some comparisons with only two replicates). Altogether, the manuscript is mostly descriptive of general differences rather than conclusive. Some of the main observations have already been documented before, such as the idea that rootstock genotype affects scion leaf phenotypes. Regardless, in the current version of the manuscript, the study and its limitations are fairly presented by the authors in a manner that would be acceptable for publication if the journal considers the dataset of value in spite of these experimental limitations. Besides this general concern, I would only have a few minor comments to this version:<br><br>1. The dataset might still be undermined as only general descriptive differences are presented as conclusions, but nothing about their possible origin is mentioned. For instance, what are the known intrinsic features of the compared rootstocks according to the bibliography that could determine the observed differences in ionic composition?

	How could these rootstock-determined differences in ion accumulation affect vine performance? Similar questions would arise for other differences observed.<br>-Response: Thank you for this comment, and we share a strong interest in understanding intrinsic features of rootstocks that affect the observed differences in the grafted scion. Studies that begin to get at these questions are underway within our research team now, but unfortunately are not completed and not included in this manuscript. To address the reviewer comments here, we specified that, especially in the case of the ionome, the differences are likely due to the genetics/ pedigree of the rootstock on L521-523. Additional comments added in the last round of revision explain how we are presently unsure how individual ions map to aspects of vine performance. We know even less about the other phenotypes. Future analyses using the data set we presented, additional data that were beyond the scope of leaf phenotyping, and future data can and should address this type of question.<br><br>2. It could be more specifically pointed out that lack of DEGs in some RNA-seq comparisons could be due to the experimental limitations (e.g.: low replication and 4.1 M read depth below the minimum recommended 5 M) rather than to a real lack of effect of rootstock genotype.<br>-Response: Agreed. We added a note to the Data Description that we opted to sequence more samples at the cost of some read depth which does limit our power to detect some low-expression genes on L195-196. We recognize that replication is low for high order interactions (rootstock:row:phenology) due to only sampling two vines per cell. Because of this low replication, we did not interpret such effects because they would be underpowered. However we sampled 36 cells at each time point for a total of 216 samples (with a few removed for poor sequencing), so lower order interactions and main effects were derived from much larger pools of clonally replicated samples. Specific details on this can be found in response to Reviewer 2 and 4 in the first revision.<br><br>3. The value of including PC covariation networks would be scarce if the results are not reliable enough for interpreting the inter-connection identified between the responsible specific metabolites, ions, genes, etc.<br>-Response: It’s true, and we agree that any issues present in individual data sets will percolate into integrative analyses. Having said that, we are confident in the individual datasets and in our approach using those datasets in PC covariation networks. Focusing on PCs from each modality allowed us to capture the highest levels of variation to see how those PCs relate across modalities. We chose this analysis so that no particular modality was over-weighted and so that we could narrow down where interesting correlations lie such that we can design and craft better future studies. We recognize this approach has limitations, but after exploring many different potential options we felt this was the most appropriate given the data and the questions.<br><br>4. Several typos should be corrected in the newly added text.<br>-Response: We thank the reviewer for the close reading of the text. We have edited the manuscript for typos, grammar, and tense.<br><br>Reviewer 2:<br>I was pleased to review the resubmitted manuscript by Harris and co-workers, who have responded to my original review. The Authors have clarified a number of points regarding the RNAseq experiments including RNA extraction methods, and the tissue type that was used. More information has been added to the methods that would aid reproducibility. Additional statistics have been applied to Figures 1 and 5. Numerous formatting and grammatical changes have been made that improve the readability of the manuscript. Additional supporting references have been provided. While not all of my suggestions were included, I accept the authors responses to my original review. I have no further concerns and recommend the manuscript for publication in GigaScience.<br>-Response: We thank the reviewer for their careful considerations of our manuscript. The manuscript has been considerably improved thanks to the reviewer’s comments.<br><br>Reviewer 3:<br>I found that the Authors clearly improved the ms which might be suitable for publication<br>-Response: We thank the reviewer for their careful considerations of our manuscript.

	We especially thank the reviewer for comments on improving figures. The presentation of our work was improved by the reviewer's comments.<br><br>Reviewer 4:<br>-I saw the editor comments about appropriate data storage, but I disagree with those comments to the authors.<br>-Github is not a permanent repository and as such it's not true that it's the most appropriate place to share scripts for a publication. It is only suitable as a place for collaboration. As the authors make changes, the version of record for this manuscript will no longer be available, and the authors could delete it at any time. The publication versions should be separately reposited in a permanent repository. In my opinion, if a script is meant to be a version of record and also living, then a link to both the permanent repository and to GitHub can be given.<br>-I am not sure what is meant by 'large-scale' data. Figshare is a general use repository that I only recommended since the authors already were using it. It can host single files up to 5 gb in size, provides unlimited public space, and provides a DOI. So what exactly is unsuitable?<br>-Zenodo is another free option, and there is Data Dryad and the Data Commons.<br><br>-Response: We thank the reviewer for their careful considerations of our manuscript. We are happy to share our data and scripts in any way requested. Our intention was to use Github as a repository for a version of record, but we recognize that it is not a perfect solution. We are happy that Gigascience will host snapshots so that there is no potential for misuse. If the reviewer would like an additional home for the scripts we would be very happy to do that.
**Additional Information:**	
**Question**	**Response**
Are you submitting this manuscript to a special series or article collection?	No
**Experimental design and statistics**<br><br>Full details of the experimental design and statistical methods used should be given in the Methods section, as detailed in our Minimum Standards Reporting Checklist. Information essential to interpreting the data presented should be made available in the figure legends.<br><br>Have you included all the information requested in your manuscript?	Yes
**Resources**<br><br>A description of all resources used, including antibodies, cell lines, animals and software tools, with enough information to allow them to be uniquely identified, should be included in the Methods section. Authors are strongly encouraged to cite Research Resource Identifiers (RRIDs) for antibodies, model	Yes

organisms and tools, where possible.<br><br>Have you included the information requested as detailed in our Minimum Standards Reporting Checklist?	
**Availability of data and materials**<br><br>All datasets and code on which the conclusions of the paper rely must be either included in your submission or deposited in publicly available repositories (where available and ethically appropriate), referencing such data using a unique identifier in the references and in the “Availability of Data and Materials” section of your manuscript.<br><br>Have you have met the above requirement as detailed in our Minimum Standards Reporting Checklist?	Yes

Manuscript Click here to access/download;Manuscript;MV2017Leaves_GigaScience_rev2

Formatted: Normal

Style Definition: Heading 1

Style Definition: Heading 2

Multi-dimensional leaf phenotypes reflect root system genotype in grafted grapevine over the growing season

Zachary N. Harris[1,2*] (zachary.n.harris@slu.edu), Mani Awale[3] (maybd@mail.missouri.edu), Niyati Bhakta[1,2] (niyatisbhakta@gmail.com), Daniel H. Chitwood[4,5] (chitwoo9@msu.edu), Anne Fennell[6] (anne.fennell@sdstate.edu), Emma Frawley[1,2] (emma.frawley@wustl.edu), Laura L. Klein[1,2] (laura@leafworks.com), Laszlo G. Kovacs[7] (laszlokovacs@missouristate.edu), Misha Kwasniewski[3] (mtk5407@psu.edu), Jason P. Londo[8] (jason.londo@usda.gov), Qin Ma[9] (qin.ma@osumc.edu), Zoë Migicovsky[10] (zoe.migicovsky@dal.ca), Joel F. Swift[1,2] (joel.swift@slu.edu), and Allison J. Miller[1,2*] (allison.j.miller@slu.edu)

[1]Department of Biology, Saint Louis University, 3507 Laclede Avenue, St. Louis, MO, 63103-2010, USA

[2]Donald Danforth Plant Science Center, 975 North Warson Road, St. Louis, MO, 63132-2918, USA

[3]Division of Plant Sciences, University of Missouri, 135 Eckles Hall, Columbia, MO, 65211, USA

[4]Department of Horticulture, Michigan State University, East Lansing, MI, 48824, USA

[5]Department of Computational Mathematics, Science and Engineering, Michigan State University, East Lansing, MI, 48824, USA

[6]Department of Agronomy, Horticulture & Plant Science, South Dakota State University, Brookings, SD, 57006, USA

[7]Department of Biology, Missouri State University, 901S. National Avenue, Springfield, MO, 65897, USA

[8]United States Department of Agriculture, Agricultural Research Service: Grape Genetics Research Unit, 630 West North Street, Geneva, NY, 14456-1371, USA

[9]Department of Biomedical Informatics, The Ohio State University, 1585 Neil Ave, Columbus, OH, 43210

[10]Department of Plant, Food, and Environmental Sciences, Faculty of Agriculture, Dalhousie University, Truro, NS, B2N 5E3, Canada

* To whom correspondence should be addressed

Formatted: Normal

Abstract

Background: Modern biological approaches generate volumes of multi-dimensional data, offering unprecedented opportunities to address biological questions previously beyond reach due to small or subtle effects. A fundamental question in plant biology is the extent to which below-ground activity in the root system influences above-ground phenotypes expressed in the shoot system. Grafting, an ancient horticultural practice that fuses the root system of one individual (the rootstock) with the shoot system of a second, genetically distinct individual (the scion), is a powerful experimental system to understand below-ground effects on above-ground phenotypes. Previous studies on grafted grapevines have detected rootstock influence on scion phenotypes including physiology and berry chemistry. However, the extent of the rootstock's influence on leaves, the photosynthetic engines of the vine, and how those effects change over the course of a growing season, are still largely unknown.

Results: Here, we investigate associations between rootstock genotype and shoot system phenotypes using five multi-dimensional leaf phenotyping modalities measured in a common grafted scion: ionomics, metabolomics, transcriptomics, morphometrics, and physiology. Rootstock influence is ubiquitous but subtle across modalities with the strongest signature of rootstock observed in the leaf ionome. Moreover, we find that the extent of rootstock influence on scion phenotypes and patterns of phenomic covariation are highly dynamic across the season.

Conclusions: These findings substantially expand previously identified patterns to demonstrate that rootstock influence on scion phenotypes is complex and dynamic and underscore that broad understanding necessitates volumes of multi-dimensional data previously unmet.

Background

High-throughput data acquisition has afforded unprecedented capacity to quantify and understand plant form and function. Recent advances in imaging and computation have expanded our ability to measure plant traits or phenotypes ~~[1,2]~~[1,2], and to extend those comprehensive measurements into

Formatted: Normal

latent space phenotypes [3].[3]. Now broadly known as phenomics, this burgeoning field is characterized as the acquisition and analysis of high-dimensional phenotypic data at different hierarchical levels [4,5][4,5], often with an eye toward multiscale data integration. A holistic and hierarchical approach to plant phenotypic variation affords unique insights into plant evolution and how plants change over development and in response to environmental cues and horticultural manipulation.

A fundamental question in plant biology is how root systems influence phenomic variation in above-ground shoot systems including leaves, flowers, and fruits. Grafting, a common horticultural manipulation that joins the shoot system of one individual (the scion) with the root system of another individual (the rootstock), is commonly used in crop species to confer favorable phenotypes to commercial scions [6][6], including enhanced disease resistance [7,8][7,8], fruit quality, plant form [9][9], response to water stress [10][10], and growth on particular soils [11,12][11,12]. Because grafting often uses clonally propagated materials, it is possible to manipulate and replicate different combinations of root systems and shoot systems, offering a valuable experimental system in which root system impacts on shoot system phenotypes can be evaluated.

The European grapevine (*Vitis vinifera*) is among the most economically important grafted crops in the world. Grapevines are cultivated primarily for fruits used to make wine and juice, as well as for table grape and raisin production. Grafting in grapevines became widespread in the mid-1800’s following the accidental introduction of the root-feeding aphid phylloxera from its native North America into Europe, where it began attacking the roots of European grapevines [13].[13]. Because European grapevines often do not survive phylloxera infestation, in regions where phylloxera has been introduced most grapevine cultivation consists of European grapevines grafted to rootstocks derived from phylloxera-resistant North American *Vitis* species including *V. berlandieri*, *V. riparia*, and *V, rupestris*, and their hybrid derivatives. In addition to grapevines, more than 70 major perennial crops are grafted including many fruit trees and vines [9].[9]. Grafting decouples the breeding of shoot systems and root systems, with selection in plants targeted for use as scions focusing primarily on fruit phenotypes, and

Formatted: Normal

selection in plants targeted for use as rootstocks focused on below-ground biotic and abiotic stress resistance, as well as their impacts on shoot system phenotypes.

The effects of grafting in grapevine show a remarkable breadth of scion response patterns. For example, a study of *Vitis vinifera* cv. ‘Cabernet Sauvignon’ grafted to different rootstocks identified transcriptome reprogramming in the scion of grafted plants; this appeared to be a general effect of grafting to a rootstock and was not rootstock-specific ~~[14].~~[14]. In contrast, other studies have found signatures of rootstock genotype in the transcriptome in early berry development, although this distinction was lost in later development ~~[15,16], but see [17].~~[15,16], but see [17]. Comprehensive phenomic analyses, including those that link transcriptome data with other high-throughput phenotyping assays, offer an opportunity to expand understanding of rootstock effects on grapevine shoots. In one study, leaves of the *V. vinifera* cultivar ‘Gaglioppo’ showed variation in stilbene and abscisic acid concentrations due to rootstock genotype, as well as differences in transcriptional profiles ~~[18].~~[18]. Likewise, gene expression, ion concentrations, and leaf shape in the cultivar ‘Chambourcin’ varied in response to rootstock genotype ~~[18,19].~~[18,19]. Collectively, these studies suggest the impacts of grafting are diverse and may vary over the course of vine development. However, to date few studies have surveyed multiple high-dimensional scion phenotypes to understand rootstock influence on shoot system phenotypes over the course of the growing season or the extent to which grafting effects on the scion covary with one another.

Leaves are the photosynthetic engine of the organism and a primary site for perception and response to environmental change. Grapevine leaves have been used for centuries as markers of species and cultivar delimitation, developmental variation, disease presence, and nutrient deficiency ~~[20,21].~~[20,21]. More recently, analysis of grapevine leaf morphology has identified genetic architecture of leaf shapes ~~[22]~~[22], developmental patterns across the season ~~[23]~~[23], and signatures of evolution in the grapevine genus ~~[24].~~[24]. Grapevine leaves respond to stress through gas and water exchange with the atmosphere ~~[25,26]~~[25,26] and have been shown to differentially partition the ionome depending on their position on the shoot ~~[19]~~[19] and their rootstock genotype ~~[19,27,28]~~[19,27,28]. The volume of

Formatted: Normal

work on grapevine leaves provides a foundation for the analysis of phenomic variation in a vineyard over a season in response to grafting.

In this study, we investigate effects of grafting on high dimensional leaf phenotypes of the hybrid cultivar ‘Chambourcin’ over the course of the growing season. We quantify leaf elemental (ion) concentrations, metabolite abundance, gene expression, shape, and vine physiology in a replicated rootstock trial where the hybrid grapevine cultivar ‘Chambourcin’ is growing ungrafted and grafted to three different rootstocks. The four root-shoot combinations (‘Chambourcin’ ungrafted, ‘Chambourcin’ grafted to three different rootstocks) are replicated 72 times in a randomized block experimental design with an irrigation treatment (Supplemental Figure 1). Phenotypic data, data that describe variation for a particular trait within a particular modality, were collected either on the full 288-vine set (ion concentrations, leaf shape) or on a subset of 72 vines (the 72-vine set; metabolite abundance, gene expression, vine physiology). Using data collected at three time points that span the growing season (anthesis, veraison, and harvest), we show that all phenotyping modalities (ionomic, metabolomic, transcriptomic, morphometric, and physiology phenotypes) reflect subtle but ubiquitous responses to grafting and rootstock genotype. Rootstock effects on shoot system phenotypes were often dynamic across the season, suggesting that accounting for seasonal variation could enhance our understanding of grafting effects in viticulture.

Data Description

Leaf Ionomics

The ionome describes elemental composition of a tissue at a particular time point ~~[29].~~[29]. Three leaves per vine were collected from the 288-vine set at three seasonal time points: anthesis (~mid May), veraison (~late July), and harvest ~mid September). Leaves were sampled from a single shoot and included the youngest fully opened leaf at the shoot tip, the approximate middle leaf, and the oldest leaf at the shoot base. Teams were deployed in the vineyard so that multiple vineyard rows were being sampled

Formatted: Normal

concurrently. As such, ‘block’ represented unmeasured spatial variation, but did not strictly correlate with time of sampling due to the nature of sampling (see Methods). Whole leaves were placed in zip-lock bags in the field and stored in a cooler on ice packs, scanned for leaf shape analysis in the lab (see Leaf Shape) and then dried in coin envelopes at 50°C for one to three days for elemental analysis. Between 20 and 100 mg of leaf tissue was acid digested and 20 ions were quantified using inductively coupled plasma mass spectrometry (ICP-MS) following standard protocol of the Donald Danforth Plant Science Center (DDPSC) Ionomics Pipeline ~~[30,31].~~[30,31]. Ion quantifications were corrected for internal standard concentrations, instrument drift and by initial sample mass. The output of the Pipeline contained estimated concentrations of each of the following 20 elements: Al, As, B, Ca, Cd, Co, Cu, Fe, K, Mg, Mn, Mo, Na, Ni, P, Rb, S, Se, Sr, and Zn. For each ion concentration, we computed z-score distributions and used those values as the basis for linear models. Following convention, non-standardized values were used for machine learning analysis.

Leaf Metabolomics

The metabolome comprises small mostly organic molecules present in a tissue and represents a catalogue of the products of metabolic processes ~~[32,33].~~[32,33]. Metabolomic analysis was completed at veraison (the onset of fruit ripening) and immediately prior to harvest for the 72-vine set. For each vine, three mature leaves were sampled from the middle of a single shoot and immediately flash frozen in liquid nitrogen in the field to capture the metabolic state of the leaves when attached to the vine. Leaves were sampled by a single team near midday in row and block order, ensuring that ‘block’ captured both unmeasured spatial variation and temporal variation over the sampling window (see Methods). Frozen leaves were transported to the University of Missouri Enology Lab on dry ice and stored at -80˚C. Following the protocol of ~~[34]~~[34], whole leaves were manually ground in liquid nitrogen with a mortar and pestle, 0.5g of powder was weighed into a centrifuge tube, 1.5ml of 1:1 MeOH: ACN was added. Samples were vortexed to suspend leaf particles and sonicated for 20 minutes in an ice bath. After

Formatted: Normal

extraction, samples were centrifuged for 10 minutes at 3,000 g and filtered with a 0.22 PTFE syringe filter into a 1.5ml sample vial before injecting into a Waters XEVOTM QToF LCMS system (Waters Corporation, Milford, MA, USA). Chromatographic separation was achieved using a Waters Acquity TM Ultra Performance LC H-Class system (Waters Corporation, Milford, MA, USA) equipped with Waters Acquity BEH C18 column (2.1mmx150mm and 1.7um particle size) and a diode array detector. Samples were injected in random order across the sampling periods. The injection volume was set at 2.5ul and the flow rate was set at 0.4 ml/min. The mobile phase consisted of 0.1% formic acid in water (solvent A) and 0.1% formic acid and 5% water in acetaldehyde (solvent B) and the gradient was as follows: 100% A for 0.5 min; 0.5-18min increased to 99% B; 18-19 min. held at 99% B; mobile phase was re-equilibrated for 2 min between runs. Diode array was monitored at 225-500nm. Mass spectrometry was performed on a XevoTM QTof (Waters Corporation, Milford, MA, USA). The electrospray ionization (ESI) was operated in both positive or negative ionization modes in separate runs. The scan range was set as m/z 50-1500 with 0.2 sec accumulation time. MS settings were as follows: capillary voltage was 2.5kV; cone voltage ramped from 20-40V; collision energy was set to 6V; detector voltage was set to 1950V; desolvation gas was set to 1000 L/hour; cone gas was set to 50 L/hr; source temperature was 120℃ and desolvation temperature was set at 550℃.

LC-MS instrument files were converted to .cdf format and uploaded to XCMS online ~~[35]~~[35] for chromatogram normalization and feature detection via "single job" parameters. The 661 identified metabolomic features were used as the basis of a principal components (PC) analysis. The top 20 PCs were treated as distinct phenotypes to model according to the experimental design. In PCs that varied significantly by rootstock, features that loaded more than 1.96 standard deviations above or below the mean were fit independently with the same model design.

Leaf Gene Expression

The youngest fully-opened leaves on two shoots were collected from each plant of the 72-vine set (see Study Design). The two leaves, which were distinct from leaves used for ionomics, leaf shape,

Formatted: Normal

metabolomics and physiology data collection, were pooled for RNA sequencing. Leaves were sampled by a single team near midday between 10AM and 2PM in row order, ensuring that 'block' and 'row' accounted for unmeasured spatial variation and temporal variation over the sampling window (see Methods). Samples were sequenced using 3'-RNAseq, a method ideal for organisms with reasonably characterized reference genomes ~~[36].~~[36]. Total RNA was extracted from plant tissues using the Sigma Spectrum Plant Total RNA kit with modification of the addition of 2% PVP40 to the extraction buffer to decrease phenolic inhibitors. All RNA extractions were checked for quality control using a Nanodrop. Sequencing was conducted using the Illumina NextSeq500 platform which returned single-end 86 bp reads. To accommodate the large number of samples in this study, we opted to obtain fewer reads per sample, which might have limited our ability to detect differential expression in lowly-expressed genes. The first 12 nucleotides from each read were trimmed to remove low-quality sequences using Trimmomatic (options: HEADCROP:12; ~~[37]).~~[37]). Low quality trimmed reads were additionally identified based on overrepresentation of kmers and removed using BBduk (April 2019 release) ~~[38].~~[38]. Trimmed and QC-controlled reads were mapped to the 12Xv2 reference *Vitis vinifera* genome ~~[39,40]~~[39,40] using STAR (v2.7.2b) ~~[41]~~[41] with default alignment parameters. RNAseq read alignments were quantified using HTSeq-count (v0.11.2) ~~[42]~~[42] and a modified version of the VCost.v3 reference *V. vinifera* genome annotation ~~[40].~~[40]. To capture mis-annotated gene body boundaries in the genome, all gene boundaries in the annotation were extended 500 bp.

Variation in gene expression was assessed using two methodologies. First, we identified individual genes which responded to specific factors in the experimental design using DESeq2 (v1.24.0) ~~[43].~~[43]. Each gene was fit with the model "~ Block + Irrigation + Phenology_Rootstock" where the 'Phenology_Rootstock' model term was used to understand the potential interaction of phenology and rootstock. Genes were filtered to a gene set that included only genes with a normalized count greater than or equal to two in at least five samples. To check the validity of our expression results, we assayed two classes of housekeeping ~~gene~~genes (Ubiquitin-domain and actin-family) and eight previously annotated circadian genes ~~[44]~~[44] (Supplemental Figure 2). Differentially expressed genes were identified for each

Formatted: Normal

pairwise contrast in the model. Second, we used principal component analysis (PCA) to collapse variation in co-expressed genes into fewer dimensions. Normalized count-filtered genes from DESeq2 were transformed using the variance stabilizing transformation (VST; ~~[45]~~[45]) and input into a PCA. We then analyzed the top 100 PCs in the context of the broader experimental design. We previously showed that the transcriptome varied by the time of collection and was potentially interacting with the rootstock effect ~~[19].~~[19]. Moreover, the other modalities in this study point to weak if any effects from the irrigation treatment (see Supplemental Note 1). Due to the nature of the vineyard design, we could not identify both irrigation and time effects (marked by row) in a single model (irrigation and row are collinear; see Study Design). To approximate the impact from time of collection (row) in the vineyard on gene expression, linear models were first fit to remove variation imparted by irrigation from each of the top 100 PCs. The residuals were then used as the basis for linear models and machine learning analysis.

Leaf Shape

All leaves from a single shoot directly emerging from a trained cordon were collected from each vine in the 288-vine set at anthesis and veraison. At harvest, we collected only the oldest (first emerging leaf), middle (estimated from the middle of a whole shoot), and youngest (smallest fully emerged leaf at the shoot tip, >1cm). Leaves were collected approximately in row order (from south to north) and stored in a cooler. Each leaf was imaged using an Epson DS-50000 scanner in color against a white background at 1200 DPI and written as JPEG formatted images. Following scanning of leaves for leaf shape analysis, the oldest, middle, and youngest leaves were dried and used to estimate leaf elemental composition (see Ionomics). As the leaf shape samples and ionomics samples were identical, ‘block’ represented unmeasured spatial variation, but did not strictly correlate with time of sampling (see Methods). While all leaves were collected from a single shoot, only the oldest, middle, and youngest leaves were used in this analysis.

We assessed leaf shape using Generalized Procrustes Analysis (GPA) of landmarks. For the three leaves per vine used in leaf shape analysis, 17 homologous landmark features were identified ~~[22].~~[22].

Formatted: Normal

The GPA-rotated coordinate space was used for all subsequent statistical analysis including PCA in order to summarize variation in leaf shape ~~[46].~~[46]. From the PCA, we extracted the top 20 PCs and fit linear models and machine learning models to describe variation.

Vine physiology

Intracellular CO_2 concentration, stomatal conductance and leaf transpiration rate were measured at midday (each measured simultaneously between 10am to 1pm) on one fully expanded sun-exposed leaf for each of the vines in the 72-vine set. Physiology measurements were taken in row order ensuring that 'block' correlated with temporal variation over the sampling window. Measurements were taken using an LI-6400XT Portable Photosynthesis system coupled with a pulse amplitude-modulated (PAM) leaf chamber fluorometer (Li-Cor, Inc., Lincoln, NE, USA) with the following parameters: incident photosynthetic photo flux density level of 1000 µmol m−2 s−1 generated by a red LED array and 10% blue light to maximize stomatal opening, CO_2 mixer of 400 µmol/s, fixed flow of 300 µmol/s, and ambient leaf and block temperature. Soil moisture was measured for each plant in the 72-vine set using a fieldScout TDR 300 Moisture meter equipped with 20 cm rods (Spectrum Technologies, Inc. Aurora, IL, USA). Midday stem water potential was measured using a pressure bomb/chamber (PMS Instrument Co., Albany, OR, USA) after enclosing the leaves in an aluminum foil bag for at least 15 minutes to equilibrate the water potential of the xylem in the stem to that attached leaf (for a discussion on equilibration time, see ~~[47,48]).~~[47,48]).

Formatted: Font: Cardo

Analyses

Leaf ionome

To characterize the leaf ionome over the growing season, we sampled the youngest, middle, and oldest leaf from a single shoot from each of the vines within the 288-vine set at three phenological stages

Formatted: Normal

and measured the concentrations of 20 ions in each leaf individually. Bivariate correlations showed that ion concentrations are not independent of each other, but that the strength and direction of relationships between ions vary with respect to phenological stage and leaf position (Supplemental Figure 3). As such, we fit independent linear models to each ion. Leaf position, phenological stage, or the interaction of phenological stage and leaf position explained the highest amount of variation for most ions (Figure 1A-B). Many ions significant for the interaction showed a clear signal of leaf position at anthesis and veraison, and either no explainable variation or muted variation at harvest. For example, calcium (Figure 1B) varied with leaf position (22.7% variation explained; $p < 1e\text{-}05$), phenology (24.0%; $p < 1e\text{-}05$), and their interaction (7.4%, $p < 1e\text{-}05$). All possible pairwise combinations of leaf position were significantly different at anthesis, and both the youngest and middle leaves were different from the oldest leaves at veraison and harvest. In the case of potassium (Figure 1B), significant variation was explained by leaf position (16.1%; $p < 1e\text{-}05$), phenology (19.6%; $p < 1e\text{-}05$), and their interaction (10.6%; $p < 1e\text{-}05$). However, post-hoc comparisons of phenology-wise mean calcium concentrations showed that differences were present only at anthesis and veraison.

Rootstock genotype showed remarkable influence on the composition of the leaf ionome. All ions except aluminum, sodium, and zinc were significant for rootstock as a single fixed effect (Figure 1A). Rootstock explained between 0.4% (rubidium; $p = 3.2e\text{-}05$) and 14.3% (nickel; $p < 1e\text{-}05$) of variation ion concentrations (Figure 1A). For some ion concentrations (such as cobalt and nickel), significant variation was explained by the interaction of rootstock and phenology; this pattern was observed mostly in ions that responded weakly to the interaction of leaf position and phenology. These ions showed similar patterns to the leaf position by phenology interaction where a clear signal was exhibited at anthesis and veraison then ~~is~~was either absent or muted at harvest. For example, cobalt was most abundant in '1103P'-grafted vines at anthesis (Figure 1C). At veraison, both '1103P'-grafted and 'SO4'-grafted had elevated concentrations compared to Ungrafted and '3309'-grafted vines. However, by harvest, cobalt concentration variation was muted and only 'SO4'-grafted vines showed evidence of elevated concentration. Similarly, nickel showed significant variation partitioned into the rootstock by the phenology effect (Figure 1C). Both anthesis and

Formatted: Normal

veraison show reduced nickel concentration in '1103P'-grafted vines and elevated concentrations in 'SO4'-grafted vines. However, at harvest, no comparisons are significant.

Machine learning on ion concentrations confirms that the leaf ionome contains a signature from the rootstock genotype and the interactions of rootstock genotype with phenology and leaf position. A random forest model trained to predict rootstock showed an overall accuracy of 75.2% (Figure 1D). Ions important for this classification were nickel (Mean Decrease in Accuracy (MDA)=0.089), molybdenum (MDA=0.058), and magnesium (MDA=0.054), corroborating the rootstock term's significance in the linear models. Notably, when we trained a model to simultaneously predict rootstock and phenological stage, rootstock prediction accuracy increased appreciably (Figure 1E). For example, the ability of the model to detect ungrafted vines (the balanced accuracy of ungrafted predictions) improved from 81.7% accuracy overall to 91.1% accuracy at anthesis and 85.9% at harvest. Generally, performance at veraison matched the rootstock-only model performance. The ions most important for this joint (rootstock/phenological stage) prediction were nickel (MDA=0.167), phosphorus (MDA=0.110), and strontium (MDA=0.065). The rootstock by phenology model term was significant in the linear models for these ions, but was not a largest descriptor of variation. The joint prediction of rootstock and leaf position performed substantially better than chance ($p < 1e\text{-}05$), but accounting for leaf position did not improve rootstock prediction as was the case in the joint prediction of rootstock and phenology (Figure 1F). Ions important for this classification were sulfur (MDA = 0.051), rubidium (MDA = 0.051), and nickel (MDA = 0.049).

Leaf metabolomics

We performed untargeted metabolomics on leaves from the 72-vine set at veraison and harvest, quantifying the concentrations of 661 metabolites (Figure 2). The top 20 PCs accounted for a total of 67.3% of the total metabolomic variation, with the top three capturing 23.1%, 9.2%, and 6.2%, respectively. Individual PCs after the top 20 explained less than 0.82% of the metabolome. Linear models for each of the top 20 PCs found that the strongest drivers of variation in leaf metabolomics were

Formatted: Normal

phenology and temporal blocking factor. For example, 90.6% of variation on PC1 was due to phenology ($p < 1e-05$; Figure 2A). PC2 primarily reflected the interaction of phenology and temporal block (26.4%, $p < 1e-05$) and temporal block as a main effect (18.9%, $p < 1e-05$). The patterns of variation attributable to PC2 were similar in PCs 3-10 (Figure 2A).

PC17 was controlled by rootstock as a main effect (18.5%, $p < 1e-03$; Figure 2B). On PC17, ungrafted vines were significantly different from vines grafted to ‘3309C’ ($p = 0.02$) and ‘SO4’ ($p < 1e-05$). Vines grafted to ‘1103P’ were also significantly different from vines grafted to ‘SO4’ ($p = 0.009$). Metabolites that loaded more than 1.96 sd from the mean loading on PC17 were extracted and independently fit to additional linear models. We identified four metabolite features (M374T1 [rt = 1.33, m/z = 374.1146], M117T1 [rt = 0.61, m/z = 117.0583], M175T1_1 [rt = 0.87, m/z = 175.1269], and M333T1_3 [rt = 0.71; m/z = 333.1582]) which were influenced by rootstock as a main effect and the metabolite (M112T1 [rt = 1.48, m/z = 112.0061]) which was influenced by the interaction of rootstock genotype and phenological stage. At this time, the identification of these features remains unknown.

Linear discriminant analysis confirmed that many experimental factors likely influence the metabolome. For example, when trained to maximize variation between classes of rootstocks, the model identified a space that weakly separates ‘1103P’-grafted and ‘SO4’-grafted vines from ungrafted and ‘3309C’-grafted vines (LD1) and separates ‘3309C’-grafted vines from other classes (on LD2) (Figure 2C). Despite this, machine learning showed minimal predictability for any class other than phenology, which was predictable with an accuracy of 100% for withheld samples. Rootstock genotype based on the metabolome was not predictable with accuracy only marginally better than chance (34.6%).

Gene Expression

We performed 3’-RNAseq on the youngest fully-opened leaves of the 72-vine set at three time points (Figure 3). On average, each sample contained 4.1 million 3’-reads and measured the expression of 17,852 genes. Overall, we identified variation in 23,460 genes that had a DESeq2-normalized count greater than two in at least five samples. We computed the expression of two classes of housekeeping

Formatted: Normal

genes, and showed that they are generally stable across samples over phenological time (Supplemental Figure 2). We noted that some variation is expected for housekeeping genes; see, for example, ~~[49].~~[49]. Moreover, we showed that patterns of previously annotated circadian genes conform to expected results over the sampling window. For example, predicted orthologs of *LHY* and *RVE1* are correlated and decreasing over our sampling window, and a predicted *TOC1* ortholog is invariant. The results of these analyses provide general confidence in the gene expression data presented here.

Using a traditional differential expression analysis framework based on established DGE software (Deseq2), all genes returned as significantly differentially expressed by rootstock appeared to be false positives, evidenced by a single extreme outlier altering group means. Hierarchical clustering of the 500 most variable genes after variance stabilizing transformation (VST) showed strong latent structure in the transcriptome and that most variation in the transcriptome was explained by the phenological stage (Figure 3A). The top 100 PCs on the VST-transformed gene counts accounted for nearly 92.3% of variation in the transcriptome. Linear models on each of the top 100 PCs indicated that 82.4% and 61.4% of the variation on PC1 and PC2 respectively were attributable to the phenological stage (Figure 3B-C). Row was also a significant descriptor of variation as a single, fixed effect and in interactions with rootstock and phenological stage. For example, row accounted for 36.0% and 43.3% of the variation on PC4 and PC6, respectively. Interacting with the phenological stage, row accounted for >10% of variation on 17 additional PCs.

Patterns of gene expression identified through LDA corresponded to phenological stage, vine row, and rootstock. LDA separated phenological stages into three distinct, non-overlapping groups in the space spanning LD1 and LD2 (Supplemental Figure 4). When trying to separate rows into distinct classes, the model converged on a ‘horseshoe’ shape in the LD1- LD2 space (Figure 3D), suggesting either a circadian topology to the transcriptome or continuous spatial variation over the vineyard ~~[50].~~[50]. LD1 maximized the variation between row 8 (sampled early in the day) and row 16 (sampled a few hours later). LD2 maximized the separation of both rows 8 and 16 with row 12 (the row sampled in the middle of the sampling window). A model trained to separate rootstock classes (Figure 3E) showed that LD1

Formatted: Normal

separated the rootstock 1103P from other rootstock genotypes, and LD2 primarily separated the rootstock '3309C' from ungrafted vines (Supplemental Figure 4).

Formal machine learning on gene expression PCs largely supported the linear models. A random forest trained to predict phenological stage classified testing samples with 92.9% accuracy. Anthesis was the most predictable class with a balanced accuracy of 100%; veraison and harvest displayed balanced accuracies of 92.7% and 92.4%, respectively. The PCs most important in phenology prediction were PC1 (MDA = 0.16) and PC2 (MDA = 0.12). Gene expression PCs were unable to predict rootstock, with a total prediction accuracy of 23.4%. While no features were especially important in the prediction processes, PC44 showed the largest mean decrease in Gini impurity corroborating its signal in the linear models.

Leaf shape

We collected leaves from the 288-vine set at three time points and landmarked a total of 2,422 leaves (Figure 4). Homologous leaf landmarks were used for Generalized Procrustes Analysis (GPA). PCA on the GPA-rotated coordinates revealed ~97.2% of the total shape variation was captured by the top 20 principal components with PC1, PC2, and PC3 explaining 24.1%, 19.0%, and 13.3% of the variation respectively. Lower values on PC1 primarily capture leaves with shallow petiolar sinuses and short midvein distance from the depth of the superior sinus to the top of the midvein, whereas higher values on PC1 capture the opposite (Figure 4A). Similarly, lower values on PC2 capture deep petiolar sinuses combined with very shallow superior sinuses, and vice versa for higher values. PC3 primarily captures asymmetry (Figure 4A).

In total, 5.76% of variation on PC1 was explained by the experimental design. Of this, variation in leaf shape was explained by phenology (2.63%; padj $< 1e-05$), then rootstock (0.95%; padj < 0.001), leaf position (2.61%; padj $= 0.03$), and the interaction of phenology and leaf position (0.62%; padj $= 0.009$) (Supplemental Figure 5A). Post-hoc mean comparisons on PC1 showed that shapes of leaves from ungrafted vines were significantly different from leaves of vines grafted to 1103P ($p < 0.001$), 3309C (p <

Formatted: Normal

0.001) and SO4 ($p < 0.001$) (Supplemental Figure 5B). Moreover, PC1 captured subtle variation in the leaf position by phenological stage interaction where middle leaves showed significant differences between anthesis and veraison ($p < 1e\text{-}03$), and the oldest leaves showed significant differences when comparing anthesis to veraison ($p < 1e\text{-}05$) and anthesis to harvest ($p < 1e\text{-}03$).

For PC2, 61.4% of variation could be assigned to an experimental factor. This included significant variation from leaf position (46.9%, padj $< 1e\text{-}05$), phenology (1.4%; padj $< 1e\text{-}05$), and the interaction of leaf position and phenology (12.05%; padj $< 1e\text{-}05$; Figure 4D). Specifically, younger leaves tended to have shallower sinuses and exaggerated superior sinus depths (higher values on PC2), whereas older leaves tended to develop deeper petiolar sinuses and more shallow superior sinuses (lower values on PC2). The degree of this separation decreased across the season, and the shapes converged on the mean leaf shape on PC2, consistent with the middle leaf at all three phenological stages. PC2 additionally reflected the interaction of leaf position and rootstock (0.22%; $p = 0.04$; Supplemental Figure 5B), but post-hoc comparisons did not find any significant pairwise comparisons.

Machine learning on the GPA-rotated coordinate space identified moderate division of developmental and phenological classes. Random forest models could predict the leaf position with 73.1% accuracy, with the most important feature being the y-component of the leaf apex (MDA = 0.051). A model trained to predict phenology performed at 64.3% with the most important features being the x-components of the points corresponding to superior sinus depth (left sinus MDA = 0.030, right sinus MDA = 0.019). A model trained to predict rootstock performed only marginally better than chance at 28.1% accuracy.

Vine physiology

We measured intracellular CO_2 concentration (C_i), stomatal conductance (g_s), leaf transpiration, water potential (ψ), and soil moisture for the 72-vine set (Figure 5). Each physiological phenotype varied significantly across phenology and the block by phenology interaction (Figure 5A). For example, at harvest, we observed specific differences in leaf CO_2 concentration (A vs C: $p=0.003$; B vs C: $p=0.002$)

Formatted: Normal

and leaf transpiration (A vs B: $p < 1e-03$; A vs C: $p < 1e-05$; B vs C: $p < 1e-05$). Leaf transpiration and stomatal conductance varied significantly with the interaction of rootstock and phenology. A post-hoc comparison of means showed that leaf transpiration and stomatal conductances were elevated in 'Chambourcin' vines grafted to '1103P' at veraison as compared to leaves of ungrafted vines (leaf transpiration: $p = 0.001$; stomatal conductance: $p = 0.002$ Figure 5B-C).

Phenomic covariation

Four leaf phenotyping modalities consisted of 10 or more measured phenotypes and were measured for all plants in the 72-vine set (leaf ionome, leaf metabolomics, gene expression, leaf shape). Using these data, we explored the extent to which different phenotypes (within and between modalities) covaried over phenology and rootstock genotype (Figure 6; Supplemental Figure 6; Supplemental Figure 7). Within each phenotyping modality, we summarized the primary dimensions of phenotypic variation using PCA (see Methods), so as to not weigh any modality too heavily. From each PCA, we extracted the top 10 PCs, which explained a total of 88.9% of variation in the ionomics PCA (iPCA), 55.9% of the variation for the metabolomics PCA (mPCA), 74.8% of the variation in the gene expression PCA (gPCA) and 87.9% of the variation in the leaf shape PCA (sPCA).

Pairwise correlations of each PC within each phenological stage showed diverse correlation magnitudes and directions both within a phenotyping modality and between phenotyping modalities (Figure 6A-C; Supplemental Figure 6). Generally, the strongest relationships were between PCs within phenotyping modalities. For example, the strongest correlations identified were between gene expression PCs gPC1 and gPC2 at anthesis ($r = 0.85$, CI = [0.81, 0.87]; Supplemental Figure 6A, and metabolomics PCs mPC1 and mPC2 at harvest ($r = -0.78$, CI = [-0.82. -0.76]). Correlations between modalities represented a diversity of responses across phenological stages. For example, the correlation between gene expression gPC4 and shape sPC3 was similar across the phenological stages, but only the correlation at veraison was significant ($r = 0.41$, CI = [0.34, 0.47]; Supplemental Figure 6B). Correlations such as

Formatted: Normal

between metabolomics mPC3 and gene expression gPC6 were similar and significant at both veraison (r = -0.44, CI = [-0.50, -0.37]; Supplemental Figure 6C) and harvest (r = -0.37, CI = [-0.45, -0.28]; Supplemental Figure 5C). While many correlations varied over the course of the season, some relationships entirely shifted in direction. For example, the correlation between metabolomics mPC3 and mPC6 shifted from a positive significant relationship (r = 0.58, CI = [0.52, 0.63]) at veraison to a negative significant relationship at veraison (r = -0.66, CI = [-0.73, -0.59]) (Supplemental Figure 6D).

Pairwise comparisons of PCs within each rootstock genotype show a suite of latent phenotypes with significant presence/absence variation in significant correlations. Where each phenological stage showed modularity by phenotyping modality, variation over rootstock genotype shows a strong ionomics module with latent combination of other modalities interspersed (Supplemental Figure 7). For example, in ungrafted vines, metabolomics mPC1 was correlated with four PCs from the ionome (Supplemental Figure 7A). Each of the other rootstock genotypes had dramatically different topologies with the ionome tending to be more connected within the ionome and connected to other modalities only on the periphery (Supplemental Figure 7B-D). Examples of presence/absence variation were shown in small modules of two latent phenotypes that were present in only one rootstock genotype. For example, in the ungrafted vines, the correlation between gene expression gPC4 and metabolomics mPC3 was significant (r = -0.58, CI = [-0.65, -0.51]) and, in ‘1103P’-grafted vines, the correlation between metabolomics mPC3 and shape sPC6 (r = 0.59, CI = [0.53, 0.70]) was significant.

Discussion

In this study, we used grafted grapevines as an experimental system for characterizing root system impacts on multi-dimensional leaf phenotypes over the course of a growing season. We detected ubiquitous but subtle effects of the root system on all assayed modalities, and demonstrated that rootstock influences on leaf phenotypes can be specific to the vine’s developmental stage. The strongest signals of

Formatted: Normal

rootstock influences on leaves were observed in the ionomics dataset, phenotypes for which the root system has a noted and well-understood role.

Phenology explains significant variation in all leaf phenotypes

The timing of sampling or phenological stage of the vines (anthesis, veraison, harvest) was the strongest driver of phenomic variation for most leaf phenotypes. For example, all 20 ions varied with phenology and most ions showed that phenology, or the interaction of phenology with leaf developmental position, was the strongest source of variation (Figure 1). Nearly one third of all measured transcripts responded to seasonal variation, and the strongest effects on the transcriptome were phenology and row, a correlate for the time within a three-hour sampling window. The only phenotype for which phenology was not the most explanatory factor is leaf shape. Consistent with previous studies [23][23], we confirm that most of the leaf shape variation reflects development along a single shoot, but much of this variation is explained via interaction with phenology. These data highlight the dynamic nature of biological processes taking place within grapevines over the course of a season.

The seasonal component to grapevine phenomic variation is a subject of much research, especially in the berry. In studies designed to quantify molecular underpinnings of terroir, seasonal variation was identified as the strongest signal in the metabolome [51–54].[51–54]. Several studies have characterized transcriptomic variation over the course of the season. For example, in conjunction with metabolomics, seasonal variation of berry development was used to identify transcriptomic and metabolomic developmental markers in ‘Corvina’ [55].[55]. Follow-up analysis showed that nearly 18% of transcripts varied seasonally [56].[56]. Grapevine leaf shape also varies tremendously over the growing season [23][23] and is stable over multiple growing seasons; interestingly, grapevine leaves are patterned in the previous year, and the climate of the season in which the leaves were patterned influence aspects of leaf shape [57,58].[57,58].

Formatted: Normal

Grafting and rootstock genotype exhibit a complex and subtle signal on leaf phenotypes

Consistent with previous studies, we confirm that grafting, as well as rootstock genotype, has a complex effect on phenomic variation in the scion (the grafted shoot system). Most notably, we show that the rootstock to which a scion is grafted influences ion concentrations in leaves. Rootstock genotype is predictable from ion concentrations in the leaves, and this signal is strengthened when phenological stage is included in the model. For example, we previously showed that nickel concentration was elevated in vines grafted to the rootstock 'SO4' ~~[19].~~[19]. At a similar point in the season, we observe the same pattern, but by harvest, nickel was almost entirely excluded from the leaf. This suggests that the biological implications of this differential uptake could be missed if not surveyed across the season. We also confirm that rootstock genotype influences the metabolome of grafted grapevine, in some cases in a season-specific manner. In the transcriptome, PCA was able to identify dimensions of variation that were significantly described by rootstock and the interaction of rootstock and time of day, confirming prior observations ~~[19].~~[19]. Patterns of gene expression were associated with rootstock in some analyses; for example, supervised methodologies identified linear discriminants in the PC space that separated gene expression ~~patters~~patterns of some rootstock genotypes. However, gene-by-gene analysis found no genes modulated by rootstock genotype, or even just from the act of grafting that were not driven entirely by a single outlier. We suspect these results are due, at least in part, to the strength of the phenology effect overpowering more subtle variation imparted by rootstock genotype. Finally, of the physiology phenotypes we measured, leaf transpiration and stomatal conductance were higher in vines grafted to '1103P' in the middle of the season. Through these analyses, we have identified subtle but ubiquitous effects of rootstock genotype on shoot system phenotype across modalities, and have shown that the impact of grafting on leaf phenomic variation varies from one phenotype to the next.

Understanding the rootstock genotype influence on shoot system phenotypes is a growing area of research, especially in grapevine. For example, in 'Cabernet Sauvignon', grafting increased ion uptake globally and some rootstock genotypes provide a clear signal in the scion ~~[28,59].~~[28]. The wild *Vitis* species from which the rootstocks were derived from (*Vitis berlandieri*, *V. riparia*, and *V. rupestris*) differ

Formatted: Normal

in root architecture, preferred soil substrate, and genetic background; however, the specific aspects of their biology that contribute to differences in ion uptake are not known [27]. To our knowledge, there is not yet a strong causal link between the micronutrient component of the ionome and factors of vine growth or development that might influence traits like wine quality. However, it is noted that macronutrient deficiencies can have negative effects on such traits ~~[60,61]~~[59,60] and can be mediated by rootstock ~~[62]~~[61]. This suggests a strong understanding of the rootstock influence on the vine's ionome is warranted, and more work needs to be done to establish these relationships. Similarly, the metabolome is a key driver of the formation of the graft junction and some key metabolites could be responsible for graft incompatibility ~~[63].~~[62]. Building on this work, targeted metabolomics showed two classes of metabolites, flavanols and stilbenes, were differentially abundant at graft junctions and in the rootstocks of 'Cabernet Sauvignon' vines one month after grafting ~~[64].~~[63]. However, flavanols were not differentially abundant in the scion, but scion stilbene concentrations were apparently controlled by rootstock genotype. The effect of rootstock genotype on the scion transcriptome is perhaps the most varied. For example, 'Cabernet Sauvignon' shoot apical meristems show no effects by rootstock genotype ~~[14]~~[14], but berries of the same cultivar do, although the effect is tempered by seasonal variation ~~[15].~~[15]. Variation in 'Chambourcin' leaf shape was also driven by rootstock genotype, especially in conjunction with differences in irrigation ~~[19].~~[19]. Collectively, these studies all suggest that rootstock genotype influences scion phenotypes, but those effects will vary by phenotype, scion genotype, and perhaps other experimental conditions.

Data presented here confirm and expand upon previous observations of rootstock effects on scion phenotypes. Notably, this study was carried out using a robust experimental design (288~~-~~-vine set and 72~~-~~-vine set comprising replicates of three rootstocks grafted with a common scion and an ungrafted control~~),~~) in a vineyard that had been in the ground for eight years at the time of sampling. Our coordinated collection of five multi-dimensional leaf phenotypes, and inclusion of three sampling points spanning the growing season allowed us to investigate ~~in~~ the comprehensive nature of rootstock influences on the scion. Further, this thorough analysis demonstrates that rootstock effects on scion phenotypes shift in

Formatted: Normal

magnitude over the course of the season, indicating that aspects of time are tremendously influential to the observed results regardless of phenotype.

While the results of previous studies on grafted grapevine are worthy of comparison, the work presented here has a few limitations that render comparisons with other studies challenging for a variety of reasons. One novelty in our study is the exploration of a hybrid grapevine system, ‘Chambourcin’. ‘Chambourcin’ has a complex pedigree, including contributions from *Vitis riparia* and *V. rupestris*, species which are each parent to two of the rootstocks used in this study ~~[65].~~[64]. Many of the significant effects we observed in this study were subtle, which could reflect the genomic similarity between shoot and root systems. It might be expected that rootstocks derived from *V. riparia, V. rupestris* and other North American species might prompt more pronounced responses in European scions that lack North American *Vitis* in their pedigrees. Moreover, our results were derived from data collected in a single year at a single location. The phenotypes we measured are known to be heavily influenced by the environment, and we expect some inter-annual variation in rootstock influences on shoot system phenotypes. This study focused on a single scion, and as a result we are unable to explore how rootstock effects on shoot system phenotypes vary across scions. To our knowledge, this is among the largest populations to have been surveyed for such phenotypes in a near-decade~~-~~-old established vineyard. While many studies have been conducted in green houses or recently planted vineyards, the juxtaposition of our results and those previously established serve as a powerful foundation for the generation of hypotheses for future studies.

Phenomic covariation warrants work toward latent phenotypes

In the present study, we assess the extent of covariation among leaf phenotypes. For the primary dimensions of variation in each modality, within-modality correlations ~~are~~were strongest when accounting for phenological timing. Correlations also ~~exist~~existed between modalities, suggesting room for the analysis of latent phenomic structure or targeted integrative analyses for experimental questions. For example, aspects of the metabolome were frequently correlated with the transcriptome and leaf shape when accounting for both phenological stage and rootstock genotype. Interestingly, correlations within

Formatted: Normal

and between modalities were highly dynamic over a growing season and across rootstock genotype. For example, several correlations with leaf shape were present at veraison, but were not detected at anthesis and harvest. Moreover, the topology of connections in the ionomic network was variable over the rootstock genotype (Supplemental Figure 6). This variation in topology confirms that root system genotype has a strong influence on shoot system elemental composition, and suggests that root system genotype can alter correlative patterns in the ionome. We believe phenomic covariation warrants further investigation, specifically, by further including additional phenotypes such as lncRNA expression ~~[66,67], epigenetics [68], and microbiomes [69,70]~~[65,66], epigenetics [67], and microbiomes [68,69] which could yield more mechanistic understandings of the influence of root systems on shoot systems and how plants interact with their environments through their root systems. These mechanistic understandings could be used to further understand and optimize consumer-facing traits such as fruit quality and yield. To date, much of the work constituting phenomics in grapevine has addressed how berries develop over the growing season, how cultivars differ from one another, and how the concept of terroir influences wine ~~[51,52,55,71–73].~~[51,52,55,70–72]. Despite data integration techniques becoming more popular, there are still many open questions as to what analytical methods are most appropriate and how to most effectively utilize them (reviewed for grapevine in ~~[74,75]~~[73,74]; reviewed broadly in ~~[76,77]~~[75,76]). Ongoing work attempts to integrate high-dimensional phenomic datasets generated within a single organ system (e.g., leaves); and future studies will expand this to explore phenomic covariation in and among organs, over time, and across space.

Potential Implications

Our work on the influence of root system genotype on shoot system phenotype has broad implications for a holistic understanding of how plants detect and respond to changing environmental conditions, and how this response is coordinated among different organ systems. Data presented here demonstrate that root systems that are genetically distinct from the scion exert influence on the scion, leading to statistically significant differences in scion phenotypes based on the identity of their root

Formatted: Normal

systems. This observation suggests that the above-ground phenotype ~~of plants~~ results, at least in part, from below-ground activity of the root system. Further, these data highlight the value of coordinated collection of different multi-dimensional phenotypes for comparative studies, and for describing whole-plant phenotypic shifts over seasons and in response to horticultural manipulations.

Beyond its use as an experimental model that is ideal for studying root/shoot interaction, grafting is an important horticultural technique that is used in over 70 major crops. In grapevines, grafting was developed primarily to combat the below-ground pest phylloxera, and grapevine rootstocks were selected initially based on their resistance to this pest. Results presented here indicate that beyond phylloxera resistance, grafting to genetically distinct rootstocks is a potential source of variation for the scion. Ongoing work explores how root system impacts on shoot system phenotypes vary across scion genotypes, and how the rootstock × scion interaction changes over space. The long-term implications of this study are the potential honing of viticulture for future climates including the optimization of rootstock-scion combinations based in part on an understanding of how rootstock effects on scion phenotypes change over the course of the season. This work is relevant for breeding efforts, and may play a role in the optimization of quantitative phenotypes such as vigor, fruit quality, and yield that may be enhanced by, constrained by, or partially predicted from phenotypic variation elsewhere in the plant.

Methods

Study Design

Data were collected in 2017 from a split-plot experimental rootstock trial established in 2009 at the University of Missouri’s Southwest Research Center near Mount Vernon, MO (37.074167 N; 93.879167 W; Supplemental Figure 1). The rootstock trial includes the interspecific hybrid cultivar ‘Chambourcin’ growing ungrafted (own-rooted) and grafted to three rootstocks: ‘1103P’, ‘3309C’, and ‘SO4’ (Supplemental Figure 1D). Clonal replicates of each of the four rootstock-scion combinations were planted 72 times for a total of 288 vines planted in nine rows. Each row was treated with one of three

Formatted: Normal

irrigation treatments: full evapotranspiration replacement, partial (50%) evapotranspiration replacement (reduced deficit irrigation; RDI), or no evapotranspiration replacement (Supplemental Figure 1A). However, rainfall in 2017 likely mitigated the applied irrigation treatment (see Supplemental Note 1). Vine position in the vineyard corresponded to time of sampling for some phenotypes (metabolomics, gene expression, and physiology), as samples were taken from one end of the vineyard to the other over the course of two to three hours. Because vineyard microclimates and sampling time may be associated with phenomic variation, we defined ‘block’ as a factor that captures this spatial and temporal variation inherent in sampling for those phenotypes. In the other phenotypes (ionomics and leaf shape), neither row nor block correlated with time, so ‘block’ was simply a spatial covariate. Unique rootstock-scion combinations were planted in cells of four adjacent replicated vines (Supplemental Figure 1A-B), with rows consisting of eight cells (32 vines/row). To our knowledge, a field-planted rootstock experimental vineyard of this size and age is rare. For some phenotypes (ionomics and leaf shape), it was possible to collect samples from all vines in the experimental vineyard (the 288-vine set; Supplemental Figure 1A-B). For other phenotypes (metabolomics, gene expression, and physiology), time and/or expense associated with the phenotyping process required that we reduce sampling to a nested set of 72 vines representing the middle two vines in each four-vine cell in the front half of the vineyard (the 72-vine set; Supplemental Figure 1B-C). All phenotypes were assayed at three phenological stages: anthesis (~80% of open flowers; 22 May 2017); veraison (~50% of berries had transitioned from green to red; 30 July 2017); and immediately prior to harvest (25 September 2017). At each phenological stage, effort was made to sample on days with full to partial sun and minimal precipitation.

This design was used to assess the following questions: 1) What is the influence of root system genotype on shoot system phenotype? 2) How do systems of plant phenotypes vary over the growing season and does rootstock genotype influence this variation? And 3) how do phenotypes covary within and between phenotyping modalities?

Formatted: Normal

Linear Models

Linear models were fit to the 20 measured ion concentrations, the top 20 PCs of the leaf metabolome, the top 100 PCs of the leaf transcriptome, the top 20 PCs of leaf morphospace, and each measured physiological trait. Outliers were detected using the R function 'anomalize' (options: alpha=0.03, max_anoms=0.1). Each model was fit with fixed effect factors representing phenological stage (anthesis, veraison, or harvest), rootstock (Ungrafted, '1103P', '3309C', or 'SO4'), leaf position (youngest, middle, or oldest; only used in leaf morphology and leaf ion concentration models), and all pairwise interactions of those terms. Both irrigation and block were included as fixed, non-interacting effects with the exceptions of physiology and metabolomics, for which we allowed the interaction of 'Block' as it correlates with the time of sampling, potentially capturing temporal variation. Row, an additional correlate for time and spatial variation, was included in place of a temporal block for the gene expression models after removal of the variation attributable to irrigation, a factor collinear with row. All linear models were interpreted using a type-3 sum of squares computation using the R package 'car' ~~[78].~~[77]. Estimated p-values for each term in the models were corrected for multiple tests (within phenotype) using FDR correction as implemented by the R package 'stats' ~~[79].~~[78]. Results from the models are reported as the variation explained by a particular term in the model and the estimated p-value. When appropriate, post-hoc mean comparisons were computed using the package 'emmeans' ~~[80].~~[79]. Where multiple linear models were being simultaneously interpreted, we applied a Bonferonni correction to reduce the number of false positives.

Machine Learning to Identify Rootstock Effects

For visualization of between-class variation, we fit linear discriminant analysis models (LDA) to each modality (ionomics, metabolomics, gene expression, and leaf morphology) using the 'lda' function of the R package 'MASS' ~~[81].~~[80]. Projections of all samples into the LD space were plotted using ggplot2 ~~[82].~~[81]. In addition, we employed machine learning to capture subtle experimental effects. We partitioned data from each modality into 80% training partitions and 20% testing partitions. Models were

Formatted: Normal

fit to predict the phenological stage from which a sample was taken, the rootstock to which the scion was grafted, and the joint prediction of phenology and rootstock. We also tested the predictability of leaf position for ionomics and leaf shape, and the interaction of rootstock and leaf position for ionomics. We used the 'randomForest' ~~[83]~~[82] implementation of the random forest algorithm. Models were fit and tuned using the R package 'caret' ~~[84].~~[83]. Each performance was assessed using accuracy, with performance on each class being assessed using the balanced accuracy, the midpoint of class-wise sensitivity and specificity. Where appropriate, models were compared to 'chance', or the occurrence frequency of each class. Confusion matrices were visualized from the out-of-bag predictions using ~~ggplot2~~'ggplot2'. Important features were identified from the randomForest object based on a phenotype-specific mean decrease in model accuracy (MDA).

Phenomic trait covariation

We extracted ionomics, metabolomics, gene expression, and leaf shape data for the youngest available leaf from the 72~~-~~-vine~~-~~ set. Each data modality was summarized along the primary dimensions of variation using PCA. For each class, we extracted the top 10 PCs and fit Pearson's correlations across all pairs of PCs at each phenological stage. P-values from computed correlations were corrected using the FDR method from the package 'stats' ~~[85].~~[84]. Correlations and their strengths were visualized using the R package 'igraph' ~~[86].~~[85]. Example correlations were reported after running 10,000 bootstrapped subsamples of 90% of data for paired phenotypes. From the distribution of estimated correlation coefficients, confidence intervals were computed from the 0.025 and 0.975 quantiles. A subset of example correlations were plotted using the R package 'ggplot2' ~~[82].~~.

Acknowledgments:

This work was funded by the National Science Foundation Plant Genome Research Project 1546869. We thank members of the Miller Lab at Saint Louis University and the Donald Danforth Plant Science Center, members of the Kovacs Lab at Missouri State University, members of the Kwasniewski Lab at the

Formatted: Normal

University of Missouri, and members Londo Lab at the USDA-ARS Grape Research Unit for vineyard sampling and sample processing. We express special thanks to Matthew Rubin and Elizabeth Kellogg at the Donald Danforth Plant Science Center for valuable comments on the manuscript. Finally, we thank the reviewers of this manuscript whose comments have led to clearer and more complete manuscript.

Figure Legends:

Figure 1: The ionome shows strong signal from rootstock genotype, leaf position, and phenological stage **(A)** Percent variation captured in linear models fit to each of 20 ions measured in the ionomics pipeline. Presence of a cell indicates the model term (top) was significant (FDR; p.adj < 0.05) for that ion (left). **(B)** Example ions shown to vary significantly by the interaction of leaf position (Y=Youngest, M=Middle, O=Oldest) and phenological stage in parts per million. Boxes are bound by 25th and 75th percentile with whiskers extending 1.5 IQR from the box. Significant changes are indicated by letters above boxes, and are only meant for comparison within each phenological stage. Group means are displayed with black squares. (**C**) Example ions shown to vary significantly by the interaction of rootstock genotype and phenological stage in parts per million. Significant changes are indicated by letters above boxes, and are only meant for comparison within each phenological stage. Boxes are bound by 25th and 75th percentile with whiskers extending 1.5 IQR from the box. Group means are displayed with black squares. (**D**) Standardized heatmap for out-of-bag (OOB) predictions by a random forest trained to predict rootstock genotype, **(E)** the interaction between rootstock genotype by phenology, and **(F)** the interaction between rootstock genotype and leaf position.

Figure 2: The metabolome is influenced by rootstock genotype, phenological stage, and time of sampling. **(A)** Percent variation captured in linear models fit to each of the top 20 principal components of the metabolome (661 measured metabolites). Presence of a cell indicates the model term (top) was significant for that PC (left, percent variation explained by the PC in parentheses). **(B)** The distribution of projections onto PC17, the strongest captured rootstock effect in the metabolome. Boxes are bound by the 25th and

Formatted: Normal

75th percentiles with whiskers extending 1.5 IQR from the box. **(C)** Projections of all samples into the first two dimensions of a linear discriminant space trained to maximize variation between rootstock genotypes.

Figure 3: Gene expression primarily responds to time of season and circadian correlates

(A) Heatmap showing 500 genes with the highest variance following the filtering of lowly expressed genes and gene-by-gene variance stabilizing transformations (VST) ordered by example model factors (below). **(B)** Percent variation captured in linear models fit to the top 100 Principal Components of the VST-transformed gene-expression space. Presence of a cell indicates the model term (top) was significant for that PC (left, percent variation explained by the PC in parentheses). **(C)** Projections of all samples into the first two principal component dimensions to show that the largest descriptors of variation are due to phenology. **(D)** Projections of all samples into the first two dimensions of the linear discriminant space trained to maximize variation between the rows of the vineyard, and **(E)** rootstock genotype.

Figure 4: Leaf shape variation is primarily determined by shoot position but changes over the season

(A) Representative shapes showing leaf variation (-3 sd, mean, +3 sd) captured in each of the top 4 principal components of the Generalized Procrustes Analysis-rotated leaf shapes. **(B)** Projections of all leaves into the first two dimensions of principal component space colored by the strongest determinant of variation in the top two PCs. **(C)** Projections of all leaves into the first two dimensions of a linear discriminant space trained to maximize variation between phenological stages. **(D)** Variation in leaf shape captured on PC2 shown by leaf position and phenological stage. Large points represent the mean of the group when projected onto PC2. Bars surrounding the mean show one standard deviation. Variation in each group is shown as a composite leaf trace scaled to a standard size and centered over the mean.

Figure 5: Vine physiology varies with rootstock and the rootstock by phenology interaction

Formatted: Normal

(A) Percent variation explained by model terms (top) from linear models fit to each of four physiology traits (left). **(B)** Variation in leaf transpiration rate for each rootstock genotype over the course of the season. Boxes are bound by the 25th and 75th percentiles with whiskers extending 1.5 IQR from the box. Significant changes are indicated by letters above boxes, and are only meant for comparison within each phenological stage. Group means are displayed with black squares. **(C)** Variation in stomatal conductance for each rootstock genotype over the course of the season. Boxes are bound by the 25th and 75th percentiles with whiskers extending 1.5 IQR from the box. Group means are displayed with black squares. Significant changes are indicated by letters above boxes, and are only meant for comparison within each phenological stage.

Figure 6: Phenomic covariation varies over the course of the season

Correlation networks showing patterns of covariation within and between phenotyping modalities. Nodes of the network are connected if they are significantly correlated (Pearson, FDR; $p.adj < 0.05$). Edge thickness is proportional to the strength of correlation (multiplied by 16 for visibility). Edge color reflects the direction of the correlation where blue edges indicate positive correlations and orange edges indicate negative correlations. Modalities are indicated by a leading character and node color: ionomics (iPCs; purple), metabolomics (mPCs; pink), gene expression (gPCs; yellow), leaf shape (sPCs; green). Network topologies are shown for **(A)** anthesis, **(B)** veraison, and **(C)** harvest.

Figure Supplement Legends:

Supplemental Figure 1: Experimental Design

(A) Vineyard Map. The vineyard features a randomized block design where ‘Chambourcin’ is grown ungrafted and grafted to three rootstock genotypes: ‘1103P’, ‘3309C’, and ‘SO4’. Each row is treated with one of three irrigation treatments: full replacement of ET, reduced-deficit, no replacement of ET. Each cell of the vineyard contains four replicate grafts. **(B)** Phenotype sampling scheme across the four replicates in a cell. For example, the top panel (purple) shows all four vines in the first cell of Row 8 in

Formatted: Normal

Block D. From each vine in that cell, ionomics and leaf shape were sampled. In contrast, the lower panel shows the first cell in Row 8 in Block A. Here, the first and fourth replicates were sampled for ionomics and leaf shape while the second and third replicates were sampled for all phenotypes. All vines (288) were sampled for ionomics and leaf shape. The middle two vines in the front half of the vineyard (72) were additionally sampled for metabolomics, gene expression, and physiology. **(C)** Phenotype sample scheme within a vine (along a shoot). For each plant, young leaves were sampled for ionomics, leaf shape, and gene expression. Middle leaves were sampled for ionomics, leaf shape, metabolomics, and physiology. Older leaves were sampled for ionomics and leaf shape. Samples for ionomics and leaf shape were taken from the same shoot. All other phenotypes were sampled from independent shoots. **(D)** Rootstock relatedness. Each of the rootstocks in this trial shares a parent species with a different rootstock. ‘1103P’ is a cross between *Vitis rupestris* and *V. berlandieri*. ‘3309C’ is a cross between *V. rupestris* and *V. riparia*. ‘SO4’ is a cross between *V. riparia* and *V. berlandieri*. The parent that is shared between each pair of rootstocks is highlighted. This figure is partially reproduced from [19][19] available under a Creative Common license (CC BY 4.0).

Supplemental Figure 2: Quality and validity assessment of 3’ RNAseq data. **(A)** A survey of recently annotated circadian clock orthologs from the grapevine genome annotation [44]. Orthologs surveyed included the morning-phased RVE1 and LHY, evening-phased LUX and ELF4, and the nigh-phased TOC1 **(B)** A survey of genes with housekeeping domains related to IPR000626 (ubiquitin) and IPR004000 (actin).

Supplemental Figure 3: Patterns of ion covariation change over experimental treatments

Correlation networks showing patterns of ion covariation across phenological stages and shoot position. Nodes of the network are connected if they are significantly correlated (Pearson, FDR; p.adj < 0.05). Edge thickness is proportional to the strength of correlation (multiplied by 16 for visibility). Edge color

Formatted: Normal

reflects the direction of the correlation where blue edges indicate positive correlations and orange edges indicate negative correlations.

Supplemental Figure 4: Patterns of variation contributing to gene expression linear discriminants

(**A**) Projections of leaf gene expression samples into the first two dimensions of a linear discriminant space trained to maximize variation between phenological stages, rows in the vineyard, and rootstock genotype. For each LD, the PCs that loaded significantly (>1.96 sd from the mean loading) are listed in order of loading magnitude. (**B**) Distribution of the top loading PCs onto LD1 and LD2 for each of the trained models.

Supplemental Figure 5: Patterns of variation in leaf shape are subtle

(**A**) Percent variation captured in linear models fit to each of the top 20 principal components of leaf morphology. Presence of a cell indicates the model term (top) was significant for that PC (left, percent variation explained by the PC in parentheses). (**B**) Composite leaf traces for the main rootstock genotype effect identified on PC1.

Supplemental Figure 6: Example correlations within and between phenotyping modalities over the course of the season

(**A**) Example correlation showing a strong within-modality correlation between the ionomics gPC1 and gPC2 at anthesis. Pearson correlations by phenological stage and CIs derived from 10000 random 90% draws are shown for each panel. Generally speaking, CIs overlapping with 0 were not accepted as significant. (**B**) Example correlation showing one of the stronger between-modality correlations between the gene expression gPC4 and morphology (shape) sPC3 at veraison. (**C**) Example correlation of a relationship that is present multiple times over the course of the season between metabolomics mPC3 and

Formatted: Normal

gene expression gPC6 at both veraison and harvest. (**D**) Example correlation that is dynamic over the course of the growing season between the ionomics mPC3 and mPC6.

Supplemental Figure 7: Phenomic covariation varies over rootstock genotype

Correlation networks showing patterns of covariation within and between phenotyping modalities. Nodes of the network are connected if they are significantly correlated (Pearson, FDR; p.adj < 0.05). Edge thickness is proportional to the strength of correlation (multiplied by 16 for visibility). Edge color reflects the direction of the correlation where blue edges indicate positive correlations and orange edges indicate negative correlations. Modalities are indicated by a leading character and node color: ionomics (iPCs; purple), metabolomics (mPCs; pink), gene expression (gPCs; yellow), leaf shape (sPCs; green). Network topologies are shown for **(A)** Ungrafted, **(B)** ‘1103P’-grafted vines, **(C)** ‘3309C’-grafted vines, and **(D)** ‘SO4’-grafted vines.

Availability of Data:

Ionomics data are available at https://dx.doi.org/10.6084/m9.figshare.13200980 . Metabolomics data are available at https://dx.doi.org/10.6084/m9.figshare.13201043. Gene expression data are available in the Sequence Read Archive under BioProject PRJNA674915. Leaf scans and leaf landmarks are available at https://dx.doi.org/10.6084/m9.figshare.13200953. Weather and physiology data are available at https://dx.doi.org/10.6084/m9.figshare.13198682 and https://dx.doi.org/10.6084/m9.figshare.13201016, respectively.

Availability of Code:

All code for this paper including shell scripts for RNAseq analysis and Jupyter Notebooks for data analysis in R can be found on the Vitis Underground GitHub (https://github.com/PGRP1546869/mt_vernon_2017_leaf).

Formatted: Normal

Author Contributions:

AJM, DHC, AF, LGK, MK, JPL, and QM designed the experiment. ZNH, LLK, MA, JFS, ZM, NB, EF, and JPL contributed to sample collection and sample processing. ZNH, LLK, JFS, and MA contributed to data analysis. ZNH and AJM contributed to the writing of the manuscript. All authors contributed to manuscript editing.

References:

~~1. Gehan MA, Fahlgren N, Abbasi A, Berry JC, Callen ST, Chavez L, et al.. PlantCV v2: Image analysis software for high-throughput plant phenotyping. *PeerJ*. 5:e40882017;~~

~~2. Ubbens JR, Stavness I. Deep Plant Phenomics: A Deep Learning Platform for Complex Plant Phenotyping Tasks. *Front Plant Sci*. 8:11902017;~~

~~3. Ubbens J, Cieslak M, Prusinkiewicz P, Stavness I. Latent Space Phenotyping: Automatic Image-Based Phenotyping for Treatment Studies.~~

~~4. Soulé M. PHENETICS OF NATURAL POPULATIONS I. PHENETIC RELATIONSHIPS OF INSULAR POPULATIONS OF THE SIDE-BLOTCHED LIZARD. *Evolution*. 21:584–911967;~~

~~5. Houle D, Govindaraju DR, Omholt S. Phenomics: the next challenge. *Nat Rev Genet*. 11:855–662010;~~

~~6. Mudge K, Janick J, Scofield S, Goldschmidt EE. A History of Grafting. In: Janick J, editor. *Horticultural Reviews*. Hoboken, NJ, USA: John Wiley & Sons, Inc.; p. 437–93.~~

~~7. Pouget R. Histoire de la lutte contre le phylloxéra de la vigne en France: 1868-1895. *Hist Sci Med*. INRA; 1990;~~

~~8. Walker MA, Lund K, Agüero C, Riaz S, Fort K, Heinitz C, et al.. BREEDING GRAPE ROOTSTOCKS FOR RESISTANCE TO PHYLLOXERA AND NEMATODES – IT'S NOT ALWAYS EASY. Acta Horticulturae.~~

~~9. Warschefsky EJ, Klein LL, Frank MH, Chitwood DH, Londo JP, von Wettberg EJB, et al.. Rootstocks: Diversity, Domestication, and Impacts on Shoot Phenotypes. *Trends Plant Sci*. Elsevier Current Trends; 21:418–372016;~~

~~10. Tramontini S, Vitali M, Centioni L, Schubert A, Lovisolo C. Rootstock control of scion response to water stress in grapevine. Environmental and Experimental Botany.~~

~~11. Bavaresco L, Lovisolo C. Effect of grafting on grapevine chlorosis and hydraulic conductivity. *VITIS-Journal of Grapevine Research*. Citeseer; 2015;~~

~~12. Ferlito F, Distefano G, Gentile A, Allegra M, Lakso AN, Nicolosi E. Scion–rootstock interactions influence the growth and behaviour of the grapevine root system in a heavy clay soil. Australian Journal of Grape and Wine Research.~~

Formatted: Normal

13. Ordish G. The great wine blight. J.M. Dent & Sons;

14. Cookson SJ, Ollat N. Grafting with rootstocks induces extensive transcriptional re-programming in the shoot apical meristem of grapevine. *BMC Plant Biol*. BioMed Central; 13:1–142013;

15. Corso M, Vannozzi A, Ziliotto F, Zouine M, Maza E, Nicolato T, et al.. Grapevine Rootstocks Differentially Affect the Rate of Ripening and Modulate Auxin-Related Genes in Cabernet Sauvignon Berries. *Front Plant Sci*. Frontiers; 2016; doi: 1. Gehan MA, Fahlgren N, Abbasi A, Berry JC, Callen ST, Chavez L, et al.. PlantCV v2: Image analysis software for high-throughput plant phenotyping. *PeerJ*. 5:e40882017;

2. Ubbens JR, Stavness I. Deep Plant Phenomics: A Deep Learning Platform for Complex Plant Phenotyping Tasks. *Front Plant Sci*. 8:11902017;

3. Ubbens J, Cieslak M, Prusinkiewicz P, Stavness I. Latent Space Phenotyping: Automatic Image-Based Phenotyping for Treatment Studies.

4. Soulé M. PHENETICS OF NATURAL POPULATIONS I. PHENETIC RELATIONSHIPS OF INSULAR POPULATIONS OF THE SIDE-BLOTCHED LIZARD. *Evolution*. 21:584–911967;

5. Houle D, Govindaraju DR, Omholt S. Phenomics: the next challenge. *Nat Rev Genet*. 11:855–662010;

6. Mudge K, Janick J, Scofield S, Goldschmidt EE. A History of Grafting. In: Janick J, editor. *Horticultural Reviews*. Hoboken, NJ, USA: John Wiley & Sons, Inc.; p. 437–93.

7. Pouget R. Histoire de la lutte contre le phylloxéra de la vigne en France: 1868-1895. *Hist Sci Med*. INRA; 1990;

8. Walker MA, Lund K, Agüero C, Riaz S, Fort K, Heinitz C, et al.. BREEDING GRAPE ROOTSTOCKS FOR RESISTANCE TO PHYLLOXERA AND NEMATODES - IT'S NOT ALWAYS EASY. Acta Horticulturae.

9. Warschefsky EJ, Klein LL, Frank MH, Chitwood DH, Londo JP, von Wettberg EJB, et al.. Rootstocks: Diversity, Domestication, and Impacts on Shoot Phenotypes. *Trends Plant Sci*. Elsevier Current Trends; 21:418–372016;

10. Tramontini S, Vitali M, Centioni L, Schubert A, Lovisolo C. Rootstock control of scion response to water stress in grapevine. Environmental and Experimental Botany.

11. Bavaresco L, Lovisolo C. Effect of grafting on grapevine chlorosis and hydraulic conductivity. *VITIS-Journal of Grapevine Research*. Citeseer; 2015;

12. Ferlito F, Distefano G, Gentile A, Allegra M, Lakso AN, Nicolosi E. Scion–rootstock interactions influence the growth and behaviour of the grapevine root system in a heavy clay soil. Australian Journal of Grape and Wine Research.

13. Ordish G. The great wine blight. J.M. Dent & Sons;

14. Cookson SJ, Ollat N. Grafting with rootstocks induces extensive transcriptional re-programming in the shoot apical meristem of grapevine. *BMC Plant Biol*. BioMed Central; 13:1–142013;

15. Corso M, Vannozzi A, Ziliotto F, Zouine M, Maza E, Nicolato T, et al.. Grapevine Rootstocks Differentially Affect the Rate of Ripening and Modulate Auxin-Related Genes in Cabernet Sauvignon

Formatted: Normal

Berries. *Front Plant Sci*. Frontiers; 2016; doi: 10.3389/fpls.2016.00069.

16. Berdeja M, Nicolas P, Kappel C, Dai ZW, Hilbert G, Peccoux A, et al.. Water limitation and rootstock genotype interact to alter grape berry metabolism through transcriptome reprogramming. *Hortic Res*. 2:150122015;

17. Zombardo A, Crosatti C, Bagnaresi P, Bassolino L, Reshef N, Puccioni S, et al.. Transcriptomic and biochemical investigations support the role of rootstock-scion interaction in grapevine berry quality. *BMC Genomics*. 21:4682020;

18. Chitarra W, Perrone I, Avanzato CG, Minio A, Boccacci P, Santini D, et al.. Grapevine Grafting: Scion Transcript Profiling and Defense-Related Metabolites Induced by Rootstocks. *Front Plant Sci*. Frontiers; 2017; doi: 16. Berdeja M, Nicolas P, Kappel C, Dai ZW, Hilbert G, Peccoux A, et al.. Water limitation and rootstock genotype interact to alter grape berry metabolism through transcriptome reprogramming. *Hortic Res*. 2:150122015;

17. Zombardo A, Crosatti C, Bagnaresi P, Bassolino L, Reshef N, Puccioni S, et al.. Transcriptomic and biochemical investigations support the role of rootstock-scion interaction in grapevine berry quality. *BMC Genomics*. 21:4682020;

18. Chitarra W, Perrone I, Avanzato CG, Minio A, Boccacci P, Santini D, et al.. Grapevine Grafting: Scion Transcript Profiling and Defense-Related Metabolites Induced by Rootstocks. *Front Plant Sci*. Frontiers; 2017; doi: 10.3389/fpls.2017.00654.

19. Migicovsky Z, Harris ZN, Klein LL, Li M, McDermaid A, Chitwood DH, et al.. Rootstock effects on scion phenotypes in a "Chambourcin" experimental vineyard. *Horticulture Research*. Nature Publishing Group; 6:1–132019;

20. Galet P. A Practical Ampelography: Grapevine Identification. Comstock Pub. Associates;

21. Mullins MG, Bouquet A, Williams LE. Biology of the Grapevine. Cambridge University Press;

22. Chitwood DH, Ranjan A, Martinez CC, Headland LR, Thiem T, Kumar R, et al.. A modern ampelography: a genetic basis for leaf shape and venation patterning in grape. *Plant Physiol*. 164:259–722014;

23. Chitwood DH, Klein LL, O'Hanlon R, Chacko S, Greg M, Kitchen C, et al.. Latent developmental and evolutionary shapes embedded within the grapevine leaf. New Phytologist.

24. Klein LL, Caito M, Chapnick C, Kitchen C, O'Hanlon R, Chitwood DH, et al.. Digital Morphometrics of Two North American Grapevines (Vitis: Vitaceae) Quantifies Leaf Variation between Species, within Species, and among Individuals. *Front Plant Sci*. 8:3732017;

25. Grimes DW, Williams LE. Irrigation Effects on Plant Water Relations and Productivity of Thompson Seedless Grapevines. *Crop Sci*. 30:2551990;

26. Williams LE, Grimes DW. Modelling vine growth-development of a data set for a water balance subroutine. *Proceedings of the Sixth Australian Wine Industry Technical Conference*. p. 169–74.

27. Gautier A, Cookson SJ, Lagalle L, Ollat N, Marguerit E. Influence of the three main genetic backgrounds of grapevine rootstocks on petiolar nutrient concentrations of the scion, with a focus on phosphorus. *OENO One*. 54:1–132020;

Formatted: Normal

28. Lecourt J, Lauvergeat V, Ollat N, Vivin P, Cookson SJ. Shoot and root ionome responses to nitrate supply in grafted grapevines are rootstock genotype dependent: Rootstock and nitrogen supply affect grapevine ionome. *Aust J Grape Wine Res*. 21:311–82015;

29. Salt DE, Baxter I, Lahner B. Ionomics and the study of the plant ionome. *Annu Rev Plant Biol*. 59:709–332008;

30. Baxter I. Ionomics: The functional genomics of elements. *Brief Funct Genomics*. 9:149–562010;

31. Ziegler G, Terauchi A, Becker A, Armstrong P, Hudson K, Baxter I. Ionomic Screening of Field-Grown Soybean Identifies Mutants with Altered Seed Elemental Composition. The Plant Genome.

32. Oliver SG, Winson MK, Kell DB, Baganz F. Systematic functional analysis of the yeast genome. *Trends Biotechnol*. 16:373–81998;

33. Tweeddale H, Notley-McRobb L, Ferenci T. Effect of slow growth on metabolism of Escherichia coli, as revealed by global metabolite pool (“metabolome”) analysis. *J Bacteriol*. 180:5109–161998;

34. Islam MN, Downey F, Ng CKY. Comparative analysis of bioactive phytochemicals from Scutellaria baicalensis, Scutellaria lateriflora, Scutellaria racemosa, Scutellaria tomentosa and Scutellaria wrightii by LC-DAD-MS. *Metabolomics*. Springer; 7:446–532011;

35. Tautenhahn R, Patti GJ, Rinehart D, Siuzdak G. XCMS Online: a web-based platform to process untargeted metabolomic data. *Anal Chem*. 84:5035–92012;

36. Tandonnet S, Torres TT. Traditional versus 3’ RNA-seq in a non-model species. *Genom Data*. 11:9–162017;

37. Bolger AM, Lohse M, Usadel B. Trimmomatic: a flexible trimmer for Illumina sequence data. *Bioinformatics*. 30:2114–202014;

38. Bushnell B. BBTools software package. *URL http://sourceforge net/projects/bbmap*. 2017;

39. Jaillon O, Aury J-M, Noel B, Policriti A, Clepet C, Casagrande A, et al.. The grapevine genome sequence suggests ancestral hexaploidization in major angiosperm phyla. *Nature*. 449:463–72007;

40. Canaguier A, Grimplet J, Di Gaspero G, Scalabrin S, Duchêne E, Choisne N, et al.. A new version of the grapevine reference genome assembly (12X.v2) and of its annotation (VCost.v3). *Genom Data*. 14:56–622017;

41. Dobin A, Davis CA, Schlesinger F, Drenkow J, Zaleski C, Jha S, et al.. STAR: ultrafast universal RNA-seq aligner. *Bioinformatics*. 29:15–212013;

42. Anders S, Pyl PT, Huber W. HTSeq: Analysing high-throughput sequencing data with Python.

43. Love MI, Huber W, Anders S. Moderated estimation of fold change and dispersion for RNA-seq data with DESeq2. *Genome Biol*. 15:5502014;

44. Carbonell-Bejerano P, Rodríguez V, Royo C, Hernáiz S, Moro-González LC, Torres-Viñals M, et al.. Circadian oscillatory transcriptional programs in grapevine ripening fruits. *BMC Plant Biol*. 14:782014;

45. Anders S, Huber W. Differential expression analysis for sequence count data. *Genome Biol*. 11:R1062010;

Formatted: Normal

46. Dryden IL, Mardia KV. Statistical Shape Analysis: With Applications in R. John Wiley & Sons;

47. Shackel K, Gross R. Using midday stem water potential to assess irrigation needs of landscape valley oaks. 2002;

48. Levin AD. Re-evaluating pressure chamber methods of water status determination in field-grown grapevine (Vitis spp.). *Agric Water Manag*. Elsevier BV; 221:422–92019;

49. Liang W, Zou X, Carballar-Lejarazú R, Wu L, Sun W, Yuan X, et al.. Selection and evaluation of reference genes for qRT-PCR analysis in Euscaphis konishii Hayata based on transcriptome data. *Plant Methods*. Springer Science and Business Media LLC; 2018; doi: 19. Migicovsky Z, Harris ZN, Klein LL, Li M, McDermaid A, Chitwood DH, et al.. Rootstock effects on scion phenotypes in a “Chambourcin” experimental vineyard. *Hortic Res*. 6:642019;

20. Galet P. A Practical Ampelography: Grapevine Identification. Comstock Pub. Associates;

21. Mullins MG, Bouquet A, Williams LE. Biology of the Grapevine. Cambridge University Press;

22. Chitwood DH, Ranjan A, Martinez CC, Headland LR, Thiem T, Kumar R, et al.. A modern ampelography: a genetic basis for leaf shape and venation patterning in grape. *Plant Physiol*. 164:259–722014;

23. Chitwood DH, Klein LL, O’Hanlon R, Chacko S, Greg M, Kitchen C, et al.. Latent developmental and evolutionary shapes embedded within the grapevine leaf. New Phytologist.

24. Klein LL, Caito M, Chapnick C, Kitchen C, O’Hanlon R, Chitwood DH, et al.. Digital Morphometrics of Two North American Grapevines (Vitis: Vitaceae) Quantifies Leaf Variation between Species, within Species, and among Individuals. *Front Plant Sci*. 8:3732017;

25. Grimes DW, Williams LE. Irrigation Effects on Plant Water Relations and Productivity of Thompson Seedless Grapevines. *Crop Sci*. 30:2551990;

26. Williams LE, Grimes DW. Modelling vine growth-development of a data set for a water balance subroutine. *Proceedings of the Sixth Australian Wine Industry Technical Conference*. p. 169–74.

27. Gautier A, Cookson SJ, Lagalle L, Ollat N, Marguerit E. Influence of the three main genetic backgrounds of grapevine rootstocks on petiolar nutrient concentrations of the scion, with a focus on phosphorus. *OENO One*. 54:1–132020;

28. Lecourt J, Lauvergeat V, Ollat N, Vivin P, Cookson SJ. Shoot and root ionome responses to nitrate supply in grafted grapevines are rootstock genotype dependent: Rootstock and nitrogen supply affect grapevine ionome. *Aust J Grape Wine Res*. 21:311–82015;

29. Salt DE, Baxter I, Lahner B. Ionomics and the study of the plant ionome. *Annu Rev Plant Biol*. 59:709–332008;

30. Baxter I. Ionomics: The functional genomics of elements. *Brief Funct Genomics*. 9:149–562010;

31. Ziegler G, Terauchi A, Becker A, Armstrong P, Hudson K, Baxter I. Ionomic Screening of Field-Grown Soybean Identifies Mutants with Altered Seed Elemental Composition. The Plant Genome.

32. Oliver SG, Winson MK, Kell DB, Baganz F. Systematic functional analysis of the yeast genome. *Trends Biotechnol*. 16:373–81998;

Formatted: Normal

33. Tweeddale H, Notley-McRobb L, Ferenci T. Effect of slow growth on metabolism of Escherichia coli, as revealed by global metabolite pool (“metabolome”) analysis. *J Bacteriol*. 180:5109–161998;

34. Islam MN, Downey F, Ng CKY. Comparative analysis of bioactive phytochemicals from Scutellaria baicalensis, Scutellaria lateriflora, Scutellaria racemosa, Scutellaria tomentosa and Scutellaria wrightii by LC-DAD-MS. *Metabolomics*. Springer; 7:446–532011;

35. Tautenhahn R, Patti GJ, Rinehart D, Siuzdak G. XCMS Online: a web-based platform to process untargeted metabolomic data. *Anal Chem*. 84:5035–92012;

36. Tandonnet S, Torres TT. Traditional versus 3’ RNA-seq in a non-model species. *Genom Data*. 11:9–162017;

37. Bolger AM, Lohse M, Usadel B. Trimmomatic: a flexible trimmer for Illumina sequence data. *Bioinformatics*. 30:2114–202014;

38. Bushnell B. BBTools software package. *URL http://sourceforge net/projects/bbmap*. 2017;

39. Jaillon O, Aury J-M, Noel B, Policriti A, Clepet C, Casagrande A, et al.. The grapevine genome sequence suggests ancestral hexaploidization in major angiosperm phyla. *Nature*. 449:463–72007;

40. Canaguier A, Grimplet J, Di Gaspero G, Scalabrin S, Duchêne E, Choisne N, et al.. A new version of the grapevine reference genome assembly (12X.v2) and of its annotation (VCost.v3). *Genom Data*. 14:56–622017;

41. Dobin A, Davis CA, Schlesinger F, Drenkow J, Zaleski C, Jha S, et al.. STAR: ultrafast universal RNA-seq aligner. *Bioinformatics*. 29:15–212013;

42. Anders S, Pyl PT, Huber W. HTSeq: Analysing high-throughput sequencing data with Python.

43. Love MI, Huber W, Anders S. Moderated estimation of fold change and dispersion for RNA-seq data with DESeq2. *Genome Biol*. 15:5502014;

44. Carbonell-Bejerano P, Rodríguez V, Royo C, Hernáiz S, Moro-González LC, Torres-Viñals M, et al.. Circadian oscillatory transcriptional programs in grapevine ripening fruits. *BMC Plant Biol*. 14:782014;

45. Anders S, Huber W. Differential expression analysis for sequence count data. *Genome Biol*. 11:R1062010;

46. Dryden IL, Mardia KV. Statistical Shape Analysis: With Applications in R. John Wiley & Sons;

47. Shackel K, Gross R. Using midday stem water potential to assess irrigation needs of landscape valley oaks. 2002;

48. Levin AD. Re-evaluating pressure chamber methods of water status determination in field-grown grapevine (Vitis spp.). *Agric Water Manag*. Elsevier BV; 221:422–92019;

49. Liang W, Zou X, Carballar-Lejarazú R, Wu L, Sun W, Yuan X, et al.. Selection and evaluation of reference genes for qRT-PCR analysis in Euscaphis konishii Hayata based on transcriptome data. *Plant Methods*. Springer Science and Business Media LLC; 2018; doi: 10.1186/s13007-018-0311-x.

~~50. Podani J, Miklós I. Resemblance coefficients and the horseshoe effect in principal coordinates analysis. *Ecology*. Wiley; 83:3331–432002;~~

Formatted: Normal

51. Degu A, Hochberg U, Sikron N, Venturini L, Buson G, Ghan R, et al.. Metabolite and transcript profiling of berry skin during fruit development elucidates differential regulation between Cabernet Sauvignon and Shiraz cultivars at branching points in the polyphenol pathway. *BMC Plant Biol*. 14:1882014;

52. Anesi A, Stocchero M, Dal Santo S, Commisso M, Zenoni S, Ceoldo S, et al.. Towards a scientific interpretation of the terroir concept: plasticity of the grape berry metabolome. *BMC Plant Biol*. 15:1912015;

53. Cuadros-Inostroza A, Ruíz-Lara S, González E, Eckardt A, Willmitzer L, Peña-Cortés H. GC-MS metabolic profiling of Cabernet Sauvignon and Merlot cultivars during grapevine berry development and network analysis reveals a stage- and cultivar-dependent connectivity of primary metabolites. *Metabolomics*. 12:392016;

54. Dal Santo S, Fasoli M, Negri S, D'Incà E, Vicenzi N, Guzzo F, et al.. Plasticity of the Berry Ripening Program in a White Grape Variety. *Front Plant Sci*. 7:9702016;

55. Zamboni A, Di Carli M, Guzzo F, Stocchero M, Zenoni S, Ferrarini A, et al.. Identification of putative stage-specific grapevine berry biomarkers and omics data integration into networks. *Plant Physiol*. 154:1439–592010;

56. Dal Santo S, Tornielli GB, Zenoni S, Fasoli M, Farina L, Anesi A, et al.. The plasticity of the grapevine berry transcriptome. *Genome Biol*. 14:r542013;

57. Chitwood DH, Rundell SM, Li DY, Woodford QL, Yu TT, Lopez JR, et al.. Climate and Developmental Plasticity: Interannual Variability in Grapevine Leaf Morphology. *Plant Physiol*. 170:1480–912016;

58. Chitwood DH, Mullins J, Migicovsky Z, Frank M, VanBuren R, Londo JP. Vein-to-blade ratio is an allometric indicator of climate-induced changes in grapevine leaf size and shape. bioRxiv.

59. Gautier A, Cookson SJ, Lagalle L, Ollat N, Marguerit E. Influence of the three main genetic backgrounds of grapevine rootstocks on petiolar nutrient concentrations of the scion, with a focus on phosphorus. *OENO One*. 54:1–132020;

60. Bravdo B. EFFECT OF MINERAL NUTRITION AND SALINITY ON GRAPE PRODUCTION AND WINE QUALITY. *Acta Horticulturae*. 512:23–302000;

61. Brunetto G, Melo GWBDE, Toselli M, Quartieri M, Tagliavini M. The role of mineral nutrition on yields and fruit quality in grapevine, pear and apple. *Rev Bras Frutic*. FapUNIFESP (SciELO); 37:1089–1042015;

62. Gautier A, Cookson SJ, Hevin C, Vivin P, Lauvergeat V, Mollier A. Phosphorus acquisition efficiency and phosphorus remobilization mediate genotype-specific differences in shoot phosphorus content in grapevine. *Tree Physiol*. Oxford University Press (OUP); 38:1742–512018;

63. Canas S, Assunção M, Brazão J, Zanol G, Eiras-Dias JE. Phenolic compounds involved in grafting incompatibility of Vitis spp: development and validation of an analytical method for their quantification. *Phytochem Anal*. 26:1–72015;

64. Prodhomme D, Valls Fonayet J, Hévin C, Franc C, Hilbert G, de Revel G, et al.. Metabolite profiling during graft union formation reveals the reprogramming of primary metabolism and the induction of

Formatted: Normal

stilbene synthesis at the graft interface in grapevine. *BMC Plant Biol*. 19:5992019;

65. Migicovsky Z, Sawler J, Money D, Eibach R, Miller AJ, Luby JJ, et al.. Genomic ancestry estimation quantifies use of wild species in grape breeding. *BMC Genomics*. 17:4782016;

66. Vitulo N, Forcato C, Carpinelli EC, Telatin A, Campagna D, D'Angelo M, et al.. A deep survey of alternative splicing in grape reveals changes in the splicing machinery related to tissue, stress condition and genotype. *BMC Plant Biol*. 14:992014;

67. Harris ZN, Kovacs LG, Londo JP. RNA-seq-based genome annotation and identification of long-noncoding RNAs in the grapevine cultivar "Riesling." *BMC Genomics*. BioMed Central; 18:9372017;

68. Williams BR, Edwards CE, Kwasniewski MT, Miller AJ. Epigenomic patterns reflect irrigation and grafting in the grapevine clone'Chambourcin'. *bioRxiv*. Cold Spring Harbor Laboratory; 2020;

69. Marasco R, Rolli E, Fusi M, Michoud G, Daffonchio D. Grapevine rootstocks shape underground bacterial microbiome and networking but not potential functionality. *Microbiome*. 6:32018;

70. Swift JF, Hall ME, Harris ZN, Kwasniewski MT, Miller AJ. Grapevine microbiota reflect diversity among compartments and complex interactions within and among root and shoot systems. bioRxiv.

71. Palumbo MC, Zenoni S, Fasoli M, Massonnet M, Farina L, Castiglione F, et al.. Integrated network analysis identifies fight-club nodes as a class of hubs encompassing key putative switch genes that induce major transcriptome reprogramming during grapevine development. *Plant Cell*. Am Soc Plant Biol; 26:4617–352014;

72. Savoi S, Wong DCJ, Arapitsas P, Miculan M, Bucchetti B, Peterlunger E, et al.. Transcriptome and metabolite profiling reveals that prolonged drought modulates the phenylpropanoid and terpenoid pathway in white grapes (Vitis vinifera L.). *BMC Plant Biol*. 16:672016;

73. Savoi S, Wong DCJ, Degu A, Herrera JC, Bucchetti B, Peterlunger E, et al.. Multi-Omics and Integrated Network Analyses Reveal New Insights into the Systems Relationships between Metabolites, Structural Genes, and Transcriptional Regulators in Developing Grape Berries (Vitis vinifera L.) Exposed to Water Deficit. *Front Plant Sci*. 8:11242017;

74. Wong DCJ, Matus JT. Constructing Integrated Networks for Identifying New Secondary Metabolic Pathway Regulators in Grapevine: Recent Applications and Future Opportunities. *Front Plant Sci*. 8:5052017;

75. Fabres PJ, Collins C, Cavagnaro TR, Rodríguez López CM. A Concise Review on Multi-Omics Data Integration for Terroir Analysis in Vitis vinifera. *Front Plant Sci*. 8:10652017;

76. Huang S, Chaudhary K, Garmire LX. More Is Better: Recent Progress in Multi-Omics Data Integration Methods. *Front Genet*. 8:842017;

77. Stein-O'Brien GL, Arora R, Culhane AC, Favorov AV, Garmire LX, Greene CS, et al.. Enter the Matrix: Factorization Uncovers Knowledge from Omics. *Trends Genet*. 34:790–8052018;

78. Fox J, Friendly M, Weisberg S. Hypothesis tests for multivariate linear models using the car package. *R J*. Citeseer; 5:39–522013;

79. R Core Team. R: A language and environment for statistical computing. Vienna, Austria;

Formatted: Normal

~~80. Lenth R, Singmann H, Love J, Others. Emmeans: Estimated marginal means, aka least-squares means. *R package version*. 12018;~~

~~81. Ripley BD. Modern applied statistics with S. Springer;~~

~~82. Wickham H. ggplot2: Elegant Graphics for Data Analysis. Springer;~~

~~83. Liaw A, Wiener M, Others. Classification and regression by randomForest. *R news*. 2:18–222002;~~

~~84. Kuhn M. Predictive Modeling with R and the caret Package. *Google Scholar*. 2013;~~

~~85. Team RC, Others. R foundation for statistical computing. *Vienna, Austria*. 32013;~~

~~86. Csardi G, Nepusz T, Others. The igraph software package for complex network research. *InterJournal, complex systems*. 1695:1–92006;~~

50. Podani J, Miklós I. Resemblance coefficients and the horseshoe effect in principal coordinates analysis. *Ecology*. Wiley; 83:3331–432002;

51. Degu A, Hochberg U, Sikron N, Venturini L, Buson G, Ghan R, et al.. Metabolite and transcript profiling of berry skin during fruit development elucidates differential regulation between Cabernet Sauvignon and Shiraz cultivars at branching points in the polyphenol pathway. *BMC Plant Biol.* 14:1882014;

52. Anesi A, Stocchero M, Dal Santo S, Commisso M, Zenoni S, Ceoldo S, et al.. Towards a scientific interpretation of the terroir concept: plasticity of the grape berry metabolome. *BMC Plant Biol.* 15:1912015;

53. Cuadros-Inostroza A, Ruíz-Lara S, González E, Eckardt A, Willmitzer L, Peña-Cortés H. GC-MS metabolic profiling of Cabernet Sauvignon and Merlot cultivars during grapevine berry development and network analysis reveals a stage- and cultivar-dependent connectivity of primary metabolites. *Metabolomics*. 12:392016;

54. Dal Santo S, Fasoli M, Negri S, D'Incà E, Vicenzi N, Guzzo F, et al.. Plasticity of the Berry Ripening Program in a White Grape Variety. *Front Plant Sci*. 7:9702016;

55. Zamboni A, Di Carli M, Guzzo F, Stocchero M, Zenoni S, Ferrarini A, et al.. Identification of putative stage-specific grapevine berry biomarkers and omics data integration into networks. *Plant Physiol.* 154:1439–592010;

56. Dal Santo S, Tornielli GB, Zenoni S, Fasoli M, Farina L, Anesi A, et al.. The plasticity of the grapevine berry transcriptome. *Genome Biol.* 14:r542013;

57. Chitwood DH, Rundell SM, Li DY, Woodford QL, Yu TT, Lopez JR, et al.. Climate and Developmental Plasticity: Interannual Variability in Grapevine Leaf Morphology. *Plant Physiol.* 170:1480–912016;

58. Chitwood DH, Mullins J, Migicovsky Z, Frank M, VanBuren R, Londo JP. Vein-to-blade ratio is an allometric indicator of climate-induced changes in grapevine leaf size and shape. bioRxiv.

59. Bravdo B. EFFECT OF MINERAL NUTRITION AND SALINITY ON GRAPE PRODUCTION AND WINE QUALITY. *Acta Horticulturae*. 512:23–302000;

Formatted: Normal

60. Brunetto G, Melo GWBDE, Toselli M, Quartieri M, Tagliavini M. The role of mineral nutrition on yields and fruit quality in grapevine, pear and apple. *Rev Bras Frutic*. FapUNIFESP (SciELO); 37:1089–1042015;

61. Gautier A, Cookson SJ, Hevin C, Vivin P, Lauvergeat V, Mollier A. Phosphorus acquisition efficiency and phosphorus remobilization mediate genotype-specific differences in shoot phosphorus content in grapevine. *Tree Physiol*. Oxford University Press (OUP); 38:1742–512018;

62. Canas S, Assunção M, Brazão J, Zanol G, Eiras-Dias JE. Phenolic compounds involved in grafting incompatibility of Vitis spp: development and validation of an analytical method for their quantification. *Phytochem Anal*. 26:1–72015;

63. Prodhomme D, Valls Fonayet J, Hévin C, Franc C, Hilbert G, de Revel G, et al.. Metabolite profiling during graft union formation reveals the reprogramming of primary metabolism and the induction of stilbene synthesis at the graft interface in grapevine. *BMC Plant Biol*. 19:5992019;

64. Migicovsky Z, Sawler J, Money D, Eibach R, Miller AJ, Luby JJ, et al.. Genomic ancestry estimation quantifies use of wild species in grape breeding. *BMC Genomics*. 17:4782016;

65. Vitulo N, Forcato C, Carpinelli EC, Telatin A, Campagna D, D'Angelo M, et al.. A deep survey of alternative splicing in grape reveals changes in the splicing machinery related to tissue, stress condition and genotype. *BMC Plant Biol*. 14:992014;

66. Harris ZN, Kovacs LG, Londo JP. RNA-seq-based genome annotation and identification of long-noncoding RNAs in the grapevine cultivar "Riesling." *BMC Genomics*. BioMed Central; 18:9372017;

67. Williams BR, Edwards CE, Kwasniewski MT, Miller AJ. Epigenomic patterns reflect irrigation and grafting in the grapevine clone'Chambourcin'. *bioRxiv*. Cold Spring Harbor Laboratory; 2020;

68. Marasco R, Rolli E, Fusi M, Michoud G, Daffonchio D. Grapevine rootstocks shape underground bacterial microbiome and networking but not potential functionality. *Microbiome*. 6:32018;

69. Swift JF, Hall ME, Harris ZN, Kwasniewski MT, Miller AJ. Grapevine microbiota reflect diversity among compartments and complex interactions within and among root and shoot systems. bioRxiv.

70. Palumbo MC, Zenoni S, Fasoli M, Massonnet M, Farina L, Castiglione F, et al.. Integrated network analysis identifies fight-club nodes as a class of hubs encompassing key putative switch genes that induce major transcriptome reprogramming during grapevine development. *Plant Cell*. Am Soc Plant Biol; 26:4617–352014;

71. Savoi S, Wong DCJ, Arapitsas P, Miculan M, Bucchetti B, Peterlunger E, et al.. Transcriptome and metabolite profiling reveals that prolonged drought modulates the phenylpropanoid and terpenoid pathway in white grapes (Vitis vinifera L.). *BMC Plant Biol*. 16:672016;

72. Savoi S, Wong DCJ, Degu A, Herrera JC, Bucchetti B, Peterlunger E, et al.. Multi-Omics and Integrated Network Analyses Reveal New Insights into the Systems Relationships between Metabolites, Structural Genes, and Transcriptional Regulators in Developing Grape Berries (Vitis vinifera L.) Exposed to Water Deficit. *Front Plant Sci*. 8:11242017;

73. Wong DCJ, Matus JT. Constructing Integrated Networks for Identifying New Secondary Metabolic Pathway Regulators in Grapevine: Recent Applications and Future Opportunities. *Front Plant Sci*. 8:5052017;

74. Fabres PJ, Collins C, Cavagnaro TR, Rodríguez López CM. A Concise Review on Multi-Omics Data Integration for Terroir Analysis in Vitis vinifera. *Front Plant Sci*. 8:10652017;

75. Huang S, Chaudhary K, Garmire LX. More Is Better: Recent Progress in Multi-Omics Data Integration Methods. *Front Genet*. 8:842017;

76. Stein-O'Brien GL, Arora R, Culhane AC, Favorov AV, Garmire LX, Greene CS, et al.. Enter the Matrix: Factorization Uncovers Knowledge from Omics. *Trends Genet*. 34:790–8052018;

77. Fox J, Friendly M, Weisberg S. Hypothesis tests for multivariate linear models using the car package. *R J*. Citeseer; 5:39–522013;

78. R Core Team. R: A language and environment for statistical computing. Vienna, Austria;

79. Lenth R, Singmann H, Love J, Others. Emmeans: Estimated marginal means, aka least-squares means. *R package version*. 12018;

80. Ripley BD. Modern applied statistics with S. Springer;

81. Wickham H. ggplot2: Elegant Graphics for Data Analysis. Springer;

82. Liaw A, Wiener M, Others. Classification and regression by randomForest. *R news*. 2:18–222002;

83. Kuhn M. Predictive Modeling with R and the caret Package. *Google Scholar*. 2013;

84. Team RC, Others. R foundation for statistical computing. *Vienna, Austria*. 32013;

85. Csardi G, Nepusz T, Others. The igraph software package for complex network research. *InterJournal, complex systems*. 1695:1–92006;

Formatted: Normal

Figure 1

Click here to access/download;Figure;Figure 1.pdf

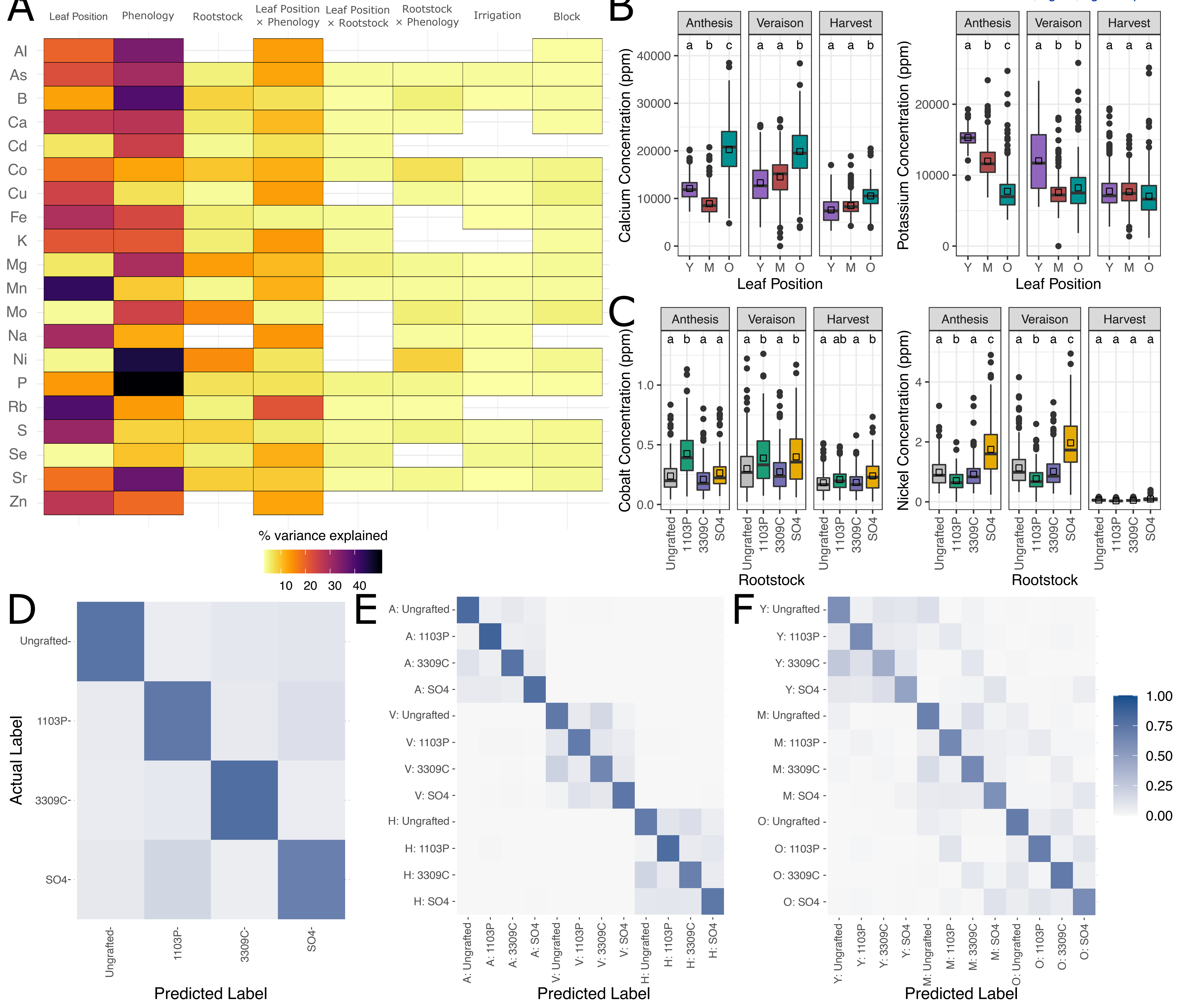

Figure 2

Click here to access/download;Figure;Figure 2.pdf

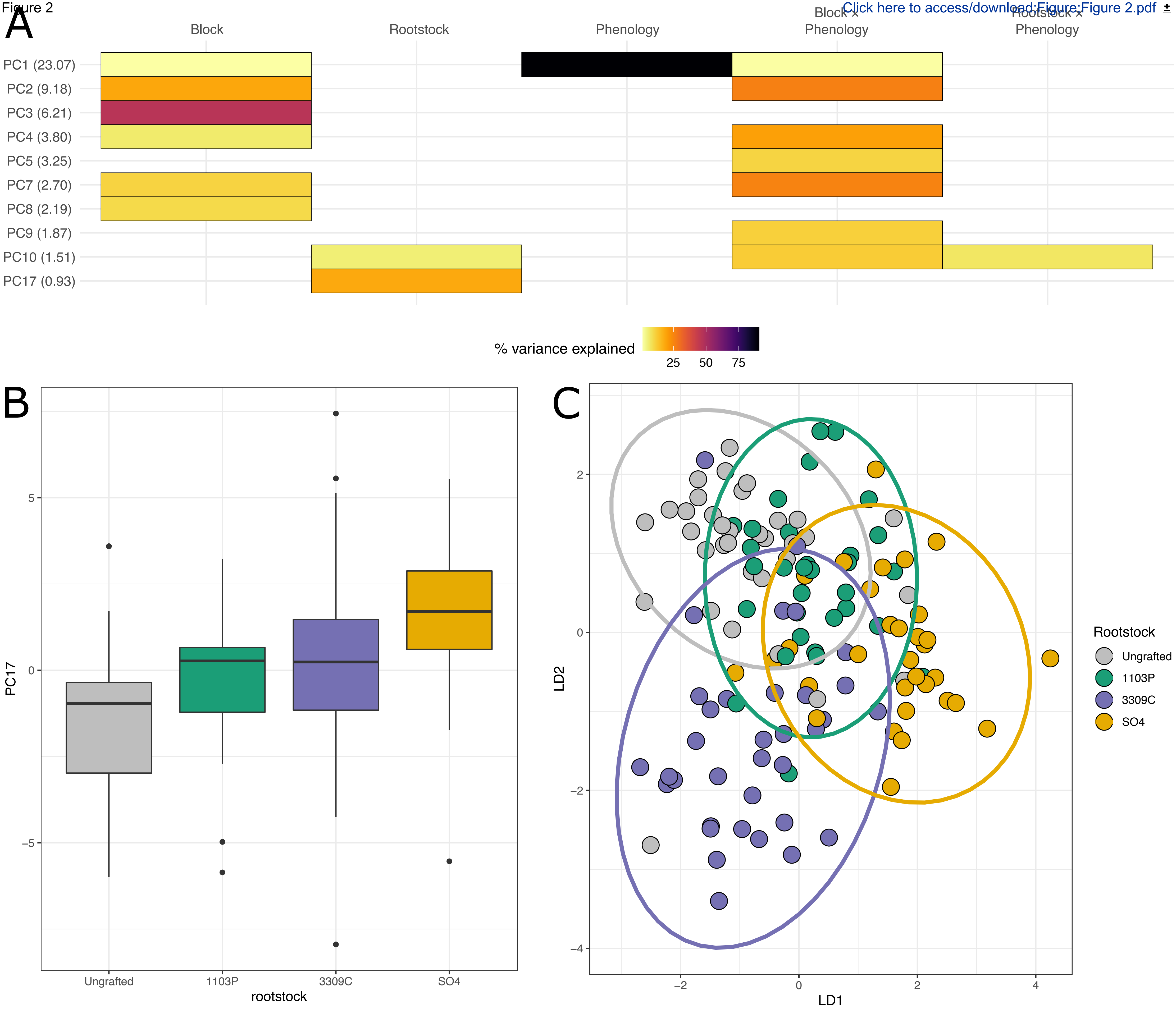

Figure 3

Click here to access/download;Figure;Figure 3.pdf

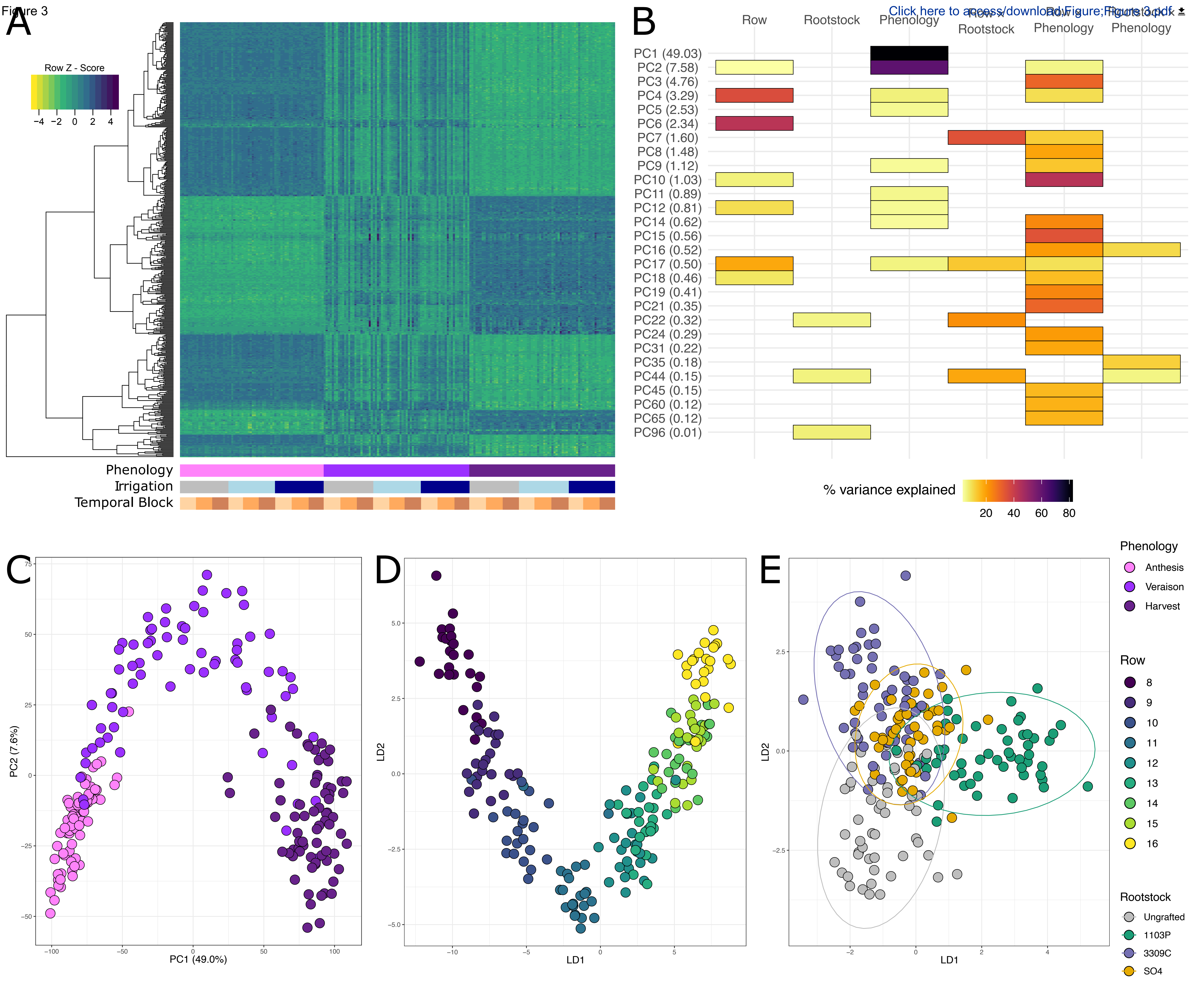

Figure 4
Click here to access/download;Figure;Figure 4.pdf

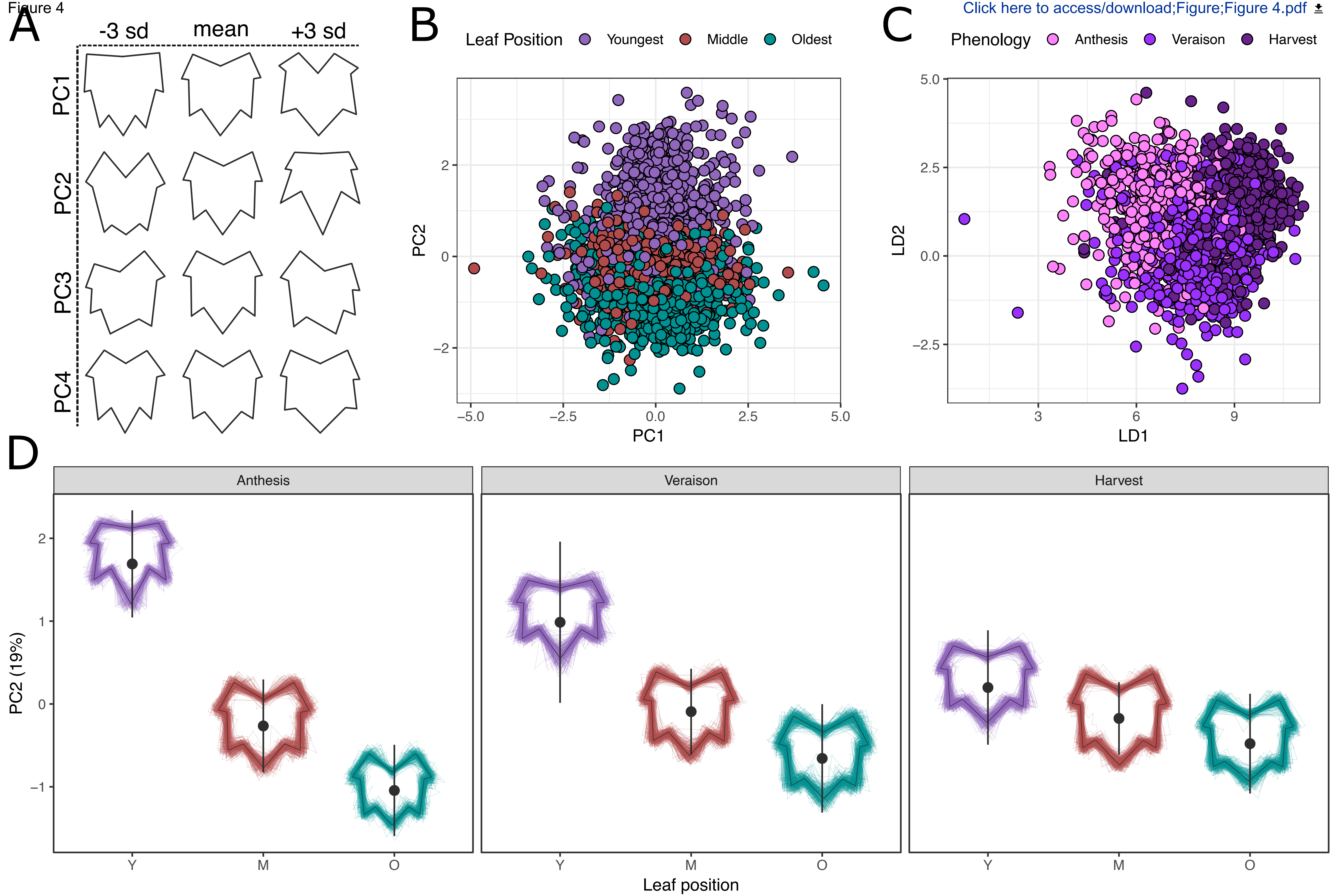

Figure 5

Click here to access/download;Figure;Figure 5.pdf

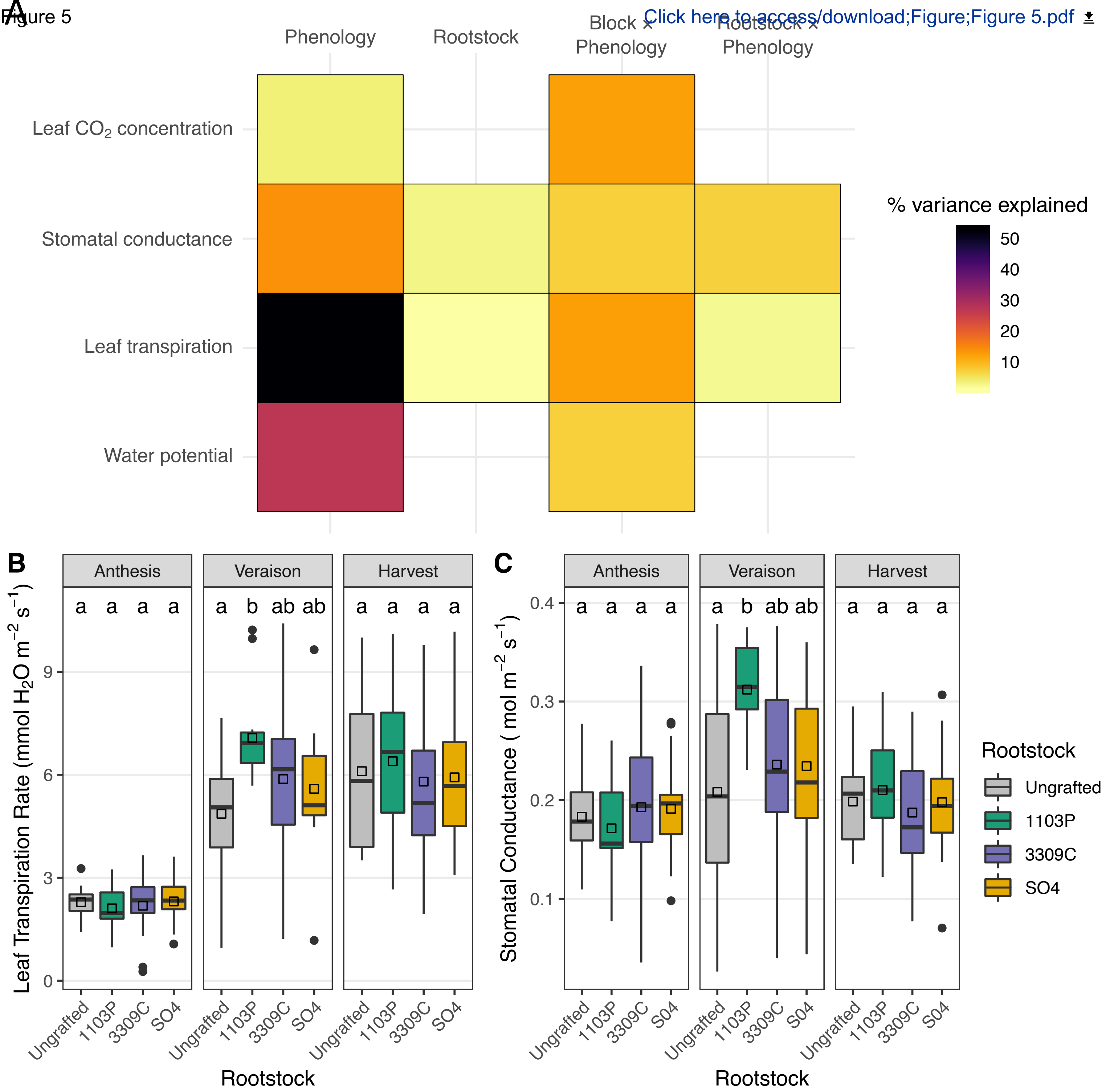

Figure 6

Click here to access/download;Figure;Figure 6.pdf

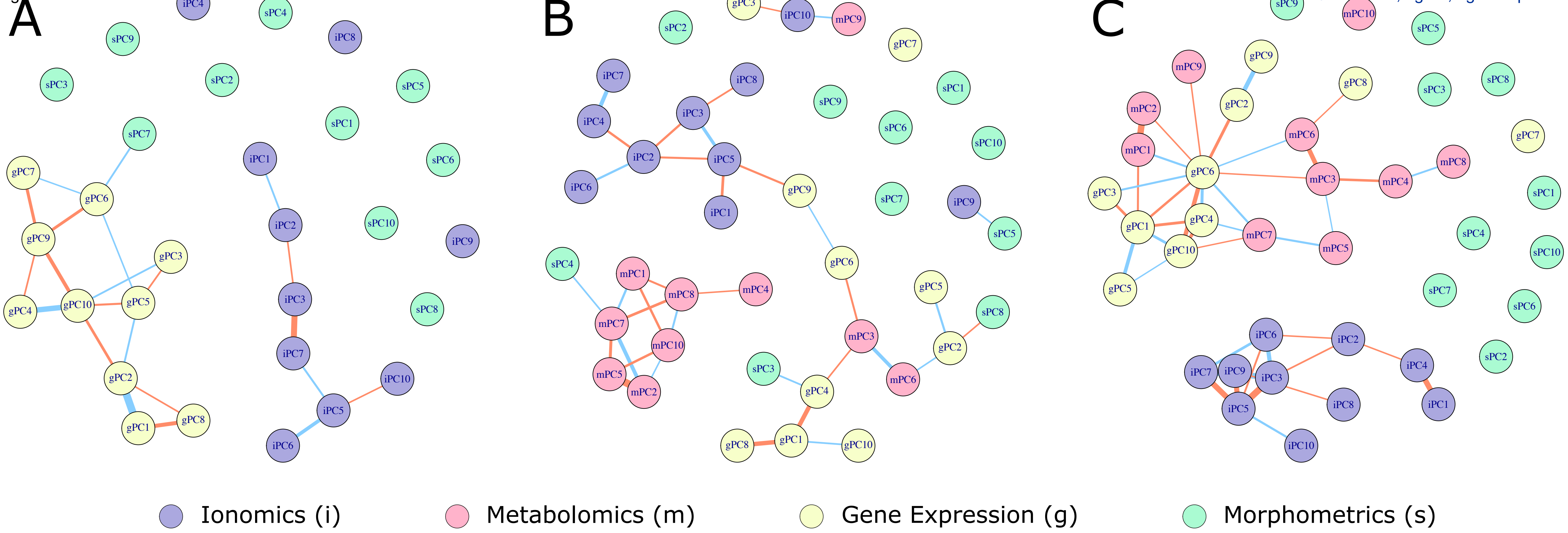

Supplemental Figure 1

Click here to access/download
Supplementary Material
Supplemental Figure 1.pdf

Supplemental Figure 2

Click here to access/download
Supplementary Material
Supplemental Figure 2.pdf

Supplemental Figure 3

Click here to access/download
Supplementary Material
Supplemental Figure 3.pdf

Supplemental Figure 4

Click here to access/download
Supplementary Material
Supplemental Figure 4.pdf

Supplemental Figure 5

Click here to access/download
Supplementary Material
Supplemental Figure 5.pdf

Supplemental Figure 6

Click here to access/download
Supplementary Material
Supplemental Figure 6.pdf

Supplemental Figure 7

Click here to access/download
Supplementary Material
Supplemental Figure 7.pdf

Click here to access/download
Supplementary Material
Supplemental Note 1.pdf

Personal Cover Click here to access/download;Personal Cover;MV2017Leaves_GigaScience_AuthorResponse_2.docx

Dr. Nicole Nogoy,

We are happy to re-submit our revised manuscript (GIGA-D-21-00137) for review. Again, we would like to thank the reviewers for the careful review of the manuscript and thoughtful comments.

We have made changes to the manuscript in order to address the comments by the reviewers. Most notably, we included comments in the manuscript to address potential intrinsic features of the rootstock that might be driving the changes we saw in the ionome, and we addressed our tradeoff between sample number and read depth for the RNAseq analyses.

In addition, we responded to comments by reviewers 1 and 4 about integrative analyses, typos, and proper data/script placement. In the response below, we also respond to editor comments to express our willingness to share data and scripts as broadly as is needed.

We appreciate the time that everyone has spent looking at this manuscript, and we thank the reviews and editor for their hard work so far. We look forward to hearing back.

Best,
Zachary N Harris and Allison J Miller

Black Text = Reviewer Comments

Grey Text = Author Response

Note to all: Microsoft Word on macOS does not allow correct continuous line numbering with “track changes” on. All referenced line numbers were identified such that they were continuous. If line numbers appear way off, try changing “All Markup” to “Simple Markup” under the Review tab to align the line numbers.

All revisions from this round are labelled Revision_2.

Editor Comments:

With regards to Reviewer #4 comments and Github - if the manuscript is deemed acceptable for publication, GigaScience will always take snapshots and host that, along with other supporting data and metadata under a CC0 license. So despite the reviewer's' concern about GitHub not being a permanent repository, there will be copies permanent in our open repository, GigaDB.

Response: We thank the editor for their work on this manuscript. We are happy to have additional copies of all of our scripts and data sets hosted redundantly across multiple repositories. Our intention with GitHub was to store the analysis scripts as permanent versions of record. As we do not come from software development, we were using GitHub as a convenient home rather than as a live repository for ongoing projects. One additional note: we uploaded all metabolomics data to Metabolites, but have not received a response from that submission. We would be happy to store an additional copy on GigaDB, if appropriate.

Reviewer 1:

Relevant methodological information that was missing from the previous submission has been added to the revised manuscript by Harris and co-workers, which enables a more conscious interpretation of the results. Experimental limitations and external sources of variation have also been considered when discussing the results. In addition, cross-check of expected expression profiles for a selection of genes has been included as a validation of the RNA-seq experiment reliability.
Response: We thank the reviewer for their careful review and re-review of the manuscript. Comments made by reviewers have considerably strengthened the manuscript and we really appreciate it.

Considering all the information, despite a huge multilevel dataset was generated, its value is limited by experimental design deficiencies recognized by the authors (e.g.: only one year of study under field conditions, noise of environmental/circadian variation during extensive physiological phenotyping and RNA-seq sampling throughout relatively

long periods of the day, theoretically low power of the RNA-seq experiment due to relatively low read depth and low replication in some comparisons with only two replicates). Altogether, the manuscript is mostly descriptive of general differences rather than conclusive. Some of the main observations have already been documented before, such as the idea that rootstock genotype affects scion leaf phenotypes. Regardless, in the current version of the manuscript, the study and its limitations are fairly presented by the authors in a manner that would be acceptable for publication if the journal considers the dataset of value in spite of these experimental limitations. Besides this general concern, I would only have a few minor comments to this version:

1. The dataset might still be undermined as only general descriptive differences are presented as conclusions, but nothing about their possible origin is mentioned. For instance, what are the known intrinsic features of the compared rootstocks according to the bibliography that could determine the observed differences in ionic composition? How could these rootstock-determined differences in ion accumulation affect vine performance? Similar questions would arise for other differences observed.

- Response: Thank you for this comment, and we share a strong interest in understanding intrinsic features of rootstocks that affect the observed differences in the grafted scion. Studies that begin to get at these questions are underway within our research team now, but unfortunately are not completed and not included in this manuscript. To address the reviewer comments here, we specified that, especially in the case of the ionome, the differences are likely due to the genetics/ pedigree of the rootstock on L521-523. Additional comments added in the last round of revision explain how we are presently unsure how individual ions map to aspects of vine performance. We know even less about the other phenotypes. Future analyses using the data set we presented, additional data that were beyond the scope of leaf phenotyping, and future data can and should address this type of question.

2. It could be more specifically pointed out that lack of DEGs in some RNA-seq comparisons could be due to the experimental limitations (e.g.: low replication and 4.1 M read depth below the minimum recommended 5 M) rather than to a real lack of effect of rootstock genotype.

- Response: Agreed. We added a note to the Data Description that we opted to sequence more samples at the cost of some read depth which does limit our power to detect some low-expression genes on L195-196. We recognize that replication is low for high order interactions (rootstock:row:phenology) due to only sampling two vines per cell. Because of this low replication, we did not interpret such effects because they would be underpowered. However we sampled 36 cells at each time point for a total of 216 samples (with a few removed for poor

sequencing), so lower order interactions and main effects were derived from much larger pools of clonally replicated samples. Specific details on this can be found in response to Reviewer 2 and 4 in the first revision.

3. The value of including PC covariation networks would be scarce if the results are not reliable enough for interpreting the inter-connection identified between the responsible specific metabolites, ions, genes, etc.

- Response: It's true, and we agree that any issues present in individual data sets will percolate into integrative analyses. Having said that, we are confident in the individual datasets and in our approach using those datasets in PC covariation networks. Focusing on PCs from each modality allowed us to capture the highest levels of variation to see how those PCs relate across modalities. We chose this analysis so that no particular modality was over-weighted and so that we could narrow down where interesting correlations lie such that we can design and craft better future studies. We recognize this approach has limitations, but after exploring many different potential options we felt this was the most appropriate given the data and the questions.

4. Several typos should be corrected in the newly added text.

- Response: We thank the reviewer for the close reading of the text. We have edited the manuscript for typos, grammar, and tense.

Reviewer 2:

I was pleased to review the resubmitted manuscript by Harris and co-workers, who have responded to my original review. The Authors have clarified a number of points regarding the RNAseq experiments including RNA extraction methods, and the tissue type that was used. More information has been added to the methods that would aid reproducibility. Additional statistics have been applied to Figures 1 and 5. Numerous formatting and grammatical changes have been made that improve the readability of the manuscript. Additional supporting references have been provided. While not all of my suggestions were included, I accept the authors responses to my original review. I have no further concerns and recommend the manuscript for publication in GigaScience.

- Response: We thank the reviewer for their careful considerations of our manuscript. The manuscript has been considerably improved thanks to the reviewer's comments.

Reviewer 3:

I found that the Authors clearly improved the ms which might be suitable for publication

- Response: We thank the reviewer for their careful considerations of our manuscript. We especially thank the reviewer for comments on improving figures. The presentation of our work was improved by the reviewer's comments.

Reviewer 4:

- I saw the editor comments about appropriate data storage, but I disagree with those comments to the authors.
- Github is not a permanent repository and as such it's not true that it's the most appropriate place to share scripts for a publication. It is only suitable as a place for collaboration. As the authors make changes, the version of record for this manuscript will no longer be available, and the authors could delete it at any time. The publication versions should be separately reposited in a permanent repository. In my opinion, if a script is meant to be a version of record and also living, then a link to both the permanent repository and to GitHub can be given.
- I am not sure what is meant by 'large-scale' data. Figshare is a general use repository that I only recommended since the authors already were using it. It can host single files up to 5 gb in size, provides unlimited public space, and provides a DOI. So what exactly is unsuitable?
- Zenodo is another free option, and there is Data Dryad and the Data Commons.

- Response: We thank the reviewer for their careful considerations of our manuscript. We are happy to share our data and scripts in any way requested. Our intention was to use Github as a repository for a version of record, but we recognize that it is not a perfect solution. We are happy that Gigascience will host snapshots so that there is no potential for misuse. If the reviewer would like an additional home for the scripts we would be very happy to do that.
