# Supplementary figures and images for: Multi-dimensional leaf phenotypes reflect root system genotype in grafted grapevine over the growing season
Source: Gigascience. 2021 Dec 29;10(12):giab087. doi: 10.1093/gigascience/giab087 (PMC8716362; doi:10.1093/gigascience/giab087)

A

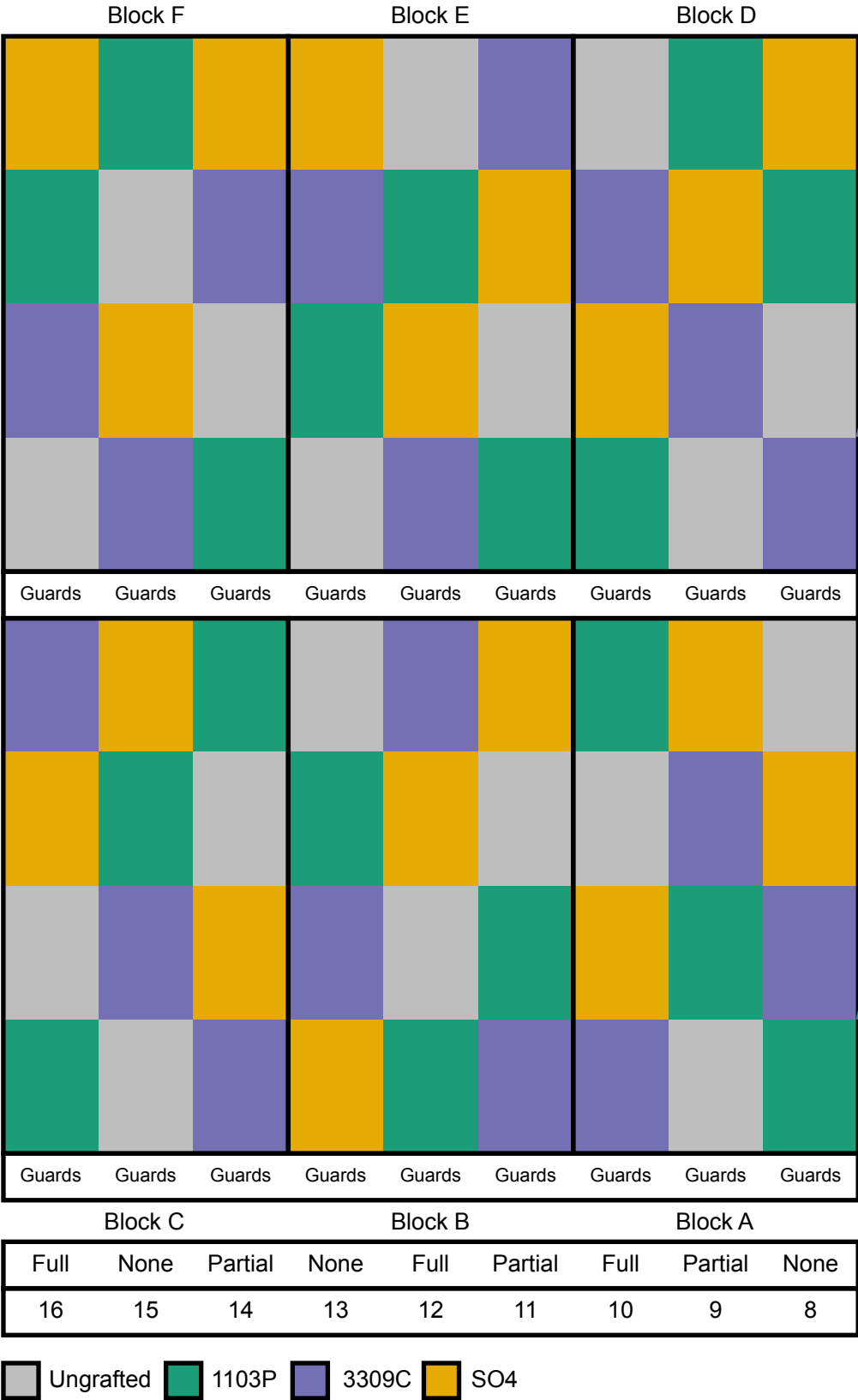

B

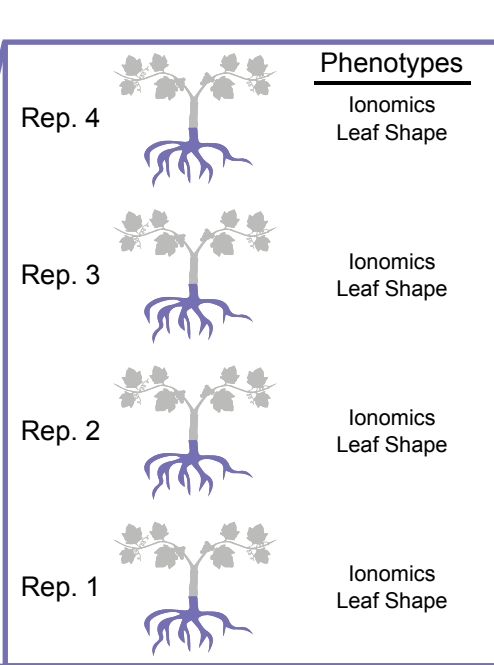

C

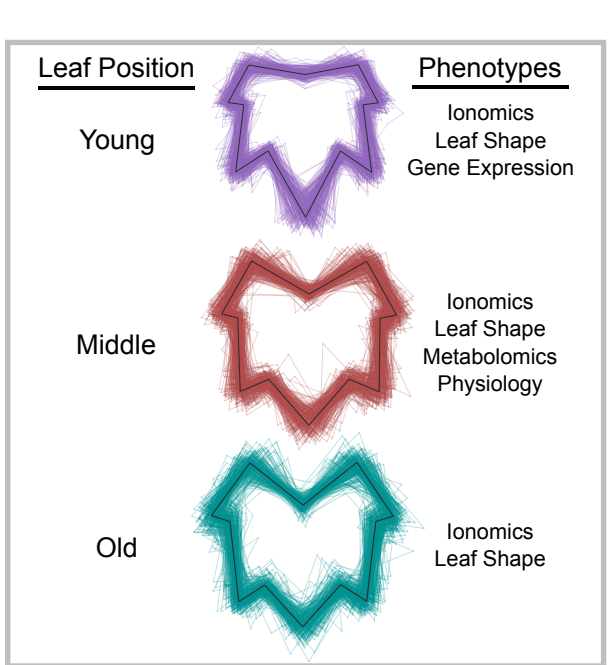

D

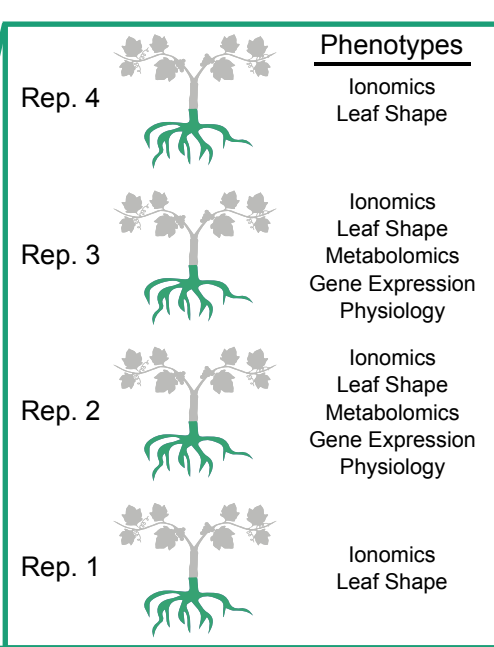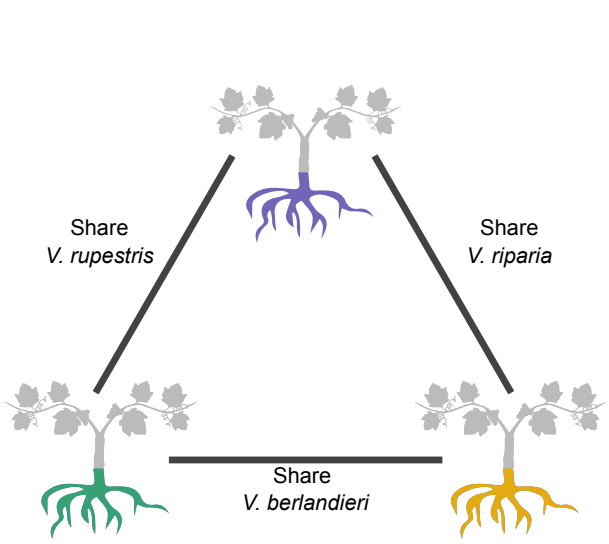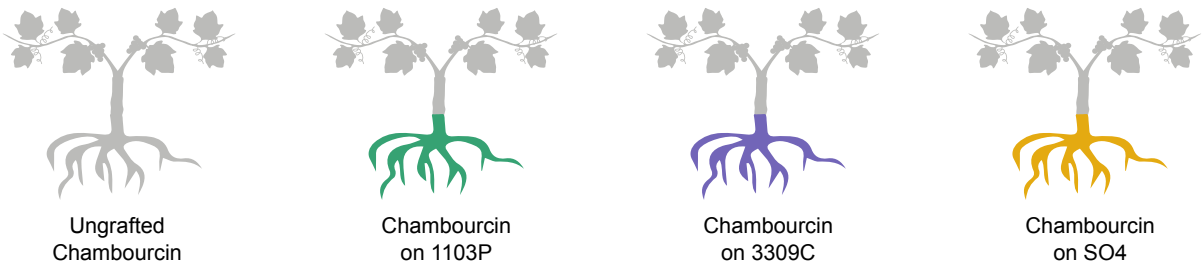

Supplement: giab087_Supplemental_Files [file giab087_supplemental_files.zip › Supplemental Figure 1.pdf]

# Anthesis

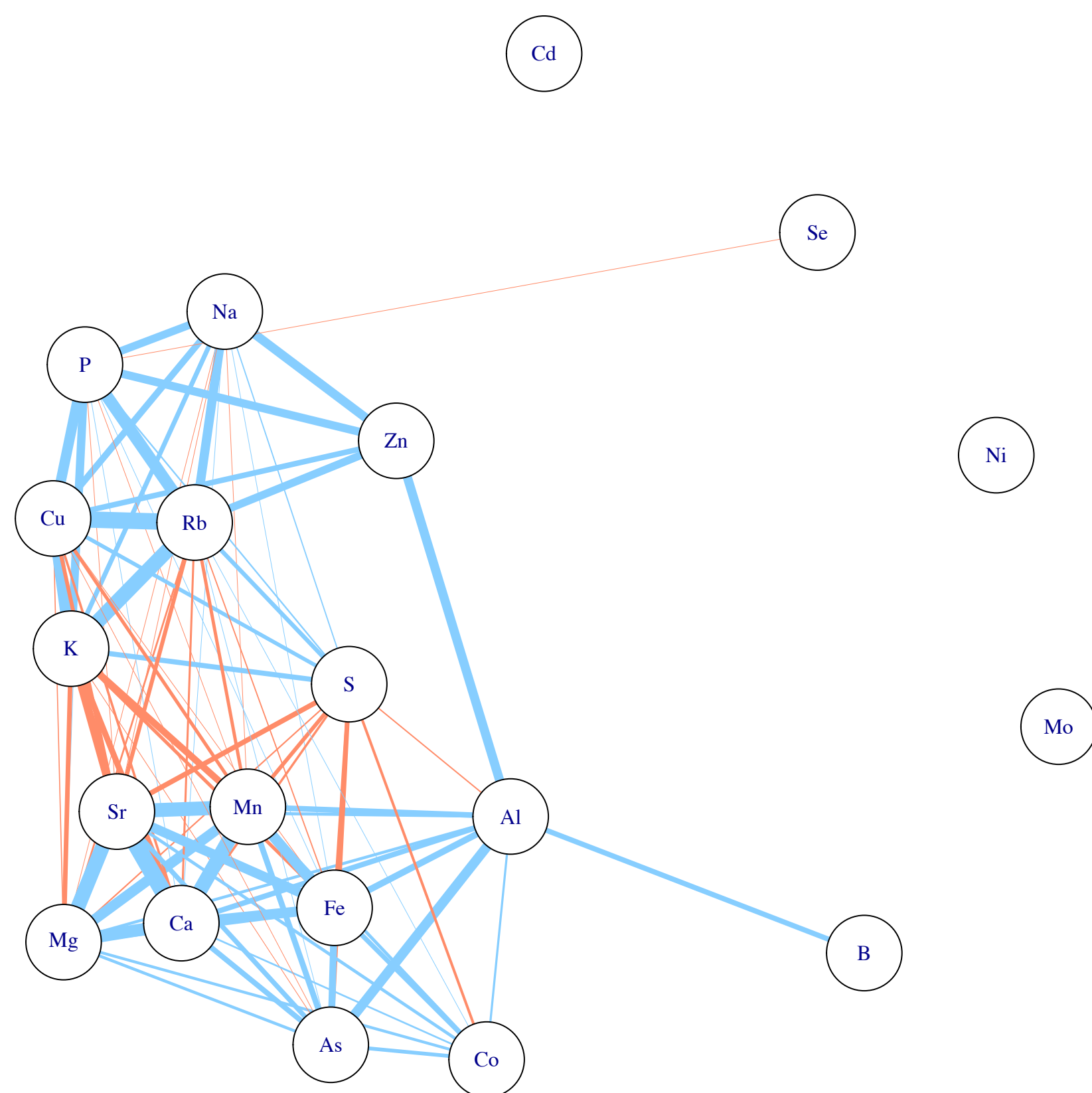

# Veraison

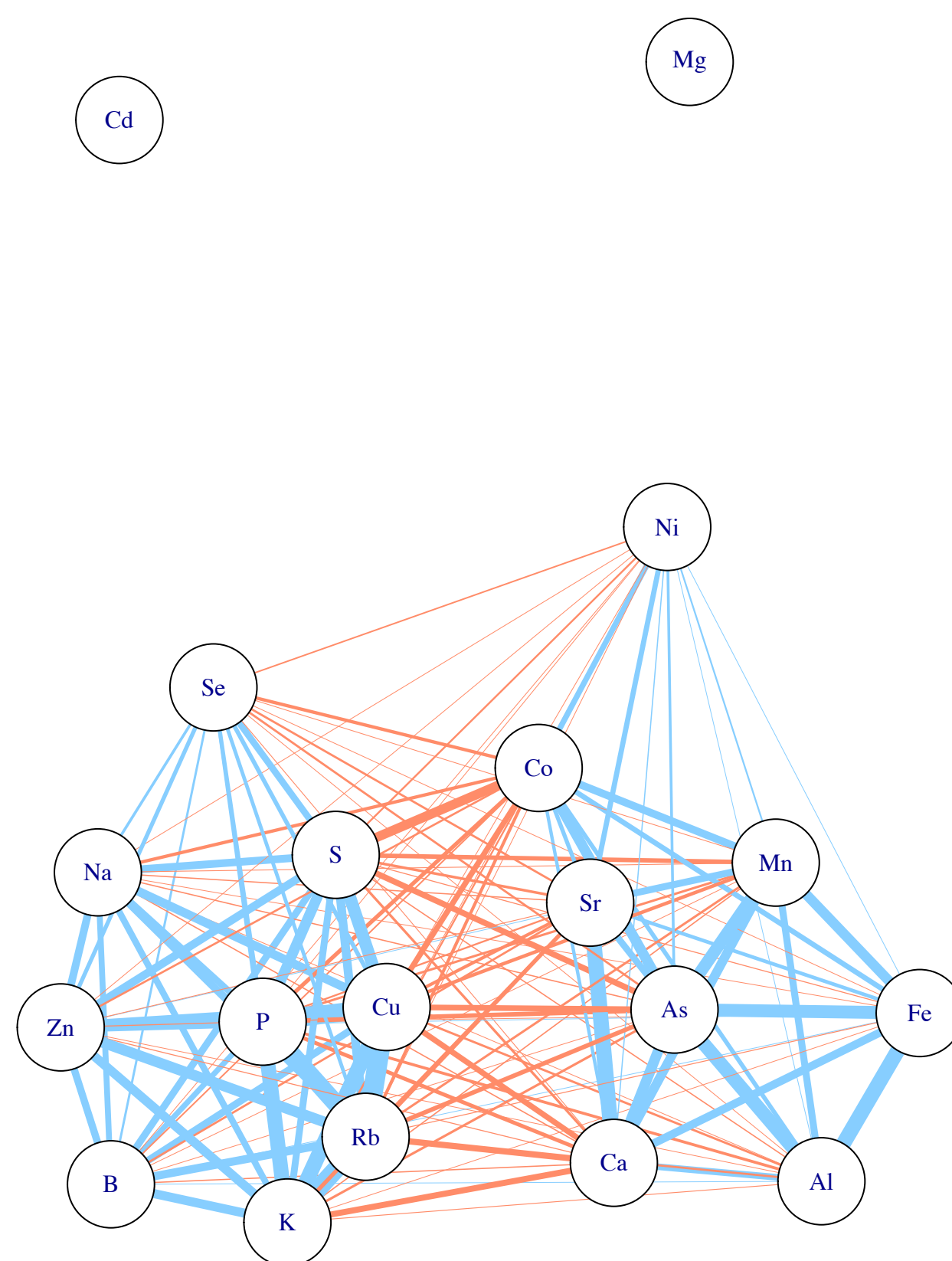

# Harvest

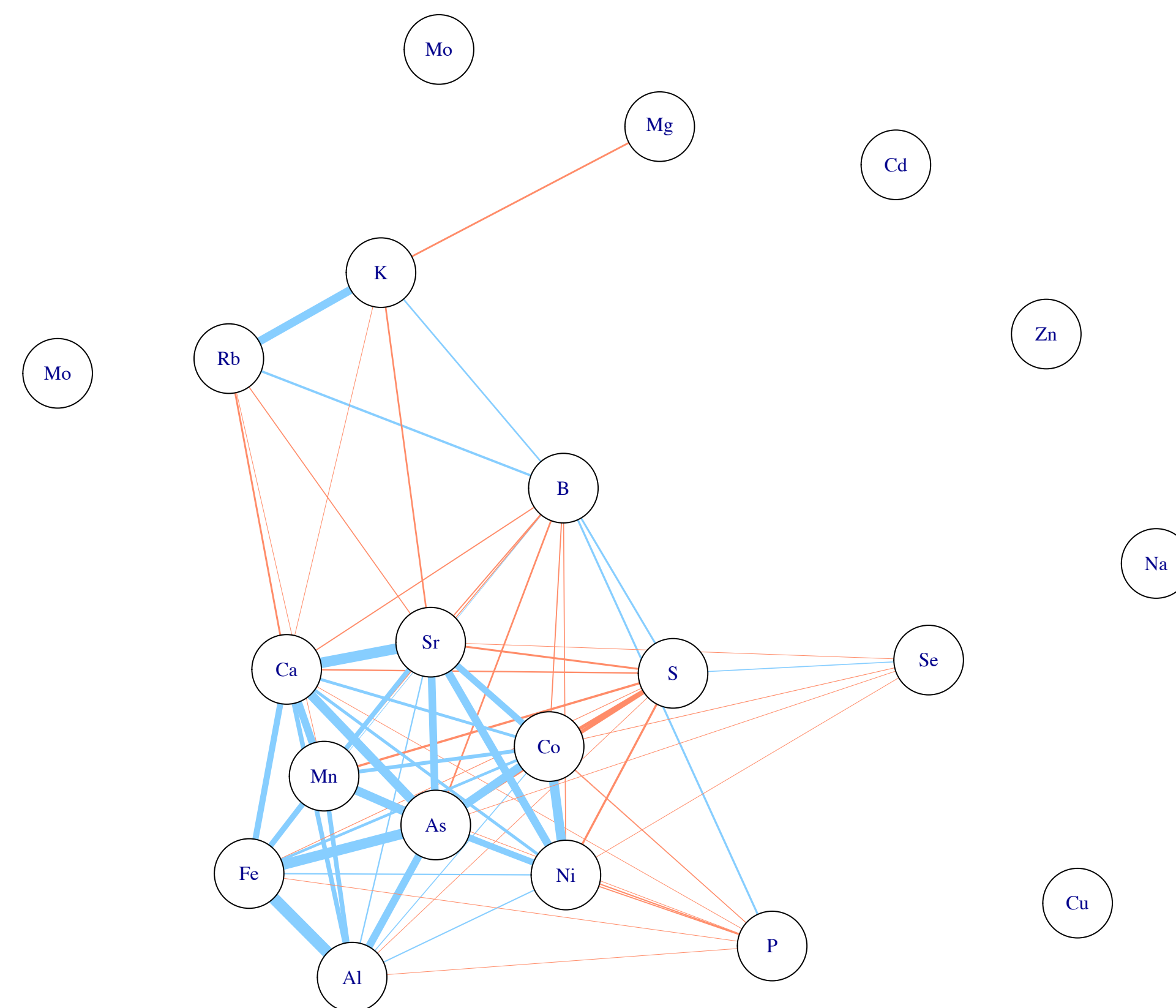

# Youngest

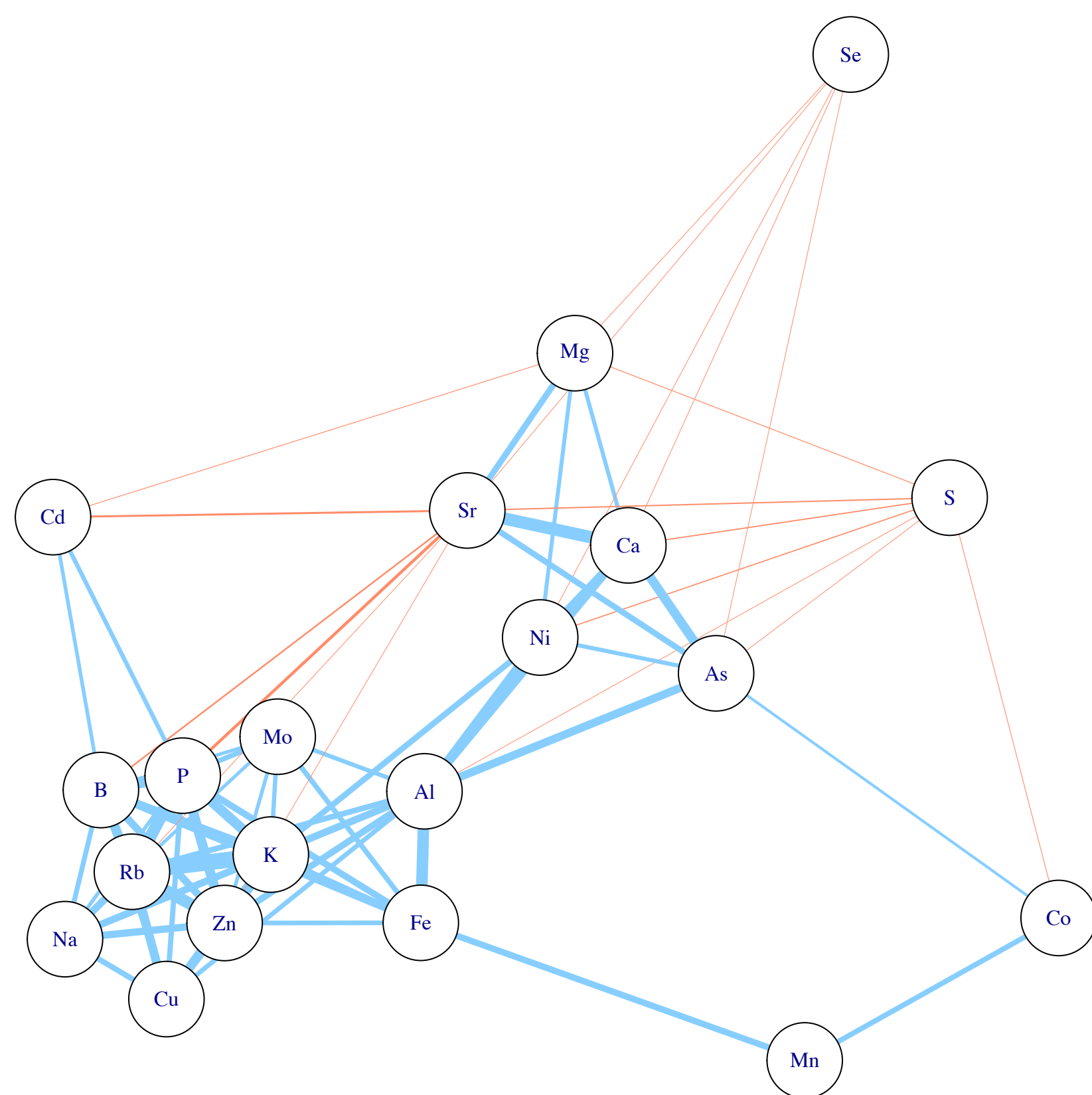

# Middle

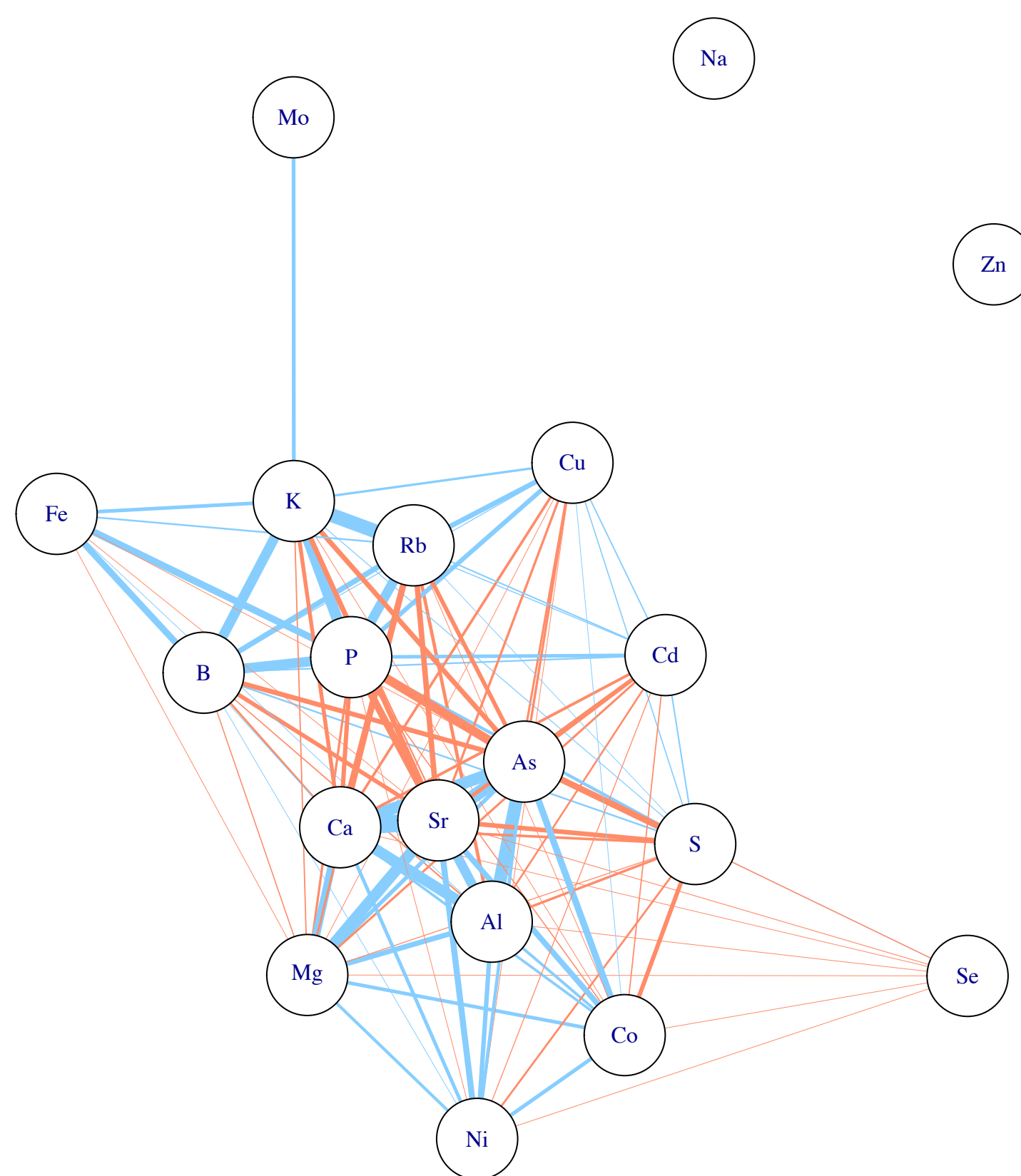

# Oldest

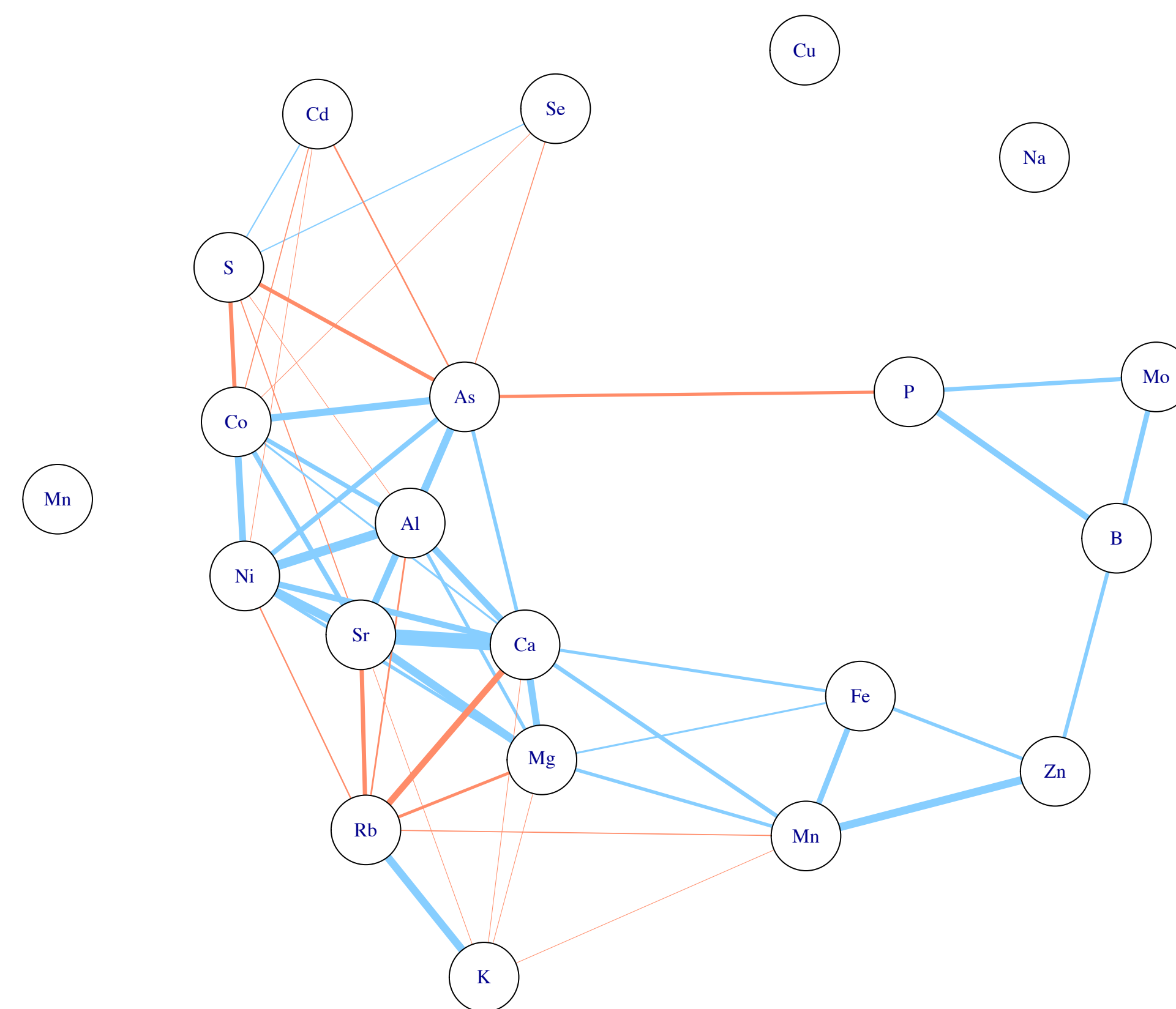

Supplement: giab087_Supplemental_Files [file giab087_supplemental_files.zip › Supplemental Figure 3.pdf]

A

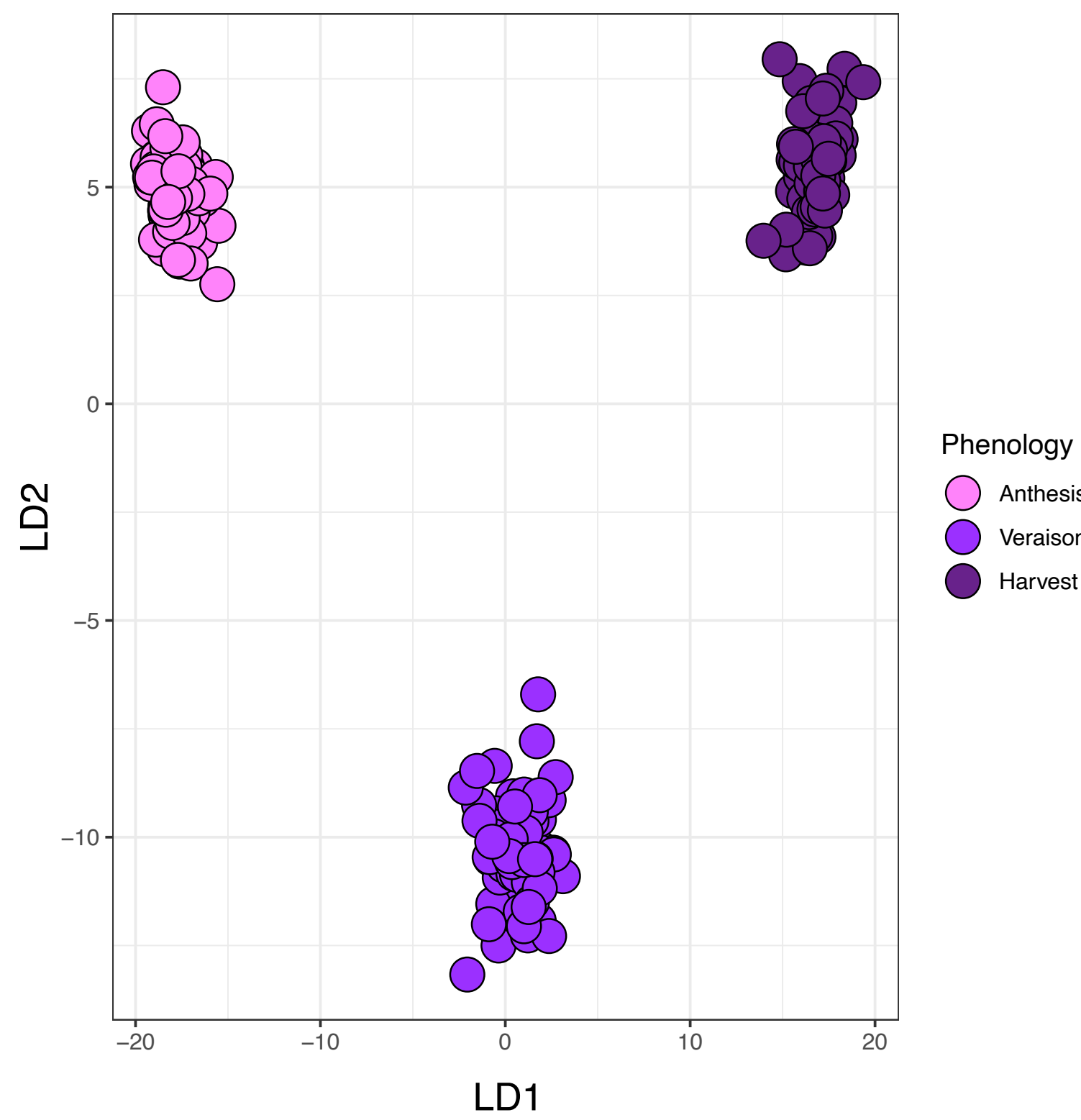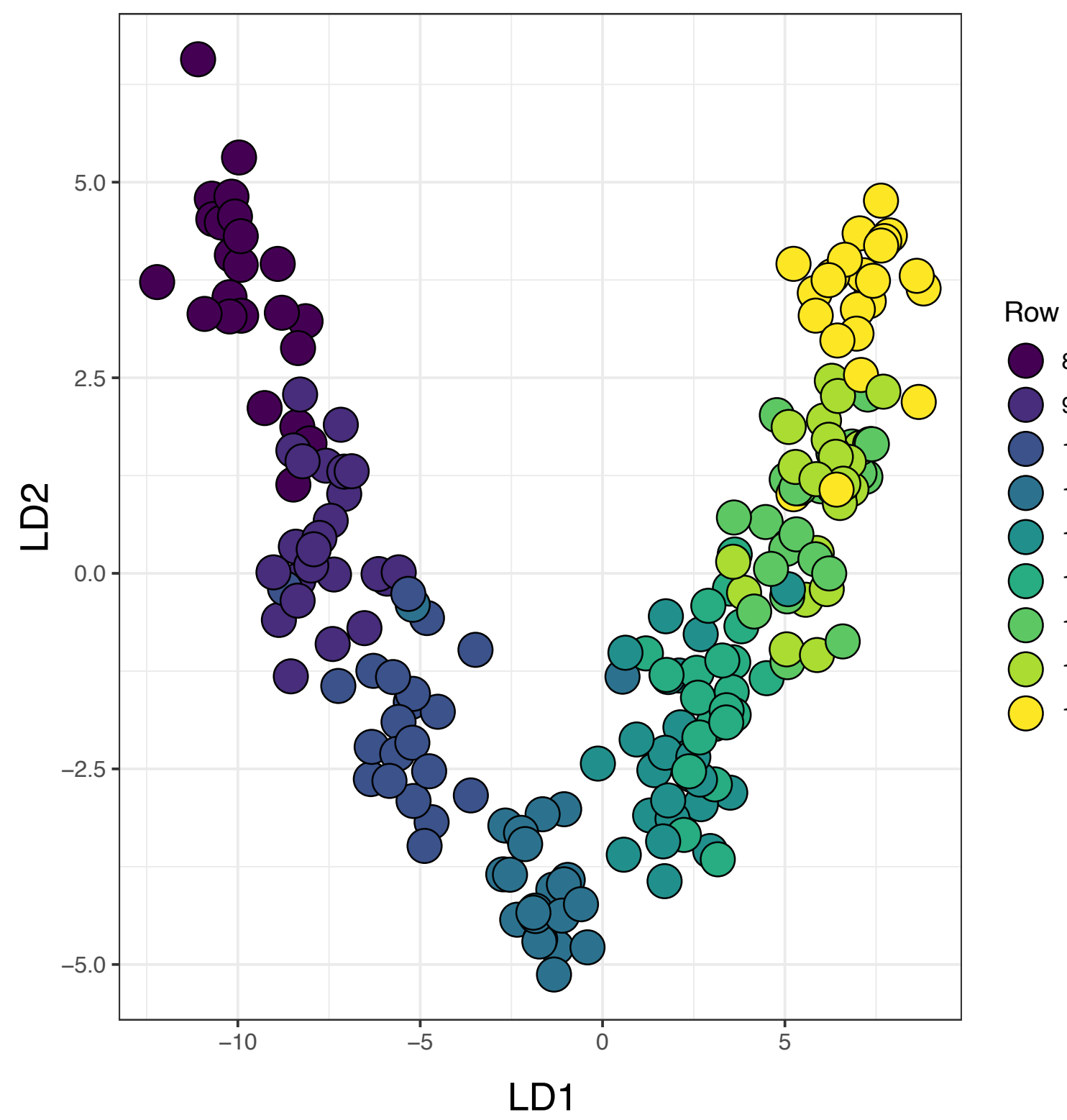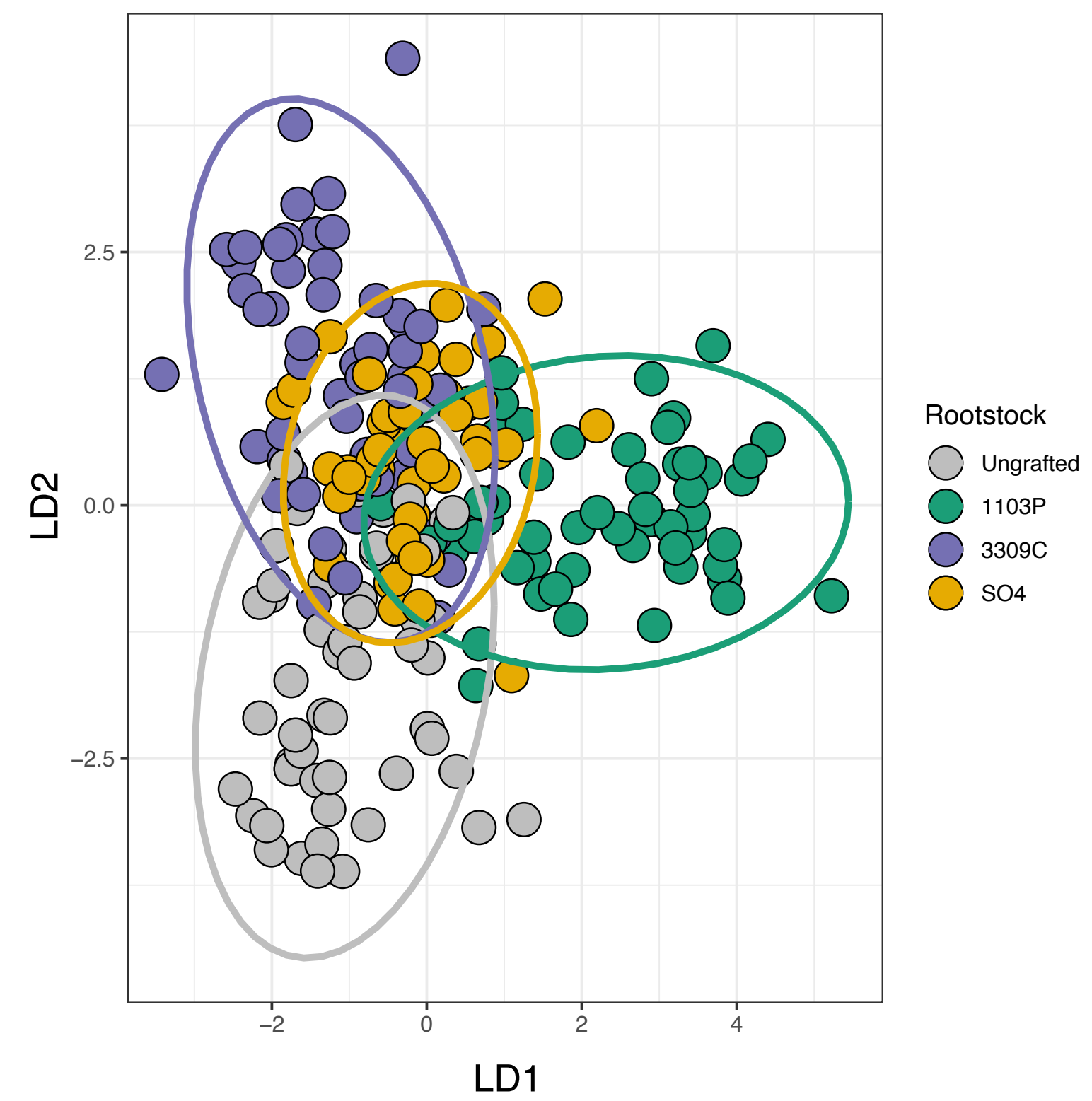

B

LD1

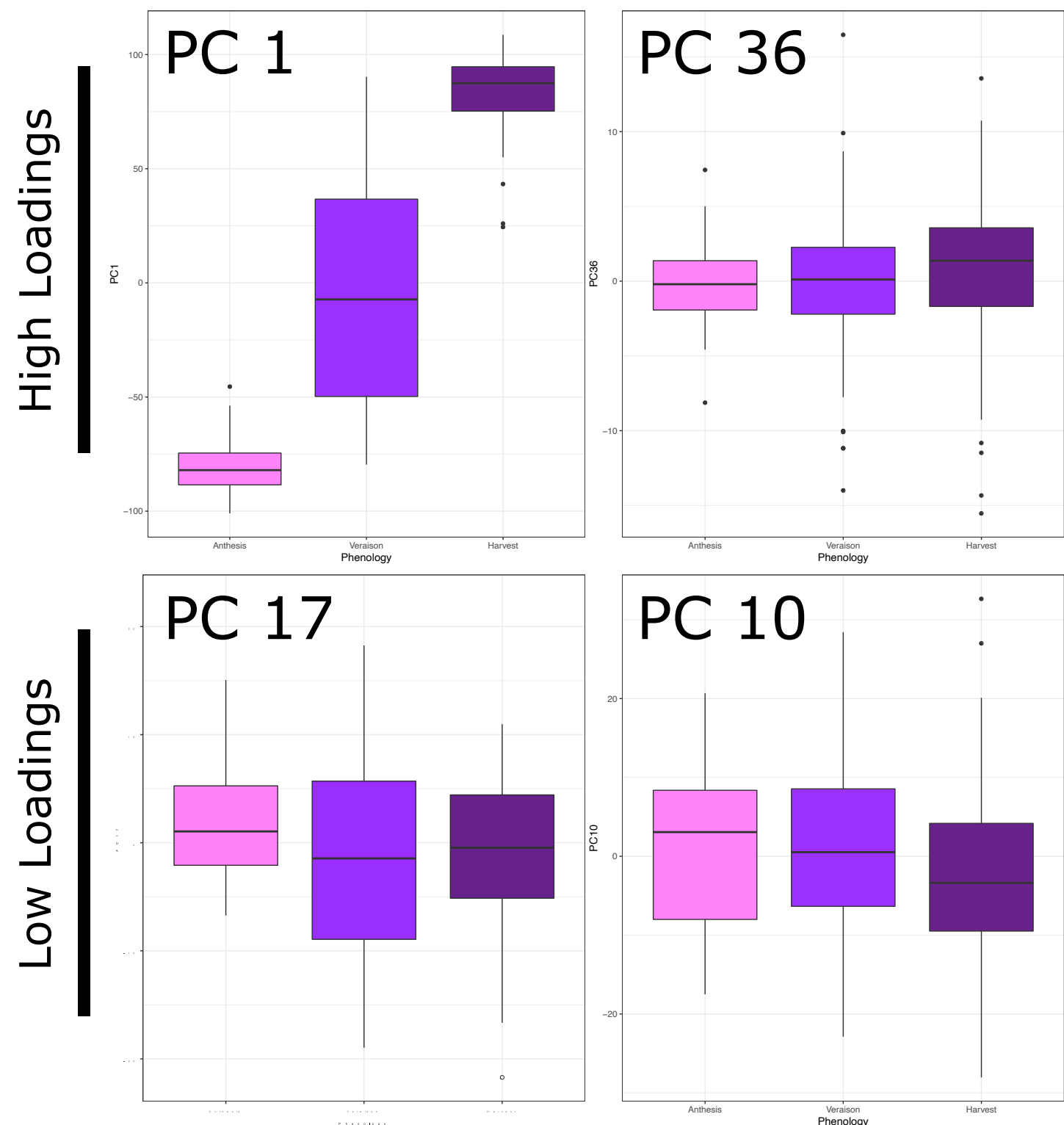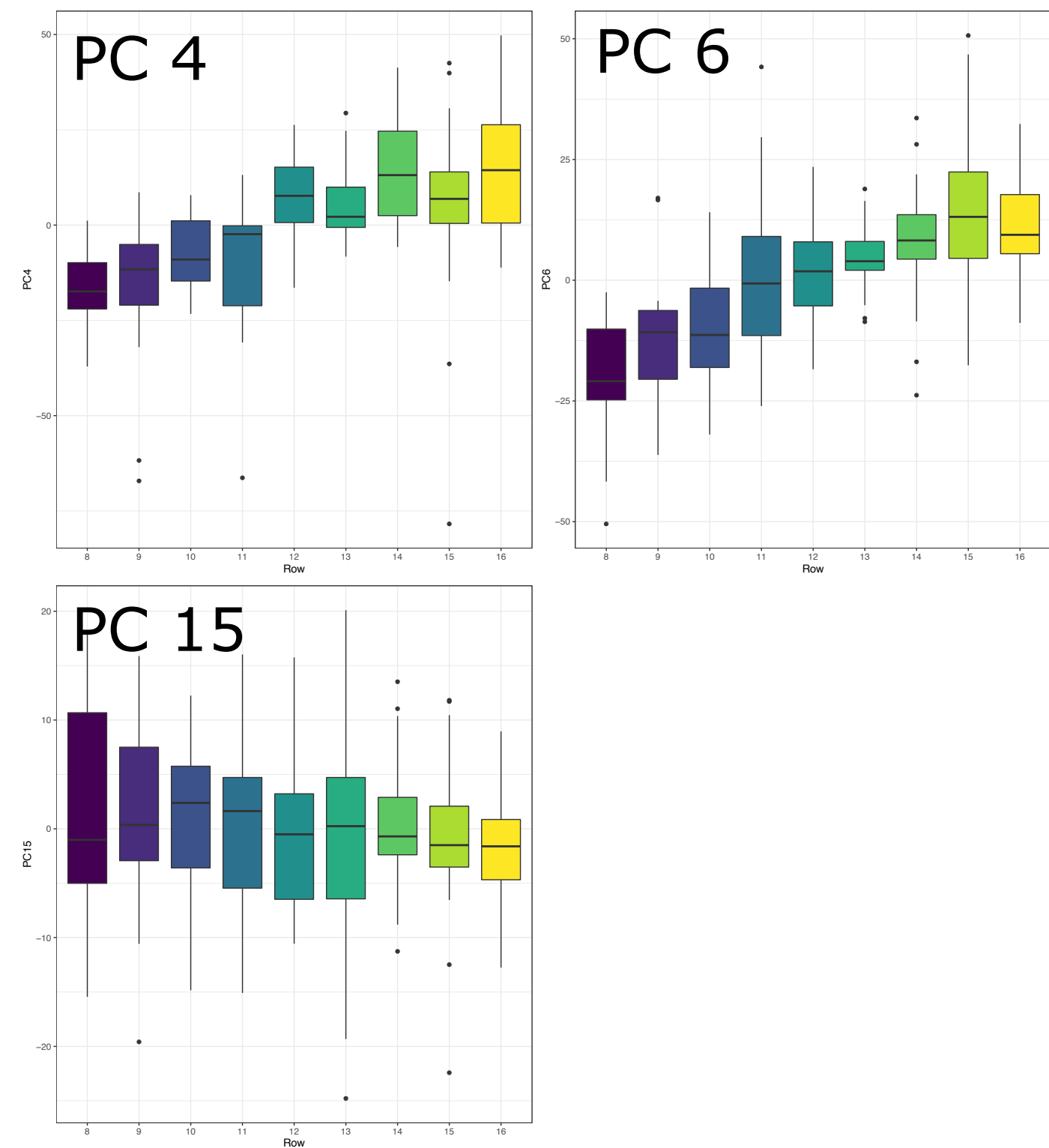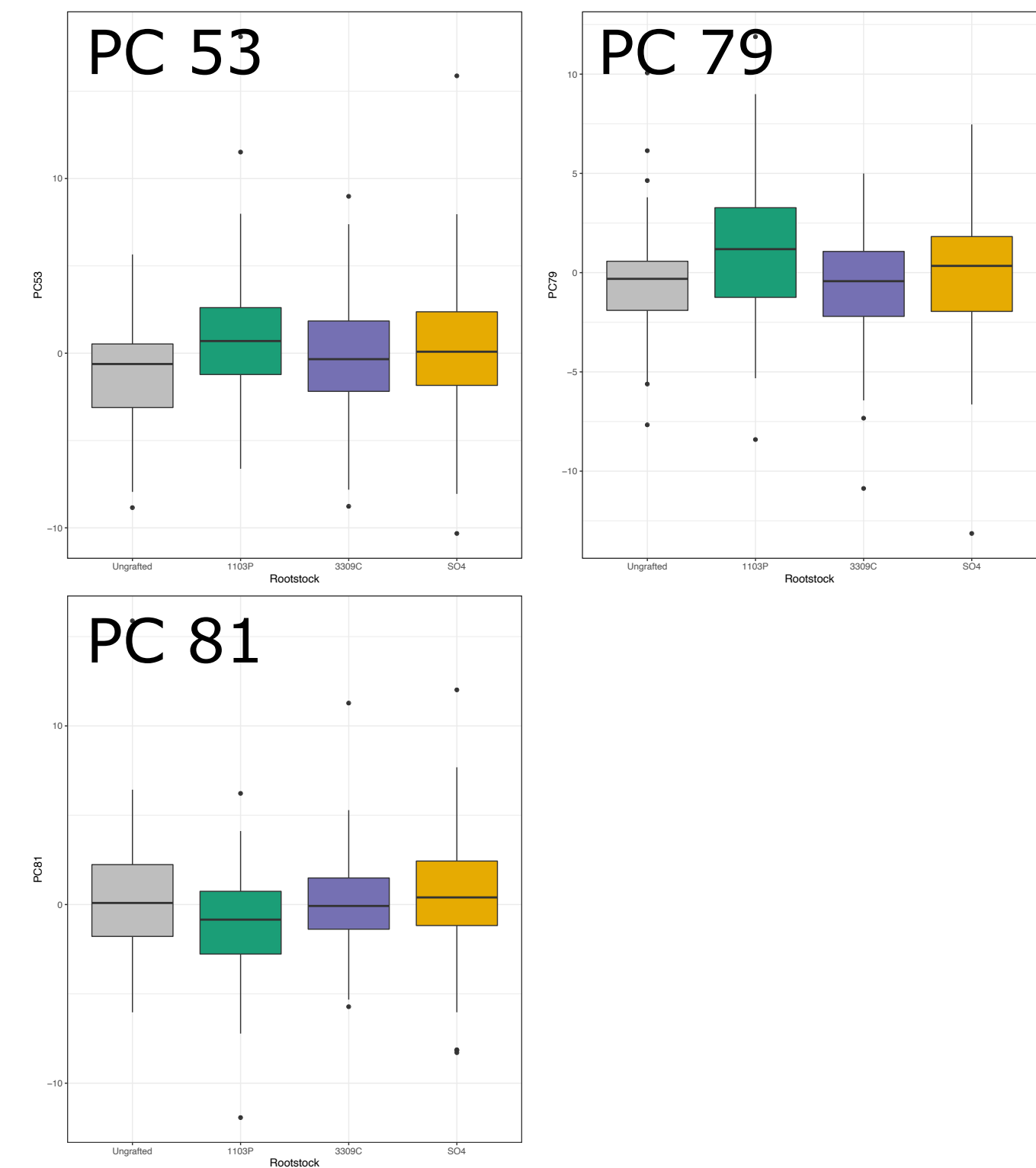

LD2

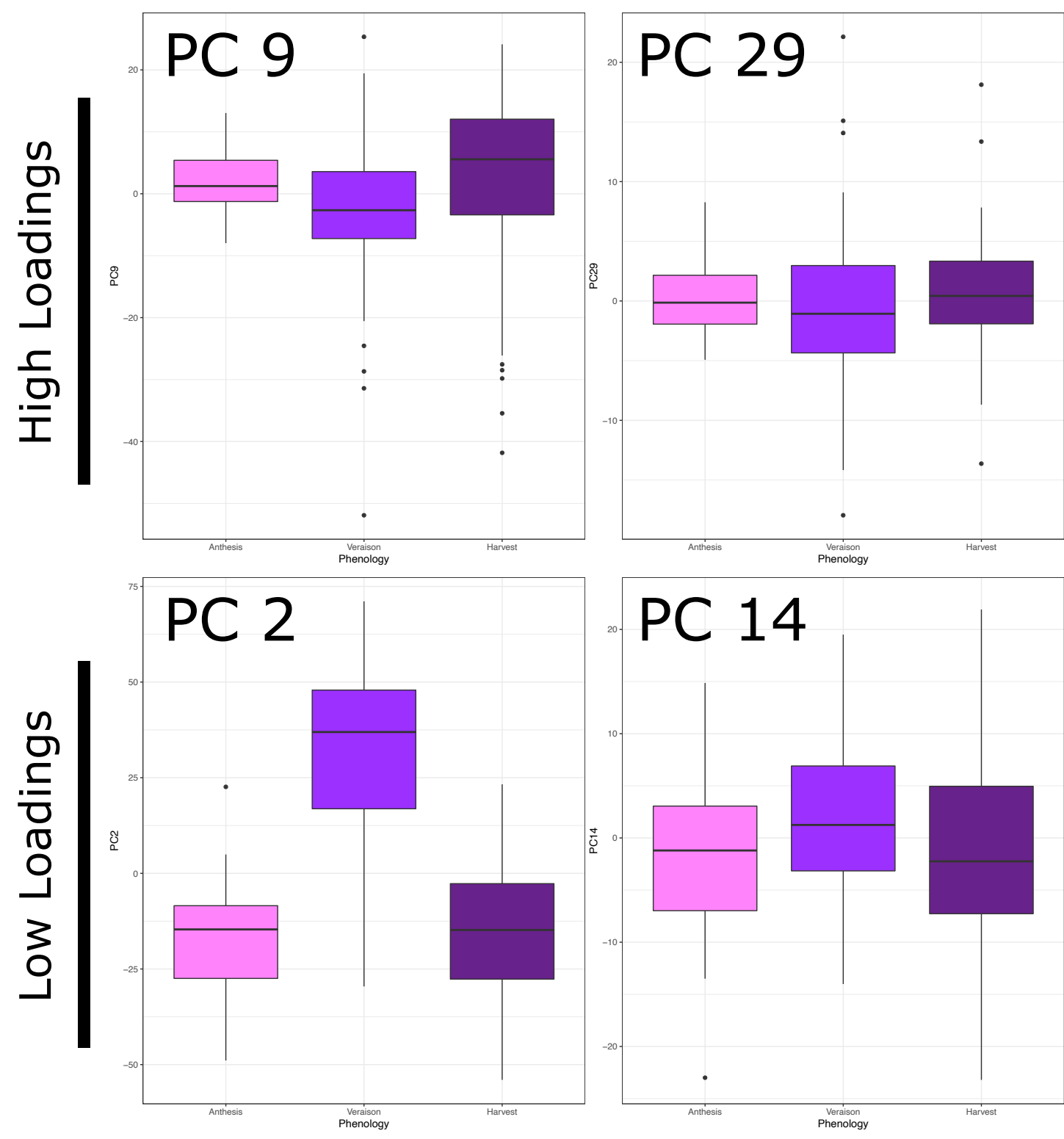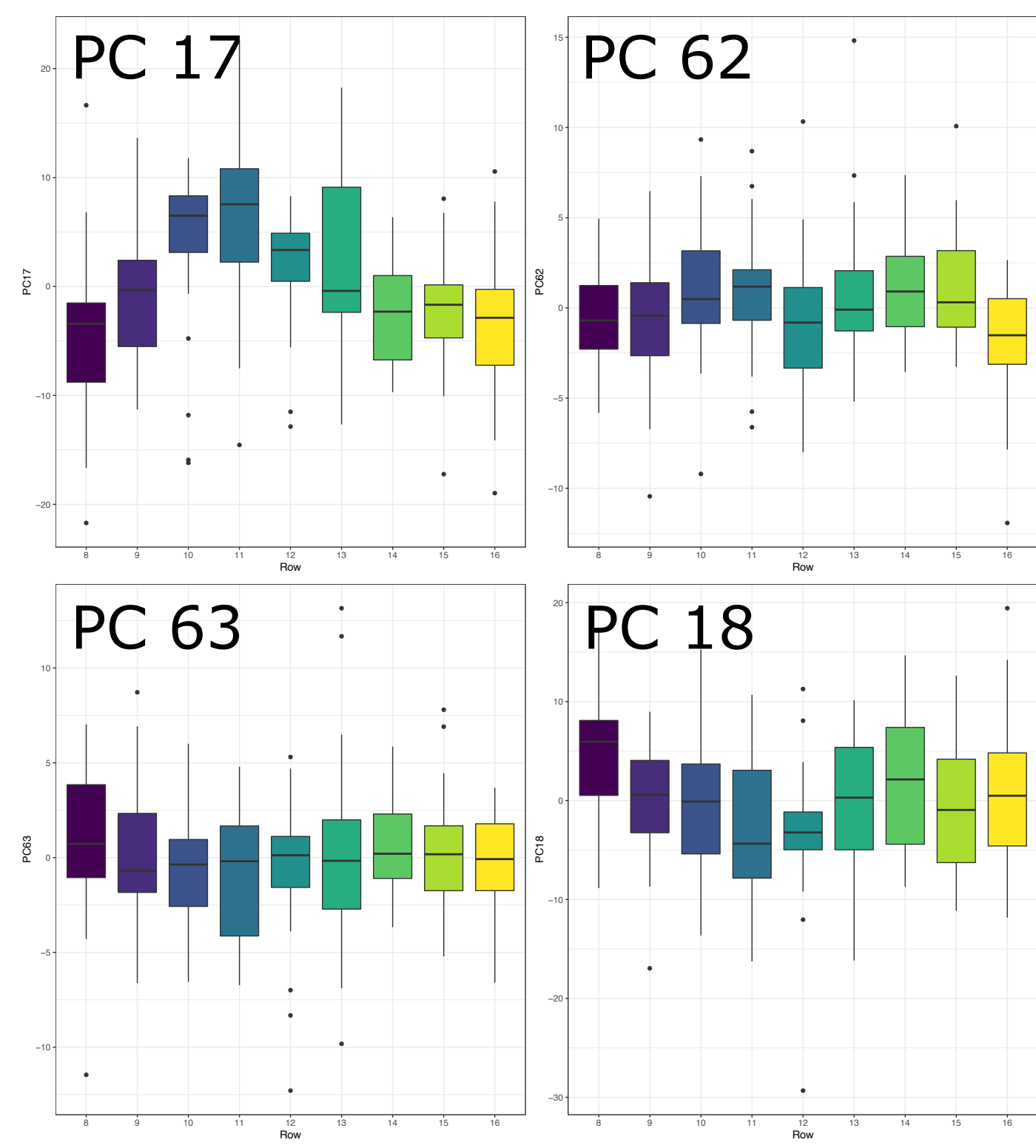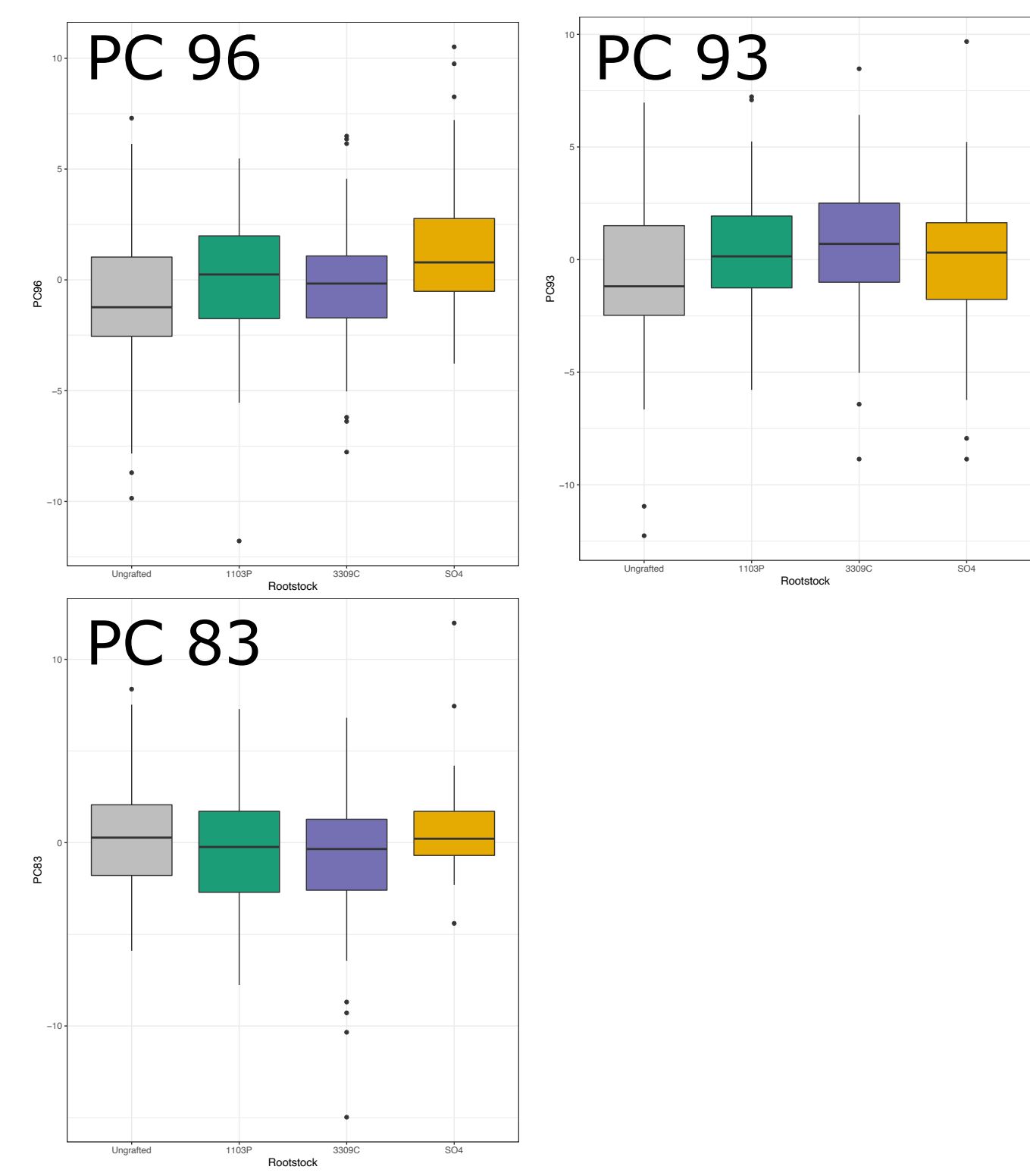

Supplement: giab087_Supplemental_Files [file giab087_supplemental_files.zip › Supplemental Figure 4.pdf]

# A

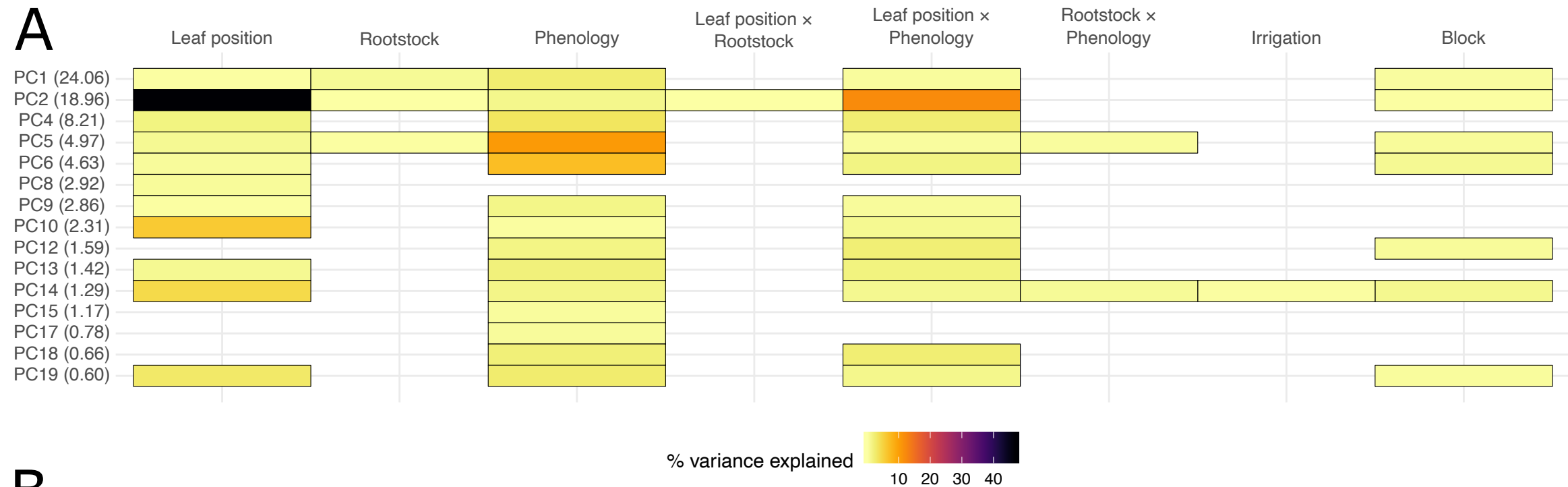

# B

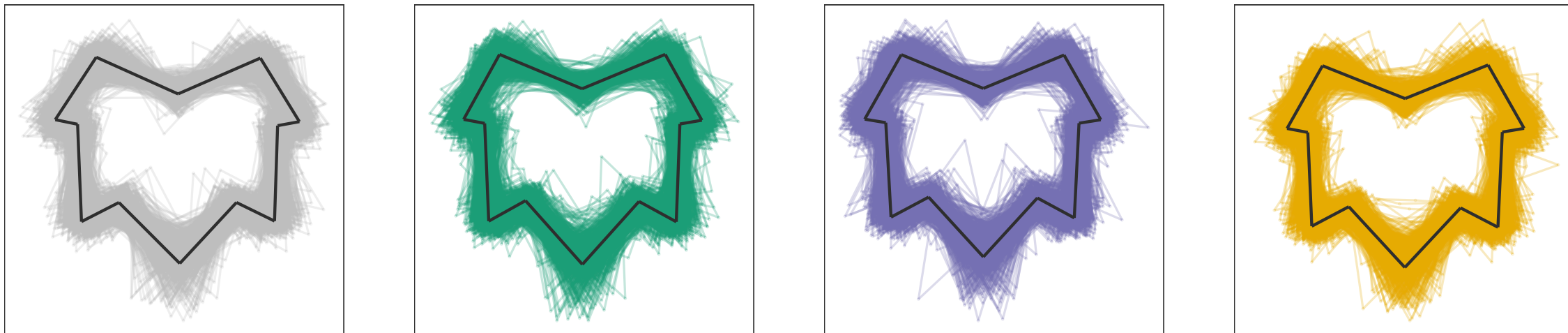

Supplement: giab087_Supplemental_Files [file giab087_supplemental_files.zip › Supplemental Figure 5.pdf]

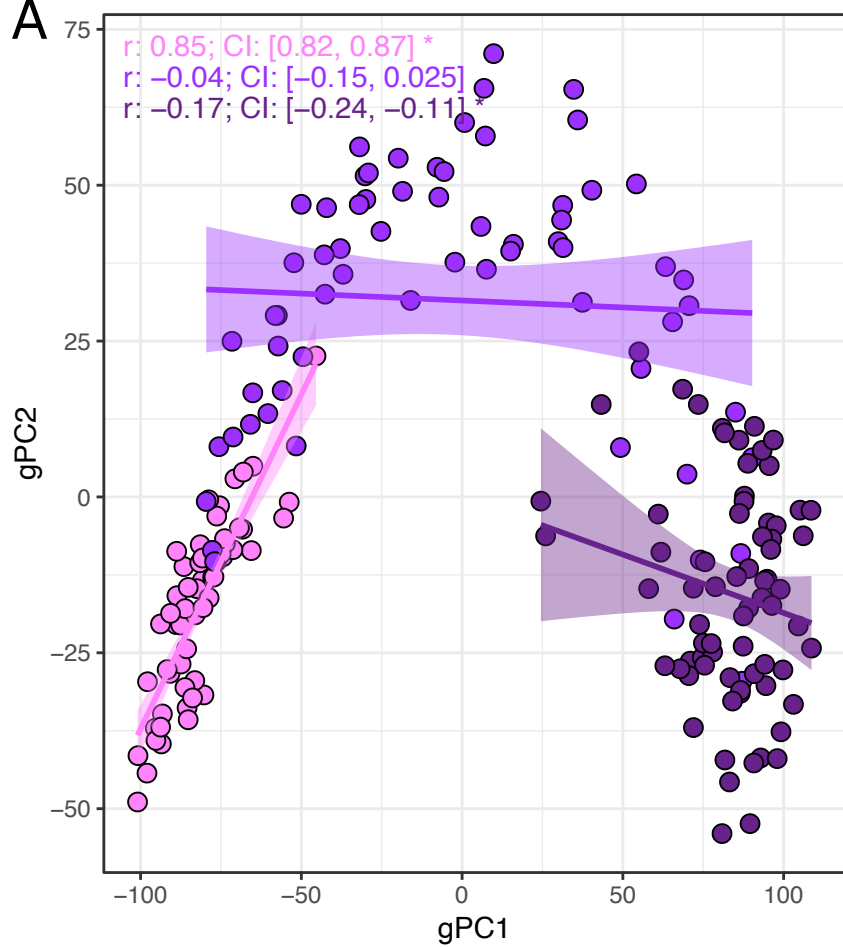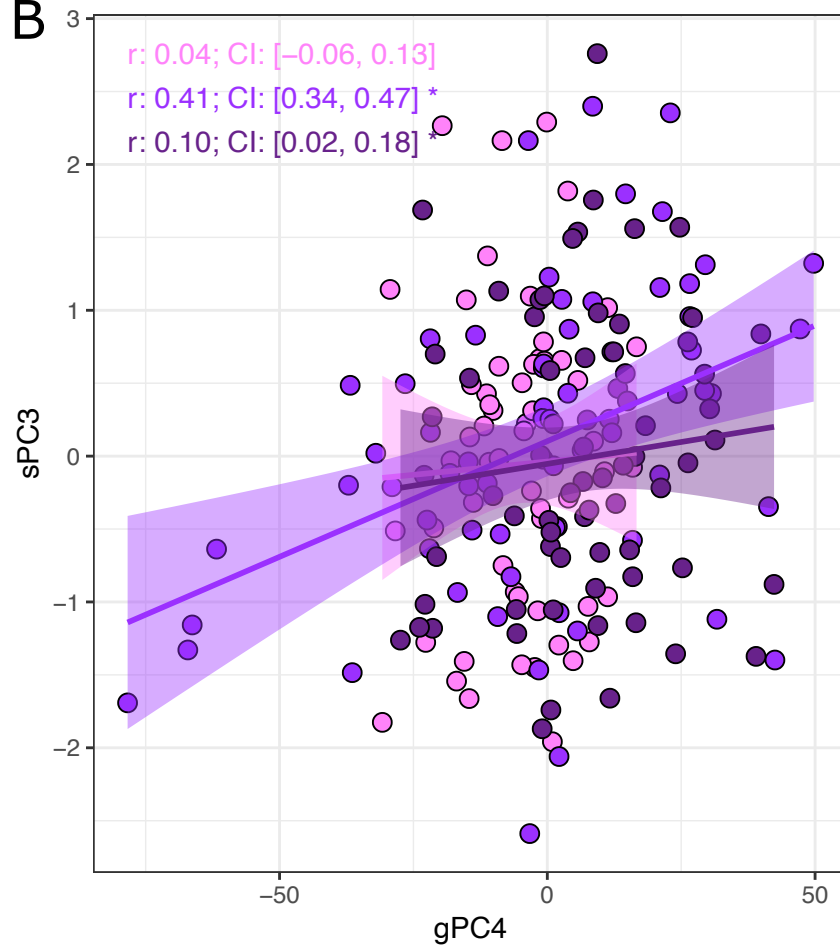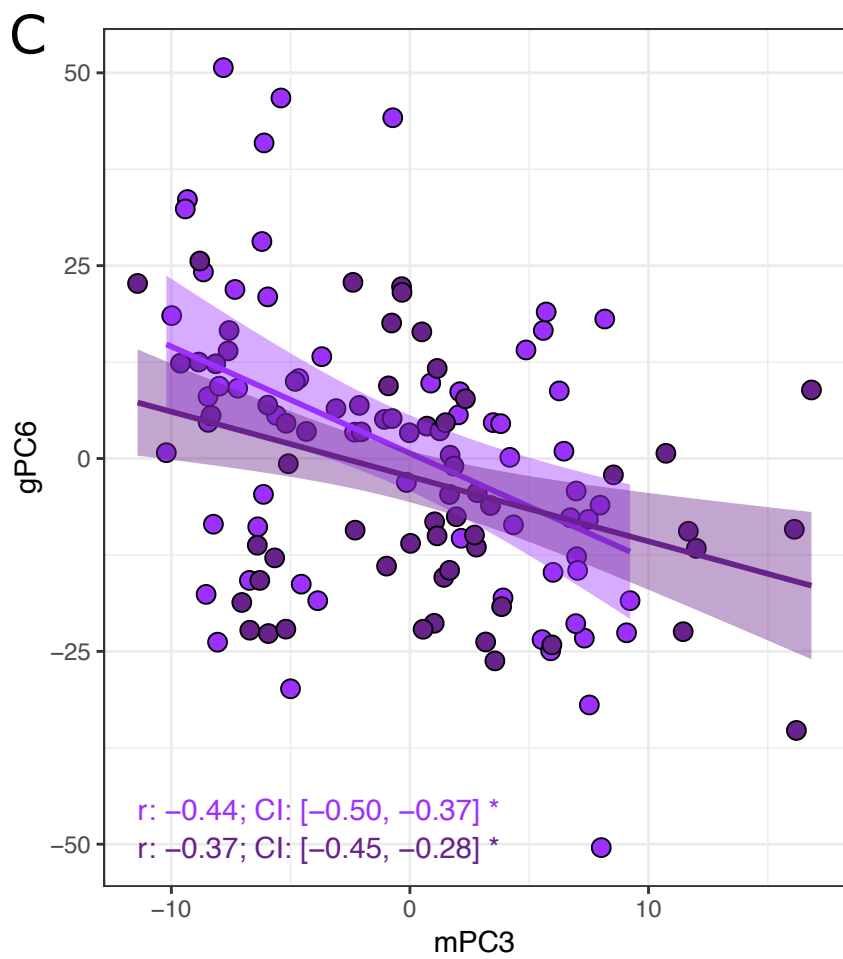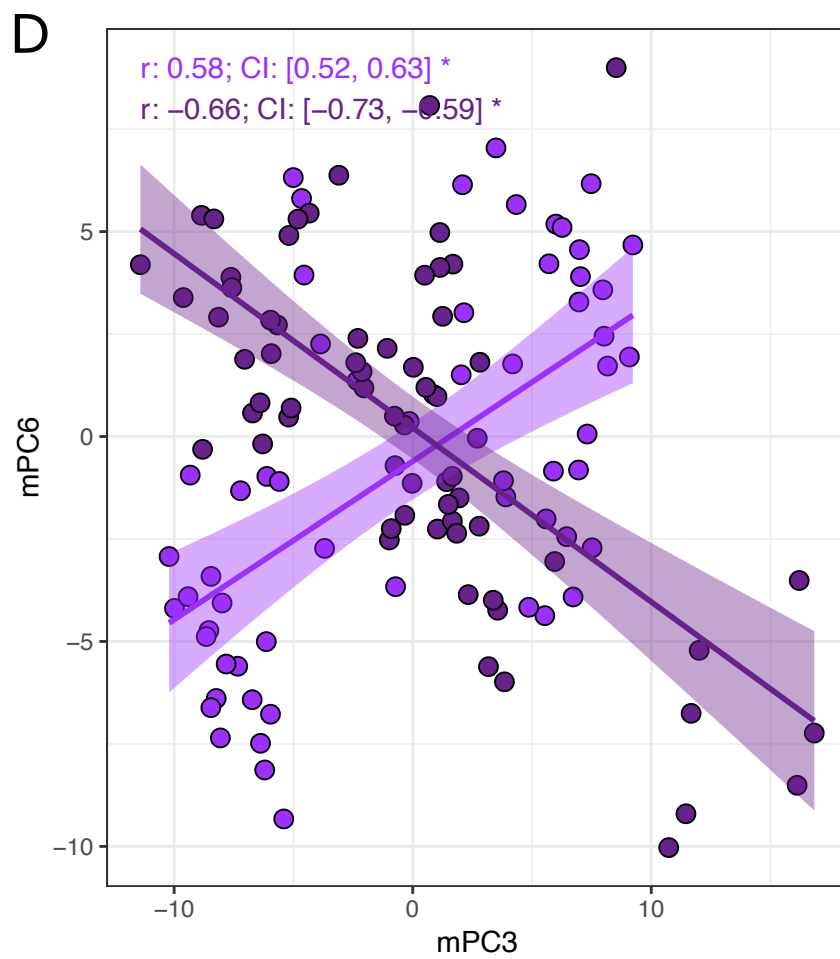

Phenology

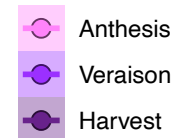

Supplement: giab087_Supplemental_Files [file giab087_supplemental_files.zip › Supplemental Figure 6.pdf]

A

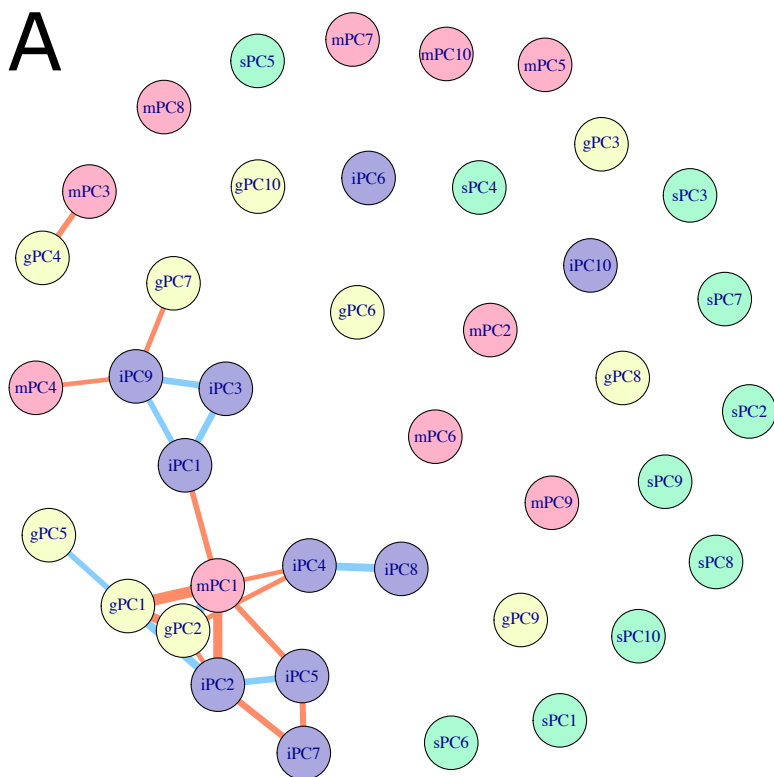

B

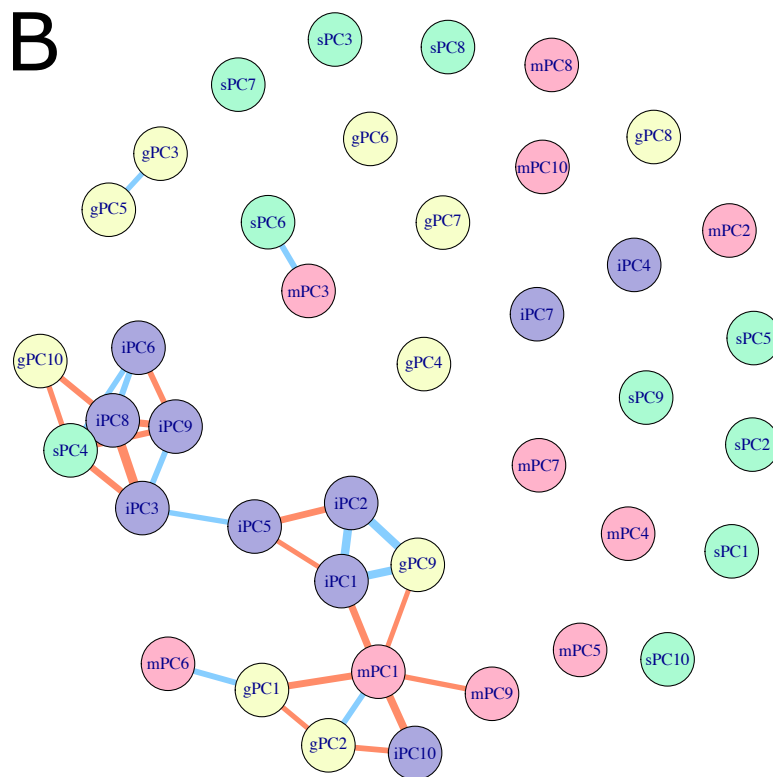

C

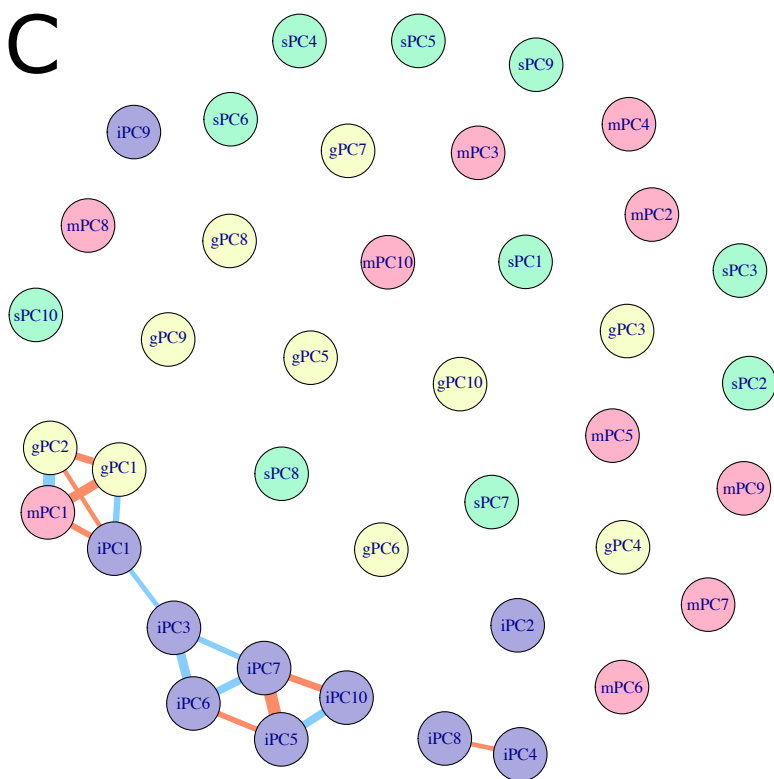

D

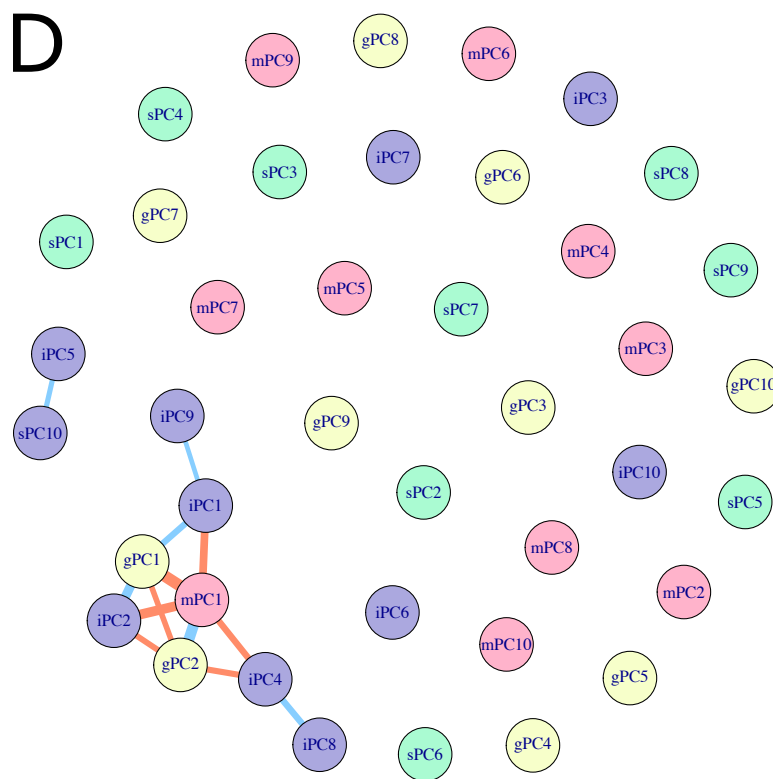

- Ionomics (i)
- Metabolomics (m)
- Gene Expression (g)
- Morphometrics (s)

Supplement: giab087_Supplemental_Files [file giab087_supplemental_files.zip › Supplemental Figure 7.pdf]
