# Supplementary material for: Multi-dimensional leaf phenotypes reflect root system genotype in grafted grapevine over the growing season
Source: Gigascience. 2021 Dec 29;10(12):giab087. doi: 10.1093/gigascience/giab087 (PMC8716362; doi:10.1093/gigascience/giab087)
Supplement: giab087_Supplemental_Files [file Supplemental Figure 2.pdf › Supplemental Figure 2.pdf]

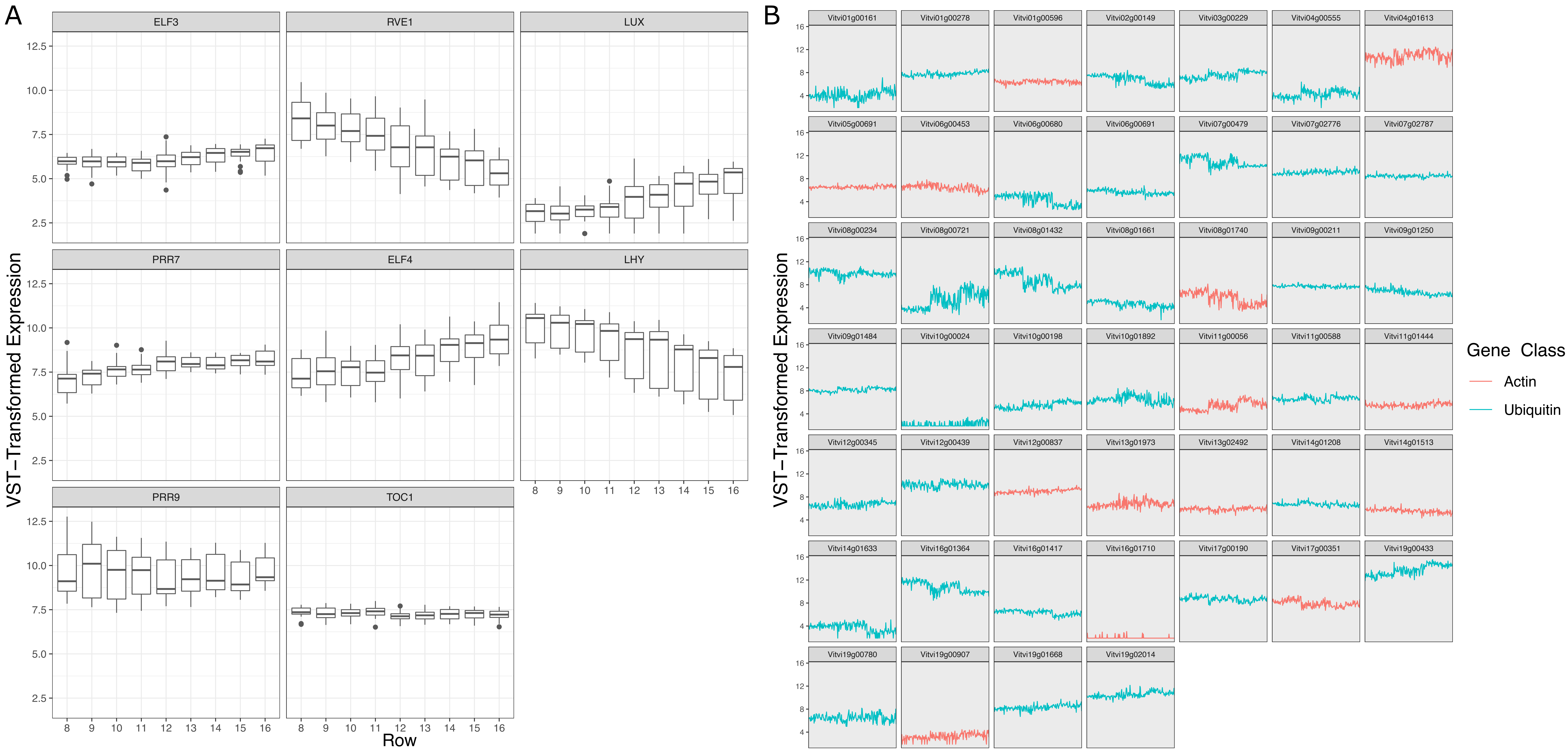

A
ELF3
RVE1
LUX
PRR7
ELF4
LHY
PRR9
TOC1
12.5
10.0
7.5
5.0
2.5
8 9 10 11 12 13 14 15 16
VST−Transformed Expression
Row
B
Vitvi01g00161
Vitvi01g00278
Vitvi01g00596
Vitvi02g00149
Vitvi03g00229
Vitvi04g00555
Vitvi04g01613
Vitvi05g00691
Vitvi06g00453
Vitvi06g00680
Vitvi06g00691
Vitvi07g00479
Vitvi07g02776
Vitvi07g02787
Vitvi08g00234
Vitvi08g00721
Vitvi08g01432
Vitvi08g01661
Vitvi08g01740
Vitvi09g00211
Vitvi09g01250
Vitvi09g01484
Vitvi10g00024
Vitvi10g00198
Vitvi10g01892
Vitvi11g00056
Vitvi11g00588
Vitvi11g01444
Vitvi12g00345
Vitvi12g00439
Vitvi12g00837
Vitvi13g01973
Vitvi13g02492
Vitvi14g01208
Vitvi14g01513
Vitvi14g01633
Vitvi16g01364
Vitvi16g01417
Vitvi16g01710
Vitvi17g00190
Vitvi17g00351
Vitvi19g00433
Vitvi19g00780
Vitvi19g00907
Vitvi19g01668
Vitvi19g02014
16
12
8
4
VST−Transformed Expression
Gene Class
Actin
Ubiquitin
