# Supplementary material for: Multi-dimensional leaf phenotypes reflect root system genotype in grafted grapevine over the growing season
Source: Gigascience. 2021 Dec 29;10(12):giab087. doi: 10.1093/gigascience/giab087 (PMC8716362; doi:10.1093/gigascience/giab087)
Supplement: giab087_Response_to_Reviewer_Comments_Original_Submission [file giab087_response_to_reviewer_comments_original_submission.pdf]

Author's Response To Reviewer Comments

Close

Editor Comments:
Overall, the reviewers have highlighted that in its current form, the manuscript requires more biological validation and more detailed methods to ensure reproducibility of the work presented. Limitations on the study should be discussed and how they may impact the results; and more data should be provided to understand the reliability of the RNA-seq experiment. Furthermore, RNA-seq methods are incomplete - we strongly encourage all authors to add their detailed methods to protocols.io (if not already open in protocols.io and cite the protocol DOI in the paper.
- Response: Thank you for this. We have added all details that have been requested on L191-195. In addition, we added a supplemental figure (Supplemental Figure 2) showcasing our analysis for gene expression validity. A summary of this analysis is on L207-210.

We also see reviewer #4 suggests to use Figshare - but this is not an appropriate database to share large-scale data, such as this work presents. Github is still the most appropriate place to share scripts and associated documentation, and our open repository, GigaDB can host the other metadata not already open in other community approved repositories; we will also host snapshots of your scripts in GitHub.
- Response: In addressing Reviewer #4's specific recommendation, we have moved the document in question from GitHub to a Supplementary Note in the manuscript. In line with previous communications, we are also in the curation process of metabolomics data uploaded to Metabolights. We are happy to jointly upload other data sets to preferred repositories and databases, we just need guidance on the preferred locations for those data. As of today, we are unaware of standard databases for ionomics and leaf shape data. Scripts used for analysis are still available on GitHub, but can be hosted elsewhere if this is of concern.

Reviewer #1

The manuscript by Harris and co-workers presents a characterization of rootstock genotype effects on multilevel leaf phenotypes of one grafted grapevine scion cultivar. Three rootstock genotypes along with the ungrafted cultivar were compared for ionomics, transcriptomics, metabolomics, leaf morphology and physiology in three phenological stages. Analytical and statistical analyses applied were generally sound. While authors identify larger effects in most cases for stage and vineyard position/sampling time, ionic composition was the phenotype most significantly affected by rootstock genotype. Co-variance among multilevel phenotypes is also presented.

Major issues
1. The study comprises a vast dataset, with a total of 288 plants independently analyzed for two phenotypes (morphometry and ionomics) and 72 plants were used for the rest of phenotypes. Nevertheless, the experiment is limited in terms of genotypes tested and reproducibility. Only one year of study and under the specific soil and climate conditions of a single field plot. Moreover, the effects were only tested on a single scion genotype, a bred interspecific hybrid including Vitis riparia and V. rupestris in its pedigree. At least one of these species is also in the pedigree of the three rootstocks tested, which might involve lower diversity than in common interactions between rootstocks and V. vinifera cultivars. These limitations should at least be considered when discussing the results.
- Response: The comments provided by the reviewer are all excellent observations that were clearly missing from the discussion of our work. We have added a paragraph to the Discussion (L544 - L559) to better couch how our work should be compared to other studies and the considerations that may account for those differences.

2. The RNA-seq assay did not identify differentially expressed genes (DEGs) in response to rootstock genotype, which disagrees with previous reports. While the origin of the lack of effects here is unclear, further data should be provided to understand the reliability of the RNA-seq experiment:
- Response: We thank the reviewer for this comment. We were similarly interested to see that our results differ from previously published analyses in similar systems. We added comments to the

discussion to clarify why we think these differences might be present. To ensure such reliability, we confirmed in our data that previously established patterns of house-keeping and circadian phased genes were behaving as expected. The following has been added to the manuscript:
- Added to data description L208-210, “To check the validity of our expression results, we assayed two classes of housekeeping gene (Ubiquitin-domain and actin-family) and eight previously annotated circadian genes (Carbonell-Bejerano et al. 2014)”
- Added to Analyses, L339 - 345, “We computed the expression of two classes of housekeeping genes, and showed that they are generally stable across samples over phenological time (Supplemental Figure 2). We noted that some variation is expected for housekeeping genes; see, for example, [49]. Moreover, we showed that patterns of previously annotated circadian genes conform to expected results over the sampling window. For example, predicted orthologs of LHY and RVE1 are correlated and decreasing over our sampling window, and a predicted TOC1 ortholog is invariant. The results of these analyses provide general confidence in the gene expression data presented here.‟
- Created and added Supplemental Figure (now supplemental Figure 2) showing these patterns.

2.1. Which was the timing of sample collection for RNA-seq samples? Was the same sampling order followed for the three phenological stages? Which were the weather conditions on each of the three sampling dates? It is relevant to describe that information since environmental and circadian changes between and within days can alter gene expression.
- Response: We added the following to the section describing the gene expression data set (L187-190): “Leaves were sampled by a single team near midday between 10AM and 2PM in row order ensuring that ‘block’ and ‘row’ accounted for unmeasured environmental variation and temporal variation over the sampling window.” A statement was added on L637-638 that “At each phenological stage, effort was made to sample on days with full to partial sun and minimal precipitation.‟

2.2. Data on RNA sequencing depth should be provided to understand the resolution of the transcriptomics experiment. For instance, how many bases/reads per sample were produced? How many genes per sample were called as expressed (DESeq2-norm counts >2 according to authors own threshold)?
- Response: Information on sequencing depth and genes per sample were added to the Analyses section. Specifically, we added the following to L337-338: “On average, each sample contained 4.1 million 3’-reads and showed the expression of 17,852 genes.”

3. The interpretation of the origin of the results is generally shallow and several questions or limitations are overlooked. For instance:
3.1. It is described that physiological parameters were measured from 10 am to 1 pm, a wide interval with expected changes in environmental conditions affecting these measurements. To understand for possible covariances, it should be indicated if these measurements were carried out simultaneously and following the same order than that of leaf sample collection for the other phenotyping.
- Response: We agree that we missed crucial details about the timing of this sampling. To fix this, we included the clarifications that (on L241) all physiology measurements were being taken simultaneously by different groups moving though the vineyard and (on L243-245) the measurements were all taken in row order ensuring that the vineyard blocking factor captured temporal variation. SImilar notes were added for the other phenotypes to better explain sampling. As was noted in the next reviewer comment, block is missing from Figure 5 which means it was not significant as a main effect.

3.2. Related to the previous, why block effect alone was not considered for physiological measurements in Figure 5?
- Response: Thank you for this comment. Block (or row for gene expression) was included in the models as a main effect for every modality (see analysis section). Non-significant factors were dropped from figures summarizing linear models; because the block main effect was not significant, it was not included in Figure 5. .

3.3. Did the horseshoe shape for row effect on the transcriptome correlate with oscillation of environmental/circadian clock conditions during the sampling interval or with vineyard heterogeneity? Functional analysis of the genes contributing to row effect could be informative on the origin of these effects that might have hindered the identification of rootstock effect on the transcriptome.
- Response: This is a really interesting comment. We agree with the reviewer that the horseshoe shape in LDA space is either a function of circadian conditions or spatial heterogeneity. We have added to the Data Description section a comment on assaying genes with known circadian topology (L207-210) and show in Supplemental Figure 2 that those genes are variable over our sampling window. In addition, we commented on this outcome in the Analyses section on L360-362. We show that the impact of vineyard

position/spatial variation is weak in other measured phenotypes (captured by the ‘block’ model term; see, for example, Figure 1A and Supplemental Figure 5A). Future studies should assess potential intra-vineyard variation either through blocking (as we did here) or explicit measurement (for example, soil composition) and control for that variation.

3.4. Is there a rootstock effect on vigor, biomass, fruit fertility and production that could explain or condition the effects in leaf phenotypes that were measured? Were these factors normalized in any way, either by agronomic practices or statistical treatment?
- Response: This is an excellent question that is perhaps beyond the scope of this comprehensive analysis of leaf phenotypes, but one that is certainly an important next step in our research trajectory. Conditioning or normalizing on aspects of vigor or yield or looking for correlates of those traits in early season leaf phenotypes would be immensely valuable to viticulture and a general understanding of grapevine biology. To explore this idea, we have amended the language of the Potential Implications section on L588-608. In addition, some of these data were collected and are being prepared for papers focused toward berry phenotypes. In the meantime, we point to (https://doi.org/10.1002/pld3.324) to show that this is absolutely a valid direction of inquiry for future work and data integration efforts.

4. This study comprises similar experiments to these already published by the same group in the same set of plants (Migicovsky et al., Hort Res 2019), although extended to include metabolomics and physiology data and two additional phenologcal stages. While the effect of phenology is clearly presented here, the addition of the metabolite data is undermined. What are the metabolites determining rootstock effect in Figure 2C? What about metabolites determining a rootstock effect depending on phenology that could be inferred from PC10?
- Response: We thank the reviewer for this comment and appreciate the careful consideration of this manuscript in the context of the Migicovsky et al, 2019 manuscript. We completely agree that the value of the metabolomics data is undermined in the manuscript. This is primarily the result of current challenges in mapping peaks from LC-MS onto named metabolites. The current state of untargeted metabolomics from LC-MS would require significant chemical laboratory work to narrow down the space of potential metabolites. While we believe this work should absolutely be done, our goal with this study was not necessarily to identify specific metabolites but to determine if the metabolome was a potential avenue through which the rootstock is influencing scion phenotypes. To address this, we used only a portion of the runs available to show there is a signal. Future work will focus on merging the various additional LC-MS runs (not presented here) and chemical experimentation to uncover the full scope of this effect. We note that we are uploaded raw data to Metabolights, QC/filtered data to FigShare, and reported the retention times and m/z ratios for the compounds of putative interest in the manuscript. We hope that these data may be useful in future analyses of grapevine metabolites, either by our group or others.

Minor revisions
1. "Ubiquitous" effects of rootstock genotype are described along the MS. However, since only one location was analyzed (leaves), would "pleotropic" be more appropriate to define the different phenotypes affected by rootstock-scion genotype interaction in this study?
- Response: This is a great point. We clarified our usage of the word “ubiquitous” to ensure its intended meaning (across modalities within leaves, not as an inherent feature of grapevine) was clear (for example, on L514) throughout the manuscript. Supporting the reviewer’s observation that this study only included one environment, we are hesitant to use the word “pleiotropy”, though we are interested in the implication that the different rootstock/scion pairs create different local environments and will consider this idea in future works.

2. Methods on RNA-seq procedures are incomplete. Which sequencing technology was used? Which type and length of reads? Etc.
- Response: Good catch. We added the following to the L194-195: “Sequencing was conducted using the Illumina NextSeq500 platform which returned single-end 86 bp reads.”

3. Inter-annual comparison for anthesis ionomics, transcriptomics and morphology between this study and their previous publication (Migicovsky et al., Hort Res 2019) could enable a broader interpretation of rootstock effects, overcoming the reproducibility limitation of considering only a single season here.
- Response: We absolutely agree that interannual analyses are required for a detailed understanding of the root system influence on shoot phenotypes and these analyses are underway. Our goal with this manuscript was to carefully quantify different phenotyping modalities and to understand how they relate to one another. The results from this study have helped us consider what is worth more detailed

investigations, and analyses that address longer (multi-year) studies for those phenotypes are currently in the works. Given the magnitude of the data presented here and the extent of analyses conducted, we struggled to fit this detailed work in a single manuscript that also covered inter-annual variation as well as additional phenotypes (berry chemistry, etc.). As a result of work presented here, we are currently exploring tradeoffs between deep analyses of individual phenotypes and shallower analyses of more modalities over longer time periods, additional scions, and multiple sites. In the meantime, wherever possible we note some comparisons to the Migicovsky 2019 study where appropriate. The Migicovsky 2019 pilot study used considerably different methods for many phenotypes, which preclude direct comparisons.

4. L426. The sentence might not be completely fair as no DEG was identified for rootstock effect (transcriptome phenotype would therefore be mostly unaffected) and developmental stage-specific could be more adequate than season-specific.
- Response: Thank you for this. We agree with the suggested change in language for the effect of phenology and changed “season specific” to “specific to the vine’s developmental stage” on L468. On gene expression, while no DEGs were identified, we were able to identify latent combinations of genes that were responsive to rootstock treatment. While this effect is subtle, it was nonetheless detectable.

5. Any biological interpretation of the specific metabolites, genes, iones, shapes determining the resulting PC covariation networks? While it can be interesting to add to covariation networks additional levels of phenomics as authors propose (lcRNA, micorobiome, epigenetics), it would also be informative to exploit the interpretation of the dataset that they have already produced.
- Response: Excellent suggestion. Unfortunately, after much consideration, at this point we do not feel comfortable with detailed biological interpretations based on specific metabolites or genes that underlie PC covariation networks shown here. Some of the limitations of our dataset, and why we are unable to make these mechanistic connections with data presented here, are detailed in the discussion. We note that the ionome offers a very rich source of data ripe for deep analysis, and that an additional manuscript describing a deep dive into multi-year, multi-time point ionomic dataset is in preparation now. We agree that future work should be targeted toward biological understanding of these relationships. On suggesting inclusion of other phenotypes, this comment reflects our enthusiasm for other existing approaches and exciting areas of research that might further uncover mechanistic understanding of the effects we are seeing from grafting and over time. The analysis presented in the paper, unfortunately, does little to advance us toward the goal of mechanistic understanding, but it does help us see where future studies could be targeted. To this end, we added language to clarify this point on L573-578.

6. L470, If the lack of rootstock effect on the transcriptome was due to the phenology effect, specific analysis at each phenology stage would identify rootstock genotype factor significant DEGs. Is it the case? Would there be any rootstock effect detected on transcriptome if the analysis was restricted to single blocks at specific phenological stages?
- Response: We thank the reviewer for this comment. This is certainly something we are trying to wade through as our results suggest that the rootstock influence on our vines is incredibly complex and works through interaction with other factors of the experimental design. Ongoing work is focused on identifying these complex effects, in a statistically robust way. We are also currently working with collaborators to identify genes and gene regions worth further exploration. Moreover, we are seeking to use results from other phenotypes to focus on genes in a more ‘hypothesis-driven’ approach that can further the ‘discovery-driven’ results observed here.

7. Apart from the seasonal effect, the "Potential implications" presented are not directly inferred from the Results obtained here but from the potential of the approach used. Any other potential implication of the specific results?
- Response: This is a very helpful suggestion. In response to this and comments from other reviewers we have re-worked the potential implications section. Other reviewers called for an enhanced focus on yield/viticultural implications, while others have asked us to minimize such speculation. Consequently, we have attempted to carefully place this work in the context of both basic plant biology and viticulture. If the current revision does not meet the expectations of the reviewer(s) or editors we would be happy to revise further.

8. Is there any data available for the distribution of soil properties across the experimental plot that could be considered to discuss the origin of block effects? Could the human factor during that extensive sampling be another variable accounting for block effect?

- Response: Thank you for this excellent observation. For the data presented here, we do not have paired soil samples. We anticipate some heterogeneity in soil properties across the experimental plot; however it is unclear how strongly this would correspond directly to block effect. Regarding the human factor, we have added a sentence into each data modality clarifying what variation is captured by the blocking factor. See each addition below:
- L136-138 added, "Teams were deployed in the vineyard so that multiple vineyard rows were being sampled concurrently. As such, 'block' represented unmeasured spatial variation, but did not strictly correlate with time of sampling due to the nature of sampling (see Methods)."
- L155 - 157 added, "ensuring that 'block' captured both unmeasured environmental variation and temporal variation over the sampling window".
- L187-190 added, "Leaves were sampled by a single team near midday between 10AM and 2PM in row order ensuring that 'block' and 'row' accounted for unmeasured spatial variation and temporal variation over the sampling window (see Methods)"
- Overall, block is not a large descriptor of variation in our study except for the phenotypes for which block is collinear with time of day. In these phenotypes (the metabolome and the transcriptome) there is a noted circadian topology. The other phenotypes (ionomics, leaf shape, and physiology) see little effect from block suggesting there is little spatial variation (or at least that the spatial variation is unimportant for those phenotypes).

9. Because half of 3309C reps would have been collected before any ungrafted rep was taken, could the LD2 effect in discriminating 3309C and ungrafted from RNA-seq data be related with sampling times? What are the genes involved in this effect?
- Response: We thank the reviewer for this comment. While it is always possible that results correlate with unmeasured confounders, rootstock genotype was not confounded with any of the terms in our model (including time of sampling, which was a correlate for row in our study; L653-655). Each rootstock was present in each row of the vineyard in cells of four replicated vines (See supplemental Figure 1A). For the transcriptome sample, we sampled leaves from the middle two vines in each cell. While it certainly takes time to sample in a vineyard, each rootstock in each row would have been sampled within minutes of one another. Due to this, the grouping we report in LD space is not confounded with time. The current results suggest that this effect is driven by complex combinations of genes (from the PCA results) and not any particular genes being strongly affected (from the traditional DGE results). We are currently working on exploring these subtle effects in more statistically robust ways in a multi-year study.

10. Any discussion on the origin of leaf position effects in specific ions?
- Response: Thank you for this comment, and this is something we think about often. Leaf position was added to this study on the ionome because it is known that leaves vary in their elemental composition over development. The major question here was whether or not rootstock would influence the elemental composition of those leaves in such a way that the known patterns might be interrupted. While we observed significant variation in ion concentrations as a function of leaf position, it did not strongly interact with rootstock genotype. In other words, the rootstock effect was present in all leaves, not just leaves of a particular age. As such, we struggled to fit a detailed description of the effect of leaf position in the current paper. However, we have made all data from this analysis publicly available if there exists specific interest for the leaf position. Ongoing work focused explicitly on the leaf ionome will provide a deep dive on how ion concentrations vary by rootstock, over development, across seasons, and across multiple years.

11. L556. Indicating in there that "only the middle two vines of the four cells in the front half of the vineyard were included in the 72-vine set" would be handy to understand the distribution of this set.
- Response: Thank you for this comment. We have clarified the description of the experimental vine throughout the manuscript. The experimental design of the vineyard included groups of four identical vines (e.g., Chambourcin grafted to 3309C) that are distributed in a randomized block experimental design throughout the vineyard. For some data modalities we were unable to process samples from all four vines per cell. In these circumstances, we opted to collect samples from the middle two vines of the four vine set. We collected from the middle two vines from a total of 36 cells for the 72 vine set. To improve the understanding of this section, we have amended the text to point to more appropriate sections of Supplemental Figure 1. In addition, we have improved the Figure Legend for this figure so it is more clear what each panel is showing with explicit descriptions for Supplemental Figure 1B, which should improve the clarity of this section. Finally, we clarified that this description only applied to the front half of the vineyard which was missing from the previous draft. We would be happy to make

additional edits to the text if this description does not provide sufficient clarification.

Reviewer #2
The manuscript by Harris et al investigates the effect of grafting on a number of physiological and molecular phenotypes within grapevine (Vitis spp.) scions. The hybrid Vitis cultivar Chambourcin was compared when grown on its own-roots, or when grafted to three different commercial hybrid rootstocks: 1103P, 3309C, and SO4. The vines were grown in the field, irrigated with different volumes of water, and sampled over a single growing season. Large data sets have been generated for leaf metabolites, solutes (ions), transcripts, shape, and physiology (stomatal conductance, transpiration). As such, the manuscript fits the scope of Gigascience well. The manuscript is well written, however I found it was very statistical and would benefit from additional biological analyses to confirm and validate the findings. The methods section is lacking some details that would enable reproducibility. Some of the figures could be improved for readability. My comments and suggestions are detailed below:

Major comments
1. There is no information on the age of the vines at the time of the experiments.
- Response: This is a great observation of information we overlooked. The vineyard had been in the ground for eight years at the time of sampling. The age of the vineyard has been added to L538 and commented on in the discussion.

2. A quantitative analysis of the elemental content of the irrigation water by ICP-MS would be beneficial. In this study, it is unknown whether the irrigation treatments contained varying levels of the elements that were measured in leaves. To this end, it is perhaps not surprising that rootstocks had minimal effect on, for example, the Na^+ content of grafted scions. However, it has been demonstrated previously that own-rooted vines cannot efficiently exclude Na^+ compared to grafted vines when irrigated with 100 mM NaCl (see Fisarakis el al (2001) Agricultural Water Management 51 13-27).
- Response: This is a really intriguing suggestion, and one we wish we would have thought of in 2017. Regrettably, it was not something that was considered for this study at the time. However, we can be confident that irrigation was not significantly altering the findings of our study due to the weak nature of the irrigation effect (See Supplemental Note 1). Had there been variation in the ionome of the irrigation water, we would have expected to see a stronger irrigation or irrigation by phenology effect, neither of which were strongly observed in this study. We thank the reviewer for pointing us to this helpful article.

3. The manuscript would be more useful to the plant science community if a subset of the actual metabolites and genes identified within the principle components were named and confirmed using a second method. It would then be possible to discuss which physiological, metabolic, and molecular processes within Vitis scions are impacted by rootstock selection.
- Response: We absolutely agree gene-level and metabolite-level understanding of the root system influence on shoot system phenotypes is the direction this work needs to head. This is perhaps one of the biggest limitations of large-scale analyses of multi-dimensional phenotypes: it is sometimes hard to narrow in on individual phenotypes for some systems. We acknowledge that there is a trade-off between large-scale analyses like the one presented here and identification of actual metabolites/genes and their functional role in the vine. We see these as very complimentary approaches that illuminate different aspects of vine biology; however, we were unable to do both in this study. Ongoing work is attempting to, in a statistically robust way, uncover those subtle effects from even deeper sampling of the transcriptome. The metabolome as described using the untargeted approach here is a whole different monster. We were able to show that some metabolites are responding to the rootstock treatment and even to the rootstock by season interaction. The current nature of LC-MS and untargeted metabolomics in Vitis generally make it incredibly non-trivial to map these metabolites. Here we sought to catalog the basic responses of multiple phenotypes to help guide more targeted analyses and guide us toward studies that could produce mechanistic understanding.

4. Similar to my comment above, some of the data could be integrated. For example, transpiration was increased for scions grafted to 1103P (Fig 5B). Were genes or metabolites involved in the regulation of stomatal aperture differentially abundant when grafted to 1103P?
- Response: This kind of data integration is an excellent suggestion. While such analyses would require work beyond the scope of this paper, we think that this comment is exactly in line with how we should be guiding future work. We proposed in the previous comment that this work was meant as a foundation on which we establish the basic responses of many complex phenotypes over the growing season with respect to the rootstock genotype. The PCA-based integration was to help us narrow down which types

of data modalities warrant future integrative work. As we move toward identifying and annotating individual genes and metabolites, these suggestions will certainly help in that future planning.

5. The ionomics data in Fig 1B and C would be easier to interpret if presented as a percentage - for example, % DW, % FW, or mM of tissue water. Currently, there are no units on the Y-axis.
- We agree that z-scores were not the ideal choice for this figure. To address this, we remade this portion of this figure to show the elements as concentrations in parts per million of acid-digested dried leaves. We have updated the Figure Legend L706 and L710 to reflect this change.

6. There is no mention of how the RNA was extracted from plant tissues. Further, a quality control would normally be performed, e.g. by measuring the 260/280 ratios at the very least. Was any quality control performed on these RNA samples? How do we know the samples were pure and not degraded?
- Response: Excellent point that was also noted by Reviewer 1. The information requested has been added to the Data Description section, L191 - 193: "Total RNA was extracted from plant tissues using the Sigma Spectrum Plant Total RNA kit with modification of the addition of 2% PVP40 to the extraction buffer to decrease phenolic inhibitors. All RNA extractions were checked for quality control using a Nanodrop. Sequencing was conducted using the Illumina NextSeq500 platform which returned single-end 86 bp reads."

7. It is unclear how many biological replicates were used for the RNAseq experiments.
- Response: Good catch- thank you for this. Language changes were made throughout the manuscript (in conjunction with other review comments in the section "Study Design" and within the Data Description for each modality to improve clarity. In short, each modality was sampled from either a 72-vine set (metabolomics, gene expression, physiology) or a 288-vine set (ionomics, leaf shape). In the 72-vine set, we only sampled the middle two vines from each four-vine cell in the vineyard (shown in Supplemental Figure 1B-C). At the highest order interaction for gene expression (rootstock:row:phenology), the number of biological replicates would be two. Since this is clearly underpowered, we put little effort into estimating or interpreting those effects. However, lower order interactions (like rootstock:row or rootstock:phenology) are averaged over the remaining samples. For example, the rootstock:row effect is estimated from 6 samples (averaged over phenology). Similarly, main effects are averaged over all other terms, so the rootstock effect would be estimated from 18 samples.

8. Usually, for genome-wide transcriptional studies, the expression patterns of a subset of genes are confirmed using another method (e.g. quantitative real-time PCR). This has not been performed in this manuscript. Authors need to confirm the validity of the RNA seq dataset.
- Response: Thank you for this comment. We agree that for studies focused on identifying genes associated with specific phenotypes, that the gold standard for genome-wide transcriptional studies is cross-validation using qPCR. The goal of this study is to understand the influence of root system genotypes on shoot system phenotypes, and here we are treating gene expression in the leaves as a shoot system phenotype. Because we are not testing explicit hypotheses about any particular gene or pathway, we determined that PCR-based confirmation would not add value. This decision was not made lightly, and was done following consideration of other recent work that applied various RNAseq platforms to address structurally similar questions, including:
1) Griffith M, Griffith OL, Mwenifumbo J, Goya R, Morrissy a S, et al. (2010) Alternative expression analysis by RNA sequencing. Nat Methods 7: 843–847. Doi:10.1038/nmeth.1503.
2) Asmann YW, Klee EW, Thompson EA, Perez E a, Middha S, et al. (2009) 3′ tag digital gene expression profiling of human brain and universal reference RNA using Illumina Genome Analyzer. BMC Genomics 10: 531. Doi:10.1186/1471-2164-10-531.
3) Wu AR, Neff NF, Kalisky T, Dalerba P, Treutlein B, et al. (2014) Quantitative assessment of single-cell RNA-sequencing methods. Nat Methods 11: 41–46. Doi:10.1038/nmeth.2694.
4) Shi Y, He M (2014) Differential gene expression identified by RNA-Seq and qPCR in two sizes of pearl oyster (Pinctada fucata). Gene 538: 313–322. Doi:10.1016/j.gene.2014.01.031.
- Moreover, we point to an excellent blogpost on the same validation-discussion that goes further in to this debate:
http://dave-bridges.blogspot.no/2014/11/validation-of-rnaseq-experiments-by-qpcr.html?m=1
- We agree that it adds value to ensure that our data show previously established patterns of house-keeping and circadian-phased genes relevant to our sampling paradigm. As such, the following have been added to the manuscript:
- Added to data description L207 - 210, "To check the validity of our expression results, we assayed two classes of housekeeping gene (Ubiquitin-domain and actin-family) and eight previously annotated

circadian genes (Carbonell-Bejerano et al. 2014)"
- Added to Analyses, L339 - 341, "We computed the expression of two classes of housekeeping genes, and show that they are generally stable (Supplemental Figure 2). We note that some variation is expected for housekeeping genes; see, for example, (Liang et al. 2018). Moreover, we show that patterns of previously annotated circadian genes show expected results over the sampling window. For example, predicted orthologs of LHY and RVE1 are correlated and decreasing over our sampling window, and a predicted TOC1 ortholog is invariant. That our samples showed such patterns suggested the gene expression data presented here were valid."
- Created and added Supplemental Figure (now supplemental Figure 2) showing these patterns.

9. The effect of the different irrigation regimes is not adequately discussed in this manuscript.
- Response: Thank you for this note - good catch. Effects of different irrigation regimes were originally presented in a Supplemental Note hosted on GitHub. To increase accessibility, we moved Supplemental Note 1 from GitHub so that it is now a Supplemental Note to the manuscript.
- Some additional notes: our study site is located in southwestern Missouri where it can be quite rainy. In 2017, the year in which samples were collected for this study, it rained a lot, essentially rendering the vines all properly irrigated despite the amount of irrigation applied as part of the study. Not surprisingly, our physiological metrics showed little/no evidence of stress. As such, we opted to include irrigation as a term in the model that is there and could impart variation into some phenotypes, but those effects are very small.

10. The Abstract must be structured into three separate sections: Background; Results; Conclusions.
- Response: The abstract has been restructured to meet the suggested format. We thank the reviewer for catching this oversight.

Minor comments
1. A lot of the information under "data description" should be moved to the methods section. For me, the data description should provide more of a background and rationale of the work, while the methods should provide the actual steps that were taken.
- Response: Thank you for this note. We struggled to balance the need to provide enough information for readers to understand the work up front, while saving the majority of methodological details for the methods section. In its current version, we hope that we have provided the appropriate information in the approximately preferred locations according to the journal's instructions. If there are persistent issues with information placement in the current version of the manuscript, we would be happy to address those in whatever way the editor/reviewers request.

2. Although it is alluded to in the introduction and data description, the tissue type that was harvested and used for the RNAseq experiments is not mentioned in the methods or analyses sections.
- Response: Good catch, and we regret this omission. The tissue type used for RNAseq experiments were young, fully opened leaves. We added information about the tissue type in the analysis section, and assured it was explicitly mentioned in the data description.
- L185 starts "The youngest fully-opened leaves"
- L336 added "youngest fully-opened leaves"

3. The Figure 1B legend should denote what Y, M, and O mean. I realise that it is young, middle and old, but the legend should stand alone.
- Response: Another good catch. We added a short key to L705 - 706 indicating Y (young), M (middle), and O (old).

4. Significant differences in Fig 1B and Fig 5 B&C could be annotated within the Figure, for example with an asterisk.
- Response: Excellent suggestion. Significant comparisons have been labelled with letters in the identified figures, and the figure legends have been edited to explain them.

5. Please be careful to use the past tense consistently, for example P16, L403 "correlation between gPC4 and pPC3 is similar" should be 403 "correlation between gPC4 and pPC3 was similar".
- Response: Thank you for this important catch. Tense was changed in the cited examples and edited throughout the manuscript.

6. P19, L472 "stomatal conductance were higher vines" should be "stomatal conductance were higher in vines".

- Response: Added ‘in’ to L512

7. P19, L475 "Understanding of rootstock genotype influence shoot system phenotypes" should be "Understanding of how rootstock genotype influence shoot system phenotypes".
- Response: Edited L516 to read, “Understanding rootstock genotype influence on shoot system phenotypes”

8. Perhaps consider re-writing the title to the Fig 5 legend. "Vine physiology measurements show signal from most experimental manipulation" does not make sense to me.
- Response: Edited title on L746 to “Vine physiology varies with rootstock and the rootstock by phenology interaction”

Nice work.
Thank you for this. We appreciate the detailed review.

Reviewer #3
This study investigate associations between rootstock genotype and shoot system phenotypes using five multi-dimensional approaches contributing to elucidate how root systems influence vine phenotype.

the influence of rootstock on the traits analyzed are roughly well documented in literature and authors are aware about this since they very often commented that results are consistent with previous study. Hence the reader might question about the limited new information provided. I would recommend the authors at the "potential implications" paragraph to avoid speculation on "yield" and to emphasis the novelty of engaging a simultaneously analysis as they did in order to speed up comparative studies.
- Response: This is a very helpful suggestion. In response to this and comments from other reviewers we have re-worked the potential implications section. Other reviewers called for an enhanced focus on yield/viticultural implications; however, we agree with this reviewer’s request to minimize speculation. Consequently, we have attempted to carefully place this work in the context of both basic plant biology and viticulture. If the current revision does not meet the expectations of the reviewer(s) or editors we would be happy to revise further.

Minor comments
1. At line 226-227, check "umol/s" replace with [?]mol s-1 ?
- Response: Unfortunately the symbol the reviewer suggested did not render in the communications (we can’t see it). We have replaced the umol with the more commonly accepted μmol where appropriate. If another symbol is preferred or a different symbol was meant, please let us know and we would be happy to make the requested change.

2. At line 231, is 15 min interval time enough to equilibrate? Considering that usually 30 or 60 min are required (e.g., J.Int.Sci.VigneVin, 2012, 46, n°3, 207-219, See https://urldefense.com/v3/__https://doi.org/10.20870/IVES-TR.2020.3620__;!!K543PA!bnhJBaYGb-6O8nkV-F90YalIxoa2UGVyHkLiToTGXSjDbduO2MrZFPISJayIAoKRcnAWzw$See ISBN 978-90-481-9282-3 at pag 89), please justify your 15 min interval.
- Response: We thank the reviewer for this thoughtful comment. We acknowledge that this is a topic of much debate. A 15 minute equilibration has been used in the past to measure midday stem water potential for tree species, and explicit testing showed that there was little difference between a 10-15 equilibration and a >1hr equilibration in oak trees (https://www.fs.fed.us/psw/publications/documents/psw_gtr184/psw_gtr184_035_ShackelGross.pdf). A more recent study (published after our work was completed) suggests that there is a small effect from different equilibration times in grapevine, but that effect is smaller than the effect from the person operating the pressure chamber (https://doi.org/10.1016/j.agwat.2019.03.026). We have amended the section of the manuscript to include these references for future readers (L254-255).

3. Please note that "old" and "young" communicate leaf age rather than leaf position, what's about top, middle, bottom?
- Response: This has been a topic of much debate on our team and we really appreciate this comment. The designation of “old”, “middle” and “young” stem essentially reflect terminology used by our team since the inception of the project. We totally agree that these terms reflect leaf age rather than position. However, in grapevines these are equivalent because the oldest leaf along a vine is at the bottom of the shoot and the youngest leaves are at the top of the shoot. All things being equal we would readily make this change; however, the current terminology is used in this paper and in many other completed or

ongoing manuscripts being carried out by members of our team. If it is amenable to the editor and the reviewer, we would prefer to retain the “old” “middle” and “young” designation. However, if this is unworkable we will make changes to the language.

4. It is not clear why 1103 P had a very little variability of gs at anthesis compared to other rootstocks, for these plant water status seems to range from well irrigated to deep stressed vines while 1103P vines seem to be all roughly well irrigated.
- Response: We appreciate this observation. It is not immediately clear why vines grafted to 1103P showed such little variation in stomatal conductance at anthesis. Unfortunately we don’t think we can test this with the current study. To investigate this and related questions we completed a greenhouse study with 1103P and other rootstocks grafted with a common scion with an irrigation treatment. This work is in preparation now.

5. Providing VPD data might help to explain why transpiration is low at anthesis (approx. 2.5 mmol m-2 s-1) while gs at anthesis is comparable to that of other sampling time.
- Response: Thank you for this interesting point. We agree that features of the environment (like VPD) will partially explain the differences we see across the time point in this and future studies. Ongoing work is attempting to identify features of the environments that correlate and can explain some of the variation we see in these traits. This is partially undermined by natural season changes, so these relationships are hard to untangle and require a substantial amount of data, much beyond the three time points presented here. However, we appreciate this comment and hope to address this in future works.

6. "leaf position" should also be discussed against "leaf angle" (e.g., https://urldefense.com/v3/__https://doi.org/10.3389/fpls.2020.00595__;!!K543PA!bnhJBaYGb-6O8nkV-F90YalIxoa2UGVyHkLiToTGXSjDbduO2MrZFPISJayIAoLo-b4lwA$) which likely change across the season due to change of soil water availability. was leaf angle accounted for image analysis? Considering that soil moisture reasonably differed at the three stages considered (Fig. 5).
- Response: Thank you for this insightful comment. We agree that leaf angle is important for physiology, and would likely correlate with some of the traits we measured. Unfortunately, leaf angle was not quantified at the time of collection in the field. Leaves were simply chosen from vines that emerged directly from the cordon and had intact young, middle, and old leaves. Leaf scans were completed in the lab after leaves had been removed from the vine, and it was not possible to quantify leaf angle at this time. Having said that, this is an important consideration for future studies and we very much appreciate this observation.

7. Please add the mean leaf water potential and soil moisture values directly in the Fig. 5 panels to help the readers.
- Response: We thank the reviewer for this suggestion on improving our figures. We have added the mean value for reach rootstock/phenology combination in Figure 5 and, for consistency, to Figure 1. Figurel legends have been edited to address these changes on L712 and L751.

Reviewer #4
This 'big data' manuscripts offers a comprehensive snapshot of the grape phenome as influenced by several factors, including ionomics, leaf morphology, physiological data, metabolomics and transcriptomics. The overall scope is ambitious and a step forward for the phenomics community. Overall the paper was well-written and the design and analysis are sound, though I had a few questions below.
- Response: Thank you for these very kind and encouraging words.

I had a few suggestions.
1. Phenomic and phenotypic are used interchangeably, and I would ask they be clearly defined - should they really mean the same thing? What's the difference between a phenome and a phenotype?
- Draft response: Thank you for this important observation. We define “phenomics” to be a “field characterized as the acquisition and analysis of high-dimensional phenotypic data at hierarchical levels, often with an eye toward multiscale data integration” in the introduction. We define a phenotype as a single particular trait (e.g., calcium concentration). As such, we have amended usages of these words to comport with this definition: phenomic (and phenomics) now refer to the joint analysis of multiple data modalites, each of which contain several phenotypes (or a single multi-dimensional phenotype). In addition, we recognize that we were being imprecise with language here, so we have fixed this and other

terms used interchangeably (trait/phenotype, phenotypic/phenomic, and data type/modality) throughout the manuscript.

2. Is the paper considering transcriptomics as phenomics? I know it's a debated issue really, but would be good to state so and why.
- Draft response: Thanks for this comment, like many groups we have spent a lot of time thinking about the question of whether or not the transcriptome is a phenomic modality. In the introduction of this manuscript, we loosely acknowledge phenomics as the field of study concerned with high-throughput data acquisition through multiple simultaneous trait measurements, often requiring advanced computation to analyze and integrate L62-63. Following this definition, we treat the transcriptome as a multi-dimensional phenotype (or that the extent to which a particular gene is expressed at a particular time in a particular place is a measurable trait/phenotype). In the analysis and interpretation of the data in this manuscript, we treat the transcriptome like the other data modalities presented here.

3. Related, phenotype and trait are inconsistently used as detailed below. I recommend to define them and use consistently. This is a huge problem for phenomics and I think prevents clear discussion of the topic.
- Response: We thank the reviewer for this comment on clarity. Throughout the manuscript we have edited the language we used to describe phenotypes to be consistent. In particular, we have edited each usage of ‘trait’ to ‘phenotype. As above, we recognize that we were being imprecise with language, so we have fixed this and other terms used interchangeably (trait/phenotype, phenotypic/phenomic, and data type/modality) throughout the manuscript.
-

4. I had some questions about the experimental design and randomization, detailed in line comments. I'm not sure about the claim of 72 replicates. Maybe it's a question of what should be considered an experimental unit.
- Response: Other reviewers also noted lack of clarity with respect to experimental design, and we appreciate this observationt. A full response to this concern can be found in our response to your comment on L561 (below); which is partially copied here: I think some additional confusion may stem from us using “replicate” as a vague stand-in for both clonal replicates and statistical replicates. To address this, we have amended the language about the four rootstock scion combinations as follows on L617: “Clonal replicates of each of the four rootstock-scion combinations were planted 72 times for a total of 288 vines planted in nine rows”. In addition, we included the specific type of design (split-plot) to this section. Finally, we addressed the number of true replicates in a comment by reviewer 2 concerning RNAseq. The same logic can be used to derive the total number of biological replicates for leaf shape and ionomics at the highest order interactions (4) and for all other phenotypes (2). In the case where the number of biological replicates is two, the estimation and interpretation of effects is minimized due to lack of power.

5. The analysis of individual datasets (or modalities, good word) seems good, and I think the approach to combine into a larger set using the PCA is pretty clever. I still wondered how 'fused' the data really is but can't really think of a better way other than combining all the raw data except then the number of genes and metabolites would just swamp the analysis I guess. Perhaps the authors could articulate why this is a good fusion approach they've used, and perhaps what could be done in the future.
- Response: Thank you for this kind observation and really insightful comment. We considered a larger integrative framework that would include all phenotypes measured in the study. However, as the reviewer identified, this would include a heavy bias toward gene expression (expression data for 24,000+ transcripts) and metabolomics (600+ different features measured) which would likely overpower leaf shape (17 x,y coordinates) and ionomics (20 ions). We felt that the PCA approach allowed us to weigh each modality more evenly in order to see if further integrative efforts were warranted. Based on these high-level results, it looks like integration among modalities is a warranted effort, especially if we could collect more targeted data that could expand mechanistic understanding of observed patterns. However, the scope of these integrative techniques is broad and several papers could likely be written just exploring differences in integration techniques with just a single phenotype, for example, gene expression. We have edited the sentence on L430-431 to reflect this logic: “Within each phenotyping modality, we summarized the primary dimensions of phenotypic variation using PCA (see Methods), so as to not weigh any modality too heavily.”

6. I Biologically, I'd like to see more insights to why these traits matter. How could understanding that these traits change help production? I think some arm waving is warranted. Especially, how is

understanding the correlation among modalities important? One idea is to identify trade-offs and synergisms?
- Response: We thank the reviewer for this suggestion. We modified the language of the potential implications to suggest some ways in which this kind of work could balloon into other phenotypes (not measured for this study) that are more useful to breeding through synergistic relationships (enhancement), trade-offs (constraint), or just simply predictability. Moreover, we maintain that the broadest implication is the notion that there is a strong temporal component to phenotypic expression in long-lived perennial plants and that grafting and rootstock genotype add another dimension to it.

7. Last, I'm happy to see how much data is shared. However, GitHub is not appropriate for sharing data, which should all be on a public repository, including the analysis scripts. I think FigShare has been used for other permanent data, so I recommend to share the scripts there.
- Response: We thank the reviewer for this insight. The note on irrigation, which was initially uploaded to GitHub, has been added as a Supplemental Note to this manuscript. This note will additionally stay on github for easy access. All phenotypic data from the ionome, metabolome, leaf shape, and physiology are on Figshare and the gene expression are on the SRA. In addition, we are in the process of submitting raw metabolomics data to the Metabolights database, as requested by GigaScience.
Line comments and other details follow:

39: In my opinion, the 'hyphens' are not needed in belowground and aboveground.
- Response: From what I can gather, above-ground and aboveground are considered to have the same meaning. We leave this stylistic choice up to the editor.

45: "change"
- Response: L46: changes -> change

46: long sentence with semicolon, consider making that a period, but the use of many interjections make it a little hard to parse still
- Response: Good catch. This sentence has been split into two (now L44).

99: are phenotypes and traits taken to be completely synonymous in this paper? Given that many definitions are used of each, it would be helpful to define. For example, both can be used to describe the 'general' properties like 'eye color' or the specific like 'blue eyes.' Phenotype, in addition, is sometimes used to describe the totality of all trait values in an organism. More careful and exact usage would benefit the paper. For example, trait value can also describe the specific like 'blue eyes' while trait the general 'eye color.' The title of your paper suggests that you additionally consider the phenotype as all traits (or trait values?). Leaf shape is referred to as a phenotype at line 439, so consistent with the 'general trait' definition.
- Response: We agree this was a persistent problem in the initial version of the manuscript. As above, we recognize that we were being imprecise with language, so we have fixed this and other terms used interchangeably (trait/phenotype, phenotypic/phenomic, and data type/modality) throughout the manuscript.

111: what would the difference bet tween phenomic and phenotypic variation? what is the definition of the phenome? phenotypic variation is also used at line 434
- Response: Another good catch. We have edited the language throughout the text so that it is now consistent. We did not actually mean to distinguish between these two things in the highlighted example. As above, we recognize that we were being imprecise with language, so we have fixed this and other terms used interchangeably (trait/phenotype, phenotypic/phenomic, and data type/modality) throughout the manuscript.

115: Were there any hypotheses? Is the intent to be descriptive?
- Response: The goals of this study were to address three questions: 1) what is the influence of root system genotype on shoot system phenotype? 2) How do systems of plant phenotypes vary over the growing season and does rootstock genotype influence this variation? And 3) how do phenotypes covary within and between phenotyping modalities? For clarity, we have enumerated these questions in the Study Design section. L639 - 642.

118: Are the details of the experimental design needed here because of the wonky format of a GigaScience paper with methods at the end? Not your fault, but I find these formats so confusing and redundant since authors try to move methods into other sections to make up for it.

- Response: We appreciate this comment. This was certainly a design choice by us so that the paper could be understood linearly.

139: if this pipeline is capitalized and sort of 'official' - is there a citation or access to details of it?
- Response: This is a standardized pipeline at the Donald Danforth Plant Science Center. The sentences surrounding this line (now L140-146) have been restructured to make this more clear: "Between 20 and 100 mg of leaf tissue was acid digested and 20 ions were quantified using inductively coupled plasma mass spectrometry (ICP-MS) following standard protocol of the Donald Danforth Plant Science Center (DDPSC) Ionomics Pipeline [30,31]. Ion quantifications were corrected for internal standard concentrations, instrument drift and by initial sample mass. The output of the Pipeline contained measures for each of the following 20 elements: Al, As, B, Ca, Cd, Co, Cu, Fe, K, Mg, Mn, Mo, Na, Ni, P, Rb, S, Se, Sr, and Zn."

140: Why the difference for ML?
- Response: Z-scores were used in the linear models for ion concentrations so that models could be compared. However, the random forest is a single model that needs no adjustment on the input space. We included a small comment that non-standardized input is the convention for random forests (however many ML models do need to be standardized to equally weight each feature).

141: This Leaf Ionomics section, to me, describes the method to sample and measure, but fails to describe the final output? How many ions? which? I don't fully understand why GigaScience requests this format, but it does mention the background should be given. SO, I think you should say why the ionome is important, and the same for other trait conglomerates mentioned in the paper.
- Response: This is helpful - thank you. We have added the following:
- to L145-146 to explain the ionomics data set, "The output of the Pipeline contained measures for each of the following 20 elements: Al, As, B, Ca, Cd, Co, Cu, Fe, K, Mg, Mn, Mo, Na, Ni, P, Rb, S, Se, Sr, and Zn."
- To L179 to explain the metabolomics data set, "The 661 identified metabolomic features..."

143: carbon-based molecules? For example, not nutrient ions?
- Response: Added "mostly organic" to enhance description of the metabolome

144: I had to look up 'veraison' - could you put 'ripening' in parentheses if that captures that idea?
- Response: Clarified as the "onset of fruit ripening" on L153

210: scanning details? background, color, DPI, image format?
- Response: L228 - 229: added "in color against a white background at 1200 DPI and written as JPEG formatted images".

236: recommend to again announce the number of ions analyzed
- Response: Added "and measured the concentrations of 20 ions" to L262

244: It's not clear what the percentage refers to, I imagine percentage of total variation accounted for by that factor, ie the effect size. recommend to include 'effect size'
- Response: Added variation explained to first usage

249: Giving the effect sizes is a reasonable summary given your multiple factors, however I think giving some indication of absolutely changes is also relevant? Like, what type of percent changes were observed across all the samples in absolute terms, or give the min and max for some ions? Obviously you can't be exhaustive, but this would put the effect size in some type of context of biological influence, like rootstock explaining 10% of variation in a 1% change in Ca vs a 100% change in in Ca. Hope that's understandable. Perhaps these absolute changes would be most relevant where you highlight the influence of rootstocks?
- Response: Thanks for this thoughtful comment. The value of effect sizes like percent variation explained are that they can be directly compared if the models are parameterized in an identical manner. However, I think this confusion could be clarified by projecting samples back into a real concentration space which has now been done for the figure.

267: Could MDA be spelled out on first mention?
- Response: L293 now includes Mean Decrease in Accuracy. It is also defined in the methods.

350: personal placeholder to check discussion for how so much variation isn't accounted for - seems

surprising!
- Response: We agree that the lack of variation explained in the models for leaf shape is quite interesting. Future work will certainly explore factors such as variation imparted from individual vine and environmental variation to attempt to explain this.

400: I'm confused that the PCs should correlate from the same modality, something which I thought didn't usually happen?
- Response: There is statistical literature on this topic. In short, principal components are orthogonal, however orthogonal does not always mean uncorrelated. See Rodgers, Nicewander, and Toothaker, 1984.

462: Good to bring up the biological implications - what are they? Are these changes relevant for growth, taste, etc?
- Response: We agree that the earlier version of this manuscript was missing key information about why the ionome is important. Unfortunately, there is not a lot of work tying together the elements of the ionome that we identified as responsive to rootstock genotype. Traits of biological interest, features that are known to be influenced by ion uptake by the root system (rootstock) are now mentioned in the manuscript, including vine growth and fruit/wine quality. We have added a comment on this in the discussion to address that there is a known connection between macronutrients and these traits, but more work is needed is to uncover these connections with micronutrients in grapevine. To this end, we added the following to L518 - 520: "To our knowledge, there is not yet a strong causal link between the micronutrient component of the ionome and factors of vine growth or development that might influence traits like wine quality. However, it is noted that macronutrient deficiencies can have negative effects on such traits (Bravdo 2000; Brunetto et al. 2015) and can be mediated by rootstock (Gautier et al. 2018). This suggests a strong understanding of the rootstock influence on the vine's ionome is warranted, and more work needs to be done to establish these relationships"

474: Can stomatal conductance be limited by flow in the roots? Do you think it's more likely such an indirect effect, or a direct effect such as signals from the rootstock actually change the rates by stomatal closure, etc?
- Response: This is a really good question that is particularly challenging to fully address. We would wager that root architecture is a key driver of physiological variation, and we tested this in a greenhouse study the results of which are in preparation now. However, it is worth noting signals could be passed from the rootstock, but the space of signal passing through graft junctions is complex and this work was not designed to address anything to that end.

501: I think here, rather than 'phenotype' as 'traits' you meant to say something about the 'data types,' which you referred to as modalities before and would be appropriate to use here. 'traits of different modalities'. I would suggest this instead of 'data types' perhaps for consistency. Definitely it would be a benefit to the field to have ways to describe these 'groups' of traits from the same instrument, my lab also runs into this with fused, multi-dimensional data.
- Response: Great observation, and we agree. We have fixed this instance of improper usage and we have clarified the language in the discussion (and the rest of the manuscript) to be more consistent. Specific to this comment, 'data type' has been replaced with modality/ies throughout the piece. As above, we recognize that we were being imprecise with language, so we have fixed this and other terms used interchangeably (trait/phenotype, phenotypic/phenomic, and data type/modality) throughout the manuscript.

502: latent phenotypes were mentioned in the definition of phenomics (where I only see them as one possibility, not a defining feature). Some definition would be useful.
- Response: This is a good catch. We did not mean to imply latent phenotypes were the only possible outcome of this work. This sentence was expanded a bit to include that idea that latent structure is one possibility, but using this to target integrative analysis is also a strong possibility. (L565)

510: back to phenomic correlation - what's the difference with phenotypic correlation?
- Response: Good catch of this persistent issue in the earlier version of the manuscript. We did not mean to imply there was a difference, or to make any kind of statement on this distinction. As above, we recognize that we were being imprecise with language, so we have fixed this and other terms used interchangeably (trait/phenotype, phenotypic/phenomic, and data type/modality) throughout the manuscript.

533: Very little information was provided about how the changes measured here in traits would affect

yield or other consumer-facing traits. Not only that, but why is the multi-dimensionality important? Does it reveal trade-offs in traits, for example? I'm trying to help you improve the biological impact component. Some arm waving may be warranted.
- Response: We very much appreciate the direction the reviewer is going here, and we have attempted to address this in the potential implications section of the manuscript and elsewhere. Multi-dimensional data are data that consist of many different observations (for example, the ionome which includes measurements of 20 different ions). Multi-dimensional data offer more robust, approaching comprehensive observations of plant phenotypes. They offer a rich source of information that can be used to more comprehensively understand the basic biology of the organism - for example, how root systems influence features of shoot systems in grafted plants. This is described in, for example, L94-100 of the introduction. The influence of the phenotypes we measured on yield or other consumer facing traits are under active investigation. For example, ongoing work by others members of our project team describes berry chemistry and wine volatiles for the experimental vineyard described here. The volume of data was so large; this manuscript represents the first step in processing and interpreting multiple multi-dimensional phenotypes and trying to understand what approaches can be used to understand how they relate to one another. The next steps will be to connect these data with observations that might be more directly relevant to viticulturists. Our hope is that this manuscript will provide the foundation for those analyses that integrate multi-dimensional data from different organ systems, such as leaves and berries.

457: This note is very thorough and appreciated, however a github link is not permanent and therefore I suggest to include as supplemental to this paper or else place on a 'permanent' public repository such data dryad, Zenodo, etc. If the irrigation factor was ignored, you should say so.
- Response: Good point. The note on irrigation has been added as a Supplemental Note to this manuscript. Irrigation was treated as an additional blocking factor in the analyses done here. While we will keep the other data available on Figshare, we are exploring other homes for the data that are in line with GigaScience’s preferences.

561: After reading this section, I wasn't sure about the experimental design, especially what type of randomization was used. I would guess that an appropriate design here would have been split plot block design taking into account irrigation (which I guess you are saying you ignored in the end). Were genotype randomized? the groups of 4 are mentioned, should that be taken as the experimental unit? I'm not super picky about stats, but some might say there are flaws here, and perhaps the 72 should be divided by 4 as as far as complete replicates? In Supp Fig 1 in the map, I see up to Block F - so should it be 6 true replicates? In cases likes this, I usually think of the additional plants as subreplicates. Your design seems basically just like a annual crop field trial with small plots with multiple plants. We usually measure a trait on those subreps then average it to the plot level for further analysis. In that case, the subreplication isn't used in stats directly, but does allow a better approximation of the value for each plot and decrease overall 'random' or 'environmental' error.
- Response: This is a great catch by the reviewer. We regret that the earlier version of this manuscript did not fully explain the experimental design of the research vineyard used in this study. These details have been filled in in section Study Design of the manuscript. Further, I think some additional confusion may stem from us using “replicate” as a vague stand-in for both clonal replicates and statistical replicates. To address this, we have amended the language about the four rootstock scion combinations as follows on L617: “Clonal replicates of each of the four rootstock-scion combinations were planted 72 times for a total of 288 vines planted in nine rows”. In addition, we included the specific type of design (split-plot) to this section. Finally, we addressed the number of true replicates in a comment by reviewer 2 concerning RNAseq. The same logic can be used to derive the total number of biological replicates for leaf shape and ionomics at the highest order interactions (4) and for all other phenotypes (2). In the case where the number of biological replicates is two, the estimation and interpretation of effects is minimized due to lack of power.

Close
