# Supplementary material for: Multi-dimensional leaf phenotypes reflect root system genotype in grafted grapevine over the growing season
Source: Gigascience. 2021 Dec 29;10(12):giab087. doi: 10.1093/gigascience/giab087 (PMC8716362; doi:10.1093/gigascience/giab087)
Supplement: giab087_Response_to_Reviewer_Comments_Revision_1 [file giab087_response_to_reviewer_comments_revision_1.pdf]

Author's Response To Reviewer Comments

Close

Note to all: Microsoft Word on macOS does not allow correct continuous line numbering with "track changes" on. All referenced line numbers were identified such that they were continuous. If line numbers appear way off, try changing "All Markup" to "Simple Markup" under the Review tab to align the line numbers.

All revisions from this round are labelled Revision_2.

Editor Comments:
With regards to Reviewer #4 comments and Github - if the manuscript is deemed acceptable for publication, GigaScience will always take snapshots and host that, along with other supporting data and metadata under a CC0 license. So despite the reviewer's' concern about GitHub not being a permanent repository, there will be copies permanent in our open repository, GigaDB.
Response: We thank the editor for their work on this manuscript. We are happy to have additional copies of all of our scripts and data sets hosted redundantly across multiple repositories. Our intention with GitHub was to store the analysis scripts as permanent versions of record. As we do not come from software development, we were using GitHub as a convenient home rather than as a live repository for ongoing projects. One additional note: we uploaded all metabolomics data to Metabolites, but have not received a response from that submission. We would be happy to store an additional copy on GigaDB, if appropriate.

Reviewer 1:
Relevant methodological information that was missing from the previous submission has been added to the revised manuscript by Harris and co-workers, which enables a more conscious interpretation of the results. Experimental limitations and external sources of variation have also been considered when discussing the results. In addition, cross-check of expected expression profiles for a selection of genes has been included as a validation of the RNA-seq experiment reliability.
Response: We thank the reviewer for their careful review and re-review of the manuscript. Comments made by reviewers have considerably strengthened the manuscript and we really appreciate it.

Considering all the information, despite a huge multilevel dataset was generated, its value is limited by experimental design deficiencies recognized by the authors (e.g.: only one year of study under field conditions, noise of environmental/circadian variation during extensive physiological phenotyping and RNA-seq sampling throughout relatively long periods of the day, theoretically low power of the RNA-seq experiment due to relatively low read depth and low replication in some comparisons with only two replicates). Altogether, the manuscript is mostly descriptive of general differences rather than conclusive. Some of the main observations have already been documented before, such as the idea that rootstock genotype affects scion leaf phenotypes. Regardless, in the current version of the manuscript, the study and its limitations are fairly presented by the authors in a manner that would be acceptable for publication if the journal considers the dataset of value in spite of these experimental limitations.
Besides this general concern, I would only have a few minor comments to this version:

1. The dataset might still be undermined as only general descriptive differences are presented as conclusions, but nothing about their possible origin is mentioned. For instance, what are the known intrinsic features of the compared rootstocks according to the bibliography that could determine the observed differences in ionic composition? How could these rootstock-determined differences in ion accumulation affect vine performance? Similar questions would arise for other differences observed.
- Response: Thank you for this comment, and we share a strong interest in understanding intrinsic features of rootstocks that affect the observed differences in the grafted scion. Studies that begin to get at these questions are underway within our research team now, but unfortunately are not completed and not included in this manuscript. To address the reviewer comments here, we specified that, especially in the case of the ionome, the differences are likely due to the genetics/ pedigree of the rootstock on L521-523. Additional comments added in the last round of revision explain how we are presently unsure how individual ions map to aspects of vine performance. We know even less about the other phenotypes.

Future analyses using the data set we presented, additional data that were beyond the scope of leaf phenotyping, and future data can and should address this type of question.

2. It could be more specifically pointed out that lack of DEGs in some RNA-seq comparisons could be due to the experimental limitations (e.g.: low replication and 4.1 M read depth below the minimum recommended 5 M) rather than to a real lack of effect of rootstock genotype.
- Response: Agreed. We added a note to the Data Description that we opted to sequence more samples at the cost of some read depth which does limit our power to detect some low-expression genes on L195-196. We recognize that replication is low for high order interactions (rootstock:row:phenology) due to only sampling two vines per cell. Because of this low replication, we did not interpret such effects because they would be underpowered. However we sampled 36 cells at each time point for a total of 216 samples (with a few removed for poor sequencing), so lower order interactions and main effects were derived from much larger pools of clonally replicated samples. Specific details on this can be found in response to Reviewer 2 and 4 in the first revision.

3. The value of including PC covariation networks would be scarce if the results are not reliable enough for interpreting the inter-connection identified between the responsible specific metabolites, ions, genes, etc.
- Response: It’s true, and we agree that any issues present in individual data sets will percolate into integrative analyses. Having said that, we are confident in the individual datasets and in our approach using those datasets in PC covariation networks. Focusing on PCs from each modality allowed us to capture the highest levels of variation to see how those PCs relate across modalities. We chose this analysis so that no particular modality was over-weighted and so that we could narrow down where interesting correlations lie such that we can design and craft better future studies. We recognize this approach has limitations, but after exploring many different potential options we felt this was the most appropriate given the data and the questions.

4. Several typos should be corrected in the newly added text.
- Response: We thank the reviewer for the close reading of the text. We have edited the manuscript for typos, grammar, and tense.

Reviewer 2:
I was pleased to review the resubmitted manuscript by Harris and co-workers, who have responded to my original review. The Authors have clarified a number of points regarding the RNAseq experiments including RNA extraction methods, and the tissue type that was used. More information has been added to the methods that would aid reproducibility. Additional statistics have been applied to Figures 1 and 5. Numerous formatting and grammatical changes have been made that improve the readability of the manuscript. Additional supporting references have been provided. While not all of my suggestions were included, I accept the authors responses to my original review. I have no further concerns and recommend the manuscript for publication in GigaScience.
- Response: We thank the reviewer for their careful considerations of our manuscript. The manuscript has been considerably improved thanks to the reviewer’s comments.

Reviewer 3:
I found that the Authors clearly improved the ms which might be suitable for publication
- Response: We thank the reviewer for their careful considerations of our manuscript. We especially thank the reviewer for comments on improving figures. The presentation of our work was improved by the reviewer’s comments.

Reviewer 4:
- I saw the editor comments about appropriate data storage, but I disagree with those comments to the authors.
- Github is not a permanent repository and as such it's not true that it's the most appropriate place to share scripts for a publication. It is only suitable as a place for collaboration. As the authors make changes, the version of record for this manuscript will no longer be available, and the authors could delete it at any time. The publication versions should be separately reposited in a permanent repository. In my opinion, if a script is meant to be a version of record and also living, then a link to both the permanent repository and to GitHub can be given.
- I am not sure what is meant by 'large-scale' data. Figshare is a general use repository that I only recommended since the authors already were using it. It can host single files up to 5 gb in size, provides unlimited public space, and provides a DOI. So what exactly is unsuitable?

- Zenodo is another free option, and there is Data Dryad and the Data Commons.

- Response: We thank the reviewer for their careful considerations of our manuscript. We are happy to share our data and scripts in any way requested. Our intention was to use Github as a repository for a version of record, but we recognize that it is not a perfect solution. We are happy that Gigascience will host snapshots so that there is no potential for misuse. If the reviewer would like an additional home for the scripts we would be very happy to do that.

Close
