# Supplementary material for: Multi-dimensional leaf phenotypes reflect root system genotype in grafted grapevine over the growing season
Source: Gigascience. 2021 Dec 29;10(12):giab087. doi: 10.1093/gigascience/giab087 (PMC8716362; doi:10.1093/gigascience/giab087)
Supplement: giab087_Response_to_Reviewer_Comments_Revision_2 [file giab087_response_to_reviewer_comments_revision_2.pdf]

Author's Response To Reviewer Comments

Close

1. We have added information into the Code Availability Section so that it now meets the required format.
2. All figshare links were added to the references. Additionally, the GigaDB citation was added to the references. These are all referenced in the Availability of Data section. We also lightly edited the Availability of Data Section to be less redundant.
3. Abbreviations, Competing Interests, and Funding information has all been added/moved to the appropriate sections.

Close
