# Supplementary material for: Multi-dimensional leaf phenotypes reflect root system genotype in grafted grapevine over the growing season
Source: Gigascience. 2021 Dec 29;10(12):giab087. doi: 10.1093/gigascience/giab087 (PMC8716362; doi:10.1093/gigascience/giab087)
Supplement: giab087_Reviewer_1_Report_Original_Submission — Pablo Carbonell-Bejerano -- 5/30/2021 Reviewed [file giab087_reviewer_1_report_original_submission.pdf]

Reviewer Report

Title: Multi-dimensional leaf phenotypes reflect root system genotype in grafted grapevine over the growing season

Version: Original Submission **Date:** 5/30/2021

Reviewer name: Pablo Carbonell-Bejerano

Reviewer Comments to Author:

The manuscript by Harris and co-workers presents a characterization of rootstock genotype effects on multilevel leaf phenotypes of one grafted grapevine scion cultivar. Three rootstock genotypes along with the ungrafted cultivar were compared for ionomics, transcriptomics, metabolomics, leaf morphology and physiology in three phenological stages. Analytical and statistical analyses applied were generally sound. While authors identify larger effects in most cases for stage and vineyard position/sampling time, ionic composition was the phenotype most significantly affected by rootstock genotype. Co-variance among multilevel phenotypes is also presented.

Major issues:

1. The study comprises a vast dataset, with a total of 288 plants independently analyzed for two phenotypes (morphometry and ionomics) and 72 plants were used for the rest of phenotypes. Nevertheless, the experiment is limited in terms of genotypes tested and reproducibility. Only one year of study and under the specific soil and climate conditions of a single field plot. Moreover, the effects were only tested on a single scion genotype, a bred interspecific hybrid including Vitis riparia and V. rupestris in its pedigree. At least one of these species is also in the pedigree of the three rootstocks tested, which might involve lower diversity than in common interactions between rootstocks and V. vinifera cultivars. These limitations should at least be considered when discussing the results.

2. The RNA-seq assay did not identify differentially expressed genes (DEGs) in response to rootstock genotype, which disagrees with previous reports. While the origin of the lack of effects here is unclear, further data should be provided to understand the reliability of the RNA-seq experiment:

2.1. Which was the timing of sample collection for RNA-seq samples? Was the same sampling order followed for the three phenological stages? Which were the weather conditions on each of the three sampling dates? It is relevant to describe that information since environmental and circadian changes between and within days can alter gene expression.

2.2. Data on RNA sequencing depth should be provided to understand the resolution of the transcriptomics experiment. For instance, how many bases/reads per sample were produced? How many genes per sample were called as expressed (DESeq2-norm counts >2 according to authors own threshold)?

3. The interpretation of the origin of the results is generally shallow and several questions or limitations are overlooked. For instance:

3.1. It is described that physiological parameters were measured from 10 am to 1 pm, a wide interval with expected changes in environmental conditions affecting these measurements. To understand for possible covariances, it should be indicated if these measurements were carried out simultaneously and

following the same order than that of leaf sample collection for the other phenotyping.
3.2. Related to the previous, why block effect alone was not considered for physiological measurements in Figure 5?
3.3. Did the horseshoe shape for row effect on the transcriptome correlate with oscillation of environmental/circadian clock conditions during the sampling interval or with vineyard heterogeneity? Functional analysis of the genes contributing to row effect could be informative on the origin of these effects that might have hindered the identification of rootstock effect on the transcriptome.
3.4. Is there a rootstock effect on vigor, biomass, fruit fertility and production that could explain or condition the effects in leaf phenotypes that were measured? Were these factors normalized in any way, either by agronomic practices or statistical treatment?
4. This study comprises similar experiments to these already published by the same group in the same set of plants (Migicovsky et al., Hort Res 2019), although extended to include metabolomics and physiology data and two additional phenologcal stages. While the effect of phenology is clearly presented here, the addition of the metabolite data is undermined. What are the metabolites determining rootstock effect in Figure 2C? What about metabolites determining a rootstock effect depending on phenology that could be inferred from PC10?
Minor revisions:
1. "Ubiquitous" effects of rootstock genotype are described along the MS. However, since only one location was analyzed (leaves), would "pleotropic" be more appropriate to define the different phenotypes affected by rootstock-scion genotype interaction in this study?
2. Methods on RNA-seq procedures are incomplete. Which sequencing technology was used? Which type and length of reads? Etc.
3. Inter-annual comparison for anthesis ionomics, transcriptomics and morphology between this study and their previous publication (Migicovsky et al., Hort Res 2019) could enable a broader interpretation of rootstock effects, overcoming the reproducibility limitation of considering only a single season here.
4. L426. The sentence might not be completely fair as no DEG was identified for rootstock effect (transcriptome phenotype would therefore be mostly unaffected) and developmental stage-specific could be more adequate than season-specific.
5. Any biological interpretation of the specific metabolites, genes, iones, shapes determining the resulting PC covariation networks? While it can be interesting to add to covariation networks additional levels of phenomics as authors propose (lcRNA, micorobiome, epigenetics), it would also be informative to exploit the interpretation of the dataset that they have already produced.
6. L470, If the lack of rootstock effect on the transcriptome was due to the phenology effect, specific analysis at each phenology stage would identify rootstock genotype factor significant DEGs. Is it the case? Would there be any rootstock effect detected on transcriptome if the analysis was restricted to single blocks at specific phenological stages?
7. Apart from the seasonal effect, the "Potential implications" presented are not directly inferred from the Results obtained here but from the potential of the approach used. Any other potential implication of the specific results?
8. Is there any data available for the distribution of soil properties across the experimental plot that could be considered to discuss the origin of block effects? Could the human factor during that extensive sampling be another variable accounting for block effect?

9. Because half of 3309C reps would have been collected before any ungrafted rep was taken, could the LD2 effect in discriminating 3309C and ungrafted from RNA-seq data be related with sampling times? What are the genes involved in this effect?
10. Any discussion on the origin of leaf position effects in specific ions?
11. L556. Indicating in there that "only the middle two vines of the four cells in the front half of the vineyard were included in the 72-vine set" would be handy to understand the distribution of this set.

Methods

Are the methods appropriate to the aims of the study, are they well described, and are necessary controls included? Choose an item.

Conclusions

Are the conclusions adequately supported by the data shown? Choose an item.

Reporting Standards

Does the manuscript adhere to the journal's guidelines on minimum standards of reporting? Choose an item.

Choose an item.

Statistics

Are you able to assess all statistics in the manuscript, including the appropriateness of statistical tests used? Choose an item.

Quality of Written English

Please indicate the quality of language in the manuscript: Choose an item.

Declaration of Competing Interests

Please complete a declaration of competing interests, considering the following questions:

- Have you in the past five years received reimbursements, fees, funding, or salary from an organisation that may in any way gain or lose financially from the publication of this manuscript, either now or in the future?
- Do you hold any stocks or shares in an organisation that may in any way gain or lose financially from the publication of this manuscript, either now or in the future?
- Do you hold or are you currently applying for any patents relating to the content of the manuscript?

- Have you received reimbursements, fees, funding, or salary from an organization that holds or has applied for patents relating to the content of the manuscript?
- Do you have any other financial competing interests?
- Do you have any non-financial competing interests in relation to this paper?

If you can answer no to all of the above, write 'I declare that I have no competing interests' below. If your reply is yes to any, please give details below.

I declare that I have no competing interests

I agree to the open peer review policy of the journal. I understand that my name will be included on my report to the authors and, if the manuscript is accepted for publication, my named report including any attachments I upload will be posted on the website along with the authors' responses. I agree for my report to be made available under an Open Access Creative Commons CC-BY license (http://creativecommons.org/licenses/by/4.0/). I understand that any comments which I do not wish to be included in my named report can be included as confidential comments to the editors, which will not be published.

Choose an item.

To further support our reviewers, we have joined with Publons, where you can gain additional credit to further highlight your hard work (see: https://publons.com/journal/530/gigascience). On publication of this paper, your review will be automatically added to Publons, you can then choose whether or not to claim your Publons credit. I understand this statement.

Yes Choose an item.
