# Supplementary material for: Multi-dimensional leaf phenotypes reflect root system genotype in grafted grapevine over the growing season
Source: Gigascience. 2021 Dec 29;10(12):giab087. doi: 10.1093/gigascience/giab087 (PMC8716362; doi:10.1093/gigascience/giab087)
Supplement: giab087_Reviewer_1_Report_Revision_1 — Pablo Carbonell-Bejerano -- 10/17/2021 Reviewed [file giab087_reviewer_1_report_revision_1.pdf]

Reviewer Report

Title: Multi-dimensional leaf phenotypes reflect root system genotype in grafted grapevine over the growing season

Version: Revision 1 **Date:** 10/17/2021

Reviewer name: Pablo Carbonell-Bejerano

Reviewer Comments to Author:

Relevant methodological information that was missing from the previous submission has been added to the revised manuscript by Harris and co-workers, which enables a more conscious interpretation of the results. Experimental limitations and external sources of variation have also been considered when discussing the results. In addition, cross-check of expected expression profiles for a selection of genes has been included as a validation of the RNA-seq experiment reliability.

Considering all the information, despite a huge multilevel dataset was generated, its value is limited by experimental design deficiencies recognised by the authors (e.g.: only one year of study under field conditions, noise of environmental/circadian variation during extensive physiological phenotyping and RNA-seq sampling throughout relatively long periods of the day, theoretically low power of the RNA-seq experiment due to relatively low read depth and low replication in some comparisons with only two replicates). Altogether, the manuscript is mostly descriptive of general differences rather than conclusive. Some of the main observations have already been documented before, such as the idea that rootstock genotype affects scion leaf phenotypes. Regardless, in the current version of the manuscript, the study and its limitations are fairly presented by the authors in a manner that would be acceptable for publication if the journal considers the dataset of value in spite of these experimental limitations.

Besides this general concern, I would only have a few minor comments to this version:

1. The dataset might still be undermined as only general descriptive differences are presented as conclusions, but nothing about their possible origin is mentioned. For instance, what are the known intrinsic features of the compared rootstocks according to the bibliography that could determine the observed differences in ionic composition? How could these rootstock-determined differences in ion accumulation affect vine performance? Similar questions would arise for other differences observed.
2. It could be more specifically pointed out that lack of DEGs in some RNA-seq comparisons could be due to the experimental limitations (e.g.: low replication and 4.1 M read depth below the minimum recommended 5 M) rather than to a real lack of effect of rootstock genotype.
3. The value of including PC covariation networks would be scarce if the results are not reliable enough for interpreting the inter-connection identified between the responsible specific metabolites, ions, genes, etc.
4. Several typos should be corrected in the newly added text.

Methods

Are the methods appropriate to the aims of the study, are they well described, and are necessary controls included? Choose an item.

Conclusions

Are the conclusions adequately supported by the data shown? Choose an item.

Reporting Standards

Does the manuscript adhere to the journal's guidelines on minimum standards of reporting? Choose an item.

Choose an item.

Statistics

Are you able to assess all statistics in the manuscript, including the appropriateness of statistical tests used? Choose an item.

Quality of Written English

Please indicate the quality of language in the manuscript: Choose an item.

Declaration of Competing Interests

Please complete a declaration of competing interests, considering the following questions:

- Have you in the past five years received reimbursements, fees, funding, or salary from an organisation that may in any way gain or lose financially from the publication of this manuscript, either now or in the future?
- Do you hold any stocks or shares in an organisation that may in any way gain or lose financially from the publication of this manuscript, either now or in the future?
- Do you hold or are you currently applying for any patents relating to the content of the manuscript?
- Have you received reimbursements, fees, funding, or salary from an organization that holds or has applied for patents relating to the content of the manuscript?
- Do you have any other financial competing interests?
- Do you have any non-financial competing interests in relation to this paper?

If you can answer no to all of the above, write 'I declare that I have no competing interests' below. If your reply is yes to any, please give details below.

I declare that I have no competing interests

I agree to the open peer review policy of the journal. I understand that my name will be included on my report to the authors and, if the manuscript is accepted for publication, my named report including any attachments I upload will be posted on the website along with the authors' responses. I agree for my

report to be made available under an Open Access Creative Commons CC-BY license (http://creativecommons.org/licenses/by/4.0/). I understand that any comments which I do not wish to be included in my named report can be included as confidential comments to the editors, which will not be published.

Choose an item.

To further support our reviewers, we have joined with Publons, where you can gain additional credit to further highlight your hard work (see: https://publons.com/journal/530/gigascience). On publication of this paper, your review will be automatically added to Publons, you can then choose whether or not to claim your Publons credit. I understand this statement.

Yes Choose an item.
