# Supplementary material for: Multi-dimensional leaf phenotypes reflect root system genotype in grafted grapevine over the growing season
Source: Gigascience. 2021 Dec 29;10(12):giab087. doi: 10.1093/gigascience/giab087 (PMC8716362; doi:10.1093/gigascience/giab087)
Supplement: giab087_Reviewer_2_Report_Original_Submission — Sam Henderson -- 6/10/2021 Reviewed [file giab087_reviewer_2_report_original_submission.pdf]

Reviewer Report

Title: Multi-dimensional leaf phenotypes reflect root system genotype in grafted grapevine over the growing season

Version: Original Submission **Date:** 6/10/2021

Reviewer name: Sam Henderson

Reviewer Comments to Author:

The manuscript by Harris et al investigates the effect of grafting on a number of physiological and molecular phenotypes within grapevine (Vitis spp.) scions. The hybrid Vitis cultivar Chambourcin was compared when grown on its own-roots, or when grafted to three different commercial hybrid rootstocks: 1103P, 3309C, and SO4. The vines were grown in the field, irrigated with different volumes of water, and sampled over a single growing season. Large data sets have been generated for leaf metabolites, solutes (ions), transcripts, shape, and physiology (stomatal conductance, transpiration). As such, the manuscript fits the scope of Gigascience well. The manuscript is well written, however I found it was very statistical and would benefit from additional biological analyses to confirm and validate the findings. The methods section is lacking some details that would enable reproducibility. Some of the figures could be improved for readability. My comments and suggestions are detailed below:

Major comments

1. There is no information on the age of the vines at the time of the experiments.
2. A quantitative analysis of the elemental content of the irrigation water by ICP-MS would be beneficial. In this study, it is unknown whether the irrigation treatments contained varying levels of the elements that were measured in leaves. To this end, it is perhaps not surprising that rootstocks had minimal effect on, for example, the Na+ content of grafted scions. However, it has been demonstrated previously that own-rooted vines cannot efficiently exclude Na+ compared to grafted vines when irrigated with 100 mM NaCl (see Fisarakis el al (2001) Agricultural Water Management 51 13-27).
3. The manuscript would be more useful to the plant science community if a subset of the actual metabolites and genes identified within the principle components were named and confirmed using a second method. It would then be possible to discuss which physiological, metabolic, and molecular processes within Vitis scions are impacted by rootstock selection.
4. Similar to my comment above, some of the data could be integrated. For example, transpiration was increased for scions grafted to 1103P (Fig 5B). Were genes or metabolites involved in the regulation of stomatal aperture differentially abundant when grafted to 1103P?
5. The ionomics data in Fig 1B and C would be easier to interpret if presented as a percentage - for example, % DW, % FW, or mM of tissue water. Currently, there are no units on the Y-axis.
6. There is no mention of how the RNA was extracted from plant tissues. Further, a quality control would normally be performed, e.g. by measuring the 260/280 ratios at the very least. Was any quality control performed on these RNA samples? How do we know the samples were pure and not degraded?
7. It is unclear how many biological replicates were used for the RNAseq experiments.
8. Usually, for genome-wide transcriptional studies, the expression patterns of a subset of genes are

confirmed using another method (e.g. quantitative real-time PCR). This has not been performed in this manuscript. Authors need to confirm the validity of the RNA seq dataset.
9. The effect of the different irrigation regimes is not adequately discussed in this manuscript.
10. The Abstract must be structured into three separate sections: Background; Results; Conclusions.
Minor comments
1. A lot of the information under "data description" should be moved to the methods section. For me, the data description should provide more of a background and rationale of the work, while the methods should provide the actual steps that were taken.
2. Although it is alluded to in the introduction and data description, the tissue type that was harvested and used for the RNAseq experiments is not mentioned in the methods or analyses sections.
3. The Figure 1B legend should denote what Y, M, and O mean. I realise that it is young, middle and old, but the legend should stand alone.
4. Significant differences in Fig 1B and Fig 5 B&C could be annotated within the Figure, for example with an asterisk.
5. Please be careful to use the past tense consistently, for example P16, L403 "correlation between gPC4 and pPC3 is similar" should be 403 "correlation between gPC4 and pPC3 was similar".
6. P19, L472 "stomatal conductance were higher vines" should be "stomatal conductance were higher in vines".
7. P19, L475 "Understanding of rootstock genotype influence shoot system phenotypes" should be "Understanding of how rootstock genotype influence shoot system phenotypes".
8. Perhaps consider re-writing the title to the Fig 5 legend. "Vine physiology measurements show signal from most experimental manipulation" does not make sense to me.
Nice work.

Methods

Are the methods appropriate to the aims of the study, are they well described, and are necessary controls included? Choose an item.

Conclusions

Are the conclusions adequately supported by the data shown? Choose an item.

Reporting Standards

Does the manuscript adhere to the journal's guidelines on minimum standards of reporting? Choose an item.

Choose an item.

Statistics

Are you able to assess all statistics in the manuscript, including the appropriateness of statistical tests used? Choose an item.

Quality of Written English

Please indicate the quality of language in the manuscript: Choose an item.

Declaration of Competing Interests

Please complete a declaration of competing interests, considering the following questions:

- Have you in the past five years received reimbursements, fees, funding, or salary from an organisation that may in any way gain or lose financially from the publication of this manuscript, either now or in the future?
- Do you hold any stocks or shares in an organisation that may in any way gain or lose financially from the publication of this manuscript, either now or in the future?
- Do you hold or are you currently applying for any patents relating to the content of the manuscript?
- Have you received reimbursements, fees, funding, or salary from an organization that holds or has applied for patents relating to the content of the manuscript?
- Do you have any other financial competing interests?
- Do you have any non-financial competing interests in relation to this paper?

If you can answer no to all of the above, write 'I declare that I have no competing interests' below. If your reply is yes to any, please give details below.

I declare that I have no competing interests

I agree to the open peer review policy of the journal. I understand that my name will be included on my report to the authors and, if the manuscript is accepted for publication, my named report including any attachments I upload will be posted on the website along with the authors' responses. I agree for my report to be made available under an Open Access Creative Commons CC-BY license (http://creativecommons.org/licenses/by/4.0/). I understand that any comments which I do not wish to be included in my named report can be included as confidential comments to the editors, which will not be published.

Choose an item.

To further support our reviewers, we have joined with Publons, where you can gain additional credit to further highlight your hard work (see: https://publons.com/journal/530/gigascience). On publication of this paper, your review will be automatically added to Publons, you can then choose whether or not to claim your Publons credit. I understand this statement.

Yes Choose an item.
