# Supplementary material for: Multi-dimensional leaf phenotypes reflect root system genotype in grafted grapevine over the growing season
Source: Gigascience. 2021 Dec 29;10(12):giab087. doi: 10.1093/gigascience/giab087 (PMC8716362; doi:10.1093/gigascience/giab087)
Supplement: giab087_Reviewer_3_Report_Original_Submission — Giuseppe Montanaro -- 6/11/2021 Reviewed [file giab087_reviewer_3_report_original_submission.pdf]

Reviewer Report

Title: Multi-dimensional leaf phenotypes reflect root system genotype in grafted grapevine over the growing season

Version: Original Submission **Date:** 6/11/2021

Reviewer name: Giuseppe Montanaro

Reviewer Comments to Author:

This study investigate associations between rootstock genotype and shoot system phenotypes using five multi-dimensional approaches contributing to elucidate how root systems influence vine phenotype.
the influence of rootstock on the traits analyzed are roughly well documented in literature and authors are aware about this since they very often commented that results are consistent with previous study.
Hence the reader might question about the limited new information provided. I would recommend the authors at the "potential implications" paragraph to avoid speculation on "yield" and to emphasis the novelty of engaging a simultaneously analysis as they did in order to speed up comparative studies.
Minor comments
At line 226-227, check "umol/s" replace with ï[illegible]-mol s-1 ?
At line 231, is 15 min interval time enough to equilibrate? Considering that usually 30 or 60 min are required (e.g., J.Int.Sci.VigneVin, 2012, 46, nÂ°3, 207-219,
See https://doi.org/10.20870/IVES-TR.2020.3620
See ISBN 978-90-481-9282-3 at pag 89), please justify your 15 min interval.
Please note that "old" and "young" communicate leaf age rather than leaf position, what's about top, middle, bottom?
It is not clear why 1103 P had a very little variability of gs at anthesis compared to other rootstocks, for these plant water status seems to range from well irrigated to deep stressed vines while 1103P vines seem to be all roughly well irrigated.
Providing VPD data might help to explain why transpiration is low at anthesis (approx. 2.5 mmol m-2 s-1) while gs at anthesis is comparable to that of other sampling time.
"leaf position" should also be discussed against "leaf angle" (e.g., https://doi.org/10.3389/fpls.2020.00595) which likely change across the season due to change of soil water availability.
was leaf angle accounted for image analysis? Considering that soil moisture reasonably differed at the three stages considered (Fig. 5).
Please add the mean leaf water potential and soil moisture values directly in the Fig. 5 panels to help the readers.

Methods

Are the methods appropriate to the aims of the study, are they well described, and are necessary controls included? Choose an item.

Conclusions

Are the conclusions adequately supported by the data shown? Choose an item.

Reporting Standards

Does the manuscript adhere to the journal's guidelines on minimum standards of reporting? Choose an item.

Choose an item.

Statistics

Are you able to assess all statistics in the manuscript, including the appropriateness of statistical tests used? Choose an item.

Quality of Written English

Please indicate the quality of language in the manuscript: Choose an item.

Declaration of Competing Interests

Please complete a declaration of competing interests, considering the following questions:

- Have you in the past five years received reimbursements, fees, funding, or salary from an organisation that may in any way gain or lose financially from the publication of this manuscript, either now or in the future?
- Do you hold any stocks or shares in an organisation that may in any way gain or lose financially from the publication of this manuscript, either now or in the future?
- Do you hold or are you currently applying for any patents relating to the content of the manuscript?
- Have you received reimbursements, fees, funding, or salary from an organization that holds or has applied for patents relating to the content of the manuscript?
- Do you have any other financial competing interests?
- Do you have any non-financial competing interests in relation to this paper?

If you can answer no to all of the above, write 'I declare that I have no competing interests' below. If your reply is yes to any, please give details below.

I declare that I have no competing interests

I agree to the open peer review policy of the journal. I understand that my name will be included on my report to the authors and, if the manuscript is accepted for publication, my named report including any attachments I upload will be posted on the website along with the authors' responses. I agree for my

report to be made available under an Open Access Creative Commons CC-BY license (http://creativecommons.org/licenses/by/4.0/). I understand that any comments which I do not wish to be included in my named report can be included as confidential comments to the editors, which will not be published.

Choose an item.

To further support our reviewers, we have joined with Publons, where you can gain additional credit to further highlight your hard work (see: https://publons.com/journal/530/gigascience). On publication of this paper, your review will be automatically added to Publons, you can then choose whether or not to claim your Publons credit. I understand this statement.

Yes Choose an item.
