# Supplementary material for: Multi-dimensional leaf phenotypes reflect root system genotype in grafted grapevine over the growing season
Source: Gigascience. 2021 Dec 29;10(12):giab087. doi: 10.1093/gigascience/giab087 (PMC8716362; doi:10.1093/gigascience/giab087)
Supplement: giab087_Reviewer_4_Report_Original_Submission — Larry York -- 6/22/2021 Reviewed [file giab087_reviewer_4_report_original_submission.pdf]

Reviewer Report

Title: Multi-dimensional leaf phenotypes reflect root system genotype in grafted grapevine over the growing season

Version: Original Submission **Date:** 6/22/2021

Reviewer name: Larry York

Reviewer Comments to Author:

This 'big data' manuscripts offers a comprehensive snapshot of the grape phenome as influenced by several factors, including ionomics, leaf morphology, physiological data, metabolomics and transcriptomics. The overall scope is ambitious and a step forward for the phenomics community. Overall the paper was well-written and the design and analysis are sound, though I had a few questions below. I had a few suggestions.
Phenomic and phenotypic are used interchangeably, and I would ask they be clearly defined - should they really mean the same thing? What's the difference between a phenome and a phenotype?
Is the paper considering transcriptomics as phenomics? I know it's a debated issue really, but would be good to state so and why.
Related, phenotype and trait are inconsistently used as detailed below. I recommend to define them and use consistently. This is a huge problem for phenomics and I think prevents clear discussion of the topic.
I had some questions about the experimental design and randomization, detailed in line comments. I'm not sure about the claim of 72 replicates. Maybe it's a question of what should be considered an experimental unit.
The analysis of individual datasets (or modalities, good word) seems good, and I think the approach to combine into a larger set using the PCA is pretty clever. I still wondered how 'fused' the data really is but can't really think of a better way other than combining all the raw data except then the number of genes and metabolites would just swamp the analysis I guess. Perhaps the authors could articulate why this is a good fusion approach they've used, and perhaps what could be done in the future.
Biologically, I'd like to see more insights to why these traits matter. How could understanding that these traits change help production? I think some arm waving is warranted. Especially, how is understanding the correlation among modalities important? One idea is to identify trade-offs and synergisms?
Last, I'm happy to see how much data is shared. However, GitHub is not appropriate for sharing data, which should all be on a public repository, including the analysis scripts. I think FigShare has been used for other permanent data, so I recommend to share the scripts there.
Line comments and other details follow:
39: In my opinion, the 'hyphens' are not needed in belowground and aboveground.
45: "change"
46: long sentence with semicolon, consider making that a period, but the use of many interjections make it a little hard to parse still
99: are phenotypes and traits taken to be completely synonymous in this paper? Given that many

definitions are used of each, it would be helpful to define. For example, both can be used to describe the 'general' properties like 'eye color' or the specific like 'blue eyes.' Phenotype, in addition, is sometimes used to describe the totality of all trait values in an organism. More careful and exact usage would benefit the paper. For example, trait value can also describe the specific like 'blue eyes' while trait the general 'eye color.' The title of your paper suggests that you additionally consider the phenotype as all traits (or trait values?). Leaf shape is referred to as a phenotype at line 439, so consistent with the 'general trait' definition.
111: what would the difference bet tween phenomic and phenotypic variation? what is the definition of the phenome? phenotypic variation is also used at line 434
115: Were there any hypotheses? Is the intent to be descriptive?
118: Are the details of the experimental design needed here because of the wonky format of a GigaScience paper with methods at the end? Not your fault, but I find these formats so confusing and redundant since authors try to move methods into other sections to make up for it.
139: if this pipeline is capitalized and sort of 'official' - is there a citation or access to details of it?
140: Why the difference for ML?
141: This Leaf Ionomics section, to me, describes the method to sample and measure, but fails to describe the final output? How many ions? which? I don't fully understand why GigaScience requests this format, but it does mention the background should be given. SO, I think you should say why the ionome is important, and the same for other trait conglomerates mentioned in the paper.
143: carbon-based molecules? For example, not nutrient ions?
144: I had to look up 'veraison' - could you put 'ripening' in parentheses if that captures that idea?
210: scanning details? background, color, DPI, image format?
236: recommend to again announce the number of ions analyzed
244: It's not clear what the percentage refers to, I imagine percentage of total variation accounted for by that factor, ie the effect size. recommend to include 'effect size'
249: Giving the effect sizes is a reasonable summary given your multiple factors, however I think giving some indication of absolutely changes is also relevant? Like, what type of percent changes were observed across all the samples in absolute terms, or give the min and max for some ions? Obviously you can't be exhaustive, but this would put the effect size in some type of context of biological influence, like rootstock explaining 10% of variation in a 1% change in Ca vs a 100% change in in Ca. Hope that's understandable. Perhaps these absolute changes would be most relevant where you highlight the influence of rootstocks?
267: Could MDA be spelled out on first mention?
350: personal placeholder to check discussion for how so much variation isn't accounted for - seems surprising!
400: I'm confused that the PCs should correlate from the same modality, something which I thought didn't usually happen?
462: Good to bring up the biological implications - what are they? Are these changes relevant for growth, taste, etc?
474: Can stomatal conductance be limited by flow in the roots? Do you think it's more likely such an indirect effect, or a direct effect such as signals from the rootstock actually change the rates by stomatal closure, etc?

501: I think here, rather than 'phenotype' as 'traits' you meant to say something about the 'data types,' which you referred to as modalities before and would be appropriate to use here. 'traits of different modalities'. I would suggest this instead of 'data types' perhaps for consistency. Definitely it would be a benefit to the field to have ways to describe these 'groups' of traits from the same instrument, my lab also runs into this with fused, multi-dimensional data.
502: latent phenotypes were mentioned in the definition of phenomics (where I only see them as one possibility, not a defining feature). Some definition would be useful.
510: back to phenomic correlation - what's the difference with phenotypic correlation?
533: Very little information was provided about how the changes measured here in traits would affect yield or other consumer-facing traits. Not only that, but why is the multi-dimensionality important? Does it reveal trade-offs in traits, for example? I'm trying to help you improve the biological impact component. Some arm waving may be warranted.
457: This note is very thorough and appreciated, however a github link is not permanent and therefore I suggest to include as supplemental to this paper or else place on a 'permanent' public repository such data dryad, Zenodo, etc. If the irrigation factor was ignored, you should say so.
561: After reading this section, I wasn't sure about the experimental design, especially what type of randomization was used. I would guess that an appropriate design here would have been split plot block design taking into account irrigation (which I guess you are saying you ignored in the end). Were genotype randomized? the groups of 4 are mentioned, should that be taken as the experimental unit? I'm not super picky about stats, but some might say there are flaws here, and perhaps the 72 should be divided by 4 as as far as complete replicates? In Supp Fig 1 in the map, I see up to Block F - so should it be 6 true replicates? In cases likes this, I usually think of the additional plants as subreplicates. Your design seems basically just like a annual crop field trial with small plots with multiple plants. We usually measure a trait on those subreps then average it to the plot level for further analysis. In that case, the subreplication isn't used in stats directly, but does allow a better approximation of the value for each plot and decrease overall 'random' or 'environmental' error.

Methods

Are the methods appropriate to the aims of the study, are they well described, and are necessary controls included? Choose an item.

Conclusions

Are the conclusions adequately supported by the data shown? Choose an item.

Reporting Standards

Does the manuscript adhere to the journal’s guidelines on [minimum standards of reporting?]() Choose an item.

Choose an item.

Statistics

Are you able to assess all statistics in the manuscript, including the appropriateness of statistical tests used? Choose an item.

Quality of Written English

Please indicate the quality of language in the manuscript: Choose an item.

Declaration of Competing Interests

Please complete a declaration of competing interests, considering the following questions:

- Have you in the past five years received reimbursements, fees, funding, or salary from an organisation that may in any way gain or lose financially from the publication of this manuscript, either now or in the future?
- Do you hold any stocks or shares in an organisation that may in any way gain or lose financially from the publication of this manuscript, either now or in the future?
- Do you hold or are you currently applying for any patents relating to the content of the manuscript?
- Have you received reimbursements, fees, funding, or salary from an organization that holds or has applied for patents relating to the content of the manuscript?
- Do you have any other financial competing interests?
- Do you have any non-financial competing interests in relation to this paper?

If you can answer no to all of the above, write 'I declare that I have no competing interests' below. If your reply is yes to any, please give details below.

None.

I agree to the open peer review policy of the journal. I understand that my name will be included on my report to the authors and, if the manuscript is accepted for publication, my named report including any attachments I upload will be posted on the website along with the authors' responses. I agree for my report to be made available under an Open Access Creative Commons CC-BY license (http://creativecommons.org/licenses/by/4.0/). I understand that any comments which I do not wish to be included in my named report can be included as confidential comments to the editors, which will not be published.

Choose an item.

To further support our reviewers, we have joined with Publons, where you can gain additional credit to further highlight your hard work (see: https://publons.com/journal/530/gigascience). On publication of this paper, your review will be automatically added to Publons, you can then choose whether or not to claim your Publons credit. I understand this statement.

Yes Choose an item.
